# Supplementary material for: Associations between HIV status and self-reported hypertension in a high HIV prevalence sub-Saharan African population: a cross-sectional study
Source: BMJ Open. 2023 Jan 12;13(1):e067327. doi: 10.1136/bmjopen-2022-067327 (PMC9843216; doi:10.1136/bmjopen-2022-067327)
Supplement: Supplementary data [file bmjopen-2022-067327supp001.pdf]

Supplementary material

Supplementary table 1. Reported hypertension (HPN) prevalence by HIV status, age, and sex. 2

Supplementary figure 1. Prevalence of reported hypertension in men and women by age and ART exposure. Error bars: 95% confidence intervals. 3

Supplementary table 2. Prevalence of current antiretroviral therapy usage and adherent current antiretroviral therapy usage among people living with HIV (N=936) 4

Supplementary table 3. Current antiretroviral therapy usage as a determinant of hypertension in people living with HIV. 5

Supplementary table 4. Adherent current antiretroviral therapy usage as a determinant of hypertension in people living with HIV. 7

Manicaland Study Questionnaires. 9

Supplementary table 1. Reported hypertension (HPN) prevalence by HIV status, age, and sex.

Sex	Age	People living with HIV			HIV-negative people			F-statistic	p-value
		Weighted prevalence, % (95% Confidence Interval)	N for those with HPN[1]	N for those with no HPN[1]	Weighted prevalence, % (95% Confidence Interval)	N for those with HPN[1]	N for those with no HPN[1]		
Men	15-24	3.2% (0-8.5%)	1	29	1.1% (0.6-1.6%)	17	1,590	1.3	0.261
Men	25-34	1.5% (0-4.5%)	1	57	2.4% (1.3-3.6%)	18	721	0.2	0.645
Men	35-44	7.1% (1.6-12.6%)	6	80	4.7% (2.8-6.5%)	23	474	0.9	0.343
Men	45-54	3.8% (0.1-7.4%)	4	100	8.8% (5.5-12.2%)	25	261	2.9	0.090
Men	55-64	15.1% (2.7-27.6%)	5	26	11.6% (6.0-17.2%)	15	116	0.3	0.586
Men	65+	6.7% (0-19.3%)	1	18	31.2% (25.3-37.2%)	74	168	4.1	0.043
Women	15-24	10.3% (3.1-17.5%)	7	60	6.4% (5.3-7.4%)	133	1,932	1.7	0.197
Women	25-34	18.1% (11.6-24.7%)	24	107	12.9% (10.8-15.1%)	123	819	2.6	0.104
Women	35-44	16.7 (11.5-21.9%)	33	163	19.4% (16.4-22.3%)	134	555	0.7	0.402
Women	45-54	20.2% (13.8-26.7%)	30	118	30.1% (25.7-34.5%)	124	287	5.4	0.021
Women	55-64	39.1% (24.1-54.0%)	16	25	41.5% (36.0-47.1%)	124	176	0.1	0.763
Women	65+	60.5% (38.5-82.5%)	11	8	57.2% (52.3-62.1%)	233	174	0.1	0.778

[1]Values are unweighted. Individuals without a recorded HIV status or with missing data for hypertension reporting were excluded.

Supplementary figure 1. Prevalence of reported hypertension in men and women by age and ART exposure. Error bars: 95% confidence intervals.

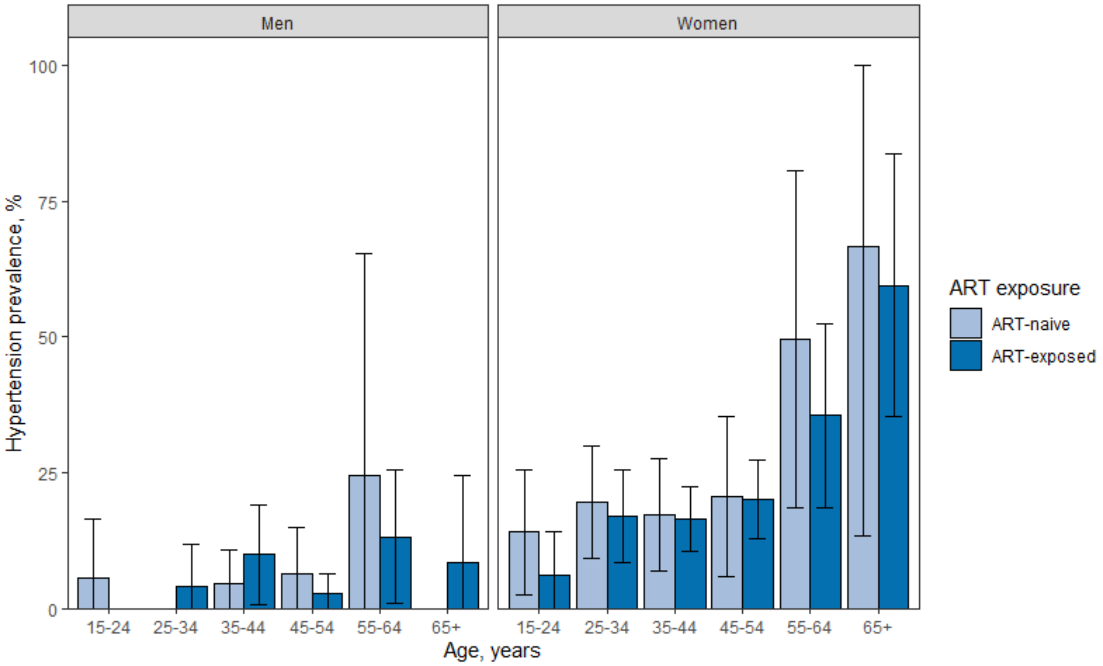

Supplementary table 2. Prevalence of current antiretroviral therapy usage and adherent current antiretroviral therapy usage among people living with HIV (N=936)

	Weighted proportion, % (95% Confidence Interval)	N[1]
**Current ART usage[2]**		
Not using ART	34.5% (31.4%-37.6%)	328
Using ART	65.5% (62.3%-68.6%)	607
Missing	-	1
**Adherent current ART usage[2]**		
Not currently using ART or not adherent to ART	39.8% (36.6%-43.0%)	377
Currently using and adherent to ART	60.2% (57.0%-63.4%)	558
Missing	-	1

[1]Values are unweighted.

[2]ART, antiretroviral therapy.

Supplementary table 3. Current antiretroviral therapy usage as a determinant of hypertension in people living with HIV.

	Weighted hypertension prevalence, % (95% Confidence Interval)	Odds ratio adjusted for only age and sex (95% confidence interval)	P-value	Fully adjusted odds ratio (95% confidence interval)	P-value
**Current ART usage[1]**			0.383		0.321
Not using ART	13.2% (9.5%-16.8%)	1		1	
Using ART	14.6% (11.8%-17.5%)	0.83 (0.54-1.27)		0.80 (0.51-1.25)	
**Sex**			<0.001		<0.001
Male	6.0% (3.3%-8.8%)	1		1	
Female	20.3% (17.1%-23.6%)	5.11 (2.95-8.84)		5.74 (3.24-10.19)	
**Age, years**			<0.001		<0.001
15-24	7.9% (2.6%-13.1%)	1		1	
25-34	12.3% (7.8%-16.8%)	1.69 (0.72-3.94)		2.07 (0.87-4.90)	
35-44	13.1% (9.2%-17.0%)	1.87 (0.83-4.23)		2.64 (1.14-6.11)	
45-54	12.0% (8.2%-15.8%)	2.01 (0.88-4.58)		2.87 (1.21-6.82)	
55-64	24.7% (14.7%-34.6%)	6.19 (2.43-15.75)		9.75 (3.48-27.31)	
65 and over	27.8% (13.8%-41.9%)	7.53 (2.71-20.94)		12.86 (4.22-39.14)	
**Site**			<0.001		<0.001
Bonda Mission (subsistence farming area)	13.3% (7.8%-18.9%)	1		1	
Eastern Highlands (tea estate)	6.1% (2.0%-10.2%)	0.64 (0.26-1.57)		0.63 (0.26-1.56)	
Selbourne (forestry estate)	8.3% (3.9%-12.6%)	0.82 (0.37-1.82)		0.81 (0.37-1.80)	
Nyazura (town)	19.6% (13.5%-25.8%)	2.13 (1.10-4.14)		2.15 (1.10-4.18)	
Nyanga (town)	6.1% (0.1%-12.0%)	0.44 (0.13-1.45)		0.43 (0.13-1.44)	
Watsomba (roadside settlement)	10.6% (5.8%-15.3%)	0.84 (0.42-1.67)		0.81 (0.40-1.62)	
Sakubva (urban)	30.9% (20.9%-40.8%)	4.58 (2.21-9.51)		4.49 (2.16-9.32)	
Hobhouse (urban)	25.8% (14.4%-37.3%)	3.29 (1.43-7.54)		3.26 (1.42-7.47)	
**Employment**			0.352		-
Unemployed	16.0% (12.7%-19.3%)	1		-	
Employed or in education	12.3% (9.3%-15.4%)	1.20 (0.81-1.78)		-	
**Relationship status**			0.671		-
Married or in a long-term relationship	12.4% (9.6%-15.2%)	1		-	
Never been in a long-term relationship	11.1% (4.2%-18.0%)	0.81 (0.33-2.03)		-	
Separated or divorced	17.2% (10.9%-23.4%)	1.17 (0.70-1.95)		-	
Widowed	19.8% (13.6%-25.9%)	0.81 (0.50-1.31)		-	

**Education**			0.562		-
None or primary	17.6% (12.8%-22.4%)	1		-	
Secondary or higher	12.6% (10.0%-15.1%)	1.15 (0.72-1.81)		-	
**Asset-based wealth**			0.003		-
1 (poorest)	8.8% (5.1%-12.5%)	1		-	
2	12.0% (7.5%-16.5%)	1.67 (0.88-3.16)		-	
3	12.6% (8.0%-17.2%)	1.64 (0.86-3.15)		-	
4	18.0% (12.7%-23.3%)	2.75 (1.49-5.09)		-	
5 (richest)	22.9% (15.3%-30.5%)	3.15 (1.64-6.07)		-	

[1]ART, antiretroviral therapy.

Supplementary table 4. Adherent current antiretroviral therapy usage as a determinant of hypertension in people living with HIV.

	Weighted hypertension prevalence, % (95% Confidence Interval)	Odds ratio adjusted for only age and sex (95% confidence interval)	P-value	Fully adjusted odds ratio (95% confidence interval)	P-value
**Adherent current ART usage[1]**			0.125		0.107
Not currently using ART or not adherent to ART	14.0% (10.5%-17.5%)	1		1	
Currently using and adherent to ART	14.2% (11.3%-17.1%)	0.73 (0.49-1.09)		0.70 (0.46-1.08)	
**Sex**			<0.001		<0.001
Male	6.0% (3.3%-8.8%)	1		1	
Female	20.3% (17.1%-23.6%)	5.11 (2.95-8.84)		5.86 (3.32-10.36)	
**Age, years**			<0.001		<0.001
15-24	7.9% (2.6%-13.1%)	1		1	
25-34	12.3% (7.8%-16.8%)	1.69 (0.72-3.94)		2.12 (0.89-5.07)	
35-44	13.1% (9.2%-17.0%)	1.87 (0.83-4.23)		2.74 (1.18-6.34)	
45-54	12.0% (8.2%-15.8%)	2.01 (0.88-4.58)		3.00 (1.27-7.10)	
55-64	24.7% (14.7%-34.6%)	6.19 (2.43-15.75)		10.30 (3.69-28.74)	
65 and over	27.8% (13.8%-41.9%)	7.53 (2.71-20.94)		13.77 (4.52-41.96)	
**Site**			<0.001		<0.001
Bonda Mission (subsistence farming area)	13.3% (7.8%-18.9%)	1		1	
Eastern Highlands (tea estate)	6.1% (2.0%-10.2%)	0.64 (0.26-1.57)		0.63 (0.26-1.56)	
Selbourne (forestry estate)	8.3% (3.9%-12.6%)	0.82 (0.37-1.82)		0.80 (0.36-1.78)	
Nyazura (town)	19.6% (13.5%-25.8%)	2.13 (1.10-4.14)		2.16 (1.11-4.20)	
Nyanga (town)	6.1% (0.1%-12.0%)	0.44 (0.13-1.45)		0.42 (0.13-1.39)	
Watsomba (roadside settlement)	10.6% (5.8%-15.3%)	0.84 (0.42-1.67)		0.79 (0.40-1.58)	
Sakubva (urban)	30.9% (20.9%-40.8%)	4.58 (2.21-9.51)		4.37 (2.10-9.11)	
Hobhouse (urban)	25.8% (14.4%-37.3%)	3.29 (1.43-7.54)		3.22 (1.40-7.42)	
**Employment**			0.352		-
Unemployed	16.0% (12.7%-19.3%)	1		-	
Employed or in education	12.3% (9.3%-15.4%)	1.20 (0.81-1.78)		-	
**Relationship status**			0.671		-
Married or in a long-term relationship	12.4% (9.6%-15.2%)	1		-	

Never been in a long-term relationship	11.1% (4.2%-18.0%)	0.81 (0.33-2.03)		-	
Separated or divorced	17.2% (10.9%-23.4%)	1.17 (0.70-1.95)		-	
Widowed	19.8% (13.6%-25.9%)	0.81 (0.50-1.31)		-	
**Education**			0.562		-
None or primary	17.6% (12.8%-22.4%)	1		-	
Secondary or higher	12.6% (10.0%-15.1%)	1.15 (0.72-1.81)		-	
**Asset-based wealth**			0.003		-
1 (poorest)	8.8% (5.1%-12.5%)	1		-	
2	12.0% (7.5%-16.5%)	1.67 (0.88-3.16)		-	
3	12.6% (8.0%-17.2%)	1.64 (0.86-3.15)		-	
4	18.0% (12.7%-23.3%)	2.75 (1.49-5.09)		-	
5 (richest)	22.9% (15.3%-30.5%)	3.15 (1.64-6.07)		-	

[1]ART, antiretroviral therapy.

Manicaland Study Questionnaires.

The household and individual questionnaires are copied below.

MANICALAND HIV/STD PREVENTION STUDY: ROUND 7 version: 11/03/18

HOUSEHOLD QUESTIONNAIRE ***FORM A***

Individual questionnaire eligibility:

**IVQs: young adults**	Y N
**IVQs: older adults**	Y N

Questionnaire processing dates:

Corrections generated
Corrections completed
Batch

HOUSEHOLD IDENTIFICATION

**Q001**	**Census district:**	______ **Ward:** ☐
**Q002**	**Village:**	______
**Q003**	**Name of household head (R6):**	______
**Q004**	**Name of household head (R7):**	______
**Q005**	**Category of household (R6)*:**	☐
**Q006**	**Study site reference & cluster.**	☐☐
**Q007**	**Household number:**	☐
**Q008**	**Growth point or compound:**	**Yes** 1 ☐<br>**No** 2 ☐
**Q009**	**Distance from nearest (tarred) roadside business centre**	☐
**Q010**	**GPS coordinates**	**Longitude** ☐<br>**Latitude** ☐<br>**Altitude** ☐

**INTERVIEWER VISIT**		**1**	**2**	**3**
**Q011**	**Date:**	___	___	___
**Q012**	**Time:**	___	___	___
**Q013**	**Interviewer:**	___	___	___
**Q014**	**Result**:**	☐	☐	☐
**Q015**	***How many deaths have there been in the household in the last 12 months?***			☐
**Q016**	***How many of the people who died were aged 15-59 years at their last birthday?***			☐

Do NOT correct after completing interview.

CHECKED BY SUPERVISOR

**Q017**	**Signature:**	______
**Q018**	**Date:**	______

*HOUSEHOLD CATEGORY

Follow-up household from R6	1
Household refused at R6	4
Household missed at R6	5
New household in the area since R6	8
New study site	6
Other (specify) ______________________	98

**RESULT CODES

Completed	1
No one at home	2
Household away for duration of survey	3
Postponed	4
Refused	5
Dwelling vacant or address not a dwelling	6
Dwelling destroyed	7
Dwelling not found	8
Other (specify) ______________________	9
Household relocated - within study areas	10
Household relocated - outside study areas	11
Household dispersed (split up)	12

HOUSEHOLD MEMBERS FORM: UPDATE ON MEMBERS RECORDED IN 2012-2013 SURVEY VISIT *Q. No:*

"Now I would like to get some information about the people who were staying in the household when we came to see you last time"...

LINE NO	USUAL RESIDENTS AND REGULAR VISITORS (ROUND 6)	REL'SHIP TO H.O.H. (R6)	(R7)	SPOUSE IN SAME HOUSEHOLD		SEX	AGE ('12-'13)	PARENTS SURVIVAL FOR CHILDREN UNDER 18 YEARS OLD: FALIVE	FBC	YFDIED	MALIVE	MBC	YMDIED	EDUCATION		HOUSEHOLD MEMBER'S SURVIVAL STATUS NOW			RESIDENCE				ELIGIBILITY FOR INTERVIEW: IVDONE ('12-'13)	CAT'GY (R6)	SELECT (R6)
Q019	Q020	Q021	Q022	Q023		Q024	Q025	Q026		Q027	Q028		Q029	Q030	Q031	Q032			Q034	Q035	Q036	Q037		Q038	Q039
	Circle line no. of respondent.			*Is (NAME) married to another member of this household?* If yes: record spouse's line no.				*Is (NAME)'s NATURAL BIOLOGICAL father still alive?* Tick "BC" if checked identity - birth certificate.		Year died	*Is (NAME)'s NATURAL BIOLOGICAL mother still alive?* Tick "BC" if checked identity - birth certificate.		Year died	*State highest level of education completed.***		*Is (NAME) still alive?* If died, record month and year of death. Go to Q035 if more than a year ago.			*Was (NAME) staying here in the same month last year?*	*Does (NAME) still stay in this household on a regular basis? (at death)*	*How many nights is it since (NAME) last slept in / left (see Q035) this household?*	*How many nights has (NAME) slept here in the last month?*	Codes for CATEGORY: …		
				Y N	#	M F	Years	Y N DK	BC	Year	Y N DK	BC	Year	Level	Years	Y N DK	Mth	Yr	Y N	Y N	# or >1yr	#	MUT6NO	#	Code
1	Serina Nyauzanza	1		1 2		2	46	1 2 8			1 2 8					1 2 8			1 2	1 2				1	7
2	Angelina Nyazuna	3		1 2		2	27	1 2 8			1 2 8					1 2 8			1 2	1 2			1	2	1
3	Edmore Nyazuna	5		1 2		1	8	1 2 8			1 2 8					1 2 8			1 2	1 2				3	
4	Telma Nyazuna	5		1 2		2	4	1 2 8			1 2 8					1 2 8			1 2	1 2				4	

HOUSEHOLD MEMBERS FORM *Q. No:* [ ]

"Now I would like to get some information about the people who were NOT members of the household when we came 5 years ago but who are staying here now or who have done so since we came last time " ...

LINE NO	USUAL RESIDENTS AND REGULAR VISITORS‡	RELATED TO H.O.H. (R7)	SPOUSE IN SAME HOUSEHOLD		SEX	AGE	PARENTS SURVIVAL RELATIONSHIP TO CARER FOR CHILDREN UNDER 18 YEARS OLD						EDUCATION		HOUSEHOLD MEMBER'S SURVIVAL STATUS		RESIDENCE					ELIGIBILITY FOR INTERVIEW CAT'Y	SELECT
Q019	Q020	Q022	Q023		Q024	Q025	Q026		Q027	Q028		Q029	Q030	Q031	Q032		Q033	Q034	Q035	Q036	Q037	Q038	Q039
	Please give me the names of the new persons who have been staying (physically) in this househo on a regular basis (starting with the head of household) **Circle line no. of respondent.**	***What is the relationship of (NAME) to the head of household?***	***Is (NAME) married to another member of this household?*** **If yes: record spouse's line no.**		***Is (NAME) male or female?***	***How old is (NAME) ?***	***Is (NAME)'s NATURAL BIOLOGICAL father still alive?*** **Tick "BC" if checked identity - birth certificate.**		**Year died.**	***Is (NAME)'s NATURAL BIOLOGICAL mother still alive?*** **Tick "BC" if checked identity - birth certificate.**		**Year died.**	***State highest level of education completed.*****		***Is (NAME) still alive?***		***When did (NAME) start staying in this house-hold?***	***Was (NAME) staying here in the same month last year?***	***Does (NAME) STILL stay in this household on a regular basis? (/at death)***	***How many nights is it since (NAME) last slept in this household?***	***How many nights has (NAME) slept here in the last month?***	Codes …	**Circle line no if person selected for inter-view*****
		See footnote*	Y N	#	M F	Years	Y N DK	BC	Year	Y N DK	BC	Year	Level	Years	Y N DK	Mth Yr	Mth Yr	Y N	Y N	#	#		
70 1			1 2		1 2		1 2 8			1 2 8					1 2 8			1 2	1 2				70 1
70 2			1 2		1 2		1 2 8			1 2 8					1 2 8			1 2	1 2				70 2
70 3			1 2		1 2		1 2 8			1 2 8					1 2 8			1 2	1 2				70 3
70 4			1 2		1 2		1 2 8			1 2 8					1 2 8			1 2	1 2				70 4
70 5			1 2		1 2		1 2 8			1 2 8					1 2 8			1 2	1 2				70 5
70 6			1 2		1 2		1 2 8			1 2 8					1 2 8			1 2	1 2				70 6
70 7			1 2		1 2		1 2 8			1 2 8					1 2 8			1 2	1 2				70 7
70 8			1 2		1 2		1 2 8			1 2 8					1 2 8			1 2	1 2				70 8
70 9			1 2		1 2		1 2 8			1 2 8					1 2 8			1 2	1 2				70 9
71 0			1 2		1 2		1 2 8			1 2 8					1 2 8			1 2	1 2				71 0

Tick here, if a further continuation sheet is used: [ ]

Enter total numbers of individuals selected for interview:
AGYW (15-24 yrs) [ ]
Young men (15-29 yrs) [ ]
Older adults (25/30+ yrs) [ ]

"Just to make sure that I have a complete listing" ...

Q040 ***Are there any other persons such as small children or infants that we have not yet listed?*** **Number:** [ ] **Add each in table above.** [ ]

Q041 ***In addition, are there any other people who may not be members of your family, such as lodgers, friends or domestic servants who live here now or have done so in the last three years?*** **Number:** [ ] **Add each in table above.** [ ]

Q042 ***Are there any other family members or other people who usually stay elsewhere, but who sleep here from time to time and stayed overnight within the last month?*** **Number:** [ ] **Add each in table above.** [ ]

Q043 ***Are there any other people who stayed here for a while but have now passed away?*** **Number:** [ ] **Add each in table above.** [ ]

* CODES FOR Q021: RELATIONSHIP TO HEAD OF HOUSEHOLD

01	Head	07	Parent-in-law	13	Paternal uncle
02	Wife or husband	08	Brother or sister (natural)	14/15	Maternal grandfather/mother
03	Son or daughter (natural)	09	Stepfather/mother (father's co-wife)	16/17	Paternal grandfather/mother
04	Son or daughter-in-law	10	Maternal aunt	18	Other relative/cousin
05	Grandchild	11	Paternal aunt	19	Adopted/foster child
06	Father / mother (natural)	12	Maternal uncle	20	Not related

** CODES FOR Q030 & Q031: LEVEL & YEARS OF EDUCATION

Level:		Years: years completed:	
0	None	0	Less than one year completed
1	Primary	98	Don't know
2	Secondary		

***ELIGIBLE PERSONS (Q038/Q039)

Usual residents or regular visitors who stayed in the household at least 4 nights last month
11 AGYW (15-24 yrs)
12 Young men (15-29 yrs)
13 Older adults (25/30+ yrs) - 'Yes' households only

‡ Regular visitors are people who, on average, stay in the household at least once a month

FORM A

HOUSEHOLD SOCIO-ECONOMIC STATUS ***Q. No:*** ____

REF.	QUESTIONS & FILTERS	CODING CATEGORIES		SKIP TO
Q044	*What is the main source of drinking water for members of your household?* ______	Piped into residence	1	
		Private tap in yard or plot	2	
		Communal tap	3	
		Own well or borehole	4	
		Other well or borehole	5	
		Protected spring	6	
		Other (specify)	10	
Q045	*What kind of toilet facility does your household have?* ______	Flush toilet	1	
		Blair toilet	2	
		Pit latrine	3	
		Other (specify)	4	
		No facilities	5	- Q047
Q046	*Is this toilet facility used by members of your household alone, shared with neighbours, or is it communal?*	Household alone	1	
		Shared with neighbours	2	
		Communal	3	
Q047	*Does your household have:*		Y N	
	Electricity?	Electricity	1 2	
	A refridgerator?	Refridgerator	1 2	
	A radio?	Radio	1 2	
	A television?	Television	1 2	
Q048	Record house type.	Pole and dagga structure	1	
		Brick house - thatched roof	2	
		Brick house - tiled/sheeting roof	3	
		Cabin/other	8	
Q049	Observe and record type of floor of the main dwelling.	Natural floor (earth/sand/dung)	1	
		Rudimentary (planks/palm/bamboo)	2	
		Finished (wood/cement/carpet...)	3	
Q050	*Does any member of your household own? …*		Y N	
	A bicycle?	Bicycle	1 2	
	A motorcycle?	Motorcycle	1 2	
	A car?	Car	1 2	
	A tractor?	Tractor	1 2	
Q051	*How many cattle does your household own?*	Number of cattle		
Q052	*Now I would like to ask you some questions about food: During the last 12 MONTHS, was there a time when:*		Y N DK	
	1) You or others in your household worried about not having enough food to eat because of a lack of money or other resources?		1 2 98	
	2) Still thinking about the last 12 MONTHS, was there a time when you or others in your household were unable to eat healthy and nutritious food because of a lack of money or other resources?		1 2 98	
	3) Was there a time when you or others in your household ate only a few kinds of food because of lack of money or other resources?		1 2 98	
	4) Was there a time when you or others in your household had to skip a meal because there was not enough money or other resources to get food?		1 2 98	
	5) Still thinking about the last 12 MONTHS, was there a time when you or others in your household ate less than you thought you should because of a lack of money or other resources?		1 2 98	
	6) Was there a time when your household ran out of food because of a lack of money or other resources?		1 2 98	
	7) Was there a time when you or others in your household were hungry but did not eat because there was a lack of money or other resources for food?		1 2 98	
	8) Was there a time when you or other in your household went without eating for a whole day because of a lack of money or other resources?		1 2 98	

HOUSEHOLD MEMBERS FORM: INDIVIDUAL INTERVIEW PROCESSING

Q. No:

Study site:

HHID:

Village:

Growth point:

Name of household head (R7):

Cell #:

LINE NO	HOUSEHOLD MEMBERS SELECTED FOR INDIVIDUAL INTERVIEW	CONTACT DETAILS	APPOINTMENTS									IVQ REF.
Q053	Q054	Q055	Q056			Q057			Q058			Q059
From Q019 (ii/iii)	Name	Cell #	1st App't			2nd App't			3rd App't			Note ref.
No.	*Adults (15 yrs +)*		Time	Day/Mth	Res(1)	Time	Day/Mth	Res(2)	Time	Day/Mth	Res(3)	MUT7NO

(1) RESULT CODES

1. Completed
2. Partially completed - questionnaire only
3. Partially completed - BE only
4. Partially completed - questionnaire & BE only (HTC outstanding)
5. Missed appointment - rescheduled
6. Missed appointment - requires follow-up
7. Attended but refused
8. Other (specify)

* Result fields to be completed by supervisors

Individual questionnaire - baseline survey

MANICALAND HIV/STD PREVENTION STUDY: ROUND 7

INDIVIDUAL QUESTIONNAIRE ***FORM B***

MUT7number:

Questionnaire processing dates:

Consent form
Corrections completed

QUESTIONNAIRE IDENTIFICATION

**Q001**	**Census district:** ______	**Ward:**	☐
**Q002**	**Village:** ______	**Cluster:**	☐
**Q003**	**Name of head of household:** ______		
**Q004**	**Category of respondent:**	R6 MUTNO	☐
**Q005**	**Study site reference:** Date left R6 area	R6 site	☐
**Q006**	**Household number:**		☐
**Q007**	**Line number on household questionnaire:**		☐

INTERVIEWER VISIT

		1	2	3
**Q008**	**Date:**	___	___	___
**Q009**	**Time:**	___	___	___
**Q010**	**Interviewer:**	___	___	___
**Q011**	**Result**:**	☐	☐	☐

Q012 **Interview timing:**
Before BE measurement **1**
After BE measurement **2**

CHECKED BY SUPERVISOR

Q013 **Signature:** ______

Q014 **Date:** ______

*RESPONDENT CATEGORY	
Follow-up respondent (from R6)	1
New respondent: previously under age	2
New respondent: in-migrant (since R6)	5
New respondent: selected but unavailable R6	6
New respondent: selected but refused R6	7
New respondent: household missed R6	9
New respondent: new study site in R7	10
Other (specify)______	8

**RESULT CODES	
Completed	1
Not at home	2
Refused	3
Partially completed	4
Sick/hospital	5
Out-migrated	100
Other (specify)______	8

INDIVIDUAL QUESTIONNAIRE: **INVITATION TO JOIN THE STUDY**

Explain the purpose of the study - including potential benefits nationally and to the community.
Explain what is involved in participating in the study - show/read invitation letter and consent form.
Ask about and discuss any concerns the respondent might have.
Seek consent to participate in the study - request signature on consent form.

REF.	QUESTIONS & FILTERS	CODING CATEGORIES		SKIP TO
Q101	Indicate whether the respondent wishes to participate in the study.	Yes<br>No	1<br>2	- Q103
Q102	Indicate the main reason why he/she does not wish to participate.<br>* Ask for another appointment if reason is insufficient time.	Insufficient time*<br>DBS samples<br>Information too personal<br>Other (specify)	1<br>3<br>4<br>8	
Q103	Record details of others present at this point.	Children under 10<br>Husband/wife<br>Other males<br>Other females	Yes / No<br>1 2<br>1 2<br>1 2<br>1 2	

INDIVIDUAL QUESTIONNAIRE: **BACKGROUND CHARACTERISTICS** *Q. No:*

REF.	QUESTIONS & FILTERS	CODING CATEGORIES		SKIP TO
Q201	Record the current time (24 hour clock).	Hour / Minutes	hr / mins	
Q202	Record sex of respondent.	Male<br>Female	1<br>2	
Q203	*In what month and year were you born?*		mnth / yr	
Q204	*How old were you at your last birthday?*<br>Check consistency with Q203.	Age in COMPLETED years	yrs	
Q205	*Are you currently enrolled in school full-time?*	Yes<br>No	1<br>2	- Q208
Q206	*How old were you when you left school?*	Age in completed years<br>Never been to school	yrs<br>99	 - Q209
Q207	*What was your reason for leaving school?*<br>If parents decided, probe for the underlying reason.	Insufficient funds<br>Found a job<br>To go to college or university<br>Inadequate exam passes<br>Needed to help at home<br>Marriage<br>Pregnancy/childbirth - voluntarily<br>Pregnancy - expelled<br>Caught having sex - expelled<br>Expelled - other reasons<br>Finished secondary school<br>Other (specify)	1<br>2<br>3<br>4<br>5<br>6<br>7<br>8<br>9<br>10<br>11<br>18	
Q208	*What is the highest grade of school you have completed?*<br>For "years", enter number of years (excl. repeats) at highest level reached.	None<br>Primary<br>Secondary<br>Higher	Level / Years<br>0<br>1 / 1-7<br>2 / 1-6<br>3 / 1-6	 <br><br>- Q210<br>- Q210
Q209	*Can you read a letter or newspaper in any language?*	Yes<br>No	1<br>2	
Q210	*How long have you been living in this homestead?*	Years<br>Since birth<br>Visitor	yrs<br>995<br>996	

INDIVIDUAL QUESTIONNAIRE:		**BACKGROUND CHARACTERISTICS**			*Q. No:*
**REF.**	**QUESTIONS & FILTERS**	**CODING CATEGORIES**			**SKIP TO**
**Q211**	*How long have you been living in (NAME OF VILLAGE)?*	**Years**		yrs	
		**Since birth**	995		- **Q213**
		**Visitor**	996		
**Q212**	*What type of place was your previous place of residence?* Record place of current home if the respondent is a visitor. "Roadside" here means a tarred road. Record the name of the place below.	**Large town or city**	1		
		**Small town**	2		
		**Growth point**	3		
		**Commercial estate/mine**	4		
		**Roadside business centre**	5		
		**Rural business centre**	6		
		**Communal/resettlement area**	7		
**Q213**	*Which church denomination do you belong to?*	**Traditional**	1		
		**Pentecostal**	2		
		**Methodist**	4		
		**Anglican**	5		
		**Roman Catholic**	6		
		**ZAOGA**	7		
		**Apostolic Faith Mission**	8		
		**Marange Apostolic**	9		
		**Mazowe Apostolic**	10		
		**Zviratidzo Apostolic**	12		
		**Other Apostolic (specify)**	13		
		**Zionist**	15		
		**Mughodi**	20		
		**Other (specify)**	17		
		**None**	97		
**Q214**	*In which sector of employment do you work?*	**Estates: tea, coffee, forestry etc**	1		
		**Manufacturing or building trade**	2		
		**Police or army**	3		
		**Teacher: primary school**	4		
		**Teacher: secondary school**	5		
		**Nurse**	6		
		**Services or retail: shops**	7		
		**Informal: petty trading (veg etc)**	8		- **Q216**
		**Informal: subsistence agriculture**	9		- **Q216**
		**Student**	10		- **Q216**
		**Unemployed: excl. agriculture**	11		- **Q216**
		**Other (specify)**	12		
**Q215**	*What type of work do you do?*	**Professional or managerial**	1		
		**Self-employed: small business**	2		
		**Skilled labour**	3		
		**Manual/unskilled labour**	4		
**Q216**	*Over the last 12 months, how many times per month, on average, have you had a drink containing alcohol?*	**Number of times per month**			- **Q218 if '0'**
**Q217**	*How many drinks containing alcohol do you have on a typical sitting?*	**Number of drinks per sitting**			
**Q218**	*How many times have you visited a bar or beer-hall in the last month?*	**Number of times**			
**Q219**	*Do you smoke cigarettes?*	**Yes**	1		
		**No**	2		
**Q220**	*Do you take any of these types of drugs for pleasure?*		Yes	No	
		**Injecting drugs**	1	2	
		**Drugs you smoke**	1	2	
		**Prescription drugs**	1	2	
		**Other drugs you swallow**	1	2	
**Q221**	*Have you ever been married or in a long-term or cohabiting relationship?* Relationships of 12 months or more should be treated as "long-term".	**Yes**	1		
		**No**	2		- **Q235**
**Q222**	*How many such relationships have you experienced in your lifetime?*	Include current relationships.			
**Q223**	*How old were you when you first entered such a relationship?*	**Age (years)**		yrs	

INDIVIDUAL QUESTIONNAIRE: **BACKGROUND CHARACTERISTICS** ***Q. No:***

REF.	QUESTIONS & FILTERS	CODING CATEGORIES					SKIP TO
**Q224**	*Are you currently widowed, divorced or separated from your most recent spouse/partner?*	**Widowed**	1				- **Q235**
		**Divorced**	2				- **Q235**
		**Separated**	3				- **Q235**
		**Still in union**	4				
**Q225**	*How many spouses/long-term partners do you have at present?* For women, ask how many other wives her husband has.	**(Not zero!)** Ask questions Q230 to Q238 for first spouse, then the second, and so on ...					
**Q226**	*How old was your partner at his/her last birthday?*	**Age in completed years**	yrs	yrs	yrs	yrs	
		**Don't know**	98	98	98	98	
**Q227**	*Did you and your spouse have an HIV test before you agreed to get married?*	**Self only tested**	2	2	2	2	
		**Partner only tested**	3	3	3	3	
		**Neither tested**	4	4	4	4	- **Q229**
		**Not yet 'married'**	5	5	5	5	- **Q229**
		**Both tested seperately**	11	11	11	11	
		**Both tested together**	12	12	12	12	- **Q229**
**Q228**	*Did you tell each other your results?* Ask equivalent if only one tested.	**Yes**	1	1	1	1	
		**No**	2	2	2	2	
**Q229**	*Do you know whether this person has HIV infection now?* If yes, ask for partner's status.	**Infected**	1	1	1	1	
		**Uninfected**	2	2	2	2	- **Q231**
		**Prefers not to say**	96	96	96	96	- **Q231**
		**Don't know**	98	98	98	98	- **Q231**
**Q230**	*Is this person receiving ART (i.e. treatment to prevent AIDS)?*	**Yes**	1	1	1	1	
		**No**	2	2	2	2	
		**Prefers not to say**	96	96	96	96	
		**Don't know**	98	98	98	98	
**Q231**	*Has he/she had a sexually transmitted infection (other than HIV) in the last months?*	**Yes**	1	1	1	1	- **Q233 if respondent is male**
		**No**	2	2	2	2	
		**Don't know**	98	98	98	98	
**Q232**	*Has he been circumcised?* Show respondent pictures to establish whether fully or partially circumcised.	**Yes - full**	1	1	1	1	
		**Yes - partial**	2	2	2	2	
		**No**	3	3	3	3	
		**Don't know**	98	98	98	98	
**Q233**	*What is the highest grade of school your partner has completed?*	**None**	0	0	0	0	
		**Primary**	1	1	1	1	
		**Secondary**	2	2	2	2	
		**Higher**	3	3	3	3	
**Q234**	*In which sector of employment does he/she work?*	**Estates**	1	1	1	1	
		**Manuf'trg/building**	2	2	2	2	
		**Police/army**	3	3	3	3	
		**Teacher: primary**	4	4	4	4	
		**Teacher: secondary**	5	5	5	5	
		**Nurse**	6	6	6	6	
		**Services/retail: shops**	7	7	7	7	
		**Informal: trading**	8	8	8	8	- **Q235**
		**Informal: incl agric**	9	9	9	9	- **Q235**
		**Student**	10	10	10	10	- **Q235**
		**Unemployed: excl. agr**	11	11	11	11	- **Q235**
		**Other (specify)**	12	12	12	12	
**Q235**	*What type of work does he/she do?*	**Prof/manage't**	1	1	1	1	
		**Self-employed**	2	2	2	2	
		**Skilled labour**	3	3	3	3	
		**Manual/unskilled**	4	4	4	4	
**Q236**	*Which of the following groups exist in your home area and which are you a member of?* For those where he/she is a member: *Would you say that this group functions: (1) well; (2) OK; or (3) poorly?*			Exist	Member	Rating	
		**Church groups**					
		**Women's groups**					
		**Co-operative**					
		**Farmers group**					
		**Burial society**					
		**Savings club (RCS)**					
		**Youth group**					
		**Sports club**					
		**HIV/AIDS group**					
		**Political party**					
**Q237**	*What is the principal activity of the group you spend the most time with?*	**Code from Q235**					
		**None**	99				

INDIVIDUAL QUESTIONNAIRE: **BACKGROUND CHARACTERISTICS** *Q. No:*

REF.	QUESTIONS & FILTERS	CODING CATEGORIES	Yes	No	SKIP TO
**Q238**	*Do you play one or more of these roles in your community?*	**Chief, Headman or Kraal Head**	1	2	
		**DA or CEO**	1	2	
		**Political leader (MP, councillor …)**	1	2	
		**Church leader**	1	2	
		**Teacher**	1	2	
		**Traditional healer**	1	2	
		**Faith healer**	1	2	
		**Doctor or nurse**	1	2	
		**Village health worker**	1	2	
		**Formal sector manager/employer**	1	2	
	______________________	**Other (specify)**	1	2	
**Q239**	*Are you related to a young woman aged between 15-24 years in the following ways?*	**Parent**	1	2	
		**Sister or brother (same parents)**	1	2	
		**Aunt or uncle**	1	2	
		**Grandparent**	1	2	
		**Spouse or regular partner**	1	2	
**Q240**	*Are you related to a young man aged between 15-29 years in the following ways?*	**Parent**	1	2	
		**Sister or brother (same parents)**	1	2	
		**Aunt or uncle**	1	2	
		**Grandparent**	1	2	
		**Spouse or regular partner**	1	2	

PSYCHOLOGICAL HEALTH

"In the past 2 weeks, how often have you been bothered by any of the following problems? …"

		Not at all	Several days	> 1/2 days	Nearly every day
**Q301**	*Little interest or pleasure in doing things*	1	2	3	4
**Q302**	*Feeling down, depressed, or hopeless*	1	2	3	4
**Q303**	*Trouble falling asleep, staying asleep, or sleeping too much*	1	2	3	4
**Q304**	*Feeling tired or having little energy*	1	2	3	4
**Q305**	*Poor appetite or overeating*	1	2	3	4
**Q306**	*Feeling bad about yourself - or that you're a failure or have let yourself or your family down*	1	2	3	4
**Q307**	*Trouble concentrating on things such as reading a newspaper or watching TV*	1	2	3	4
**Q308**	*Moving or speaking so slowly that other people could have noticed. Or, the opposite - being fidgety or restless that you have been moving around a lot more than usual*	1	2	3	4
**Q309**	*Thoughts that you would be better off dead or of hurting yourself in some way*	1	2	3	4

		Not hard at all	Some-what hard	Very hard	Extreme-ly hard
**Q310**	*If you had any of these problems, how hard have these problems made it for you to do your work, take care of things at home, or get along with other people?*	1	2	3	4

INDIVIDUAL QUESTIONNAIRE: **SEXUAL RELATIONSHIPS** *Q. No:*

REF.	QUESTIONS & FILTERS	CODING CATEGORIES			SKIP TO
Q401	10 minutes of informal discussion first to build rapport, trust & stress absence of prejudice.<br>Explain the need to ask questions on the respondent's own experience of sexual relationships.<br>Stress the importance of providing accurate information.<br>Stress that strict confidentiality will be maintained - request privacy.				
Q402	*How old were you when you had sex for the first time?*<br>Explain what we mean by "having sex".	Age in years		yrs	- Q404
		Not yet had sex	99		
Q403	*What is the main reason you have not yet started to have sexual relations?*	Too young	1		- Q434
		Not met partner	2		- Q434
		Not yet married	3		- Q434
		Risk of pregnancy	4		- Q434
		Risk of HIV/AIDS	5		- Q434
		Other (specify)	8		- Q434
Q404	*How many days is it since you last had sex?*			days	
	Skip to Q406 if less than one month.	More than one year	99		
Q405	*What is the main reason you are currently abstaining from sexual relations?*<br>Options 1-4 could refer to the respondent or (if male) to his regular partner.	Current pregnancy	1		
		Recent birth	2		
		Terminal abstinence	3		
		Self or partner has an STD	4		
		Currently living apart	5		
		Risk of catching HIV/AIDS	6		
		Risk of passing on HIV/AIDS	7		
		Self or partner has HIV/AIDS	8		
		Religious reasons	9		
		Not currently married	10		
		No partner - although would like one	11		
		Ill-health	12		
		Under 1 year since spouse died	13		
		Don't like / not interested in having sex	14		
		Other (specify)	20		
		Don't know	98		
Q406	*Did you use condoms throughout the last time you had sex?*	Yes	1		
		No	2		
Q407	*Do you know whether the last person you had sex with has HIV infection?*<br>If infected, ask if on ART.	Infected - on ART	1		
		Infected - not on ART	2		
		Uninfected	3		
		Prefers not to say	4		
		Don't know	98		
Q408	*If you took a guess, how many partners other than yourself (and any co-wives) do you think your current spouse/partner has had in the last 12 months?*	Number of non-regular partners			
Q409	Indicate data collection method used.<br>For 'secret voting':<br>(i) explain the procedure and the confidentiality safeguards carefully.<br>(ii) establish whether able to use tablet.	Secret voting		1	
		Interview (if respondent unable to use tablet)		2	
Q410	*How many different REGULAR sexual partners have you had in your LIFETIME?*<br>*By REGULAR, I mean someone you have been having sex with for a year or more.*	Number of partners		1	

INDIVIDUAL QUESTIONNAIRE: **SEXUAL RELATIONSHIPS** *Q. No:*

REF.	QUESTIONS & FILTERS	CODING CATEGORIES		SKIP TO
Q411	*For how many years have you been using condoms EVERY TIME you have sex with a REGULAR partner?*	**Years**<br>**Ask respondent to write "0" if he/she does not use condoms with current regular partner(s).**	2	
Q412	*How many different NON-REGULAR sexual partners have you had in your LIFETIME?*	**Number of partners**	3	
Q413	*For how many years have you been using condoms EVERY TIME you have sex with a NON-REGULAR sexual partner?*	**Years**<br>**Ask respondent to write "0" if he/she doesn't use condoms with non-regular sexual partner(s) now.**<br>**Ask respondent to write "P" if he/she has never had a non-regular sexual partner.**	4	
Q414	*How many different sexual partners have you had in the LAST 12 MONTHS?*	**Number of partners**	5	
Q415	*How many of these partners were you having sex with for the FIRST TIME?*	**Number of new partners in last 12 months (STRESS)**	6	
Q416	*How many of the sexual partners that you had in the last 12 months were REGULAR partners?*	**Number of regular partners**	7	
Q417	*How many of the sexual partners that you had in the last 12 months were NON-REGULAR partners?*<br>**Ask respondent to check that this and the previous answer should add up to box #5.**	**Number of non-regular partners**	8	
Q418	*How many sexual relationships do you consider yourself to be involved in at the moment?*	**Number of current relationships**	9	
Q419	*How many different partners have you had sex with in the last month?*	**Number of partners in last month (total)**	10	

Q420 **Explain that you now wish to ask some questions about the last 3 persons the respondent had sex with. Note: NO time restriction. Stress that these may be people he/she had sex with only once.**

If secret voting is being used, ask the respondent to enter a "P" in each box if he/she has not had the minimum required number of partners in his/her lifetime.

REF.	QUESTIONS & FILTERS	CODING CATEGORIES	LAST	PREVIOUS ...		
Q421	*How many times have you had sexual intercourse with this partner in the last 2 weeks?*	**Number of times**				1
Q422	*On how many of these occasions did you and your partner use condoms THROUGHOUT?*	**Number of times**				2
Q423	*Did you use condoms throughout the LAST TIME you had sex with this partner?*	**Yes**<br>**No**				3
Q424	*What was the month and year when you had sexual intercourse with this person for the FIRST time?*	**Month first then year**	mnth / yr	mnth / yr	mnth / yr	4
Q425	*When was the month and year you LAST had sexual intercourse with this person?*	**Month first then year**	mnth / yr	mnth / yr	mnth / yr	5

INDIVIDUAL QUESTIONNAIRE: **SEXUAL RELATIONSHIPS** ***Q. No:***

REF.	QUESTIONS & FILTERS	CODING CATEGORIES		SKIP TO
**Q426**	***Are you still having sex with this person?***	**Yes**<br>**No**		6
**Q427**	***Where were you when you had sex with this person for the first time?***<br>**If secret voting is being used, show respondent the picture codes. (Code numbers as for Q212).**	**Code**		7
**Q428**	***How many years old is this person?***	**Age in years**	yrs yrs yrs	8
**Q429**	***Is / was this person someone you are / were married to or cohabiting with?***	**Yes**<br>**No**		9
**Q430**	***Is this person married to someone other than yourself?***	**Yes**<br>**No**<br>**Don't know**		10
**Q431**	***Have you given or received money, goods or services in exchange for sex with this person in the last month?***	**Yes**<br>**No**		11
**Q432**	**For men:**<br>***Did you have sex with one or more men in the last 12 months?***	**Yes**<br>**No**	1<br>2	12
**Q433**	**For men:**<br>***Have you EVER been involved in a non-marital relationship of any kind where you gave anything in exchange for sex?***	**Yes**<br>**No**	1<br>2	- **Q435**<br>13
**Q434**	**For women:**<br>***Have you EVER been involved in a non-marital relationship of any kind where you received anything in exchange for sex?***	**Yes**<br>**No**	1<br>2	14
**Q435**	**Record details of others present at this point.**	**Children under 10**<br>**Husband/wife**<br>**Other males**<br>**Other females**	Y(1) N(2)<br>Y(1) N(2)<br>Y(1) N(2)<br>Y(1) N(2)	

INDIVIDUAL QUESTIONNAIRE: **HIV PREVENTION METHODS** *Q. No:*

REF.	QUESTIONS & FILTERS	CODING CATEGORIES	SKIP TO
Q501	*"Now I would like to ask you some questions about the methods of HIV prevention that you know about ..."*		
Q502	*What methods of HIV prevention have you heard about?*<br><br>Ask first without probing and then probe for factors that are not mentioned spontaneously.	Columns: Spont / Probed / Where / Who<br>VMMC<br>PrEP<br>Male condoms<br>Female condoms<br>Spiritual protection<br>Faithfulness to one partner<br>Abstinence<br>HIV testing & counselling<br>Treatment as prevention<br>Other (specify)	
Q503	For each method of HIV prevention mentioned (after probing), except TasP, ask WHERE (venue or activity) they obtained most of their information about this method. Enter in the 3rd column in Q502 the code from the list given here that matches the response.	HIV testing service (integrated) 1<br>HIV testing service (stand-alone) 2<br>Health centre (staff and/or posters, leaflets etc.) 3<br>School (lessons, health visitors, clubs) 4<br>Home (visits from health or community worker) 5<br>Workplace 6<br>Church service or meeting 7<br>Shop (e.g. pharmacy) 8<br>Social media 9<br>Internet (other) 10<br>TV, radio or newspaper 11<br>Community group meetings (e.g. womens group, RCS …) 12<br>Other community meetings 13<br>Word of mouth 14<br>Other (specify) 15	
Q504	For each method of HIV prevention mentioned (after probing), except TasP, WHO was the organisation or person that they got most of their information about this method from).<br>Enter in the 4th column in Q502 the code from the list given here that matches the response.	Ministry of Health staff 1<br>Health centre committee member(s) 2<br>Community health worker 3<br>Community leaders (Chief, counsellor) 4<br>Church leader or members 5<br>Traditional healer 6<br>PSI 7<br>FACT 8<br>Other NGO (specify) 9<br>Other (specify) 12	
Q505	*What were the main APPROACHES used by these organisations to encourage people like you to use each of these methods of HIV prevention?*<br>1. Info'n campaigns - e.g. mass media, posters, leaflets<br>2. Practical skills training - e.g. how to use the method<br>3. Social skills training - e.g. negotiating skills<br>4. Counselling<br>5. Mass distribution - e.g. mobile or outreach services<br>6. Text message reminders<br>7. Financial or material incentives<br>Tick each approach that applies for each method of HIV prevention mentioned in Q502. Enter '99' if not applicable. (e.g. because the HIV prevention method is not known).	Columns: 1 / 2 / 3 / 4 / 5 …<br>VMMC<br>PrEP<br>Male condoms<br>Female condoms<br>Faithfulness to one partner<br>Abstinence<br>HIV testing & counselling	
Q506*	*Which methods of HIV prevention have you used in your lifetime?*<br><br>Enter '1' is 'Yes'; '2' if No'.<br><br>Probe to find out if more than one method has been used.	Columns: Ever / Now / Provider<br>VMMC<br>PrEP<br>Male condoms<br>Female condoms<br>Faithfulness to one partner<br>Abstinence<br>TasP<br>Other (specify)	
Q507**	*Which of the methods you know are you using now?*<br>Probe to find out if more than one method is being used.	Enter response in 2nd column in Q506.	
Q508	For methods used, ask for and record the current or most recent provider. Use codes from Q504.	Enter response in 3rd column in Q506.	
	Introduction explaining / defining VMMC & traditional circumcision.<br>For women, go to Q533.		

INDIVIDUAL QUESTIONNAIRE: **HIV PREVENTION METHODS** *Q. No:*

REF.	QUESTIONS & FILTERS	CODING CATEGORIES		SKIP TO
Q509**	*Have you ever had medical, traditional or religious male circumcision?*	Medical	1	- Q511
		Traditional or Religious (non-medical)	2	
		Both	3	- Q511
		None	8	
Q510	*Have you ever been offered VMMC?*	Yes	1	- Q519
		No	2	- Q519
	If not been offered, ask if heard of VMMC before.	Not heard of VMMC before today	99	- Q519
Q511	*Was it full or partial circumcision?*	Full	1	
	Show pictures to help distinguish.	Partial	2	- Q519
Q512	*Was the medical circumcision a surgical or non-surgical procedure?*	Surgical	1	
		Non-surgical	2	
	e.g. PREPEX is a non-surgical form of VMMC.	Don't know	98	
Q514	*How old were you when you received VMMC?*		yrs	- Q531 if <10 yrs
Q515	*Which of these organisations provided you with the VMMC service?*		Yes / No	
		Ministry of Health	1 / 2	
		PSI	1 / 2	
		ITEC	1 / 2	
		Other (specify)	1 / 2	
Q516	*How many visits to this place did you have to make for this procedure?*	Number of visits		
	i.e. from the first appointment to the final check-up. Record date of first appointment.	Date of first appointment	mth / yr	
Q517	*What costs did you incur when you had the VMMC done?*	Medical fees	$	
		Transport costs	$	
		Accommodation costs	$	
		Foregone income	$	
		Other costs (specify)	$	
Q518	*After you received VMMC, did you:*		More / Same / Less	
	(1) Increase, decrease or not change your number of sexual partners?		1 / 2 / 3	
	(2) Use condoms more, less or the same as before?		1 / 2 / 3	
	(3) Use condoms consistently with casual partners?	Yes	1	- Q529
		No	2	- Q529
		NA - no casual partners	99	- Q529
Q519	*Please tell me whether you strongly disagree, disagree, neither agree nor disagree, agree or strongly agree with the following statements …*		SD / D / N / A / SA	
	(1) I am confident I can get VMMC if I wanted to		1 / 2 / 3 / 4 / 5	
	(2) I am confident that I can get VMMC even if my partner disapproves of this		1 / 2 / 3 / 4 / 5	
	(3) I am confident I can get VMMC even if my friends disapprove of this		1 / 2 / 3 / 4 / 5	
	(4) I am confident I can get VMMC even if my parents and family elders disapprove		1 / 2 / 3 / 4 / 5	
Q520	*Are you able to discuss getting VMMC with your partner?*	Yes - already done so	1	
		Yes - not done already but can do this	2	
		No	3	
		No regular partner	99	- Q522
Q521	*Would your partner disapprove if you had VMMC?*	Yes	1	
		No	2	
		Don't know	3	
Q522**	*If you wanted to get VMMC yourself, do you know a place where someone like you can easily have it done?*	Yes	1	
		No	2	
Q523*	*If you wanted to get VMMC yourself, how easy would it be for you to access the service?*	Very easy	1	
		Easy	2	
		Neither easy nor difficult	3	
	Ask for an answer on a scale of 5 - from 1 (very easy) to 5 (very difficult).	Difficult	4	
		Very difficult	5	
		Don't know	98	

INDIVIDUAL QUESTIONNAIRE: **HIV PREVENTION METHODS** *Q. No:*

REF.	QUESTIONS & FILTERS	CODING CATEGORIES			SKIP TO
**Q524**	***What factors make it impractical or unsuitable for someone like you to access VMMC services?***		Spont	Probed	
	Ask first without probing and then probe for factors that are not mentioned spontaneously.	**High costs (including loss of income)**			
		**Inability to work during/after procedure**			
		**Healthcare providers are female**			
		**Lack of privacy / confidentiality**			
		**Limited opening hours**			
		**Distance / travel difficulties**			
		**Respondent is HIV+ (not suitable for PLHIV)**			**- Q532**
		**Other (specify)**			
**Q525****	***Do you want to get VMMC if the service was freely accessible to you?***	**Yes**	1		
		**No**	2		
		**Not sure**	8		
**Q526***	***How definitely do you want to get VMMC if the service was freely accessible to you?***	**Definitely**	1		
		**Probably**	2		
	Ask for an answer on a scale of 5 - from 1 (definitely) to 5 (definitely not).	**No opinion**	3		
		**Probably not**	4		
		**Definitely not**	5		
**Q527**	***Do you plan to get VMMC?***	**Yes**	1		**- Q529**
		**No**	2		**- Q529**
		**Don't know**	98		
**Q528**	***How soon do you plan to have VMMC?***	**Number of months / years**	mths	yrs	

REF.	QUESTIONS & FILTERS	CODING CATEGORIES	Pos	Neg	Neutral	Probed
**Q529**	***What factors were/are important in encouraging or discouraging you to have VMMC?***	**HIV/STI/HPV protection (for myself)**	1	2	8	
		**HIV/STI/HPV protection (for my partner(s))**	1	2	8	
	For each factor given, ask if this was a positive (encouraging) or negative (discouraging) factor.	**Method of HIV prevention I can control**	1	2	8	
		**Pain**	1	2	8	
	Ask first without probing and then probe for factors that are not mentioned spontaneously.	**Operation cannot be reversed**	1	2	8	
		**Make me feel responsible or ashamed**	1	2	8	
	Tick probed box to indicate probed responses.	**Make me feel more manly**	1	2	8	
		**Own sexual pleasure (better/worse)**	1	2	8	
		**Partner's sexual pleasure (better/worse)**	1	2	8	
		**Able to have erection for longer/shorter**	1	2	8	
		**More able to attract partners**	1	2	8	
		**Risk of getting an infection**	1	2	8	
		**Penis will be cleaner / look better**	1	2	8	
		**Using other prevention method (note method)**	1	2	8	
		**Abstaining or faithful to one trusted partner**	1	2	8	
		**Other (specify) ______________**	1	2	8	
**Q530**	***Which people's views were/are important in encouraging or discouraging you to have VMMC?***	**Religious leaders (religious beliefs)**	1	2	8	
		**Parents' or family elders' approval/views**	1	2	8	
		**Partner's views**	1	2	8	
	Instructions as for last question.	**Friends views**	1	2	8	
		**Community views**	1	2	8	
		**Other (specify) ______________**	1	2	8	

REF.	QUESTIONS & FILTERS	CODING CATEGORIES			SKIP TO
**Q531**	***How far away is it from where you live to the nearest place where VMMC services are provided?***	**Distance in kms**		kms	
		**Don't know**	98		
**Q532**	***By how much do you think VMMC reduces a man's risk of getting HIV infection?*** Ask for or convert response to a percent.	**Percent reduction**		%	
**Q533**	***Have many of your friends (or their male partners) had VMMC?***	**Yes**	1		
		**No**	2		
		**Don't know**	98		
**Q534**	***If you have a son who is a teenager or a young man, do you think it would be a good idea if he got VMMC?***		Teen-ager	Young man	
		**Yes**	1	1	**- Q536 if male respondent**
		**No**	2	2	
		**Don't know**	98	98	
**Q535**	***Do you think it is a good thing for your male partner to have VMMC?***	**Yes**	1		
		**No**	2		
		**Don't know**	98		

INDIVIDUAL QUESTIONNAIRE: **HIV PREVENTION METHODS** ***Q. No:***

REF.	QUESTIONS & FILTERS	CODING CATEGORIES		SKIP TO
Q536*	*The following statement is correct. Did you know this already?* 'Pre-exposure prophylaxis (PrEP) is when someone who does not have HIV takes a pill on an ongoing basis to prevent them getting HIV. Most people who use PrEP take a pill everyday. PrEP needs to be taken BEFORE sex for it to be effective.'	I knew this already	1	
		I wasn't sure about this	2	
		I didn't know about this	3	- Q566
		I don't understand this	4	- Q566
Q537**	*Have you ever taken PrEP?*	Yes	1	- Q539
		No	2	
Q538	*Have you ever been offered PrEP?*	Yes	1	
		No	2	- Q548
Q539**	*Are you taking PrEP currently?*	Yes	1	
		No	2	- Q545
Q540	*When did you start taking PrEP this time?* Emphasise this is for the current period of use.		mth / yr	
Q541	*Which of these organisations is providing you with PrEP?*		Yes / No	
		Ministry of Health	1 / 2	
		PSI	1 / 2	
		FACT	1 / 2	
		Other (specify)	1 / 2	
Q542	*How often have you taken PrEP in the last month?*	Every day	1	- Q546
		Most days	2	
		Occasionally	3	
		Never	4	
Q543	*What was the longest number of days that you didn't take the PrEP pills in the last month?*	Number of days		
Q544	*How many days in the last week (7 days) did you take the PrEP pills?*	Number of days		- Q546 if all 7 days
Q545	*Why did you stop using PrEP / not use PrEP on some days?*	High costs	1	
		Forgot to take pills	2	
		Ran out of pills	3	
		Judgemental staff / stigma	4	
		Lack of confidentiality	5	
		Limited opening hours	6	
		Distance / travel difficulties	7	
		Sexual partner(s) disapproved	8	
		Parents / elders / friends disapproved	9	
		Using another prevention method (specify method	10	
		Not currently sexually active	11	- Q548
		Abstaining or faithful to one trusted partner	12	
		Experienced side effects	13	
		Stock-outs of PrEP drugs at clinic	14	
		Other (specify)	15	
		Don't know or no particular reason	98	
Q546	*How often do you have a meal at the same time when you take PrEP?*	Always	1	
		Most of the time	2	
		Sometimes	3	
		Never	4	
Q547	*After you started taking PrEP, did you:*		More / Same / Less	
	(1) Increase, decrease or not change your number of sexual partners?		1 / 2 / 3	
	(2) Use condoms more, less or the same as before?		1 / 2 / 3	
	(3) Use condoms consistently with casual partners?	Yes	1	
		No	2	
		NA - no casual partners	99	
Q548	*Please tell me whether you strongly disagree, disagree, neither agree nor disagree, agree or strongly agree with the following statements …*		SD / D / N / A / SA	
	(1) I am confident I can use PrEP if I wanted to		1 / 2 / 3 / 4 / 5	
	(2) I am confident I can use PrEP even if I have to take it every day		1 / 2 / 3 / 4 / 5	
	(3) I am confident I can use PrEP even if I have to take it always after a meal		1 / 2 / 3 / 4 / 5	
	(4) I am confident I can use PrEP even if I have to hide it from my partner		1 / 2 / 3 / 4 / 5	
	(5) I am confident I can use PrEP even if my friends disapprove of this		1 / 2 / 3 / 4 / 5	
	(6) I am confident I can use PrEP even if my parents and family elders disapprove		1 / 2 / 3 / 4 / 5	
	(7) I am confident I can use PrEP even if my community would think I have HIV		1 / 2 / 3 / 4 / 5	
Q549	*Have you ever received instructions or counselling on how to use PrEP?*	Yes	1	
		No	2	

INDIVIDUAL QUESTIONNAIRE: **HIV PREVENTION METHODS** ***Q. No:***

REF.	QUESTIONS & FILTERS	CODING CATEGORIES		SKIP TO
Q550	*Are you able to discuss taking PrEP with your partner?*	Yes - already done so<br>Yes - not done already but can do this<br>No current sexual partner	1<br>2<br>8	
Q551	*Would / does you partner disapprove if you use PrEP?*	Yes<br>No<br>No current sexual partner	1<br>2<br>8	
Q552**	*If / when you want to use PrEP, do you know a place where someone like you can easily get it?*	Yes<br>No	1<br>2	
Q553*	*If / when you want to take PrEP yourself, how easy is it for you to access the service?*<br>Ask for an answer on a scale of 5 - from 1 (very easy) to 5 (very difficult).	Very easy<br>Easy<br>Neither easy nor difficult<br>Difficult<br>Very difficult<br>Don't know	1<br>2<br>3<br>4<br>5<br>98	
Q554	*What factors make it impractical or unsuitable for someone like you to access PrEP services?*<br>Ask first without probing and then probe for factors that are not mentioned spontaneously.	High costs<br>Lack of privacy / confidentiality<br>Embarrassed to go / ask<br>Limited opening hours<br>Distance / travel difficulties<br>Not suitable for men<br>Below eligible age for PrEP<br>Other (specify)<br>Not currently sexually active<br>Respondent is HIV+ (not suitable for PLHIV)	Spont / Probed	Q559 - if on PrEP now<br>- Q557<br>- Q562
Q555**	*Do you want to use PrEP if it was freely accessible to you?*	Yes<br>No<br>Not sure	1<br>2<br>8	
Q556*	*How definitely do you want to use PrEP if it was freely accessible to you?*<br>Ask for an answer on a scale of 5 - from 1 (definitely) to 5 (definitely not).	Definitely<br>Probably<br>No opinion<br>Probably not<br>Definitely not	1<br>2<br>3<br>4<br>5	
Q557	*Do you plan to start using PrEP?*	Yes<br>No<br>Don't know	1<br>2<br>98	 - Q559
Q558	*How soon do you plan to start using PrEP?*	Number of months	mths	
Q561	*How far away is it from where you live to the nearest place where PrEP services are provided?*	Distance in kms	kms	
Q562	*By how much do you think PrEP reduces a person's risk of getting HIV infection?*<br>Ask for or convert response to a percent.	Percent reduction	%	
Q563	*Are many of your friends (or their partners) using PrEP?*	Yes<br>No<br>Don't know	1<br>2<br>98	

Q559 *What factors were/are important in encouraging or discouraging you to use PrEP?*

For each factor given, ask if this was a positive (encouraging) or negative (discouraging) factor.

Ask first without probing and then probe for factors that are not mentioned spontaneously. Tick probed box to indicate probed responses.

Factor	Pos	Neg	Neutral	Probed
HIV/STI protection	1	2	8	
Method of HIV prevention I can control	1	2	8	
Allow me to become pregnant if I want	1	2	8	
Possible side effects	1	2	8	
Don't think PrEP works	1	2	8	
High costs involved	1	2	8	
Make me feel responsible or ashamed	1	2	8	
Inconvenient to take pills daily	1	2	8	
Using other prev'n method (note method)	1	2	8	
Faithful to one trusted partner	1	2	8	
Not currently sexually active	1	2	8	
Other (specify) ______________	1	2	8	

Q560 *Which people's views were/are important in encouraging or discouraging you to use PrEP?*

Instructions as for last question.

For partner/friends' views - if mentioned spontaneously, ask for the reason.

Factor	Pos	Neg	Neutral	Probed
Religious leaders (religious beliefs)	1	2	8	
Parents' or family elders' approval/views	1	2	8	
Partner(s) will think I have HIV	1	2	8	
Partner's views (other)	1	2	8	
Friends / community think I have HIV	1	2	8	
Friends / community views (other)	1	2	8	
Other (specify) ______________	1	2	8	

INDIVIDUAL QUESTIONNAIRE: **HIV PREVENTION METHODS** *Q. No:*

REF.	QUESTIONS & FILTERS	CODING CATEGORIES			SKIP TO
**Q564**	*If you have or had a daughter (or sister) who is a teenager or a young woman and she started having sex before getting married, do you think it would be a good thing for her to use PrEP?*		Teen-ager	Young woman	
		Yes	1	1	
		No	2	2	
		Don't know	98	98	
**Q565**	*If your sexual partner wanted to use PrEP, would you agree to him or her doing this?*		Non-reg	Regular	
		Yes	1	1	
		No	2	2	
	Ask first for non-regular partners (if any).	Don't know	98	98	
	Then for regular partners.	NA	99	99	
**Q566****	*How often have you used a male condom when you had sexual intercourse with your REGULAR partner or partners in the last 2 weeks?*	Every time	1		
		Most times	2		
		Occasionally	3		
		Never	4		
	Emphasise asking about male condoms only here.	NA - no regular partner	99		
**Q567****	*How often have you used a male condom when you had sexual intercourse with NON-REGULAR partners in the last 2 weeks?*	Every time	1		- Q569 if 'every time' for reg & non-regs
		Most times	2		
		Occasionally	3		
		Never	4		
		NA - no non-regular partners	99		
**Q568**	*Why did you not use male condoms on some occasions when you had sex in the last 2 weeks?*	High costs	1		
		Judgemental staff / stigma	2		
		Lack of privacy / confidentiality	3		
		Limited times when accessible	4		
		Distance / travel difficulties to access	5		
		Sexual partner(s) disapproved	6		
		Parents / elders / friends disapproved	7		
		Condoms reduce pleasure of sex	8		
		Condoms not effective	9		
	______	Using different prevention method (specify metho	10		
		Faithful to one trusted partner	11		
	______	Other (specify)	12		
		None	96		
		Don't know	98		
**Q569**	*Has a male condom ever broken when you were using it?*	Yes	1		
		No	2		- Q571
		NA - not using male condoms	99		- Q571
**Q570**	*What do you do when male condoms break?*	Replace the condom with a new one	1		
		Continue without replacing the condom	2		
	______	Other (specify)	99		
**Q571**	*After you started using male condoms, did you increase or decrease your number of sexual partners?*	Increase	1		
		Decrease	2		
		No change	8		
		Never used male condoms	99		

Q572 *Please tell me whether you strongly disagree, disagree, neither agree nor disagree, agree or strongly agree with the following statements …*

Statement	SD	D	N	A	SA
(1) I am confident I can use male condoms if I wanted to	1	2	3	4	5
(2) I am confident I can use male condoms even if I have to use them every time	1	2	3	4	5
(3) I am confident I can use male condoms even if my partner dislikes/disapproves	1	2	3	4	5
(4) I am confident I can use male condoms even if I'm drunk or have taken drugs	1	2	3	4	5
(5) I am confident I can use male condoms even if my friends disapprove	1	2	3	4	5
(6) I am confident I can use male condoms even if my parents and family elders disapprove	1	2	3	4	5

REF.	QUESTIONS & FILTERS	CODING CATEGORIES		SKIP TO
**Q573**	*Have you ever received instructions or counselling on how to use male condoms?*	Yes	1	
		No	2	
**Q574**	*Are you able to discuss using male condoms with your REGULAR partner?*	Yes - already done so	1	
		Yes - not done already but can do this	2	
		No	3	
		Never had sex	97	- Q580
		No regular partner	99	- Q577
**Q575**	*Would / does your regular partner disapprove if you use male condoms?*	Yes	1	
		No	2	
		Don't know	98	
**Q576**	*If your regular partner doesn't want to use male condoms, are you able to refuse to have sex with him/her?*	Yes - already done so	1	
		Yes - not done already but can do this	2	
		No	3	
		Don't know	98	
**Q577**	*Are you able to discuss using male condoms with your NON-REGULAR partner(s)?*	Yes - already done so	1	
		Yes - not done already but can do this	2	
		No	3	
		No non-regular partners	99	- Q580

Davis K, *et al. BMJ Open* 2023; 13:e067327. doi: 10.1136/bmjopen-2022-067327

INDIVIDUAL QUESTIONNAIRE: **HIV PREVENTION METHODS** *Q. No:*

REF.	QUESTIONS & FILTERS	CODING CATEGORIES		SKIP TO
**Q578**	***Would / do your non-regular partners disapprove if you use male condoms?***	**Yes**<br>**No**<br>**Don't know**	1<br>2<br>98	
**Q579**	***If a non-regular partner doesn't want to use male condoms, are you able to refuse to have sex with him/her?***	**Yes - already done so**<br>**Yes - not done already but can do this**<br>**No**	1<br>2<br>3	
**Q580****	***If / when you want to use male condoms, do you know a place where someone like you can easily get them?***	**Yes**<br>**No**	1<br>2	
**Q581***	***If / when you want to use male condoms yourself, how easy is it for you to access them?***<br>**Ask for an answer on a scale of 5 - from 1 (very easy) to 5 (very difficult).**	**Very easy**<br>**Easy**<br>**Neither easy nor difficult**<br>**Difficult**<br>**Very difficult**	1<br>2<br>3<br>4<br>5	

REF.	QUESTIONS & FILTERS	Place	Yes	No	DK
**Q582**	***Do you feel able to access male condoms from the following places?***<br>**Ask each in turn.**	**Health clinic**	1	2	98
		**Community-based distributor**	1	2	98
		**Bars or beer halls**	1	2	98
		**Shops**	1	2	98
		**Your sexual partner(s)**	1	2	98
		**Friends**	1	2	98
		**Other (specify)**	1	2	98

REF.	QUESTIONS & FILTERS	Factor	Spont	Probed
**Q583**	***What factors make it impractical or unsuitable for someone like you to access male condoms?***<br>**Ask first without probing and then probe for factors that are not mentioned spontaneously.**	**High costs**		
		**Lack of privacy / confidentiality**		
		**Embarrassed to go / ask**		
		**Limited opening hours**		
		**Distance / travel difficulties**		
		**Other (specify)**		

REF.	QUESTIONS & FILTERS	CODING CATEGORIES		SKIP TO
**Q584****	***Do you want to use male condoms with your regular partner(s) if they were freely accessible to you?***<br>**Stress: no right or wrong answer.**	**Yes**<br>**No**<br>**Not sure**<br>**NA - no regular partner**	1<br>2<br>98<br>99	 <br><br>**- Q587**
**Q585***	***How definitely do you want to use male condoms with your regular partner(s) if they were freely accessible to you?***<br>**Ask for an answer on a scale of 5 - from 1 (definitely) to 5 (definitely not).**	**Definitely**<br>**Probably**<br>**No opinion**<br>**Probably not**<br>**Definitely not**	1<br>2<br>3<br>4<br>5	
**Q586**	***Do you plan to use male condoms the next time you have sex with your regular partner?***	**Yes**<br>**No**<br>**Don't know**	1<br>2<br>98	
**Q587****	***Do you want to use male condoms with your non-regular partner(s) if they were freely accessible to you?***<br>**Stress: no right or wrong answer.**	**Yes**<br>**No**<br>**Not sure**<br>**NA - no non-regular partner(s)**	1<br>2<br>98<br>99	 <br><br>**- Q590**
**Q588***	***How definitely do you want to use male condoms with your non-regular partner(s) if they were freely accessible to you?***<br>**Ask for an answer on a scale of 5 - from 1 (definitely) to 5 (definitely not).**	**Definitely**<br>**Probably**<br>**No opinion**<br>**Probably not**<br>**Definitely not**	1<br>2<br>3<br>4<br>5	
**Q589**	***Do you plan to use male condoms the next time you have sex with a non-regular partner?***<br>**Stress: no right or wrong answer.**	**Yes**<br>**No**<br>**Don't know**	1<br>2<br>98	

REF.	QUESTIONS & FILTERS	Factor	Pos	Neg	Neutral	Probed
**Q590**	***What factors were/are important in encouraging or discouraging you to use male condoms?***<br>**For each factor given, ask if this was a positive (encouraging) or negative (discouraging) factor.**<br>**Ask first without probing and then probe for factors that are not mentioned spontaneously.**<br>**Tick probed box to indicate probed responses.**	**HIV/STI protection**	1	2	8	
		**HIV/STI protection (for my partner(s))**	1	2	8	
		**Method of HIV prevention I can control**	1	2	8	
		**Reduce risk of pregnancy (self/partner)**	1	2	8	
		**Own sexual pleasure (better/worse)**	1	2	8	
		**Partner's sexual pleasure (better/worse)**	1	2	8	
		**Make me feel responsible or ashamed**	1	2	8	
		**Faithful to one trusted partner**	1	2	8	
		**Using other prev'n method (note method)**	1	2	8	
		**Not currently sexually active**	1	2	8	
		**Other (specify) ______________**	1	2	8	

Davis K, *et al. BMJ Open* 2023; 13:e067327. doi: 10.1136/bmjopen-2022-067327

INDIVIDUAL QUESTIONNAIRE: **HIV PREVENTION METHODS** *Q. No:*

REF.	QUESTIONS & FILTERS	CODING CATEGORIES	SKIP TO
Q591	*Which people's views were/are important in encouraging or discouraging you to use male condoms?*<br><br>Instructions as for last question.<br><br>For partner/friends' views - if mentioned spontaneously, ask for the reason.	Pos / Neg / Neutral / Probed<br>Religious leaders (religious beliefs) 1 2 8<br>Parents' or family elders' approval/views 1 2 8<br>Partner(s) will think I have HIV 1 2 8<br>Partner will think I have other partners 1 2 8<br>Partner's views (other) 1 2 8<br>Friends / community think I have HIV 1 2 8<br>Friends / community views (other) 1 2 8<br>Other (specify) ______________ 1 2 8	
Q592	*By how much do you think male condoms reduce a person's risk of getting HIV infection?*<br>Ask for or convert response to a percent.	Percent reduction ___ %	
Q593	*How far away is it from where you live to the nearest place where male condoms can be obtained?*	Distance in kms ___ kms	
Q594	*Are many of your friends (or their partners) using male condoms?*	Yes 1<br>No 2<br>Don't know 98	
Q595	*In what circumstances do you think it is acceptable for a husband and wife to use condoms?*	Yes / No<br>Always 1 2<br>If one of them is HIV+ 1 2<br>If one spouse has other partners 1 2<br>If one spouse has an STD 1 2<br>To avoid pregnancy 1 2<br>Other (specify) 1 2	
Q596	*If you have or had a daughter (or sister) who was a teenager or young woman and she started having sex before getting married, do you think it would be a good thing for her to use male condoms with her partner?*	Teen-ager / Young woman<br>Yes 1 1<br>No 2 2<br>Don't know 98 98	
	"Now I would like to ask you some questions about FEMALE condoms …"		
Q597**	*How often have you used a female condom when you had sexual intercourse with your REGULAR partner or partners in the last 2 weeks?*	Every time 1<br>Most times 2<br>Occasionally 3<br>Never 4<br>NA - no regular partner 99<br>Not heard of female condoms 97	 <br><br><br><br>- Q59929
Q598**	*How often have you used a female condom when you had sexual intercourse with NON-REGULAR partners in the last 2 weeks?*	Every time 1<br>Most times 2<br>Occasionally 3<br>Never 4<br>NA - no non-regular partners 99	
Q599	*Why did you not use female condoms on some occasions when you had sex in the last 2 weeks?*	High costs 1<br>Difficult to use 2<br>Judgemental staff / stigma 3<br>Lack of privacy / confidentiality 4<br>Limited times when accessible 5<br>Distance / travel difficulties 6<br>Sexual partner(s) disapproved 7<br>Parents / elders / friends disapproved 8<br>Female condoms reduce pleasure of sex 9<br>Female condoms not effective 10<br>Using different prevention method (specify methoo 11<br>Faithful to one trusted partner 12<br>Other (specify) 13<br>None 96<br>Don't know 98	
Q59901	*Has a female condom ever broken when you were using it?*	Yes 1<br>No 2<br>NA - not using male condoms 99	 - Q59903<br>- Q59903
Q59902	*What do you do when female condoms break?*	Replace the condom with a new one 1<br>Continue without replacing the condom 2<br>Other (specify) 99	
Q59903	*After you started using female condoms, did you increase or decrease your number of sexual partners?*	Increase 1<br>Decrease 2<br>No change 8<br>Never used female condoms 99	

Davis K, *et al. BMJ Open* 2023; 13:e067327. doi: 10.1136/bmjopen-2022-067327

INDIVIDUAL QUESTIONNAIRE: **HIV PREVENTION METHODS** *Q. No:*

REF.	QUESTIONS & FILTERS	CODING CATEGORIES		SKIP TO
Q59904	*Please tell me whether you strongly disagree, disagree, neither agree nor disagree, agree or strongly agree with the following statements …*	SD D N A SA		
	(1) I am confident I can use female condoms if I wanted to		1 2 3 4 5	
	(2) I am confident I can use female condoms even if I have to use them every time		1 2 3 4 5	
	(3) I am confident I can use female condoms even if my partner dislikes/disapproves		1 2 3 4 5	
	(4) I am confident I can use female condoms even if I'm drunk or have taken drugs		1 2 3 4 5	
	(5) I am confident I can use female condoms even if my friends disapprove of this		1 2 3 4 5	
	(6) I am confident I can use female condoms even if my parents and family elders disapprove		1 2 3 4 5	
Q59905	*Have you ever received instructions or counselling on how to use female condoms?*	Yes	1	
		No	2	
Q59906	*Are you able to discuss using female condoms with your REGULAR partner?*	Yes - already done so	1	
		Yes - not done already but can do this	2	
		No	3	
		No regular partner	8	- Q59909
		Never had sex	99	- Q59912
Q59907	*Would / does your regular partner disapprove if you use female condoms?*	Yes	1	
		No	2	
		Don't know	98	
Q59908	*If your regular partner doesn't want to use female condoms, are you able to refuse to have sex with him/her?*	Yes - already done so	1	
		Yes - not done already but can do this	2	
		No	3	
		Don't know	98	
Q59909	*Are you able to discuss using female condoms with your NON-REGULAR partner(s)?*	Yes - already done so	1	
		Yes - not done already but can do this	2	
		No	3	
		No non-regular partners	99	- Q59912
Q59910	*Would / do your non-regular partners disapprove if you use female condoms?*	Yes	1	
		No	2	
		Don't know	98	
Q59911	*If a non-regular partner doesn't want to use female condoms, are you able to refuse to have sex with him/her?*	Yes - already done so	1	
		Yes - not done already but can do this	2	
		No	3	
Q59912[‡]	*If / when you want to use female condoms, do you know a place where someone like you can easily get them?*	Yes	1	
		No	2	
Q59913[‡]	*If / when you want to use female condoms yourself, how easy is it for you to access them?* Ask for an answer on a scale of 5 - from 1 (very easy) to 5 (very difficult).	Very easy	1	
		Easy	2	
		Neither easy nor difficult	3	
		Difficult	4	
		Very difficult	5	
Q59914	*Do you feel able to access female condoms from the following places?* Ask each in turn.		Yes No DK	
		Health clinic	1 2 98	
		Community-based distributor	1 2 98	
		Bars or beer halls	1 2 98	
		Shops	1 2 98	
		Your sexual partner(s)	1 2 98	
		Friends	1 2 98	
		Other (specify)	1 2 98	
Q59915*	*What factors make it impractical or unsuitable for someone like you to access female condoms?* Ask first without probing and then probe for factors that are not mentioned spontaneously.		Spont Probed	
		High costs		
		Lack of privacy / confidentiality		
		Embarrassed to go / ask		
		Limited opening hours		
		Distance / travel difficulties		
		Other (specify)		
Q59916	*Do you want to use female condoms with your regular partner(s) if they were freely accessible to you?*	Yes	1	
		No	2	
		Not sure	98	
		NA - no regular partner	99	- Q59919
Q59917*	*How definitely do you want to use female condoms with your regular partner(s) if they were freely accessible to you?* Ask for an answer on a scale of 5 - from 1 (definitely) to 5 (definitely not).	Definitely	1	
		Probably	2	
		No opinion	3	
		Probably not	4	
		Definitely not	5	

INDIVIDUAL QUESTIONNAIRE: **HIV PREVENTION METHODS** ***Q. No:***

REF.	QUESTIONS & FILTERS	CODING CATEGORIES		SKIP TO
Q59918*	*Do you plan to use female condoms the next time you have sex with your regular partner?*	Yes<br>No<br>Don't know	1<br>2<br>98	
Q59919*	*Do you want to use female condoms with your non-regular partner(s) if they were freely accessible to you?*	Yes<br>No<br>Not sure<br>NA - no non-regular partner	1<br>2<br>98<br>99	 <br><br>- Q59922
Q59920*	*How definitely do you want to use female condoms with your non-regular partner(s) if they were freely accessible to you?*<br>Ask for an answer on a scale of 5 - from 1 (definitely) to 5 (definitely not).	Definitely<br>Probably<br>No opinion<br>Probably not<br>Definitely not	1<br>2<br>3<br>4<br>5	
Q59921*	*Do you plan to use female condoms the next time you have sex with a non-regular partner?*	Yes<br>No<br>Don't know	1<br>2<br>98	

Q59922 *What factors were/are important in encouraging or discouraging you to use female condoms?*

For each factor given, ask if this was a positive (encouraging) or negative (discouraging) factor.

Ask first without probing and then probe for factors that are not mentioned spontaneously.

Tick probed box to indicate probed responses.

	Pos	Neg	Neutral	Probed
HIV/STI protection	1	2	8	
HIV/STI protection (for my partner(s))	1	2	8	
Method of HIV prevention I can control	1	2	8	
Reduce risk of pregnancy (self/partner)	1	2	8	
Own sexual pleasure (better/worse)	1	2	8	
Partner's sexual pleasure (better/worse)	1	2	8	
Make me feel responsible or ashamed	1	2	8	
Difficult to use	1	2	8	
Fear of losing condom inside vagina	1	2	8	
Faithful to one trusted partner	1	2	8	
Using other prev'n method (note method)	1	2	8	
Not currently sexually active	1	2	8	
Other (specify) ______________	1	2	8	

Q59923 *Which people's views were/are important in encouraging or discouraging you to use female condoms?*

Instructions as for last question.

For partner/friends' views - if mentioned spontaneously, ask for the reason.

	Pos	Neg	Neutral	Probed
Religious leaders (religious beliefs)	1	2	8	
Parents' or family elders' approval/views	1	2	8	
Partner(s) will think I have HIV	1	2	8	
Partner will think I have other partners	1	2	8	
Partner's views (other)	1	2	8	
Friends / community think I have HIV	1	2	8	
Friends / community views (other)	1	2	8	
Other (specify) ______________	1	2	8	

REF.	QUESTIONS & FILTERS	CODING CATEGORIES		SKIP TO
Q59923<br>Q59924	*What are the main reasons that would/did*<br>*By how much do you think female condoms reduce a person's risk of getting HIV infection?*<br>Ask for or convert response to a percent.	Percent reduction	Spont Probed<br>%	
Q59925	*How far away is it from where you live to the nearest place where female condoms can be obtained?*	Distance in kms	kms	
Q59926	*Are many of your friends (or their partners) using female condoms?*	Yes<br>No<br>Don't know	1<br>2<br>98	

Q59927 *In what circumstances do you think it is acceptable for a husband and wife to use female condoms?*

	Yes	No
Always	1	2
If one of them is HIV+	1	2
If one spouse has other partners	1	2
If one spouse has an STD	1	2
To avoid pregnancy	1	2
Other (specify)	1	2

Q59928* *If you have or had a daughter (or sister) who was teenager or young woman and she started having sex before getting married, do you think it would be a good thing for her to use female condoms with her partner?*

	Teen-ager	Young woman
Yes	1	1
No	2	2
Don't know	98	98

REF.	QUESTIONS & FILTERS	CODING CATEGORIES		SKIP TO
Q59929*	*Are you currently sticking to one regular sexual partner or not having sex?*<br>Stress: no right or wrong answer.	Yes - one regular partner<br>Yes - not having sex<br>Neither	1<br>2<br>3	 <br>- Q59931
Q59930	*For how long have you been sticking to one regular partner or not having sex?*	Number or months or years	yrs mths	

INDIVIDUAL QUESTIONNAIRE: **HIV PREVENTION METHODS** ***Q. No:***

REF.	QUESTIONS & FILTERS	CODING CATEGORIES		SKIP TO
Q59931	***Why are you currently having more than one sexual partner?***	**Multiple wives**<br>**Sex worker**<br>**Financial or material benefits / reduce poverty**<br>**Sexual pleasure**<br>**Emotional attachment (love)**<br>**Social status (impress peers etc.)**<br>**Other (specify)**<br>**Don't know**	1<br>2<br>3<br>4<br>5<br>6<br>7<br>98	
Q59932*	***Please tell me whether you strongly disagree, disagree, neither agree nor disagree, agree or strongly agree with the following statements …***<br>**(1) I am confident I can stick to one partner if I wanted to.**<br>**(2) I am confident I can stick to one partner even if my friends encourage me to have other partners**<br>**(3) I am confident I can stick to one partner even if I lose financial or material benefits**	SD D N A SA<br>1 2 3 4 5<br>1 2 3 4 5<br>1 2 3 4 5		
Q59933	***If you wanted to stick to one partner, how easy would this be for you to do?***<br><u>Ask for an answer on a scale of 5 - from 1 (very easy) to 5 (very difficult).</u>	**Very easy**<br>**Easy**<br>**Neither easy nor difficult**<br>**Difficult**<br>**Very difficult**	1<br>2<br>3<br>4<br>5	
Q59934	***What factors make it impractical or unsuitable for someone like you to stick to one partner?***<br><u>Ask first without probing and then probe for factors that are not mentioned spontaneously.</u>	**Multiple wives**<br>**Poverty**<br>**Threat of violence from partners**<br>**Peer pressure**<br>**Other (specify)**	Spont Probed	
Q59935	***How definitely do you want to stick to one partner in the near future?***<br><u>Ask for an answer on a scale of 5 - from 1 (definitely) to 5 (definitely not).</u>	**Definitely**<br>**Probably**<br>**No opinion**<br>**Probably not**<br>**Definitely not**	1<br>2<br>3<br>4<br>5	
Q59936	***Do you plan to start sticking to one partner or to stop having sex?***<br><u>Stress: no right or wrong answer.</u>	**Yes - one regular partner**<br>**Yes - stop having sex**<br>**Neither**<br>**Don't know**	1<br>2<br>3<br>98	 <br>- Q59938<br>- Q59938
Q59937	***How soon do you plan to make this change?***	**Number of months**	mths	
Q59938	***What are the main reasons that would/did motivate you to stop having sex?***<br><u>Ask first without probing and then probe for factors that are not mentioned spontaneously.</u>	**Reduce risk of HIV & STDs (self/partner)**<br>**Reduce risk of pregnancy (self or partner)**<br>**Would feel a responsible person**<br>**Poor health**<br>**Children (daughters) now sexually active**<br>**Feel too tired / old**<br>**Other (specify)**	Spont Probed	
Q59939	***What are the main reasons that would/did discourage you from stopping having sex?***<br><u>Ask first without probing and then probe for factors that are not mentioned spontaneously.</u>	**Enjoy having sex**<br>**Desire/need to have (more) children**<br>**Partner would think I have HIV**<br>**Partner would leave me**<br>**Threat of violence from partner(s)**<br>**Friends would disapprove**<br>**Parents / family elders would disapprove**<br>**Community members would disapprove**<br>**Other (specify)**	Spont Probed	
Q59940	***What are the main reasons that would/did motivate you to stick to one partner?***<br><u>Ask first without probing and then probe for factors that are not mentioned spontaneously.</u>	**Reduce risk of HIV infection & STDs**<br>**Would feel a responsible person**<br>**Regular partner would approve**<br>**Parents / family elders would approve**<br>**Friends would approve**<br>**Other (specify)**	Spont Probed	
Q59941	***What are the main reasons that would/did discourage you from sticking to one partner?***<br><u>Ask first without probing and then probe for factors that are not mentioned spontaneously.</u>	**Sexual pleasure from having multiple partners**<br>**Loss of financial or material benefits**<br>**Loss of reputation / status in the community**<br>**Already married to multiple wives**<br>**Other (specify)**	Spont Probed	
Q59942	***Have you ever received training or counselling to reduce your number of sexual partners?***	**Yes - organisation code (Q504)**<br>**No**	1<br>2	
Q59943	***Would your sexual partner(s) accept it if you left him/her/them?***	**Yes**<br>**No**	1<br>2	

INDIVIDUAL QUESTIONNAIRE: **HIV AWARENESS & SOCIAL NORMS** ***Q. No:***

REF.	QUESTIONS & FILTERS	CODING CATEGORIES			SKIP TO
**Q601**	***"Now I would like to ask you some questions about HIV and AIDS …"***				
**Q602**	***Please tell me all the ways that an adult can get HIV infection?*** **Tick 1 for each way mentioned spontaneously.** ***Are there any other ways?*** **Then proceed down the column, reading the description of each possible way not mentioned spontaneously. Make entries in "probed" column as follows:** 1/11 Yes, HIV can be transmitted this way (spont / yes when probed) 2/12 No, HIV cannot be transmitted this way (spont / yes when probed) 98 Don't know		Spont	Probed	
		**Sex with a person with HIV/AIDS**	☐	☐	
		**Touching a person with AIDS**	☐	☐	
		**Mosquito bites**	☐	☐	
		**Blood transfusion**	☐	☐	
		**Injection with a dirty needle**	☐	☐	
		**Sharing utensils with person with HIV/AIDS**	☐	☐	
		**Ritual scarification**	☐	☐	
		**Punishment from God**	☐	☐	
		**Other (specify)** ______	☐	☐	
**Q603**	***Do you know of any factors which are likely to INCREASE the chances that a person will get HIV infection?*** **Ask in same way as Q602.** Codes as in Q602.		Spont	Probed	
		**Sex with a prostitute**	☐	☐	
		**Many sex partners**	☐	☐	
		**Not being circumcised (for men)**	☐	☐	
		**Other STDs present**	☐	☐	
		**Using condoms**	☐	☐	
		**Witchcraft or spiritual curse**	☐	☐	
		**Other (specify)**	☐	☐	
**Q604**	***What are the ways in which an infant or child could have become infected with HIV?*** **Ask in same way as Q602.** Codes as in Q602.		Spont	Probed	
		**At birth - if mother infected**	☐	☐	
		**Witchcraft or spiritual curse**	☐	☐	
		**Mosquito bites**	☐	☐	
		**Injection with a dirty needle**	☐	☐	
		**Breastfed by infected woman not on ART**	☐	☐	
		**Blood transfusion**	☐	☐	
		**From an infected father**	☐	☐	
		**Other (specify)**	☐	☐	
**Q605**	***Are ALL babies born to women who have HIV born with the infection?***	**Yes**	1	☐	
		**No**	2	☐	
		**Don't know**	98	☐	
**Q606**	***Can all people infected with HIV be identified by looking at them?***	**Yes**	1	☐	
		**No**	2	☐	
		**Don't know**	98	☐	
**Q607**	***Would you be willing to take care of a family member with AIDS?***	**Yes**	1	☐	- **Q609**
		**No**	2	☐	
		**Don't know**	98	☐	
**Q608**	***Why would you not be willing to take care of a family member with AIDS?***	**Not enough time**	1	☐	
		**Too few resources**	2	☐	
		**Not enough space**	3	☐	
		**Too young**	4	☐	
		**Not experienced enough**	5	☐	
		**Unwell myself**	6	☐	
		**Frightened of being exposed to HIV**	7	☐	
		**Reluctant to be associated with people living with HIV**	8	☐	
		**Other (specify)**	12	☐	
**Q609**	***How long does it usually take for a person infected with HIV to develop symptoms if they are not on ART?***	**Number of years or months**		yrs ☐ mths ☐	
		**Don't know**	998	☐	
**Q610**	***How many people do you know who either died from HIV/AIDS or have the disease now?***	**Number (>0)**		mths ☐	
		**Doesn't know of any**	998	☐	
**Q611**	***Of these people, how many live(d) in your household; in the same village/town; and how many live(d) somewhere else?*** **Enter numbers of people in each category.**	**Household**		☐	
		**Village / town**		☐	
		**Somewhere else**		☐	
		**Check that these sum to same as in Q510.**			

INDIVIDUAL QUESTIONNAIRE: **HIV AWARENESS & SOCIAL NORMS** *Q. No:*

REF.	QUESTIONS & FILTERS	CODING CATEGORIES		SKIP TO
Q612	*What was your relationship to each of these people?*<br>Enter numbers of people in each category.	Spouse/partner<br>Father or mother<br>Son or daughter<br>Other relative<br>Friend or neighbour<br>Work colleague<br>Someone else		
Q613	*Did you help to take care of any of these people on a daily basis?*	Yes<br>No	1<br>2	
Q614	*What are the chances that you are infected with HIV now?*	Certain / almost certain<br>High<br>Moderate<br>Small<br>None	1<br>2<br>3<br>4<br>5	
Q615	*What are the chances that you will become infected with HIV in the next 12 months if you are not infected now but continue with your current behaviour?*	Certain / almost certain<br>High<br>Moderate<br>Small<br>None	1<br>2<br>3<br>4<br>5	 <br><br><br>- Q617
Q616	*Why do you think you might become infected?*	Has multiple sex partners now<br>Not using condoms<br>Regular partner has other partners<br>Future partner may have other partners<br>Partner may be infected with HIV<br>Many friends/relatives dying of HIV/AIDS<br>Caring for an infected person<br>Other (specify)	1<br>2<br>3<br>4<br>5<br>6<br>7<br>8	

Q617 *For the following statements, please tell me whether you strongly disagree, disagree, neither agree nor disagree, agree or strongly agree …*

	SD	D	N	A	SA
(1) I can get HIV even if I only have sex with one	1	2	3	4	5
(2) I have never done anything that could have exposed me to HIV	1	2	3	4	5
(3) I would rather die a violent death (e.g. gunshot or car accident) than from HIV	1	2	3	4	5
(4) I feel vulnerable to HIV infection	1	2	3	4	5
(5) I am less likely to get HIV than my friends	1	2	3	4	5
(6) I'm worried that one of my current sexual partners may give me HIV	1	2	3	4	5
(7) I think about my chances of getting HIV whenever I have a new sexual partner	1	2	3	4	5
(8) Getting (a woman) pregnant when you don't want to is worse than getting infected with	1	2	3	4	5

REF.	QUESTIONS & FILTERS	CODING CATEGORIES		SKIP TO
Q618	*How often do you worry about getting infected with HIV?*	Never<br>Rarely<br>Sometimes<br>Often<br>Almost all the time	1<br>2<br>3<br>4<br>5	
Q619	*Who do you think is more likely to be infected with HIV in Manicaland: a 15-19 year-old man or a 25-29 year-old man?*	15-19 year-old man<br>25-29 year-old man	1<br>2	
Q620	*Who do you think is more likely to be infected with HIV in Manicaland: a 15-19 year-old woman or a 25-29 year-old man?*	15-19 year-old woman<br>25-29 year-old man	1<br>2	
Q621	*Would you buy fresh vegetables from a shopkeeper or vendor if you knew this person had HIV?*	Yes<br>No<br>Don't know/not sure/it depends	1<br>2<br>98	
Q622	*Do you think that children living with HIV should be able to attend school with children who are HIV negative?*	Yes<br>No<br>Don't know/not sure/it depends	1<br>2<br>98	
Q623	*If you thought your spouse/regular partner was having sexual intercourse with (a) casual partner(s) without using condoms, could you persuade him/her to stop?*	Yes<br>No<br>No regular partner<br>Don't know	1<br>2<br>96<br>98	

INDIVIDUAL QUESTIONNAIRE: **HIV AWARENESS & SOCIAL NORMS** *Q. No:*

REF.	QUESTIONS & FILTERS	CODING CATEGORIES			SKIP TO
Q624	*Do you think there are things you can do which will prevent you from becoming infected with HIV in the future?*	Yes<br>No<br>Don't know<br>NA - already infected	1<br>2<br>98<br>99		
Q625	*Which of these statements do you agree with?*		Agree	Disagree	
	(1) Men have a sex drive that needs to be satisfied		1	2	
	(2) A man will lose respect if he admits to being sick		1	2	
	(3) Men who take sick children to the hospital, or cook at home should be proud of what they do		1	2	
	(4) Men are strong and therefore less likely to need a doctor		1	2	
	(5) Men are always ready for sex		1	2	
	(6) A man should not go with his partner for antenatal check-ups at the local clinic		1	2	
	(7) If a man is sick, he should not let others see he is in pain		1	2	
	(8) It is appropriate for a woman to be the primary breadwinner of a household		1	2	
	(9) A real man enjoys a bit of risk taking now and then		1	2	
	(10) Men should have had several sexual partners before they get married		1	2	
	Read out each in turn.				
Q626	*Which of these statements would you say was true?*		True	Not	
	(1) Minor illnesses can be fought off if you don't give in to it		1	2	
	(2) Men feel comfortable going to the hospital and have no problems seeking help		1	2	
	(3) There is no need to go and see a doctor unless you are very ill		1	2	
	(4) A man should make sure that he can recognise the early signs of an STI		1	2	
	(5) It is important for a man living with HIV to be on anti-retroviral therapy		1	2	
	(6) A man who goes to the hospital is considered weak		1	2	
	(7) A man gain respect if he goes for regular health checks		1	2	
	(8) Men get embarrassed if a brother is found to be HIV positive		1	2	
	Read out each in turn.				
Q627	*Which of these statements do you agree with?*		Agree	Disagree	
	(1) It is a good idea to make condoms available for young people in schools		1	2	
	(2) If I have / had a teenage daughter, I would tell her about condoms		1	2	
	(3) If I have / had a teenage daughter, I would tell her about PrEP		1	2	
	(4) If I have / had a teenage daughter and thought she might be having sex, I would encourage her to use PrEP and condoms		1	2	
			1	2	
	(5) If I have / had a teenage daughter and told her not to have sex until she gets married, she would comply		1	2	
	(6) If a young woman is married and her husband has HIV, she should use PrEP or condoms		1	2	
	(7) If I have / had a teenage daughter and she had sex before marriage, I would be OK with this		1	2	
	(8) Many young women have sex before marriage these days		1	2	
	(9) If I have / had a teenage son, I would encourage him to have medical circumcision		1	2	
	(10) I pay/get paid for sex because my friends do and because they encourage me		1	2	
Q628	*Do you think a lot of young people are still getting infected with HIV these days?*	Yes<br>No	1<br>2		- Q630 if<br>- male
Q629	*Have you experienced any of the following from a male intimate partner in the past 12 months?*		Yes	No	
	(1) Slapped you or threw something at you that could hurt you		1	2	
	(2) Pushed or shoved you		1	2	
	(3) Hit you with a fist or something else that could hurt you		1	2	
	(4) Kicked or dragged you or beat you up		1	2	
	(5) Choked or burnt you		1	2	
	(6) Threatened or used a gun, knife or other weapon against you		1	2	
	(7) Physically forced you to have sexual intercourse against your will		1	2	
	(8) Forced you to do something sexual she found degrading or humiliating		1	2	
	(9) Made you afraid of what would happen if you did not have sexual intercourse		1	2	
Q630	*Imagine that we flipped a fair coin 10 times. The first 9 times, the coin comes down 'heads'. What are the chances that the 10th flip will also be 'heads'?*	Percentage chance	%		

INDIVIDUAL QUESTIONNAIRE: **HIV AWARENESS & SOCIAL NORMS** ***Q. No:***

REF.	QUESTIONS & FILTERS	CODING CATEGORIES		SKIP TO
Q631	**I will ask you several questions about the chance or likelihood that certain events are going to happen. There are 10 beans in the cup. I would like you to choose some beans out of these 10 beans and put them in the plate to express what you think the likelihood or chance is of a specific event happening. One bean represents one chance out of 10. If you do not put any beans in the plate, it means you are sure that the event will NOT happen. As you add beans, it means that you think the likelihood that the event happens increases. For example, if you put one or two beans, it means you think the event is not likely to happen but it is still possible. If you pick 5 beans, it means that it is just as likely it happens as it does not happen (fifty-fifty). If you pick 6 beans, it means the event is slightly more likely to happen than not to happen. If you put 10 beans in the plate, it means you are sure the event will happen. There is no right or wrong answer, I just want to know what you think.**<br><br>**Let me give you an example. Imagine that we are playing draughts. Say, when asked about the chance that you will win, you put 7 beans in the plate. This means that you believe you would win 7 out of 10 games on average if we play for a long time.**			
Q632	**Report for each question the NUMBER OF BEANS put in the PLATE.**<br>**After each question, replace the beans in the cup (unless otherwise noted).**<br><br>**For questions Q633 to Q635: If respondent puts 10 (or 0) beans, prompt:**<br>***"Are you sure that this event will almost certainly (not) happen?"***<br><br>**CIRCLE 1 in column P if you prompted the respondent, and report the final answer only.**			
Q633	***A baby in your community will die before his or her first birthday?***	# beans on plate<br>[ ]	prompt (0 or 10)<br>1	
Q634	***You will go to the market at least once within the next 2 days?***<br><br>**LEAVE BEANS IN PLATE.**	[ ]	1	
Q635	***You will go to the market at least once within the next 2 weeks?***	[ ]	1	- **Q637 if beans added**
Q636	***Remember, as time goes by, you may find more time to go to the market. Therefore, you should have added beans to the plate. Let me ask you again. Now, add beans in the plate so that the number of beans in the plate reflects how likely you think it is that you will go to the market at least once within the next 2 weeks?***	[ ]	1	
Q637	***Please pick the number of beans that reflects how likely you think it is that …***<br><br>***You are infected with HIV now?***	# beans on plate<br>[ ]		- **Q640 if unmarried**
Q638	***Your spouse is infected with HIV now?***	[ ]		
Q639	***You will use a condom next time you have sex with your spouse / long-term partner?***<br><br>**If no regular sexual partner(s), write '99'.**	[ ]		
Q640	***You will use a condom next time you have sex with a non-regular partner?***<br><br>**If no non-regular sexual partner(s), write '99'.**	[ ]		- **Q701 if '99'**
Q641	***Your current / most recent non-regular sexual partner is infected with HIV now?***<br><br>**If no non-regular sexual partner(s), write '99'.**	[ ]		
Q642	***You will use a condom next time you have sex with a non-regular partner?***<br><br>**If no non-regular sexual partner(s), write '99'.**	[ ]		
Q643	***You will use a condom next time you have sex with someone for the first time?***	[ ]		

Davis K, *et al. BMJ Open* 2023; 13:e067327. doi: 10.1136/bmjopen-2022-067327

INDIVIDUAL QUESTIONNAIRE: **HEALTH & ACCESS TO TREATMENT** ***Q. No:***

REF.	QUESTIONS & FILTERS	CODING CATEGORIES		SKIP TO
Q701	*Has a medical doctor or nurse ever diagnosed you with the following conditions?*		Yes / No / DK / Year / Tx / Pd	
		High blood pressure	1 / 2 / 98	
		Diabetes	1 / 2 / 98	
		High cholesterol	1 / 2 / 98	
		Kidney diseases	1 / 2 / 98	
		Heart diseases (incl. stroke)	1 / 2 / 98	
		Depression	1 / 2 / 98	
		Osteoporosis	1 / 2 / 98	
		Tuberculosis	1 / 2 / 98	
		Malaria	1 / 2 / 98	
		Schistosomiasis	1 / 2 / 98	
		Hepatitis B	1 / 2 / 98	
		Hepatitis C	1 / 2 / 98	
		Pregnancy/delivery complications	1 / 2 / 98	
		Human papilloma virus	1 / 2 / 98	
		Cancer (specify type)	1 / 2 / 98	
		Genital herpes	1 / 2 / 98	
		HIV infection	1 / 2 / 98	
Q702	For EACH condition mentioned in Q701, record the year of first diagnosis in the 4th column above.			
Q703	For EACH condition mentioned in Q701, record details of the institution providing the treatment in the 5th column (Tx) using the following codes:	Public sector healthcare provider	1	
		Church Mission healthcare provider	2	
		Private sector provider	3	
		NGO or academic research institution	4	
		Other	8	
		Not currently receiving treatment	99	
Q704	For EACH condition mentioned in Q701, record details of who paid for the medical fees and the costs of the drugs in the 6th column (Pd) using the following codes:	Private medical insurance only	1	
		Private medical insurance partly	2	
		Respondent and/or family	3	
		Other	8	
		Service was free	99	
Q705	*How long is it since you last experienced an illness?*	Days/weeks	days / weeks	
		More than one year ago	97	- Q714
Q706	*What was the main symptom of the illness?*	Fever - malaria (incl. cerebral)	1	
		Fever - non-malaria	2	
		Sickness/vomiting	3	
		Diarrhoea/weight loss	4	
		Swollen lymph nodes	5	
		Skin complaints/rashes	6	
		Genital conditions: incl. STDs	7	
		Flu/pneumonia	8	
		Accident/wound	9	
		Tuberculosis	10	
		Other (specify)	12	
Q707	*How long was it between the time when you first noticed symptoms and when you first sought help for this illness?*	Days and weeks	days / weeks	- Q709
		Did not seek assistance	97	
Q708	*What is the main reason you have not sought treatment?*	Costs of treatment/travel too high	1	- Q714
		Too little time to travel / wait for treatment	2	- Q714
		Do not feel that anyone can help	3	- Q714
		Able to cope myself / illness not serious	4	- Q714
		Fear of stigma	5	- Q714
		Other (specify)	8	- Q714
Q709	*Which of the following people did you visit or consult for this illness?* Read the options and tick all that apply.		Yes / No	
		Medical doctor or nurse	1 / 2	- Q711
		N'anga	1 / 2	- if not
		Faith healer	1 / 2	- n'anga or
		Other (specify)	1 / 2	- faith hlr
Q710	*Did you seek assistance from the n'anga (or faith healer) for any of these reasons?* Read the options and tick all that apply.		Yes / No	
		Clinic treatment not effective	1 / 2	- Q714
		Spiritual cure needed	1 / 2	- if did
		Witchcraft suspected	1 / 2	- not also
		Holy water/bath or prayer	1 / 2	- visit
		More confidential than clinic	1 / 2	- hospital
		More convenient to visit than clinic	1 / 2	- or
		Less expensive to visit than clinic	1 / 2	- clinic
		Other (specify)	1 / 2	-
Q711	*How long did it take you to travel from your home to the clinic/hospital?*	Minutes<br>Convert hours to minutes if necessary.	mins	
Q712	*How long did you have to wait before you were seen by a doctor or nurse?*	Minutes<br>Convert hours to minutes if necessary.	mins	

INDIVIDUAL QUESTIONNAIRE: **HEALTH & ACCESS TO TREATMENT** ***Q. No:*** [ ]

REF.	QUESTIONS & FILTERS	CODING CATEGORIES		SKIP TO
**Q713**	***Did you receive an HIV test as part of your treatment for this illness?***	**Yes**<br>**No or don't know**	1<br>2	
**Q714**	**For men:**<br>***Some men experience white, transparent or yellowish discharge from the penis, which might not cause discomfort or may be accompanied by mild burning paid on urination. In the last 12 months, have you had these symptoms?***<br>**For women:**<br>***Some women experience an unusual discharge from the vagina or pain in the lower stomach. In the last 12 months, have you had these symptoms?***	**Yes - discharge & pain (both)**<br>**Yes - discharge only**<br>**Yes - pain only**<br>**No**<br>**Don't know**<br>**Clarify which & tick boxes as appropriate.**	1<br>2<br>3<br>4<br>98	
**Q715**	***Some (wo)men experience sores in the genital area. During the last 12 months, have you noticed any such sores?***	**Yes**<br>**No**<br>**Don't know**	1<br>2<br>98	**- Q717**<br>**- if not had**<br>**- symptoms**
**Q716**	***What happened when you were treated at the health centre for these symptoms?***<br>**Read the options and tick all that apply.**	**Not applicable - did not get treated at a clinic**<br>**Physical symptoms were inspected**<br>**Samples taken & tests done**<br>**HIV test was offered**<br>**Counseling provided**<br>**Free condoms provided**<br>**Symptoms went away after treatment**	Yes / No<br>99<br>1 / 2<br>1 / 2<br>1 / 2<br>1 / 2<br>1 / 2<br>1 / 2	
**Q717**	***On how many different occasions have you had an HIV test and received the results: (i) in your lifetime; and (ii) in the last 3 years?***<br>**Stress that these questions do *not* include self-tests.**	**Lifetime**<br>**Last 3 years**		**- If "0" go to Q725**
**Q718**	***How long is it since you last had an HIV test?***	**Period**	mths / yrs	
**Q719**	***Did you receive counselling before you agreed to have the test?***<br>**Explain what is meant by counselling.**	**Yes**<br>**No**	1<br>2	
**Q720**	***Was the result of this HIV test positive?***<br>**Stress that do not have to answer these questions but information is confidential.**	**Yes**<br>**No**<br>**Don't know**<br>**Prefers not to say**	1<br>2<br>98<br>99	
**Q721**	***After the test, did you receive counselling and referrals for the following HIV prevention methods?***<br>**Read the options and tick all that apply.**<br>**Don't ask about VMMC or PrEP if respondent is HIV-positive.**	**VMMC**<br>**PrEP**<br>**Male condoms**<br>**Female condoms**<br>**Being faithful to one partner**<br>**Not having sex**<br>**Treatment as prevention**	Counselling (Yes / No) — Referrals (Yes / No)<br>1 / 2 — 1 / 2<br>1 / 2 — 1 / 2<br>1 / 2 — 1 / 2<br>1 / 2 — 1 / 2<br>1 / 2 — 1 / 2<br>1 / 2 — 1 / 2<br>1 / 2 — 1 / 2	
**Q722**	***What is the name of the organisation that provided this HIV testing service?***	**PSI - New Start**<br>**FACT**<br>**FHI360**<br>**Hospital/clinic (ANC/PMTCT)**<br>**Hospital/clinic (other)**<br>**Other (specify)**<br>**Don't know**	1<br>2<br>3<br>4<br>5<br>8<br>98	
**Q723**	***After the HIV test, did you:***<br>**(1) Use condoms more or less than before?**<br>**(2) Start having more or fewer sexual partners?**		More / Same / Less<br>1 / 2 / 3<br>1 / 2 / 3	
**Q724**	***Did you join a post-test club or a group for people living with HIV and, if so, are you still a member?***<br>**If HIV test result was +ve, go to Q737.**	**Post-test club**<br>**PLWHA**<br>**Neither**	Still / Was / Never<br>1 / 2 / 3<br>1 / 2 / 3<br>8	
**Q725**	***Please tell me whether you strongly disagree, disagree, neither agree nor disagree, agree or strongly agree with the following statements …***<br>**(1) I am confident I can get tested for HIV if I wanted to**<br>**(2) I am confident I can get tested for HIV even if the healthworker cannot keep the results confidential**<br>**(3) I am confident I can get tested for HIV even if my partner disapproves**<br>**(4) I am confident I can get tested for HIV even if I am afraid to be HIV+**<br>**(5) I am confident I can get tested for HIV even if I was stigmatised in my community if I was known or suspected to be HIV+**		SD / D / N / A / SA<br>1 / 2 / 3 / 4 / 5<br>1 / 2 / 3 / 4 / 5<br>1 / 2 / 3 / 4 / 5<br>1 / 2 / 3 / 4 / 5<br>1 / 2 / 3 / 4 / 5	

INDIVIDUAL QUESTIONNAIRE: **HEALTH & ACCESS TO TREATMENT** *Q. No:*

REF.	QUESTIONS & FILTERS	CODING CATEGORIES		SKIP TO
Q726	*Are you able to discuss HIV testing with your partner?*	Yes - already done so	1	
		Yes - not done already but can do this	2	
		No	3	
		No current partner	99	
Q727	*Would your partner disapprove if you got tested for HIV?*	Yes	1	
		No	2	
		Don't know	98	
Q728	*Would your partner come with you to get tested for HIV?*	Yes - already done so	1	
		Yes - not done already but can do this	2	
		No	3	
		Don't know	98	
Q729	*If / when you want to have an(other) HIV test, do you know a place where someone like you can easily get it?*	Yes	1	
		No	2	
Q730	*If / when you want to have an(other) HIV test yourself, how easy is it for you to access the service?*	Very easy	1	
		Easy	2	
		Neither easy nor difficult	3	
		Difficult	4	
	Ask for an answer on a scale of 5 - from 1 (very easy) to 5 (very difficult).	Very difficult	5	
		Don't know	98	
Q731	*What factors make it difficult or unsuitable for someone like you to get access to HIV testing services?*		Spont / Probed	
		High costs		
		Judgemental staff		
		Lack of privacy or confidentiality		
	Ask first without probing and then probe for factors that are not mentioned spontaneously.	Limited opening hours		
		Distance / travel difficulties		
		Not appropriate for me to go there		
		Other (specify)		
Q732	*How far away is it from where you live to the nearest place where HIV tests are provided?*	Distance in kms	kms	
Q733	*Do you want to have an HIV test if it was freely accessible to you?*	Yes	1	
		No	2	
		Not sure	8	
Q734	*How definitely do you want to have an HIV test if it was freely accessible to you?*	Definitely	1	
		Probably	2	
		No opinion	3	
	Ask for an answer on a scale of 5 - from 1 (definitely) to 5 (definitely not).	Probably not	4	
		Definitely not	5	
Q735	*Do you plan to have an(other) HIV test?*	Yes	1	
		No	2	- Q737
		Don't know	98	- Q737
Q736	*How soon do you plan to have an(other) HIV test?*	Number of months	mths	
Q737	*What are the main reasons that woud/did motivate you to go for an HIV test?*		Spont / Probed	
		To access HIV treatment if I need it		
		Wanted re-assurance not infected		
		Avoid infecting partner(s)		
		Partner or child sick or died		
		Past risky behaviour		
		Partner's risky behaviour		
	Ask first without probing and then probe for factors that are not mentioned spontaneously.	Many friends going for HIV tests		
		Encouraged by family		
		Reassure partner		
		Contemplating marriage		
		Contemplating having a child		
		Prevent mother-to-child infection		
		Spouse/partner tested HIV+		
		Doctor/nurse suggested it		
		Life planning		
		Other (specify)		
Q738	*What are the main reasons that would/did discourage you from having an HIV test?*		Spont / Probed	
		Psychological effects		
		Stigma & discrimination		
		Possible divorce/separation		
		Job loss		
	Ask first without probing and then probe for factors that are not mentioned spontaneously.	Positive result accelerates death		
		Fear - of being HIV+		
		Fear - of partner violence		
		Lack of confidentiality		
		Too expensive		
		Other (specify)		

INDIVIDUAL QUESTIONNAIRE: **HEALTH & ACCESS TO TREATMENT** ***Q. No:***

REF.	QUESTIONS & FILTERS	CODING CATEGORIES		SKIP TO
**Q739**	***If you have or had a daughter or son who is a teenager or a young adult and she/he started having sex, do you think it would be a good thing for her/him to have an HIV test?***		Teen / YA	
		**Yes**	1 / 1	
		**No**	2 / 2	
		**Don't know**	98 / 98	
**Q740**	Explain that there are HIV tests that you can do yourself.	**Period**	mths / yrs	
	How long is it since you last had an HIV self-test?	**Never had a self-test**	97	- Q743
		**Not heard of HIV self-tests**	99	- Q744
**Q741**	***Was the result of this HIV self-test positive or negative?***	**Positive**	1	
		**Negative**	2	
	Stress that do not have to answer these questions but information is confidential.	**Don't know**	98	
		**Prefers not to say**	99	
**Q742**	***Did you get this result confirmed at a health facility or other HIV testing facility?***	**Yes - result confirmed**	1	
		**No - went but result was different**	2	
	Stress that do not have to answer these questions but information is confidential.	**No - didn't go for confirmation test**	3	
		**Prefers not to say**	99	
**Q743**	***If / when you want to do an HIV self-test, do you know a place where someone like you can easily get a test kit?***	**Yes**	1	
		**No**	2	
**Q744**	***Have you heard of antiretroviral therapy (ART) - the drugs that prevent HIV from causing AIDS?***	**Yes**	1	
		**No**	2	- Q748
**Q745**	***How far is it from here to the nearest place where these drugs can be obtained?***	**Distance**	kms	
		**Don't know a place**	98	- Q748
**Q746**	***What type of place is this?***	**Large town or city**	1	
		**Small town**	2	
	"Roadside" here means a tarred road.	**Growth point**	3	
		**Commercial estate/mine**	4	
	Record name of place..	**Roadside business centre**	5	
		**Rural business centre**	6	
		**Communal / resettlement area**	7	
**Q747**	***How long does it take to travel from your home to this place?*** Convert hours to minutes if necessary.	**Minutes**	mins	
**Q748**	***Have you ever taken ART yourself?***	**Yes**	1	- Q750
	Check that not taking something that could be ART.	**No**	2	
**Q749**	***What is the main reason you have not started taking these drugs?***	**Costs too high**	1	- Q801
		**Not available locally**	2	- Q801
		**Not permitted by church**	3	- Q801
		**Side effects**	4	- Q801
		**Not needed: in good health**	5	- Q801
		**Not needed: HIV-**	6	- Q801
		**Other (specify)**	8	- Q801
		**Don't know**	98	- Q801
**Q750**	***How long is it since you first took these drugs?***	**Period**	weeks / years	
**Q751**	***What motivated you to start taking ART?***	**HIV+: unwell**	1	
		**HIV+: well but wanted to remain healthy**	2	
		**Other (specify)**	8	
**Q752**	***Have you stopped taking the drugs?***	**Yes**	1	
		**No**	2	- Q754
**Q753**	***Why have you stopped taking the drugs?***	**Costs too high**	1	- Q756
		**Not available locally**	2	- Q756
		**Side effects**	3	- Q756
		**Not needed: in good health**	4	- Q756
		**Other (specify)**	8	- Q756
		**Don't know**	98	- Q756
**Q754**	***Are there particular times when you take the drugs?***	**All the time**	1	
		**When feeling unwell**	2	
		**When can afford or paid for**	3	
		**Other (specify)**	8	
**Q755**	***Do you sometimes forget to take the drugs?***	**Never**	1	
		**Occasionally**	2	
		**Quite often**	3	
**Q756**	***How often have you taken ART in the last month?***	**Every day**	1	
		**Most days**	2	
		**Occasionally**	3	
		**Not at all**	4	

INDIVIDUAL QUESTIONNAIRE:	HEALTH & ACCESS TO TREATMENT	*Q. No:*

REF.	QUESTIONS & FILTERS	CODING CATEGORIES	SKIP TO
Q757	*After you started taking the drugs, did you:*<br>(1) Start having more or fewer sexual partners?<br>(2) Use condoms more or less than before?	More / Same / Less<br>1 / 2 / 3<br>1 / 2 / 3	
Q758	*Have you experienced any unpleasant side effects since you started the treatment?*	Yes 1<br>No 2	
Q759	*Have you (also) been receiving treatment from a traditional healer or faith healer for your HIV infection?*	Traditional healer 1<br>Faith healer 2<br>Neither 3	

INDIVIDUAL QUESTIONNAIRE: **FERTILITY HISTORIES** *Q. No:*

REF.	QUESTIONS & FILTERS	CODING CATEGORIES		SKIP TO
Q801	*Now I would like to talk to you about pregnancy and childbirth. Have you ever given birth (fathered a child)*?*	Yes<br>No	1<br>2	 - Q806
Q802	*Do you have any sons or daughters who are living with you now?*	Yes<br>No	1<br>2	 - Q804
Q803	*How many sons live with you? How many daughters live with you?*	Sons at home<br>Daughters at home		
Q804	*Do you have any sons or daughters who are alive but do not live with you?*	Yes<br>No	1<br>2	 - Q806
Q805	*How many sons are alive but do not live with you? How many daughters are alive but do not live with you?*	Sons elsewhere<br>Daughters elsewhere		
Q806	*Have you ever given birth to (or fathered) a boy or girl who was born alive but later died?*<br>If no, probe:<br>*Any (other) boy or girl who cried or showed any sign of life but only survived a few hours or days?*	Yes<br>No	1<br>2	 - Q808
Q807	*How many boys have died in this way? And how many girls died in this way?*	Boys who died<br>Girls who died		
Q808	Sum answers to Q803, Q805 and Q807. Enter total.	Total		
Q809	*In total, then, how many live births have you had (fathered)?*<br>Compare response with total in Q708.<br>If numbers are different, probe and correct Q801-Q808, as necessary.			- for men, go to Q901
Q810	*Have you had a live birth in the last year?*	Yes<br>No	1<br>2	
Q811	*Have you been pregnant in the last year?*	Yes<br>No	1<br>2	

***Note:** **For male respondents use question wordings indicated in brackets.**

INDIVIDUAL QUESTIONNAIRE: **RECENT PREGNANCY HISTORY** *Q. No:*

REF.	QUESTIONS & FILTERS	CODING CATEGORIES		SKIP TO
**Q901**	***Are you pregnant at the moment?***	**Yes**<br>**No**	1<br>2	 **- Q903**
**Q902**	***How many months pregnant do you think you are?***<br>**Ask for best estimate.**	**Number of months**		**- Q905**
**Q903**	***Have you ever had a pregnancy that ended in a live birth, miscarriage or still-birth?***	**Yes**<br>**No**<br>**Not sure**	1<br>2<br>98	 **- Q1001**<br>**- Q1001**
**Q904**	***When did your most recent such pregnancy end?***<br>**Stress: including miscarriages.**		mnth yr	
**Q905**	***At the time you became pregnant, did you want to become pregnant then, did you want to wait until later, or did you not want to become pregnant at all?***	**Then**<br>**Later**<br>**Not at all**	1<br>2<br>3	
**Q906**	***Did you see anyone for antenatal care during this pregnancy?***	**Yes**<br>**No**	1<br>2	 **- Q923**
**Q907**	***After how many months of the pregnancy did you first go for an antenatal check-up?***	**Months**		
**Q908**	***Did you have an HIV test while attending for ANC check-ups for this pregnancy or did you already know your status?***<br>**If knew HIV+, ask if she was on ART.**	**Yes**<br>**No: already knew HIV+ - on ART**<br>**No: already knew HIV+ - not on ART**<br>**No: did not want**	1<br>2<br>3<br>4	
**Q909**	***Did your husband (or pregnancy partner) also attend for PMTCT with you and have an HIV test during this pregnancy?***	**Yes**<br>**No: already knew HIV+**<br>**No: did not want**	1<br>2<br>3	
**Q910**	***Did YOU have HIV infection at the time of this pregnancy?***<br>**Stress confidential but voluntary.**	**Yes**<br>**No**<br>**Don't know**<br>**No response**	1<br>2<br>8<br>9	 **- Q917**<br>**- Q917**<br>**- Q917**
**Q911**	***Did the clinic provide you with any counselling on family planning for people with HIV?***	**Yes**<br>**No**<br>**Don't recall**	1<br>2<br>8	
**Q912**	***Did you receive any treatment to take YOURSELF to prevent the baby from getting infected? If so, what type?***<br>**Stress that this is treatment taken by the mother herself.**	**Yes: already on ART**<br>**Yes: initiated on ART during pregnancy**<br>**Yes: other (specify)** ____________<br>**No**	1<br>2<br>3<br>4	**- Q915**<br><br>**- Q915**<br>**- Q915**
**Q913**	***Are you still taking ART now?***	**Yes**<br>**No**	1<br>2	**- Q915**
**Q914**	***Why did you decide to stop taking ART?***<br>____________	**No longer breastfeeding the baby**<br>**Healthy so no need for ART**<br>**Side effects**<br>**Other (specify)**<br>**Don't know**	1<br>2<br>3<br><br>98	

INDIVIDUAL QUESTIONNAIRE: **RECENT PREGNANCY HISTORY** ***Q. No:***

REF.	QUESTIONS & FILTERS	CODING CATEGORIES		SKIP TO
**Q915**	***Did the baby receive any of these forms of treatment to prevent him/her from getting infected with HIV?***		Y N	
		**ART (i.e. continuous)**	1 2	
		**NVP syrup**	1 2	
		**Other (specify)**	1 2	
**Q916**	***Did you or the baby ever stop or miss taking the treatment you were given?***	**Yes**	1	
		**No**	2	
		**Baby died before completed treatment**	3	
**Q917**	***Was the baby delivered at a clinic/hospital or at home?***	**Clinic**	1	
		**Home**	2	
**Q918**	***Did this pregnancy end in a miscarriage, abortion or stillbirth?***	**Yes**	1	
		**No**	2	**- Q920**
**Q919**	***How many months pregnant were you when this pregnancy ended?***		mths	
**Q920**	***Did the baby ever have an HIV test?***	**Yes: infected**	1	
		**Yes: uninfected**	2	
	**If yes, ask for result.**	**No**	3	
	**Stress confidential but voluntary.**	**DK or rather not say**	8	
**Q921**	***Has the baby been initiated on ART?***	**Yes: started & still taking**	1	
		**Yes: started but since stopped**	2	
		**No**	3	
		**Don't know**	8	
**Q922**	***Has the baby received medical male circumcision?***	**Yes: medical circumcision**	1	**- Q927**
		**No: but traditional circumcision**	2	
		**No: not circumcised at all (yet)**	3	
		**No: baby is a girl**	99	**- Q927**
**Q923**	***Would you have liked the baby to have received medical male circumcision?***	**Yes**	1	
		**No**	2	
**Q924**	***Were medical male circumcision services for infants available in your area at the time this baby was born?***	**Yes**	1	
		**No**	2	
**Q925**	***Is the baby still alive?***	**Yes**	1	**- Q929**
		**No**	2	
**Q926**	***How old was the baby when he/she passed away?***	**Convert to months.**	mths	
		**<1m = '0' months.**		
**Q927**	***Did you ever feed this baby at the breast?***	**Yes**	1	
		**No**	2	**- Q1001**
**Q928**	***Are you still breastfeeding?***	**Yes**	1	**- Q1001**
		**No**	2	
		**Child has died**	99	
**Q929**	***For how long did you breastfeed this baby?***			
	**Exclusively? (i.e. no liquids or solids)**	**Months**		
	**In total?**	**Months**		
	**If total > 6 mths, go to Q1001.**			
**Q930**	***Why did you not breastfeed this baby (for longer)?***	**Baby sick or died**	1	
		**Mother sick**	2	
		**Risk of HIV**	3	
		**Pregnant**	4	
		**Resumed sex**	5	
		**Other**	8	

INDIVIDUAL QUESTIONNAIRE: **CONCLUSION** ***Q. No:***

REF.	QUESTIONS & FILTERS			
**Q1001**	***For how much of the last 3 years have you and your regular partner been using a method of contraception?***	**None**<br>**Some of the time**<br>**Most/all of the time**<br>**Not sure**	1<br>2<br>3<br>98	**- Q1004**
**Q1002**	***Which of these methods were the main methods you used?***<br><br>Ask about each method in turn.	**Pill**<br>**Injections**<br>**Condoms**<br>**Femidoms**<br>**Sterilization**<br>**Safe period**<br>**Withdrawal**<br>**Other (specify)**	Y N<br>1 2<br>1 2<br>1 2<br>1 2<br>1 2<br>1 2<br>1 2<br>1 2	
**Q1003**	***What were your main reasons for wanting to delay or prevent another pregnancy?***<br><br>Probe for other reasons, but do not prompt.	**Enough children**<br>**Birth spacing**<br>**Child HIV+ risk**<br>**Child orphan risk**<br>**Mother HIV+: accelerate AIDS**<br>**Not yet ready to have children**<br>**Other (specify)**	1<br>2<br>3<br>4<br>5<br>6<br>8	
**Q1004**	Record current time.	**Hour and minutes**	hr mins	
**Q1005**	Record contact details for follow-up interviews if required.	**Cell number (1)**<br>**Cell number (2)**		
**Q1006**	***What are your views of the value of this research?***	**Useful**<br>**Do not see the point**<br>**No opinion**	1<br>2<br>98	
**Q1007**	Record respondent's comments and your own observations in the space below.<br>Remind respondent of arrangements for the BE procedures (where relevant) and for HIV testing.			

RESPONDENT'S COMMENTS:

On the research?

Further HIV prevention, care and support activities needed?

ENUMERATOR'S OBSERVATIONS:
