# Supplementary material for: Associations between HIV status and self-reported hypertension in a high HIV prevalence sub-Saharan African population: a cross-sectional study
Source: BMJ Open. 2023 Jan 12;13(1):e067327. doi: 10.1136/bmjopen-2022-067327 (PMC9843216; doi:10.1136/bmjopen-2022-067327)
Supplement: Author's manuscript [file bmjopen-2022-067327.draft_revisions.pdf]

BMJ Open is committed to open peer review. As part of this commitment we make the peer review history of every article we publish publicly available.

When an article is published we post the peer reviewers' comments and the authors' responses online. We also post the versions of the paper that were used during peer review. These are the versions that the peer review comments apply to.

The versions of the paper that follow are the versions that were submitted during the peer review process. They are not the versions of record or the final published versions. They should not be cited or distributed as the published version of this manuscript.

BMJ Open is an open access journal and the full, final, typeset and author-corrected version of record of the manuscript is available on our site with no access controls, subscription charges or pay-per-view fees (http://bmjopen.bmj.com).

If you have any questions on BMJ Open's open peer review process please email info.bmjopen@bmj.com

BMJ Open

Associations between HIV status and self-reported hypertension in a high HIV prevalence sub-Saharan African population: A cross-sectional study

Journal:	*BMJ Open*
Manuscript ID	bmjopen-2022-067327
Article Type:	Original research
Date Submitted by the Author:	09-Aug-2022
Complete List of Authors:	Davis, Katherine ; Imperial College London, MRC Centre for Global Infectious Disease Analysis, Department of Infectious Disease Epidemiology<br>Moorhouse, Louisa ; Imperial College London, MRC Centre for Global Infectious Disease Analysis, Department of Infectious Disease Epidemiology<br>Maswera, Rufurwokuda; Biomedical Research and Training Institute<br>Mandizvidza, Phyllis; Biomedical Research and Training Institute<br>DADIRAI, Tawanda; Biomedical Research and Training Institute<br>Museka, Tafadzwa; Biomedical Research and Training Institute<br>Nyamukapa, Constance; Imperial College London, MRC Centre for Global Infectious Disease Analysis, Department of Infectious Disease Epidemiology; Biomedical Research and Training Institute<br>Smit, Mikaela ; Imperial College London<br>Gregson, Simon; Imperial College London, MRC Centre for Global Infectious Disease Analysis, Department of Infectious Disease Epidemiology
Keywords:	Hypertension < CARDIOLOGY, HIV & AIDS < INFECTIOUS DISEASES, EPIDEMIOLOGY, PUBLIC HEALTH

SCHOLARONE™
Manuscripts

I, the Submitting Author has the right to grant and does grant on behalf of all authors of the Work (as defined in the below author licence), an exclusive licence and/or a non-exclusive licence for contributions from authors who are: i) UK Crown employees; ii) where BMJ has agreed a CC-BY licence shall apply, and/or iii) in accordance with the terms applicable for US Federal Government officers or employees acting as part of their official duties; on a worldwide, perpetual, irrevocable, royalty-free basis to BMJ Publishing Group Ltd ("BMJ") its licensees and where the relevant Journal is co-owned by BMJ to the co-owners of the Journal, to publish the Work in this journal and any other BMJ products and to exploit all rights, as set out in our licence.

The Submitting Author accepts and understands that any supply made under these terms is made by BMJ to the Submitting Author unless you are acting as an employee on behalf of your employer or a postgraduate student of an affiliated institution which is paying any applicable article publishing charge ("APC") for Open Access articles. Where the Submitting Author wishes to make the Work available on an Open Access basis (and intends to pay the relevant APC), the terms of reuse of such Open Access shall be governed by a Creative Commons licence – details of these licences and which Creative Commons licence will apply to this Work are set out in our licence referred to above.

Other than as permitted in any relevant BMJ Author's Self Archiving Policies, I confirm this Work has not been accepted for publication elsewhere, is not being considered for publication elsewhere and does not duplicate material already published. I confirm all authors consent to publication of this Work and authorise the granting of this licence.

For peer review only

Associations between HIV status and self-reported hypertension in a high HIV prevalence sub-Saharan African population: A cross-sectional study

For peer review only

Katherine DAVIS, MRes[1], Louisa MOORHOUSE, MSc[1], Rufurwokuda MASWERA, BSw[2], Phyllis MANDIZVIDZA, SRN[2], Tawanda DADIRAI, BSc[2], Tafadzwa MUSEKA, MSc[2], Constance NYAMUKAPA, PhD[1,2], Mikaela SMIT, PhD[1], Simon GREGSON, DPhil[1,2]

1. MRC Centre for Global Infectious Disease Analysis, Department of Infectious Disease Epidemiology, Imperial College London, London, UK

2. Biomedical Research and Training Institute, Harare, Zimbabwe

Corresponding author/contact for reprints: Katherine Davis, Department of Infectious Disease Epidemiology, St Mary's Campus, Imperial College London, London, W2 1PG, UK, K.davis18@imperial.ac.uk, +44 (0)20 7594 5777.

Word count: 3,573/4,000

For peer review only

Abstract

Objectives: This study examined whether HIV status and antiretroviral therapy (ART)-exposure were associated with self-reported hypertension in Zimbabwe.

Design: Study data were taken from a cross-sectional, general population survey, which included HIV testing (July 2018 – December 2019).

Setting: The data were collected in Manicaland Province, Zimbabwe.

Participants: 9,780 people aged 15 years and above were included.

Outcome measure: Self-reported hypertension was the outcome measure. This was defined as reporting a previous diagnosis of hypertension by a doctor or nurse. After weighting of survey responses by age and gender using household census data, chi-squared tests and logistic regression were used to explore whether HIV status and ART-exposure were associated with self-reported hypertension.

Results: The weighted prevalence of self-reported hypertension was 13.6% (95% Confidence Interval: 12.9%-14.2%) and the weighted prevalence of HIV was 11.1% (10.4%-11.7%). In univariable analyses, there was no evidence of a difference in the weighted prevalence of self-reported hypertension between people living with HIV (PLHIV) and HIV-negative people (14.1%, 11.9%-16.3% vs 13.3%, 12.6%-14.0%; p=0.503) or between ART-exposed and ART-naive PLHIV (14.8%, 12.0%-17.7% vs 12.8%, 9.1%-16.4%, p=0.388). Adjusting for socio-demographic variables in logistic regression did not alter this finding (odds ratios: HIV status: 0.87, 0.69-1.09, p=0.234; ART-exposure: 0.83, 0.53-1.30, p=0.411).

Conclusions: Approximately one in seven PLHIV self-reported having hypertension, highlighting an important burden of disease. However, no associations were found between HIV status or ART-exposure and self-reported hypertension, suggesting that it will be valuable to focus on managing other risk factors for hypertension in this population. These findings should be fully accounted for as Zimbabwe re-orients its health system towards non-communicable disease control and management.

Keywords: HIV & AIDS, hypertension, epidemiology, public health

For peer review only

Strengths and limitations

- This is the first study to compare the prevalence of self-reported hypertension between people living with HIV and HIV-negative people in a non-clinical setting in Zimbabwe.
- We used data from a large population survey, which included a diverse range of men and women, and could be weighted to local census information, increasing the representativeness of our results.
- We focused on self-reported hypertension, so hypertensive people who were unaware of their status were not included in our prevalence estimates.
- Our study used cross-sectional data that excluded individuals in hospital settings and did not capture data on all risk factors for hypertension.

For peer review only

Introduction

Hypertension is the leading modifiable cause of death and disability worldwide.[1] In sub-Saharan Africa, the region most affected by infectious diseases such as HIV, the burden of hypertension is growing rapidly.[2] Yet despite these newly coinciding burdens, the relationship between HIV and hypertension is poorly understood.[3]

Globally, there is some evidence that people living with HIV (PLHIV) might face a higher burden of hypertension.[4,5] However, the relationship appears to vary by region and it has been suggested that, in African countries, PLHIV may experience a lower prevalence of hypertension.[6] For example, in a Ugandan population, PLHIV were found to have 30% lower odds of hypertension than matched HIV-negative controls.[7] Detailed information about how the burden of hypertension differs by HIV status remains scarce across sub-Saharan Africa, constraining understanding of the association between HIV and hypertension; this is of particular concern as the relationship may differ between populations.[6] In Zimbabwe, only three studies have examined the burden of hypertension among PLHIV in the community, and all lacked a comparison group of HIV-negative people.[8–10] Just one of these studies examined the effect of antiretroviral therapy (ART) on hypertension, reporting that long-term use of ART was associated with increased prevalence of hypertension, a finding which has not been confirmed in this setting.[8]

Bridging the data gap will be crucial to supporting strategic decision-making about provision of preventative services and hypertension treatment. Currently, capacity to prevent and control hypertension in Zimbabwe is limited, but major changes are being planned to manage the rising burden.[8,11–13] Delivering these changes efficiently will require an understanding of the role of HIV status and ART-exposure in hypertension. This study aimed to contribute to that understanding, by examining whether HIV status and ART-exposure were associated with self-reported hypertension in Manicaland, East Zimbabwe.

For peer review only

Methods

Data

Study data were taken from the Manicaland General Population Open Cohort Study (Manicaland Study), a long-running, large-scale HIV sero-survey.[14,15] Each survey round of the cohort has involved testing participants for HIV and asking questions on topics including participants' sociodemographic characteristics, general health, and knowledge of HIV.[15] The most recent round, which ran between July 2018 and December 2019, is unique as it provides the first insight into the burden of hypertension and other non-communicable diseases (NCDs). Consequently, cross-sectional data from the most recent round was used in this analysis (questionnaire in supplementary material).

Eight sites were included in the round: a subsistence farming area (Bonda Mission), a tea estate (Eastern Highlands), a forestry estate (Selbourne), two small towns (Nyazura and Nyanga), a roadside settlement (Watsomba) and two urban areas (Sakubva and Hobhouse). A team of Zimbabwean researchers led data collection at the sites, gathering survey responses on electronic tablets. Initially, households were enumerated in a census which included a brief questionnaire that asked about relationships between those in the household and household assets. After the census, more detailed data were collected through individual interviews. All women aged 15-24 years and all men aged 15-29 years, as well as a random sample of two thirds of older adults, in enumerated households were eligible to complete the individual interview. The research team contacted eligible participants and interviewed them at central sites, such as health centres, or in their homes. If eligible people were unavailable or couldn't be contacted when first approached, at least two further attempts were made to reach them. The team aimed to achieve a sample size of approximately 9,500, in order to detect changes in the primary outcome measures of the Manicaland Study, which will be reported elsewhere.[16–18]

During the individual interview, participants provided a range of socio-demographic and health information. For example, they self-reported whether they had ever been diagnosed with hypertension by a doctor or nurse and whether they had ever taken ART.

For peer review only

In addition to responding to interview questions, participants were offered provider-initiated testing and counselling (PITC) for HIV and asked to provide a dried blood spot (DBS) specimen for laboratory HIV testing. PITC was performed by accredited members of the research team following the standard procedures and testing algorithm used in Ministry of Health and Child Care routine PITC services. DBS specimens were tested using the same algorithm at the Biomedical Research and Training Institute (BRTI) in Harare.

Blood pressure was not measured during the household census or individual interview.

The Manicaland Study was conducted with the understanding and informed consent of participants. Ethical approval was given by Imperial College Research Ethics Committee (17IC4160), the BRTI Institutional Review Board (AP140/2017) and the Medical Research Council of Zimbabwe (MRCZ/A/2243).

Weighting

The selection criteria for the individual interview, and the pattern of response and non-response, generated a young cohort, with more women than men. As a result, weights were used to adjust variable distributions to better match the population in the household census. First, census data were analysed to calculate the proportion of the population in 5-year age groups between 15 and 74 years, as well as a 75+ year-old age-group, by gender. Iterative proportional fitting, also known as raking, was then used to generate weights that matched the age and gender proportions in the interview sample to those in the household census. Participant data were weighted for all further analyses; this approach meant that 42 people with missing age or gender information were excluded.

Association between HIV and self-reported hypertension

After weighting, prevalence estimates for self-reported hypertension and HIV were generated. Self-reported hypertension was defined as reporting ever having been diagnosed with hypertension by a doctor or nurse. HIV status was determined from PITC results; however, if a person opted out of PITC but consented to provide a DBS specimen, the DBS specimen was tested in the laboratory and

For peer review only

this result was used instead. Consequently, our sample of PLHIV included both people who knew their status at the time of interview and people who did not. Prevalence estimates were calculated as the weighted number of people living with HIV or self-reporting hypertension, divided by the weighted total population. To compare the prevalence of self-reported hypertension between PLHIV and HIV-negative people, estimates for the prevalence of self-reported hypertension stratified by HIV status, age and gender were produced in the same manner. Age was grouped into six classes: '15-24', '25-34', '35-44', '45-54', '55-64' '65 and over'. Categories were chosen to reflect the Zimbabwean population distribution, in which most people are young, but a minority have reached old age. Crude comparisons of the prevalence of self-reported hypertension between PLHIV and HIV-negative people were made using Chi-squared tests, with Rao-Scott adjustment. These were performed both for the overall population and within age and gender strata.

Logistic regression models were employed to examine the relationship between HIV and self-reported hypertension, while controlling for available sociodemographic variables. All models were complete case analyses. Initially, a model was built including HIV, age, and gender. This was then developed into a fully adjusted model. First, a range of potential sociodemographic confounders were identified through the literature. Second, any potential confounders that were found to be associated with self-reported hypertension in models including age and gender, at a threshold p-value of 0.1, were added to the initial model. Lastly, the new model was refined by backward elimination of potential confounders, with a threshold p-value of 0.05. This created a final model for generating adjusted estimates of the association between HIV status and self-reported hypertension.

The potential confounders that were considered for inclusion in the model were employment status, relationship status, education status, study site and wealth. Employment status was classified as 'employed or in education' or 'unemployed'; people who worked in the informal sector, doing petty trading or agricultural work, were categorised as 'employed or in education'. Relationship status was coded as 'married or in a long-term relationship', 'never been in a long-term relationship', 'separated or divorced' or 'widowed'. A long-term relationship was defined as a relationship of 12 months or more. Assessment of education status was based on the highest level of schooling that a person had obtained. Two categories were used: 'none or primary' and

For peer review only

'secondary or higher'; this reflected the fact that few people reported no schooling or education above secondary level. Wealth was classified using an index based on whether households owned a range of assets, as described by Lopman et al.[19] Assets included sellable items, such as bicycles and cattle, as well as fixed assets, such as water and electricity supplies.

The category representing the absence of a characteristic was chosen as the baseline for most variables. For variables where there was not an absent category, the category with the most participants, or that had been chosen as the baseline in other analyses of Manicaland Study data, was selected.[20,21]

Association between ART-exposure and self-reported hypertension

The association between ART-exposure in PLHIV and self-reported hypertension was evaluated using the approach to logistic regression described above. People who reported ever taking ART were defined as ART-exposed, allowing the prevalence of ART-exposure to be calculated as the weighted number of PLHIV who were ART-exposed, divided by the weighted total number of PLHIV. Prevalence estimates for self-reported hypertension, stratified by ART-exposure, age, and gender, were then produced and a crude comparison between ART-exposed and ART-naive PLHIV was made using Chi-squared tests, with Rao-Scott adjustment. Finally, logistic regression models were developed, using the same model building strategy as was applied for the HIV status analysis, to produce adjusted estimates.

Software

Analyses were performed in R 4.0.3 using the survey package. [22,23]

Patient and public involvement

Members of the local community provided input into the research through small meetings held with community members at the beginning of the Manicaland Study and through providing feedback at meetings that were held to disseminate results in successive rounds of the survey. Feedback included recommendations for improving the recruitment and conduct of the study, as well as for reducing the burden of participation. In addition, the team worked closely with local guides (mainly community health workers) who also provided feedback as the study progressed. Input into the

For peer review only

detail of the study design, research questions and outcome measures, was provided by local members of the research team too.

It is intended that the main results of this study will be disseminated to local stakeholders, research participants and other community members, as part of a wider plan to share results from the Manicaland Study. As in previous survey rounds, involvement of community members and local members of the research team will be key to the development of the dissemination strategy.

Results

During the census 7,610 households were contacted (Fig. 1). Of these, 7,550 households (99.2%) completed the questionnaire, making some of their members eligible for the individual interview. Among the 12,761 eligible people, 2,939 (23.0%) were unable to participate in, or did not consent to, the individual interview (reasons shown in Fig. 1). More of those who did not consent came from wealthier households (X^2=31.9, p<0.001) and urban sites (X^2=158.89, p<0.001). After exclusion of people without age or gender data, there were 9,780 participants in the study population.

Following weighting, there were marginally more women than men in the interviewed population (women: 52.2%; Table 1). The population was young; nearly a third were under 25 years old (31.0%). Unemployment was high (43.8%, 95% CI=42.9%-44.7%), yet most participants had secondary education or higher (75.7%, 74.9%-76.4%) and few lived in the poorest households (3.0%, 2.7%-3.4%).

For peer review only

Table 1. Characteristics of study participants.

	Total Population (N=9,780)[1]		People living with HIV (N=936)[1]		HIV-negative people (N=8,378)[1]	
	Weighted proportion, % (95% Confidence Interval)	N[1]	Weighted proportion, % (95% Confidence Interval)	N[1]	Weighted proportion, % (95% Confidence Interval)	N[1]
**Hypertension**						
Not reported	86.4% (85.7%-87.1%)	8,453	85.9% (83.7-88.1%)	791	86.7% (86.0-87.4%)	7,273
Reported	13.6% (12.9%-14.2%)	1,252	14.1% (11.9-16.3%)	139	13.3% (12.6-14.0%)	1,043
Missing	-	75	-	6	-	62
**HIV status**						
Negative	88.9% (88.3%-89.6%)	8,378	-	-	-	-
Positive	11.1% (10.4%-11.7%)	936	-	-	-	-
Missing	-	466	-	-	-	-
**ART-exposure[2]**						
ART-naïve	-	-	34.1% (31.0-37.2%)	323	-	-
ART-exposed	-	-	65.8% (62.8-69.0%)	612	-	-
Missing	-	-	-	1	-	-
**Gender[3]**						
Male	47.8%	4,064	43.6% (40.6%-46.7%)	331	48.2% (47.8%-48.7%)	3,544
Female	52.2%	5,716	56.4% (53.3%-59.4%)	605	51.8% (51.3%-52.2%)	4,834
**Age, years[3]**						
15-24	31.0%	3,923	7.1% (5.8%-8.5%)	97	34.7% (34.4%-35.1)	3,708
25-34	22.0%	1,988	19.8% (17.4%-22.2%)	191	22.2% (21.9%-22.6%)	1,689
35-44	18.5%	1,561	31.4% (28.6%-34.2%)	285	16.9% (16.5%-17.3%)	1,193
45-54	11.4%	1,022	27.1% (24.5%-29.7%)	253	9.4% (9.0%-9.7%)	704
55-64	7.2%	539	9.9% (7.8%-12.0%)	72	6.7% (6.4%-7.0%)	432
65+	9.8%	747	4.7% (3.3%-6.2%)	38	10.0% (9.7%-10.3%)	652
**Site**						
Bonda Mission (subsistence farming area)	15.9% (15.2%-16.7%)	1,497	14.4% (12.0%-16.8%)	129	16.7% (15.8%-17.5%)	1,343
Eastern Highlands (tea estate)	10.7% (10.0%-11.3%)	1,019	13.1% (10.9%-15.3%)	117	10.8% (10.1%-11.5%)	888
Selbourne (forestry estate)	14.4% (13.7%-15.1%)	1,377	15.6% (13.2%-18.0%)	145	14.8% (14.0%-15.6%)	1,210
Nyazura (town)	14.8% (14.1%-15.6%)	1,444	19.8% (17.1%-22.4%)	184	14.4% (13.7%-15.3%)	1,211
Nyanga (town)	11.4% (10.8%-12.0%)	1,157	6.8% (5.2%-8.4%)	69	10.0% (9.3%-10.6%)	866
Watsomba (roadside settlement)	17.4% (16.7%-18.2%)	1,694	16.1% (13.8%-18.5%)	154	18.1% (17.2%-18.9%)	1,496
Sakubva (urban)	7.8% (7.2%-8.3%)	789	8.6% (6.7%-10.4%)	82	7.5% (6.9%-8.1%)	654
Hobhouse (urban)	7.5% (7.0%-8.1%)	803	5.6% (4.2%-7.1%)	56	7.8% (7.2%-8.3%)	710

For peer review only - http://bmjopen.bmj.com/site/about/guidelines.xhtml

**Employment**						
Unemployed	43.8 (42.9%-44.7%)	4,322	47.6% (44.3%-50.8%)	472	43.4% (42.4%-44.4%)	4,722
Employed or in education	56.2 (55.3%-57.1%)	5,458	52.4% (49.2%-55.7%)	464	56.6% (55.6%-57.6%)	3,656
**Relationship status**						
Married or in a long-term relationship	59.5% (58.7%-60.3%)	5,389	60.4% (57.3%-63.6%)	536	59.4% (58.5%-60.3%)	4,594
Never been in a long-term relationship	25.9% (25.3%-26.5%)	3,033	10.2% (8.4%-12.1%)	111	28.3% (27.7%-29.0%)	2,821
Separated or divorced	6.8% (6.3%-7.4%)	663	13.2% (11.0%-15.3%)	131	5.8% (5.3%-6.4%)	483
Widowed	7.8% (7.3%-8.2%)	695	16.2% (13.8%-18.5%)	158	6.4% (5.9%-6.9%)	480
**Education**						
None or primary	22.9% (22.2%-23.7%)	2,023	27.1% (24.2%-30.1%)	244	22.4% (21.6%-23.2%)	1,685
Secondary or higher	77.1% (76.3%-77.8%)	7,595	72.9% (69.9%-75.8%)	663	77.6% (76.8%-78.4%)	6,566
Missing	-	162	-	29	-	127
**Asset-based wealth**						
1 (poorest)	3.0% (2.7%-3.4%)	287	3.7% (2.5%-5.0%)	35	3.1% (2.7%-3.5%)	251
2	38.6% (37.6%-39.6%)	3,679	41.5% (38.0%-44.8%)	388	39.2% (38.1%-40.3%)	3,193
3	29.0% (28.0%-29.9%)	2,798	30.8% (27.7%-33.9%)	283	29.0% (28.0%-30.0%)	2,401
4	26.8% (25.9%-27.7%)	2,680	22.2% (19.4%-24.9%)	209	26.2% (25.2%-27.2%)	2,246
5 (richest)	2.5% (2.2%-2.8%)	250	1.8% (0.9%-2.7%)	16	2.4% (2.1%-2.8%)	210
Missing	-	86	-	5	-	77

[1]Values are unweighted. In addition, HIV status was missing for some participants, so the unweighted Ns for People Living with HIV and HIV-negative people do not sum to the total population unweighted N.

[2]ART, antiretroviral therapy.

[3]Confidence intervals are not shown for variables used in weighting.

Association between HIV and self-reported hypertension

In this population, the weighted prevalence of self-reported hypertension was 13.6% (12.9%-14.2%) and the weighted prevalence of HIV was 11.1% (10.4%-11.7%). The prevalence of self-reported hypertension in PLHIV (14.1%, 11.9%-16.3%) was similar to the prevalence of self-reported hypertension in HIV-negative people in crude analyses (13.3%, 12.6%-14.0%, F=0.4, p=0.503). The lack of evidence for a difference remained when analysing the prevalence of self-reported hypertension by age and gender, as well as HIV status, for most groups (Fig. 2, Supplementary table 1). However, there was statistical support for lower prevalence of self-reported hypertension in PLHIV than in HIV-negative people among women aged 45-54 (PLHIV: 20.2%, 13.8%-26.7%; HIV-

For peer review only

negative: 30.1%, 25.7%-34.5%; F=5.4, p=0.021) and men aged over 65 (PLHIV: 6.7%, 0.0%-19.3%; HIV-negative: 31.2%, 25.3%-37.2%, F=4.1, p=0.043).

In initial logistic regression analyses adjusted for age and gender, no evidence was found for an association between HIV and self-reported hypertension (odds ratio, OR=0.87, 95% CI=0.70-1.07, p=0.191). Introducing further confounders (employment, relationship status, wealth, and site) to generate a fully adjusted model did not alter this finding (0.87, 0.69-1.09, p=0.234; Table 2).

By contrast, age and gender were strongly associated with self-reported hypertension, with odds of self-reporting hypertension substantially higher for the oldest people (aged 65 years and above) than the youngest (26.96, 19.84-36.64, p<0.001, versus ages 15-24 years) and for women than men (4.45, 3.69-5.38, p<0.001). In addition, employed people had 20% lower odds of self-reporting hypertension (0.80, 0.69-0.94, p=0.006), but the three wealthiest quintiles had higher odds of self-reporting hypertension than the poorest (Table 2). Relationship status was also associated with self-reporting hypertension; people who had never been in a long-term relationship or were widowed had lower odds of self-reporting hypertension than those who were married or in a long-term relationship (never had a relationship: 0.54, 0.4-0.72; widowed: 0.74, 0.58-0.94; p<0.001). Finally, self-reported hypertension varied with site, with urban participants having the highest odds of self-reporting hypertension relative to those from the subsistence farming area (Sakubva: 4.06, 2.97-5.54; Hobhouse: 1.98, 1.41-2.78; p<0.001).

For peer review only

Table 2. HIV as a determinant of hypertension.

	Weighted hypertension prevalence, % (95% Confidence Interval)	Odds ratio adjusted for only age and gender (95% confidence interval)	P-value	Fully adjusted odds ratio (95% confidence interval)	P-value
**HIV status**			0.191		0.234
Negative	13.3% (12.6%-14.0%)	1		1	
Positive	14.1% (11.9%-16.3%)	0.87 (0.70-1.07)		0.87 (0.69-1.09)	
**Gender**			<0.001		<0.001
Male	6.7% (5.9%-7.6%)	1		1	
Female	19.8% (18.8%-20.8%)	4.37 (3.71-5.15)		4.45 (3.69-5.38)	
**Age, years**			<0.001		<0.001
15-24	3.9% (3.3%-4.5%)	1		1	
25-34	8.2% (7.1%-9.3%)	2.24 (1.79-2.8)		1.96 (1.49-2.58)	
35-44	12.2% (10.7%-13.8%)	3.57 (2.87-4.34)		3.24 (2.46-4.27)	
45-54	17.6% (15.4%-19.8%)	5.79 (4.63-7.26)		5.68 (4.27-7.56)	
55-64	27.6% (24.0%-31.2%)	10.93 (8.49-14.07)		11.03 (8.00-15.20)	
65 and over	43.7% (40.1%-47.2%)	24.32 (19.37-30.53)		26.96 (19.84-36.64)	
**Site**			<0.001		<0.001
Bonda Mission (subsistence farming area)	14.1% (12.3%-15.9%)	1		1	
Eastern Highlands (tea estate)	9.4% (4.6%-7.6%)	0.92 (0.66-1.29)		0.84 (0.59-1.19)	
Selbourne (forestry estate)	9.4% (7.8%-11.0%)	1.04 (0.79-1.39)		1.15 (0.86-1.51)	
Nyazura (town)	13.3% (11.4%-15.1%)	1.40 (1.08-1.82)		1.36 (1.04-1.77)	
Nyanga (town)	9.3% (7.5%-11.1%)	1.09 (0.82-1.46)		0.82 (0.59-1.15)	
Watsomba (roadside settlement)	16.9% (15.0%-18.7%)	1.43 (1.13-1.8)		1.49 (1.17-1.89)	
Sakubva (urban)	28.2% (24.9%-31.5%)	5.28 (4.03-6.91)		4.06 (2.97-5.54)	
Hobhouse (urban)	15.5% (12.8%-18.1%)	2.73 (2.03-3.66)		1.98 (1.41-2.78)	
**Employment**			0.001		0.006
Unemployed	19.5% (18.3%-20.6%)	1		1	
Employed or in education	9.0% (8.2%-9.8%)	0.79 (0.68-0.91)		0.80 (0.69-0.94)	
**Relationship status**			<0.001		<0.001
Married or in a long-term relationship	14.6% (13.6%-15.5%)	1		1	

For peer review only - http://bmjopen.bmj.com/site/about/guidelines.xhtml

Never been in a long-term relationship	3.9% (3.2%-4.7%)	0.50 (0.38-0.66)		0.54 (0.40-0.72)	
Separated or divorced	15.8% (13.1%-18.6%)	0.92 (0.72-1.18)		1.01 (0.78-1.31)	
Widowed	35.9% (32.3%-39.4%)	0.67 (0.54-0.83)		0.74 (0.58-0.94)	
**Education**			<0.001		-
None or primary	24.1% (22.3%-26.0%)	1		-	
Secondary or higher	10.1% (9.5%-10.9%)	1.52 (1.24-1.86)		-	
**Asset-based wealth**			<0.001		<0.001
1 (poorest)	12.3% (8.2%-16.5%)	1		1	
2	12.0% (11.0%-13.1%)	1.13 (0.71-1.82)		1.27 (0.79-2.04)	
3	12.5% (11.2%-13.7%)	1.47 (0.91-2.36)		1.66 (1.03-2.68)	
4	16.5% (15.1%-18.0%)	2.61 (1.62-4.21)		2.10 (1.27-3.48)	
5 (richest)	20.8% (15.5%-26.1%)	3.29 (1.83-5.91)		3.76 (2.02-7.03)	

Association between ART-exposure and self-reported hypertension

Over half of PLHIV were ART-exposed (65.8%, 62.8-69.0%, Table 1). The prevalence of self-reported hypertension was similar among ART-exposed PLHIV (14.8%, 12.0%-17.7%) and ART-naïve PLHIV in crude comparisons (12.8%, 9.1%-16.4%, F=0.7, p=0.388) and the lack of a difference remained when analysing the prevalence of self-reported hypertension by age and gender (Supplementary fig. 1).

In logistic regression analysis, there was not strong evidence of a relationship between ART-exposure and self-reported hypertension after adjustment for age and gender (OR=0.87, 95% CI=0.56-1.33, p=0.519) or after further adjustment for site (0.83, 0.53-1.30, p=0.411; Table 3). However, amongst PLHIV, the oldest people (aged 65 years and above) had the highest odds of self-reporting hypertension (12.51, 4.16-37.67, p<0.001, versus ages 15-24 years), the odds of self-reporting hypertension were higher for women than men (5.73, 3.23-10.17, p<0.001), and urban participants had higher odds of self-reporting that they were hypertensive than those from the subsistence farming area (Table 3).

For peer review only

Table 3. Antiretroviral Therapy as a determinant of hypertension in people living with HIV.

	Weighted hypertension prevalence, % (95% Confidence Interval)	Odds ratio adjusted for only age and gender (95% confidence interval)	P-value	Fully adjusted odds ratio (95% confidence interval)	P-value
**ART-exposure[1]**			0.519		0.411
ART-naïve	12.8% (9.1%-16.4%)	1		1	
ART-exposed	14.8% (12.0%-17.7%)	0.87 (0.56-1.33)		0.83 (0.53-1.30)	
**Gender**			<0.001		<0.001
Male	6.0% (3.3%-8.8%)	1		1	
Female	20.3% (17.1%-23.6%)	5.11 (2.95-8.84)		5.73 (3.23-10.17)	
**Age, years**			<0.001		<0.001
15-24	7.9% (2.6%-13.1%)	1		1	
25-34	12.3% (7.8%-16.8%)	1.69 (0.72-3.94)		2.03 (0.86-4.8)	
35-44	13.1% (9.2%-17.0%)	1.87 (0.83-4.23)		2.58 (1.12-5.93)	
45-54	12.0% (8.2%-15.8%)	2.01 (0.88-4.58)		2.8 (1.19-6.59)	
55-64	24.7% (14.7%-34.6%)	6.19 (2.43-15.75)		9.54 (3.43-26.49)	
65 and over	27.8% (13.8%-41.9%)	7.53 (2.71-20.94)		12.51 (4.16-37.67)	
**Site**			<0.001		<0.001
Bonda Mission (subsistence farming area)	13.3% (7.8%-18.9%)	1		1	
Eastern Highlands (tea estate)	6.1% (2.0%-10.2%)	0.64 (0.26-1.57)		0.64 (0.26-1.56)	
Selbourne (forestry estate)	8.3% (3.9%-12.6%)	0.82 (0.37-1.82)		0.81 (0.37-1.8)	
Nyazura (town)	19.6% (13.5%-25.8%)	2.13 (1.1-4.14)		2.15 (1.1-4.17)	
Nyanga (town)	6.1% (0.1%-12.0%)	0.44 (0.13-1.45)		0.43 (0.13-1.44)	
Watsomba (roadside settlement)	10.6% (5.8%-15.3%)	0.84 (0.42-1.67)		0.82 (0.41-1.64)	
Sakubva (urban)	30.9% (20.9%-40.8%)	4.58 (2.21-9.51)		4.53 (2.19-9.4)	
Hobhouse (urban)	25.8% (14.4%-37.3%)	3.29 (1.43-7.54)		3.27 (1.43-7.5)	
**Employment**			0.352		-
Unemployed	16.0% (12.7%-19.3%)	1		-	
Employed or in education	12.3% (9.3%-15.4%)	1.2 (0.81-1.78)		-	
**Relationship status**			0.671		-
Married or in a long-term relationship	12.4% (9.6%-15.2%)	1		-	

Never been in a long-term relationship	11.1% (4.2%-18.0%)	0.81 (0.33-2.03)		-	
Separated or divorced	17.2% (10.9%-23.4%)	1.17 (0.7-1.95)		-	
Widowed	19.8% (13.6%-25.9%)	0.81 (0.5-1.31)		-	
**Education**			0.562		-
None or primary	17.6% (12.8%-22.4%)	1		-	
Secondary or higher	12.6% (10.0%-15.1%)	1.15 (0.72-1.81)		-	
**Asset-based wealth**			<0.001		-
1 (poorest)	8.1% (0.0%-17.0%)	1		-	
2	11.2% (8.1%-14.4%)	1.36 (0.38-4.81)		-	
3	11.5% (7.9%-15.1%)	1.48 (0.41-5.28)		-	
4	22.2% (16.4%-28.0%)	3.21 (0.9-11.42)		-	
5 (richest)	42.4% (17.8%-67.0%)	8.58 (1.57-46.88)		-	

[1]ART, antiretroviral therapy.

Discussion

This study aimed to examine whether HIV status and ART-exposure were associated with self-reported hypertension in Manicaland, East Zimbabwe. In this setting, approximately one in seven PLHIV self-reported hypertension. There was no evidence of a difference in the prevalence of self-reported hypertension by HIV status. Furthermore, no evidence was found for an association between ART-exposure and self-reported hypertension among PLHIV.

The results of this study corroborate previous estimates of the prevalence of hypertension in Zimbabwean PLHIV. The prevalence of self-reported hypertension in PLHIV in Manicaland was 14.1% (11.9%-16.3%), which is similar to earlier estimates of 8.9%, 18.3% and 19.8% from elsewhere in the country.[8–10] The alignment of this study's findings with previous estimates indicates that the results may be a realistic representation of the prevalence of hypertension. However, this means that there is already an important burden of hypertension in Zimbabwe, which is likely to grow as the population grows older and unhealthy lifestyles become more widespread.[24,25] Consequently, more prevention and treatment services may be required to avoid worsening outcomes.

For peer review only

Knowledge of the burden of hypertension experienced by PLHIV is key to providing prevention and treatment services as efficiently as possible, particularly given the scare resources available.[25,26] A variety of physiological and treatment-related mechanisms by which HIV could raise or lower hypertension risk have been proposed, making epidemiological studies vital to furthering understanding.[27,28] This study found a similar prevalence of self-reported hypertension among PLHIV and HIV-negative people. These findings differ from those from a systematic review which pooled prevalence estimates gathered across Africa and indicated that PLHIV might experience a lower prevalence of hypertension.[6] The difference may be explained in part by regional variation in the relationship between HIV and hypertension within Africa and in part due to the different approaches to detection of hypertension that were used. The systematic review focused on studies reporting clinical diagnoses of hypertension, excluding data on self-reported hypertension as is presented here.[6] Further, a study of data from the Agincourt Health and Demographic Surveillance System in South Africa, which neighbours Zimbabwe, also reported that the prevalence of hypertension among PLHIV was comparable to that of HIV-negative people.[29] The South African study and the global systematic review suggest that regional differences may exist, which should be fully accounted for when developing guidelines for scale-up of hypertension services.[6,29] Notably, it will be important to identify and manage other risk factors that are associated with hypertension in Zimbabwe.

Our findings further indicated that the prevalence of self-reported hypertension was similar among ART-exposed and ART-naïve PLHIV. Several large studies, including the Data Collection on Adverse Events of Anti-HIV Drugs (D:A:D) study, have obtained similar results, but a previous study in Zimbabwe reported a higher prevalence of hypertension among PLHIV on ART.[8,30] The conflicting results from the two Zimbabwean studies may be due to differences in how ART-exposure was measured. While this study focused on whether PLHIV had ever taken ART, the previous Zimbabwean study examined the effect of taking ART for more than five years.[8] Investigating long-term ART-exposure could have led the previous study to capture the effect of older ART, which may have changed fat distribution and increased hypertension risk more than newer treatments.[31] Detailed data on ART interruption and discontinuation over time are not available from the Manicaland Study. However, additional research examining how patterns of ART-exposure over

For peer review only

time relate to hypertension risk might clarify the source of these differences and give further insight into how hypertension services should be delivered.

Strengths and limitations

To our knowledge, this is the first study to provide estimates of the prevalence of hypertension among comparable populations of PLHIV and HIV-negative people in a non-clinical setting in Zimbabwe. This study also used robust methodology to explore links between HIV status, ART-exposure, and self-reported hypertension, building the evidence base on links between HIV and NCDs. In addition, the use of data from a large population survey should have increased the generalisability of the findings to other similar settings in Zimbabwe. Finally, all analyses were underpinned by individual-level data that included a diverse range of men and women, and could be weighted to local census data, which enhanced the representativeness of results.

However, this work also has some limitations. These fall across four broad areas: the definition of hypertension, unmeasured risk factors for hypertension, selection biases towards the healthy, and the cross-sectional nature of the analysis. Firstly, the reliance on self-report of previous diagnoses by a doctor or nurse as a proxy to detect disease may have resulted in underestimation of the underlying hypertension burden.[32] Limited access to screening and diagnosis will likely have meant that some hypertensive people in our study population were at the early stages of the hypertension care cascade and unaware of their hypertension, and so were excluded from our estimates.[33–36] This would reduce the generalisability of our findings for settings in which hypertension screening and diagnosis is more readily available. In addition, HIV-negative people may have had less contact with the healthcare system for hypertension diagnosis than PLHIV, which could have artificially reduced self-reporting of hypertension in this group.[37] Yet the prevalence of hypertension was comparable among PLHIV who knew their status (14.7%, 11.9%-17.5%) and PLHIV who did not (15.1%, 10.6%-19.5%), suggesting that contact with the healthcare system among diagnosed PLHIV did not increase self-reporting of hypertension. In future survey rounds, quantification of the prevalence of hypertension by measuring participants' blood pressure would provide more reliable estimates of this burden, and the results of this analysis could be updated accordingly.[38]

For peer review only

As well as limitations linked to the definition of hypertension, data were not available on several risk factors for hypertension, such as diet and physical activity, which precluded their use in the analysis.[39] Moreover, people with hypertension, or conditions caused by hypertension, were more likely to be seriously ill and so unable to complete the individual interview, or in locations, such as hospitals, which were not reached by the field teams. Finally, assessment of the association between HIV and ART and self-reported hypertension was constrained by the cross-sectional nature of the analysis. The development of a cohort that consisted of matched PLHIV and HIV-negative controls in Manicaland, which could be followed over time, would allow more certainty in the drawing of conclusions about the relationship.[8]

Conclusion

Approximately one in seven PLHIV self-reported hypertension in Manicaland, Zimbabwe. However, HIV status and ART-exposure were not associated with self-reported hypertension. Zimbabwe is re-orienting its health system towards control of hypertension and other NCDs; this study highlights how carefully designed, sufficiently resourced prevention and care interventions for PLHIV and HIV-negative people will be required to make this a success.

For peer review only

Acknowledgements

The authors would like to express our gratitude to everyone who has been involved with the Manicaland Study over the past 20 years, especially the participants and research staff. Without them, this study would not exist.

Funding

This work was supported by the Wellcome Trust [220098/Z/20/Z to KD]; the Bill and Melinda Gates Foundation [OPP1161471 to SG]; the Civilian Research and Development Foundation Global (prime is President's Emergency Plan for AIDS Relief) [OISE-9531011 to MS]; the National Institutes of Health [5R01MH114562-02 to SG and CN; 1R21AG053093-01 to MS] and the Medical Research Council Centre for Global Infectious Disease Analysis [MR/R015600/1 to KD, LM, CN, MS and SG], which is jointly funded by the UK Medical Research Council and the UK Foreign, Commonwealth & Development Office (FCDO), under the MRC/FCDO Concordat agreement and is also part of the EDCTP2 programme supported by the European Union.

The funders had no role in study design, data collection and analysis, decision to publish, or preparation of the manuscript.

Competing interests

SG declares holding shares in AstraZeneca and GlaxoSmithKline. All other authors declare that no competing interests exist.

Authors' contributions

MS, SG and KD conceived the analysis and developed the methods. SG, CN, RM, PM, and TM led the data collection and TD and LM cleaned, extracted, and prepared the data. KD performed the analysis and generated figures and estimates. KD led the writing of the manuscript with MS and SG, and all authors commented on and contributed to the finalisation of the manuscript.

For peer review only

Data availability

Data are available upon reasonable request. The data are deidentified participant data and can be obtained by sending a data request form (available from the Manicaland Study website: http://www.manicalandhivproject.org/data.html) to s.gregson@imperial.ac.uk.

Ethics statement

The Manicaland Study was conducted with the understanding and informed consent of participants. Ethical approval was given by Imperial College Research Ethics Committee (17IC4160), the Biomedical Research and Training Institute Institutional Review Board (AP140/2017) and the Medical Research Council of Zimbabwe (MRCZ/A/2243).

For peer review only

References

1 Stanaway JD, Afshin A, Gakidou E, *et al.* Global, regional, and national comparative risk assessment of 84 behavioural, environmental and occupational and metabolic risks or clusters of risks for 195 countries and territories 1990-2017: A systematic analysis for the Global Burden of Disease Study. *Lancet* 2018;**392**:1923–94. doi:10.1016/S0140-6736(18)32225-6

2 Mills KT, Bundy JD, Kelly TN, *et al.* Global Disparities of Hypertension Prevalence and Control. *Circulation* 2016;**134**:441–50. doi:10.1161/CIRCULATIONAHA.115.018912

3 Angkurawaranon C, Nitsch D, Larke N, *et al.* Ecological Study of HIV Infection and Hypertension in Sub-Saharan Africa: Is There a Double Burden of Disease?. *PLoS One* 2016;**11**:e0166375. doi:10.1371/journal.pone.0166375

4 Althoff KN, Smit M, Reiss P, *et al.* HIV and ageing: Improving quantity and quality of life. *Curr Opin HIV AIDS* 2016;**11**:527–36. doi:10.1097/COH.0000000000000305

5 Zoest RA van, Born BJH van den, Reiss P. Hypertension in people living with HIV. *Curr Opin HIV AIDS* 2017;**12**:513–22. doi:10.1097/COH.0000000000000406

6 Davis K, Perez-Guzman P, Hoyer A, *et al.* Association between HIV infection and hypertension: a global systematic review and meta-analysis of cross-sectional studies. *BMC Med* 2021;**19**:105. doi:10.1186/s12916-021-01978-7

7 Dalal S, Beunza JJ, Volmink J, *et al.* Non-communicable diseases in sub-Saharan Africa: what we know now. *Int J Epidemiol* 2011;**40**:885–901. doi:10.1093/ije/dyr050

8 Gonah L, Moodley I, Hlongwana K. Prevalence of diabetes mellitus and hypertension in people living with human immunodeficiency virus on antiretroviral therapy in Gweru district, Zimbabwe. *African J Prim Heal Care Fam Med* 2020;**12**:2473. doi:10.4102/PHCFM.V12I1.2473

9 Cheza A, Tlou B, Zhou DT. Incidence of non-communicable diseases (NCDs) in HIV patients on ART in a developing country: Case of Zimbabwe's Chitungwiza Central Hospital—A retrospective cohort study (2010–2019). *PLoS One* 2021;**16**:e0252180.

For peer review only

doi:10.1371/journal.pone.0252180

10 Bleasel JM, Heron JE, Shamu T, *et al.* Body mass index and noninfectious comorbidity in HIV-positive patients commencing antiretroviral therapy in Zimbabwe. *HIV Med* 2020;**21**:674–9. doi:10.1111/HIV.12934

11 Zimbabwe Ministry of Health and Child Care. Zimbabwe Health Financing Strategy 2017. Harare, Zimbabwe: 2017. https://zdhr.uz.ac.zw/xmlui/bitstream/handle/123456789/1374/Zimbabwe National Health Financing Strategy 2017.pdf?sequence=1&isAllowed=y

12 Government of Zimbabwe. Zimbabwe National HIV and AIDS strategic plan: 2021-2025. Harare: 2021. https://www.nac.org.zw/wp-content/uploads/2022/02/ZIMBABWE-NATIONAL-HIV-STATEGIC-PLAN_2021-2025-1.pdf.

13 Government of Zimbabwe. Vision 2030: Towards a Prosperous & Empowered Upper Middle Income Society by 2030. Harare: 2018. http://www.zim.gov.zw/index.php/en/government-documents.

14 Manicaland General Population Cohort. Manicaland HIV project. 2021.http://www.manicalandhivproject.org/.

15 Gregson S, Mugurungi O, Eaton J, *et al.* Documenting and explaining the HIV decline in east Zimbabwe: the Manicaland General Population Cohort. *BMJ Open* 2017;**7**:e015898. doi:10.1136/bmjopen-2017-015898

16 Manicaland Centre for Public Health Research. Multi-Level Behavioural Economics and Community Psychology Approaches to Strengthen HIV Prevention Cascades in Adolescent Girls and Young Women in East Zimbabwe - Study Protocol. Harare: 2018.

17 Thomas R, Skovdal M, Galizzi MM, *et al.* Improving risk perception and uptake of pre-exposure prophylaxis (PrEP) through interactive feedback-based counselling with and without community engagement in young women in Manicaland, East Zimbabwe: study protocol for a pilot randomized trial. *Trials 2019 201* 2019;**20**:1–9. doi:10.1186/S13063-019-3791-8

18 Thomas R, Skovdal M, Galizzi MM, *et al.* Improving risk perception and uptake of voluntary

For peer review only

medical male circumcision with peer-education sessions and incentives, in Manicaland, East Zimbabwe: Study protocol for a pilot randomised trial. *Trials* 2020;**21**:108. doi:10.1186/s13063-020-4048-2

19 Lopman B, Lewis J, Nyamukapa C, *et al.* HIV incidence and poverty in Manicaland, Zimbabwe: is HIV becoming a disease of the poor? *AIDS* 2007;**21 Suppl 7**:S57-66. doi:10.1097/01.aids.0000300536.82354.52

20 Tlhajoane M, Eaton JW, Takaruza A, *et al.* Prevalence and Associations of Psychological Distress, HIV Infection and HIV Care Service Utilization in East Zimbabwe. *AIDS Behav* 2018;**22**:1485–95. doi:10.1007/s10461-017-1705-x

21 Rhead R, Elmes J, Otobo E, *et al.* Do female sex workers have lower uptake of HIV treatment services than non-sex workers? A cross-sectional study from east Zimbabwe. *BMJ Open* 2018;**8**:18751. doi:10.1136/bmjopen-2017-018751

22 R Core Team. R: A language and environment for statistical computing. 2020.https://www.r-project.org/.

23 Lumely T. Analysis of complex survey samples. *J Stat Softw* 2004;**9**:1–19. doi:10.18637/jss.v009.i08

24 Ibrahim MM, Damasceno A. Hypertension in developing countries. *Lancet* 2012;**380**:611–9. doi:10.1016/S0140-6736(12)60861-7

25 Smit M, Olney J, Ford NP, *et al.* The growing burden of noncommunicable disease among persons living with HIV in Zimbabwe. *AIDS* 2018;**32**:773–82. doi:10.1097/QAD.0000000000001754

26 Oparil S, Acelajado MC, Bakris GL, *et al.* Hypertension. *Nat Rev Dis Prim* 2018;**4**:18014. doi:10.1038/nrdp.2018.14

27 Fahme SA, Bloomfield GS, Peck R. Hypertension in HIV-Infected Adults: Novel Pathophysiologic Mechanisms. *Hypertension* 2018;**72**:44–55. doi:10.1161/HYPERTENSIONAHA.118.10893

28 Okello S, Ueda P, Kanyesigye M, *et al.* Association between HIV and blood pressure in

For peer review only

adults and role of body weight as a mediator: Cross-sectional study in Uganda. *J Clin Hypertens* 2017;**19**:1181–91. doi:10.1111/jch.13092

29 Clark SJ, Gomez-Olive FX, Houle B, *et al.* Cardiometabolic disease risk and HIV status in rural South Africa: establishing a baseline. *BMC Public Health* 2015;**15**:135. doi:10.1186/s12889-015-1467-1

30 Hatleberg CI, Ryom L, d'Arminio Monforte A, *et al.* Association between exposure to antiretroviral drugs and the incidence of hypertension in HIV-positive persons: the Data Collection on Adverse Events of Anti-HIV Drugs (D:A:D) study. *HIV Med* 2018;**19**:605–18. doi:10.1111/hiv.12639

31 van Zoest RA, Wit FW, Kooij KW, *et al.* Higher Prevalence of Hypertension in HIV-1-Infected Patients on Combination Antiretroviral Therapy Is Associated With Changes in Body Composition and Prior Stavudine Exposure. *Clin Infect Dis* 2016;**63**:205–13. doi:10.1093/cid/ciw285

32 Ataklte F, Erqou S, Kaptoge S, *et al.* Burden of Undiagnosed Hypertension in Sub-Saharan Africa. *Hypertension* 2015;**65**:291–8. doi:10.1161/HYPERTENSIONAHA.114.04394

33 Mungati M, Manangazira P, Takundwa L, *et al.* Factors affecting diagnosis and management of hypertension in Mazowe District of Mashonaland Central Province in Zimbabwe: 2012. *BMC Cardiovasc Disord* 2014;**14**:102. doi:10.1186/1471-2261-14-102

34 Sorato MM, Davari M, Kebriaeezadeh A, *et al.* Reasons for poor blood pressure control in Eastern Sub-Saharan Africa: looking into 4P's (primary care, professional, patient, and public health policy) for improving blood pressure control: a scoping review. *BMC Cardiovasc Disord* 2021;**21**. doi:10.1186/s12872-021-01934-6

35 Wozniak G, Khan T, Gillespie C, *et al.* Hypertension Control Cascade: A Framework to Improve Hypertension Awareness, Treatment, and Control. *J Clin Hypertens* 2016;**18**:232–9. doi:10.1111/JCH.12654

36 Berry KM, Parker WA, McHiza ZJ, *et al.* Quantifying unmet need for hypertension care in South Africa through a care cascade: evidence from the SANHANES, 2011-2012. *BMJ Glob Heal* 2017;**2**:e000348. doi:10.1136/BMJGH-2017-000348

For peer review only

37 Manne-Goehler J, Montana L, Gomez-Olive FX, *et al.* The ART Advantage: Health Care Utilization for Diabetes and Hypertension in Rural South Africa. *J Acquir Immune Defic Syndr* 2017;**75**:561–7. doi:10.1097/QAI.0000000000001445

38 Thomas R, Burger R, Hauck K. Richer, wiser and in better health? The socioeconomic gradient in hypertension prevalence, unawareness and control in South Africa. *Soc Sci Med* 2018;**217**:18–30. doi:10.1016/j.socscimed.2018.09.038

39 World Health Organisation. A global brief on hypertension. Geneva: 2013. https://apps.who.int/iris/handle/10665/79059.

For peer review only

Legends

Figure 1. Flow of study participants. DBS, dried blood spot.

Figure 2. Prevalence of reported hypertension in men and women by age and HIV status. Error bars: 95% confidence intervals.

For peer review only

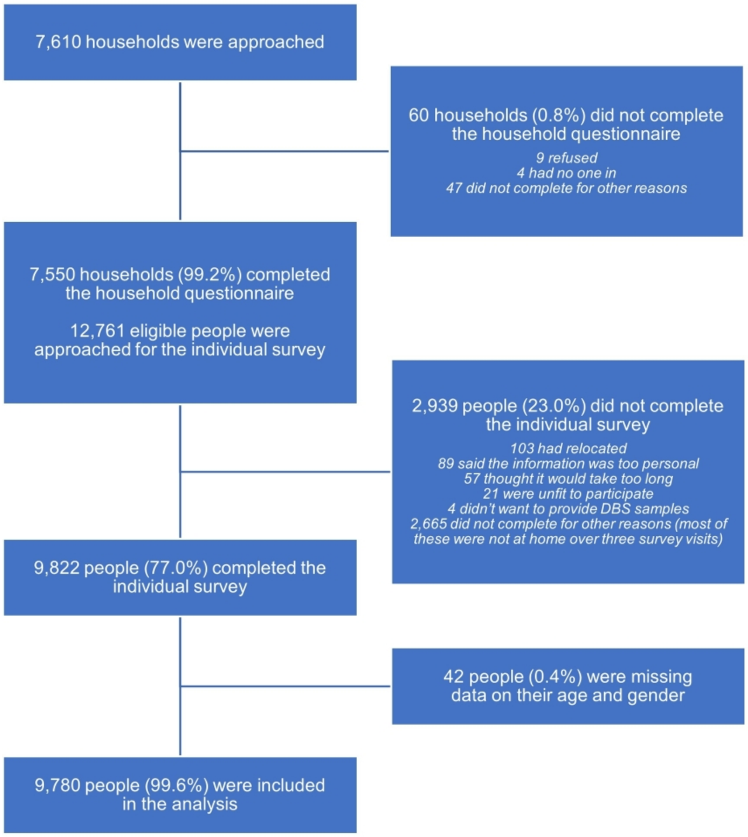

178x233mm (330 x 330 DPI)

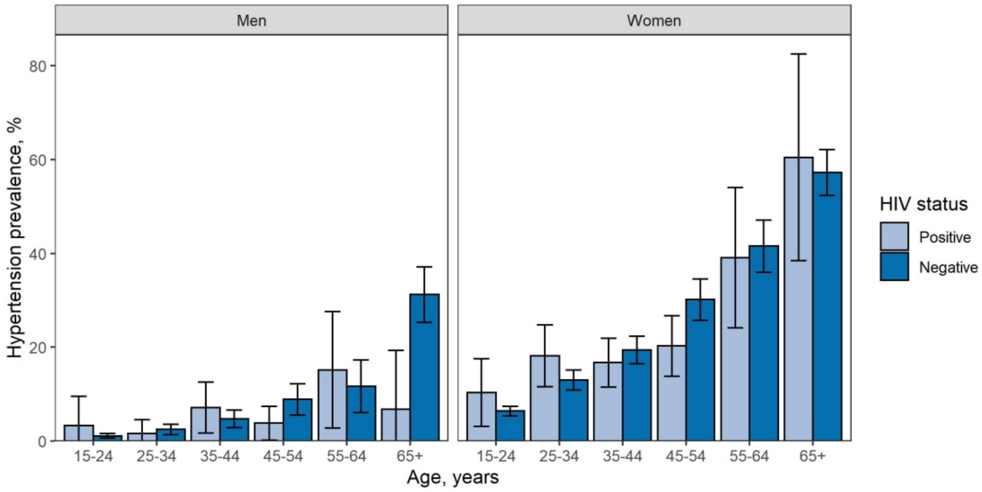

Figure 2. Prevalence of reported hypertension in men and women by age and HIV status. Error bars: 95% confidence intervals.

203x101mm (300 x 300 DPI)

Supplementary material

Supplementary table 1. Reported hypertension (HPN) prevalence by HIV status, age, and gender. 2

Supplementary figure 1. Prevalence of reported hypertension in men and women by age and ART exposure. Error bars: 95% confidence intervals. 3

Manicaland Study Questionnaires. 4

For peer review only

Supplementary table 1. Reported hypertension (HPN) prevalence by HIV status, age, and gender.

Gender	Age	People living with HIV			HIV-negative people			F-statistic	p-value
		Weighted prevalence, % (95% Confidence Interval)	N for those with HPN[1]	N for those with no HPN[1]	Weighted prevalence, % (95% Confidence Interval)	N for those with HPN[1]	N for those with no HPN[1]		
Men	15-24	3.2% (0-8.5%)	1	29	1.1% (0.6-1.6%)	17	1,590	1.3	0.261
Men	25-34	1.5% (0-4.5%)	1	57	2.4% (1.3-3.6%)	18	721	0.2	0.645
Men	35-44	7.1% (1.6-12.6%)	6	80	4.7% (2.8-6.5%)	23	474	0.9	0.343
Men	45-54	3.8% (0.1-7.4%)	4	100	8.8% (5.5-12.2%)	25	261	2.9	0.090
Men	55-64	15.1% (2.7-27.6%)	5	26	11.6% (6.0-17.2%)	15	116	0.3	0.586
Men	65+	6.7% (0-19.3%)	1	18	31.2% (25.3-37.2%)	74	168	4.1	0.043
Women	15-24	10.3% (3.1-17.5%)	7	60	6.4% (5.3-7.4%)	133	1,932	1.7	0.197
Women	25-34	18.1% (11.6-24.7%)	24	107	12.9% (10.8-15.1%)	123	819	2.6	0.104
Women	35-44	16.7 (11.5-21.9%)	33	163	19.4% (16.4-22.3%)	134	555	0.7	0.402
Women	45-54	20.2% (13.8-26.7%)	30	118	30.1% (25.7-34.5%)	124	287	5.4	0.021
Women	55-64	39.1% (24.1-54.0%)	16	25	41.5% (36.0-47.1%)	124	176	0.1	0.763
Women	65+	60.5% (38.5-82.5%)	11	8	57.2% (52.3-62.1%)	233	174	0.1	0.778

[1]Values are unweighted. Individuals without a recorded HIV status or with missing data for hypertension reporting were excluded.

For peer review only

Supplementary figure 1. Prevalence of reported hypertension in men and women by age and ART exposure. Error bars: 95% confidence intervals.

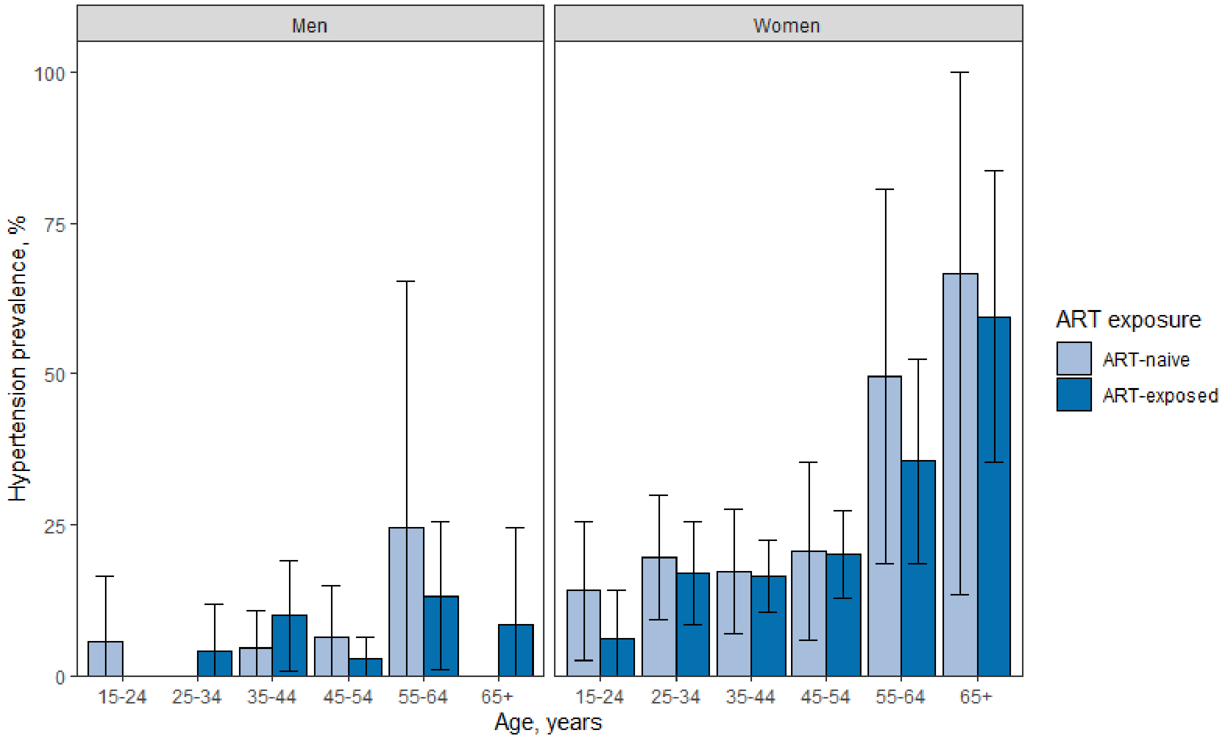

For peer review only - http://bmjopen.bmj.com/site/about/guidelines.xhtml

Manicaland Study Questionnaires.

The household and individual questionnaires are copied below.

For peer review only

HOUSEHOLD QUESTIONNAIRE **FORM A**

**Individual questionnaire eligibility:**	
**IVQs: young adults**	**Y N**
**IVQs: older adults**	**Y N**

**Questionnaire processing dates:**	
**Corrections generated**	
**Corrections completed**	
**Batch**	

HOUSEHOLD IDENTIFICATION

Q001 **Census district:** ____ **Ward:** [ ]

Q002 **Village:** ____

Q003 **Name of household head (R6):** ____

Q004 **Name of household head (R7):** ____

Q005 **Category of household (R6)*:** [ ]

Q006 **Study site reference & cluster.** [ ][ ]

Q007 **Household number:** [ ]

Q008 **Growth point or compound:** **Yes** 1 / **No** 2

Q009 **Distance from nearest (tarred) roadside business centre** [ ]

Q010 **GPS coordinates** **Longitude** [ ] **Latitude** [ ] **Altitude** [ ]

**INTERVIEWER VISIT**		**1**	**2**	**3**
**Q011**	**Date:**	____	____	____
**Q012**	**Time:**	____	____	____
**Q013**	**Interviewer:**	____	____	____
**Q014**	**Result**:**	[ ]	[ ]	[ ]

Q015 ***How many deaths have there been in the household in the last 12 months?*** [ ]

Q016 ***How many of the people who died were aged 15-59 years at their last birthday?*** [ ]

Do NOT correct after completing interview.

CHECKED BY SUPERVISOR

Q017 **Signature:** ____

Q018 **Date:** ____

*HOUSEHOLD CATEGORY	
Follow-up household from R6	1
Household refused at R6	4
Household missed at R6	5
New household in the area since R6	8
New study site	6
Other (specify) ____________	98

**RESULT CODES	
Completed	1
No one at home	2
Household away for duration of survey	3
Postponed	4
Refused	5
Dwelling vacant or address not a dwelling	6
Dwelling destroyed	7
Dwelling not found	8
Other (specify) ____________	9
Household relocated - within study areas	10
Household relocated - outside study areas	11
Household dispersed (split up)	12

For peer review only - http://bmjopen.bmj.com/site/about/guidelines.xhtml

HOUSEHOLD MEMBERS FORM: UPDATE ON MEMBERS RECORDED IN 2012-2013 SURVEY VISIT

Q. No:

"Now I would like to get some information about the people who were staying in the household when we came to see you last time"...

LINE NO	USUAL RESIDENTS AND REGULAR VISITORS (ROUND 6)	REL'SHIP TO H.O.H. (R6)	(R7)	SPOUSE IN SAME HOUSEHOLD		SEX	AGE ('12-'13)	PARENTS SURVIVAL FOR CHILDREN UNDER 18 YEARS OLD: FALIVE	FBC	YFDIED	MALIVE	MBC	YMDIED	EDUCATION		HOUSEHOLD MEMBER'S SURVIVAL STATUS NOW			RESIDENCE				ELIGIBILITY FOR INTERVIEW: IVDONE ('12-'13)	CAT'GY (R6)	SELECT (R6)
Q019	Q020	Q021	Q022	Q023		Q024	Q025	Q026		Q027	Q028		Q029	Q030	Q031	Q032			Q034	Q035	Q036	Q037		Q038	Q039
	Circle line no. of respondent.			*Is (NAME) married to another member of this household?* If yes: record spouse's line no.				*Is (NAME)'s NATURAL BIOLOGICAL father still alive?* Tick "BC" if checked identity - birth certificate.		Year died	*Is (NAME)'s NATURAL BIOLOGICAL mother still alive?* Tick "BC" if checked identity - birth certificate.		Year died	*State highest level of education completed.***		*Is (NAME) still alive?* If died, record month and year of death. Go to Q035 if more than a year ago.			*Was (NAME) staying here in the same month last year?*	*Does (NAME) still stay in this household on a regular basis? (at death)*	*How many nights is it since (NAME) last slept in / left (see Q035) this household?*	*How many nights has (NAME) slept here in the last month?*	Codes for CATEGORY: …		
				Y N	#	M F	Years	Y N DK	BC	Year	Y N DK	BC	Year	Level	Years	Y N DK	Mth	Yr	Y N	Y N	# or >1yr	#	MUT6NO	#	Code
1	Serina Nyauzanza	1		1 2		2	46	1 2 8			1 2 8					1 2 8			1 2	1 2				1	7
2	Angelina Nyazuna	3		1 2		2	27	1 2 8			1 2 8					1 2 8			1 2	1 2			1	2	1
3	Edmore Nyazuna	5		1 2		1	8	1 2 8			1 2 8					1 2 8			1 2	1 2				3	
4	Telma Nyazuna	5		1 2		2	4	1 2 8			1 2 8					1 2 8			1 2	1 2				4	

For peer review only - http://bmjopen.bmj.com/site/about/guidelines.xhtml

HOUSEHOLD MEMBERS FORM Q. No:

"Now I would like to get some information about the people who were NOT members of the household when we came 5 years ago but who are staying here now or who have done so since we came last time " ...

LINE NO	USUAL RESIDENTS AND REGULAR VISITORS‡	RELATED TO H.O.H. (R7)	SPOUSE IN SAME HOUSEHOLD		SEX	AGE	PARENTS SURVIVAL RELATIONSHIP TO CARER FOR CHILDREN UNDER 18 YEARS OLD						EDUCATION		HOUSEHOLD MEMBER'S SURVIVAL STATUS		RESIDENCE					ELIGIBILITY FOR INTERVIEW CAT'Y	SELECT
Q019	Q020	Q022	Q023		Q024	Q025	Q026		Q027	Q028		Q029	Q030	Q031	Q032		Q033	Q034	Q035	Q036	Q037	Q038	Q039
	Please give me the names of the new persons who have been staying (physically) in this househo on a regular basis (starting with the head of household) Circle line no. of respondent.	*What is the relationship of (NAME) to the head of household?*	*Is (NAME) married to another member of this household?* If yes: record spouse's line no.		*Is (NAME) male or female?*	*How old is (NAME) ?*	*Is (NAME)'s* NATURAL BIOLOGICAL *father still alive?* Tick "BC" if checked identity - birth certificate.		Year died.	*Is (NAME)'s* NATURAL BIOLOGICAL *mother still alive?* Tick "BC" if checked identity - birth certificate.		Year died.	*State highest level of education completed.***		*Is (NAME) still alive?*		*When did (NAME) start staying in this house-hold?*	*Was (NAME) staying here in the same month last year?*	*Does (NAME) STILL stay in this household on a regular basis?* (/at death)	*How many nights is it since (NAME) last slept in this household?*	*How many nights has (NAME) slept here in the last month?*	Codes …	Circle line no if person selected for inter-view***
		See footnote*	Y N	#	M F	Years	Y N DK	BC	Year	Y N DK	BC	Year	Level	Years	Y N DK	Mth Yr	Mth Yr	Y N	Y N	#	#		
70 1			1 2		1 2		1 2 8			1 2 8					1 2 8			1 2	1 2				70 1
70 2			1 2		1 2		1 2 8			1 2 8					1 2 8			1 2	1 2				70 2
70 3			1 2		1 2		1 2 8			1 2 8					1 2 8			1 2	1 2				70 3
70 4			1 2		1 2		1 2 8			1 2 8					1 2 8			1 2	1 2				70 4
70 5			1 2		1 2		1 2 8			1 2 8					1 2 8			1 2	1 2				70 5
70 6			1 2		1 2		1 2 8			1 2 8					1 2 8			1 2	1 2				70 6
70 7			1 2		1 2		1 2 8			1 2 8					1 2 8			1 2	1 2				70 7
70 8			1 2		1 2		1 2 8			1 2 8					1 2 8			1 2	1 2				70 8
70 9			1 2		1 2		1 2 8			1 2 8					1 2 8			1 2	1 2				70 9
71 0			1 2		1 2		1 2 8			1 2 8					1 2 8			1 2	1 2				71 0

Tick here, if a further continuation sheet is used: ☐

Enter total numbers of individuals selected for interview:

- AGYW (15-24 yrs) ☐
- Young men (15-29 yrs) ☐
- Older adults (25/30+ yrs) ☐

"Just to make sure that I have a complete listing" ...

Q040	*Are there any other persons such as small children or infants that we have not yet listed?*	Number: ☐	Add each in table above. ☐
Q041	*In addition, are there any other people who may not be members of your family, such as lodgers, friends or domestic servants who live here now or have done so in the last three years?*	Number: ☐	Add each in table above. ☐
Q042	*Are there any other family members or other people who usually stay elsewhere, but who sleep here from time to time and stayed overnight within the last month?*	Number: ☐	Add each in table above. ☐
Q043	*Are there any other people who stayed here for a while but have now passed away?*	Number: ☐	Add each in table above. ☐

* CODES FOR Q021: RELATIONSHIP TO HEAD OF HOUSEHOLD

01	Head	07	Parent-in-law	13	Paternal uncle
02	Wife or husband	08	Brother or sister (natural)	14/15	Maternal grandfather/mother
03	Son or daughter (natural)	09	Stepfather/mother (father's co-wife)	16/17	Paternal grandfather/mother
04	Son or daughter-in-law	10	Maternal aunt	18	Other relative/cousin
05	Grandchild	11	Paternal aunt	19	Adopted/foster child
06	Father / mother (natural)	12	Maternal uncle	20	Not related

** CODES FOR Q030 & Q031: LEVEL & YEARS OF EDUCATION

Level:		Years: years completed:	
0	None	0	Less than one year completed
1	Primary	98	Don't know
2	Secondary		

***ELIGIBLE PERSONS (Q038/Q039)

Usual residents or regular visitors who stayed in the household at least 4 nights last month
- 11 AGYW (15-24 yrs)
- 12 Young men (15-29 yrs)
- 13 Older adults (25/30+ yrs) - 'Yes' households only

‡ Regular visitors are people who, on average, stay in the household at least once a month

FORM A

For peer review only - http://bmjopen.bmj.com/site/about/guidelines.xhtml

HOUSEHOLD SOCIO-ECONOMIC STATUS

Q. No: [ ]

REF.	QUESTIONS & FILTERS	CODING CATEGORIES		SKIP TO
Q044	*What is the main source of drinking water for members of your household?*	**Piped into residence**	1	
		**Private tap in yard or plot**	2	
		**Communal tap**	3	
		**Own well or borehole**	4	
		**Other well or borehole**	5	
		**Protected spring**	6	
		**Other (specify)**	10	
Q045	*What kind of toilet facility does your household have?*	**Flush toilet**	1	
		**Blair toilet**	2	
		**Pit latrine**	3	
		**Other (specify)**	4	
		**No facilities**	5	- Q047
Q046	*Is this toilet facility used by members of your household alone, shared with neighbours, or is it communal?*	**Household alone**	1	
		**Shared with neighbours**	2	
		**Communal**	3	
Q047	*Does your household have:*		Y N	
	Electricity?	**Electricity**	1 2	
	A refridgerator?	**Refridgerator**	1 2	
	A radio?	**Radio**	1 2	
	A television?	**Television**	1 2	
Q048	**Record house type.**	**Pole and dagga structure**	1	
		**Brick house - thatched roof**	2	
		**Brick house - tiled/sheeting roof**	3	
		**Cabin/other**	8	
Q049	**Observe and record type of floor of the main dwelling.**	**Natural floor (earth/sand/dung)**	1	
		**Rudimentary (planks/palm/bamboo)**	2	
		**Finished (wood/cement/carpet...)**	3	
Q050	*Does any member of your household own? …*		Y N	
	A bicycle?	**Bicycle**	1 2	
	A motorcycle?	**Motorcycle**	1 2	
	A car?	**Car**	1 2	
	A tractor?	**Tractor**	1 2	
Q051	*How many cattle does your household own?*	**Number of cattle**	[ ]	

REF.	QUESTIONS & FILTERS	Y	N	DK
Q052	*Now I would like to ask you some questions about food: During the last 12 MONTHS, was there a time when:*			
	1) You or others in your household worried about not having enough food to eat because of a lack of money or other resources?	1	2	98
	2) Still thinking about the last 12 MONTHS, was there a time when you or others in your household were unable to eat healthy and nutritious food because of a lack of money or other resources?	1	2	98
	3) Was there a time when you or others in your household ate only a few kinds of food because of lack of money or other resources?	1	2	98
	4) Was there a time when you or others in your household had to skip a meal because there was not enough money or other resources to get food?	1	2	98
	5) Still thinking about the last 12 MONTHS, was there a time when you or others in your household ate less than you thought you should because of a lack of money or other resources?	1	2	98
	6) Was there a time when your household ran out of food because of a lack of money or other resources?	1	2	98
	7) Was there a time when you or others in your household were hungry but did not eat because there was a lack of money or other resources for food?	1	2	98
	8) Was there a time when you or other in your household went without eating for a whole day because of a lack of money or other resources?	1	2	98

For peer review only

For peer review only - http://bmjopen.bmj.com/site/about/guidelines.xhtml

HOUSEHOLD MEMBERS FORM: INDIVIDUAL INTERVIEW PROCESSING

Q. No:

**Study site:**		**HHID:**	
**Village:**		**Growth point:**	
**Name of household head (R7):**		**Cell #:**	

LINE NO	HOUSEHOLD MEMBERS SELECTED FOR INDIVIDUAL INTERVIEW	CONTACT DETAILS	APPOINTMENTS									IVQ REF.
Q053	Q054	Q055	Q056			Q057			Q058			Q059
From Q019 (ii/iii)	Name	Cell #	1st App't			2nd App't			3rd App't			Note ref.
No.	*Adults (15 yrs +)*		Time	Day/Mth	Res(1)	Time	Day/Mth	Res(2)	Time	Day/Mth	Res(3)	MUT7NO

(1) RESULT CODES

1. Completed
2. Partially completed - questionnaire only
3. Partially completed - BE only
4. Partially completed - questionnaire & BE only (HTC outstanding)
5. Missed appointment - rescheduled
6. Missed appointment - requires follow-up
7. Attended but refused
8. Other (specify)

* Result fields to be completed by supervisors

For peer review only

For peer review only - http://bmjopen.bmj.com/site/about/guidelines.xhtml

Individual questionnaire - baseline survey

For peer review only

INDIVIDUAL QUESTIONNAIRE ***FORM B***

MUT7number:

Questionnaire processing dates:

Consent form
Corrections completed

QUESTIONNAIRE IDENTIFICATION

**Q001**	**Census district:**	______	**Ward:**
**Q002**	**Village:**	______	**Cluster:**
**Q003**	**Name of head of household:**	______	
**Q004**	**Category of respondent:**		R6 MUTNO
**Q005**	**Study site reference:**	Date left R6 area	R6 site
**Q006**	**Household number:**		
**Q007**	**Line number on household questionnaire:**		

INTERVIEWER VISIT

		1	2	3
**Q008**	**Date:**			
**Q009**	**Time:**			
**Q010**	**Interviewer:**			
**Q011**	**Result**:**			

Q012 **Interview timing:**

Before BE measurement 1
After BE measurement 2

CHECKED BY SUPERVISOR

Q013 **Signature:** ______

Q014 **Date:** ______

*RESPONDENT CATEGORY	
Follow-up respondent (from R6)	1
New respondent: previously under age	2
New respondent: in-migrant (since R6)	5
New respondent: selected but unavailable R6	6
New respondent: selected but refused R6	7
New respondent: household missed R6	9
New respondent: new study site in R7	10
Other (specify) ______	8

**RESULT CODES	
Completed	1
Not at home	2
Refused	3
Partially completed	4
Sick/hospital	5
Out-migrated	100
Other (specify) ______	8

For peer review only

INDIVIDUAL QUESTIONNAIRE: **INVITATION TO JOIN THE STUDY**

Explain the purpose of the study - including potential benefits nationally and to the community.
Explain what is involved in participating in the study - show/read invitation letter and consent form.
Ask about and discuss any concerns the respondent might have.
Seek consent to participate in the study - request signature on consent form.

REF.	QUESTIONS & FILTERS	CODING CATEGORIES		SKIP TO
Q101	Indicate whether the respondent wishes to participate in the study.	Yes	1	- Q103
		No	2	
Q102	Indicate the main reason why he/she does not wish to participate.	Insufficient time*	1	
	* Ask for another appointment if reason is insufficient time.	DBS samples	3	
		Information too personal	4	
		Other (specify)	8	
Q103	Record details of others present at this point.		Yes / No	
		Children under 10	1 / 2	
		Husband/wife	1 / 2	
		Other males	1 / 2	
		Other females	1 / 2	

INDIVIDUAL QUESTIONNAIRE: **BACKGROUND CHARACTERISTICS** ***Q. No:***

REF.	QUESTIONS & FILTERS	CODING CATEGORIES		SKIP TO
Q201	Record the current time (24 hour clock).	Hour / Minutes	hr / mins	
Q202	Record sex of respondent.	Male	1	
		Female	2	
Q203	*In what month and year were you born?*		mnth / yr	
Q204	*How old were you at your last birthday?* Check consistency with Q203.	Age in COMPLETED years	yrs	
Q205	*Are you currently enrolled in school full-time?*	Yes	1	- Q208
		No	2	
Q206	*How old were you when you left school?*	Age in completed years	yrs	
		Never been to school	99	- Q209
Q207	*What was your reason for leaving school?*	Insufficient funds	1	
		Found a job	2	
		To go to college or university	3	
		Inadequate exam passes	4	
		Needed to help at home	5	
		Marriage	6	
		Pregnancy/childbirth - voluntarily	7	
		Pregnancy - expelled	8	
		Caught having sex - expelled	9	
	If parents decided, probe for the underlying reason.	Expelled - other reasons	10	
		Finished secondary school	11	
		Other (specify)	18	
Q208	*What is the highest grade of school you have completed?*		Level / Years	
		None	0	
		Primary	1 / 1-7	
	For "years", enter number of years (excl. repeats) at highest level reached.	Secondary	2 / 1-6	- Q210
		Higher	3 / 1-6	- Q210
Q209	*Can you read a letter or newspaper in any language?*	Yes	1	
		No	2	
Q210	*How long have you been living in this homestead?*	Years	yrs	
		Since birth	995	
		Visitor	996	

For peer review only

INDIVIDUAL QUESTIONNAIRE: **BACKGROUND CHARACTERISTICS** *Q. No:*

REF.	QUESTIONS & FILTERS	CODING CATEGORIES	CODE	SKIP TO
Q211	*How long have you been living in (NAME OF VILLAGE)?*	Years<br>Since birth<br>Visitor	yrs<br>995<br>996	 - Q213
Q212	*What type of place was your previous place of residence?*<br>Record place of current home if the respondent is a visitor.<br>"Roadside" here means a tarred road.<br>Record the name of the place below.	Large town or city<br>Small town<br>Growth point<br>Commercial estate/mine<br>Roadside business centre<br>Rural business centre<br>Communal/resettlement area	1<br>2<br>3<br>4<br>5<br>6<br>7	
Q213	*Which church denomination do you belong to?*	Traditional<br>Pentecostal<br>Methodist<br>Anglican<br>Roman Catholic<br>ZAOGA<br>Apostolic Faith Mission<br>Marange Apostolic<br>Mazowe Apostolic<br>Zviratidzo Apostolic<br>Other Apostolic (specify)<br>Zionist<br>Mughodi<br>Other (specify)<br>None	1<br>2<br>4<br>5<br>6<br>7<br>8<br>9<br>10<br>12<br>13<br>15<br>20<br>17<br>97	
Q214	*In which sector of employment do you work?*	Estates: tea, coffee, forestry etc<br>Manufacturing or building trade<br>Police or army<br>Teacher: primary school<br>Teacher: secondary school<br>Nurse<br>Services or retail: shops<br>Informal: petty trading (veg etc)<br>Informal: subsistence agriculture<br>Student<br>Unemployed: excl. agriculture<br>Other (specify)	1<br>2<br>3<br>4<br>5<br>6<br>7<br>8<br>9<br>10<br>11<br>12	 <br><br><br><br><br><br>- Q216<br>- Q216<br>- Q216<br>- Q216
Q215	*What type of work do you do?*	Professional or managerial<br>Self-employed: small business<br>Skilled labour<br>Manual/unskilled labour	1<br>2<br>3<br>4	
Q216	*Over the last 12 months, how many times per month, on average, have you had a drink containing alcohol?*	Number of times per month		- Q218 if '0'
Q217	*How many drinks containing alcohol do you have on a typical sitting?*	Number of drinks per sitting		
Q218	*How many times have you visited a bar or beer-hall in the last month?*	Number of times		
Q219	*Do you smoke cigarettes?*	Yes<br>No	1<br>2	
Q220	*Do you take any of these types of drugs for pleasure?*	 Injecting drugs<br>Drugs you smoke<br>Prescription drugs<br>Other drugs you swallow	Yes / No<br>1 / 2<br>1 / 2<br>1 / 2<br>1 / 2	
Q221	*Have you ever been married or in a long-term or cohabiting relationship?*<br>Relationships of 12 months or more should be treated as "long-term".	Yes<br>No	1<br>2	 - Q235
Q222	*How many such relationships have you experienced in your lifetime?*	Include current relationships.		
Q223	*How old were you when you first entered such a relationship?*	Age (years)	yrs	

For peer review only

For peer review only - http://bmjopen.bmj.com/site/about/guidelines.xhtml

INDIVIDUAL QUESTIONNAIRE: **BACKGROUND CHARACTERISTICS** *Q. No:*

REF.	QUESTIONS & FILTERS	CODING CATEGORIES					SKIP TO
Q224	*Are you currently widowed, divorced or separated from your most recent spouse/partner?*	Widowed	1				- Q235
		Divorced	2				- Q235
		Separated	3				- Q235
		Still in union	4				
Q225	*How many spouses/long-term partners do you have at present?*	(Not zero!)					
	For women, ask how many other wives her husband has.	Ask questions Q230 to Q238 for first spouse, then the second, and so on ...					
Q226	*How old was your partner at his/her last birthday?*	Age in completed years	yrs	yrs	yrs	yrs	
		Don't know	98	98	98	98	
Q227	*Did you and your spouse have an HIV test before you agreed to get married?*	Self only tested	2	2	2	2	
		Partner only tested	3	3	3	3	
		Neither tested	4	4	4	4	- Q229
		Not yet 'married'	5	5	5	5	- Q229
		Both tested seperately	11	11	11	11	
		Both tested together	12	12	12	12	- Q229
Q228	*Did you tell each other your results?*	Yes	1	1	1	1	
	Ask equivalent if only one tested.	No	2	2	2	2	
Q229	*Do you know whether this person has HIV infection now?*	Infected	1	1	1	1	
		Uninfected	2	2	2	2	- Q231
		Prefers not to say	96	96	96	96	- Q231
	If yes, ask for partner's status.	Don't know	98	98	98	98	- Q231
Q230	*Is this person receiving ART (i.e. treatment to prevent AIDS)?*	Yes	1	1	1	1	
		No	2	2	2	2	
		Prefers not to say	96	96	96	96	
		Don't know	98	98	98	98	
Q231	*Has he/she had a sexually transmitted infection (other than HIV) in the last months?*	Yes	1	1	1	1	- Q233 if respondent is male
		No	2	2	2	2	
		Don't know	98	98	98	98	
Q232	*Has he been circumcised?*	Yes - full	1	1	1	1	
	Show respondent pictures to establish whether fully or partially circumcised.	Yes - partial	2	2	2	2	
		No	3	3	3	3	
		Don't know	98	98	98	98	
Q233	*What is the highest grade of school your partner has completed?*	None	0	0	0	0	
		Primary	1	1	1	1	
		Secondary	2	2	2	2	
		Higher	3	3	3	3	
Q234	*In which sector of employment does he/she work?*	Estates	1	1	1	1	
		Manuf'trg/building	2	2	2	2	
		Police/army	3	3	3	3	
		Teacher: primary	4	4	4	4	
		Teacher: secondary	5	5	5	5	
		Nurse	6	6	6	6	
		Services/retail: shops	7	7	7	7	
		Informal: trading	8	8	8	8	- Q235
		Informal: incl agric	9	9	9	9	- Q235
		Student	10	10	10	10	- Q235
		Unemployed: excl. agr	11	11	11	11	- Q235
		Other (specify)	12	12	12	12	
Q235	*What type of work does he/she do?*	Prof/manage't	1	1	1	1	
		Self-employed	2	2	2	2	
		Skilled labour	3	3	3	3	
		Manual/unskilled	4	4	4	4	
Q236	*Which of the following groups exist in your home area and which are you a member of?*			Exist	Member	Rating	
		Church groups					
		Women's groups					
		Co-operative					
	For those where he/she is a member:	Farmers group					
		Burial society					
	Would you say that this group functions: (1) well; (2) OK; or (3) poorly?	Savings club (RCS)					
		Youth group					
		Sports club					
		HIV/AIDS group					
		Political party					
Q237	*What is the principal activity of the group you spend the most time with?*	Code from Q235					
		None	99				

For peer review only

For peer review only - http://bmjopen.bmj.com/site/about/guidelines.xhtml

INDIVIDUAL QUESTIONNAIRE: **BACKGROUND CHARACTERISTICS** *Q. No:*

REF.	QUESTIONS & FILTERS	CODING CATEGORIES			SKIP TO
Q238	*Do you play one or more of these roles in your community?*		Yes	No	
		Chief, Headman or Kraal Head	1	2	
		DA or CEO	1	2	
		Political leader (MP, councillor …)	1	2	
		Church leader	1	2	
		Teacher	1	2	
		Traditional healer	1	2	
		Faith healer	1	2	
		Doctor or nurse	1	2	
		Village health worker	1	2	
		Formal sector manager/employer	1	2	
		Other (specify)	1	2	
Q239	*Are you related to a young woman aged between 15-24 years in the following ways?*		Yes	No	
		Parent	1	2	
		Sister or brother (same parents)	1	2	
		Aunt or uncle	1	2	
		Grandparent	1	2	
		Spouse or regular partner	1	2	
Q240	*Are you related to a young man aged between 15-29 years in the following ways?*		Yes	No	
		Parent	1	2	
		Sister or brother (same parents)	1	2	
		Aunt or uncle	1	2	
		Grandparent	1	2	
		Spouse or regular partner	1	2	

PSYCHOLOGICAL HEALTH

"In the past 2 weeks, how often have you been bothered by any of the following problems? …"

REF.	QUESTION	Not at all	Several days	> 1/2 days	Nearly every day
Q301	*Little interest or pleasure in doing things*	1	2	3	4
Q302	*Feeling down, depressed, or hopeless*	1	2	3	4
Q303	*Trouble falling asleep, staying asleep, or sleeping too much*	1	2	3	4
Q304	*Feeling tired or having little energy*	1	2	3	4
Q305	*Poor appetite or overeating*	1	2	3	4
Q306	*Feeling bad about yourself - or that you're a failure or have let yourself or your family down*	1	2	3	4
Q307	*Trouble concentrating on things such as reading a newspaper or watching TV*	1	2	3	4
Q308	*Moving or speaking so slowly that other people could have noticed. Or, the opposite - being fidgety or restless that you have been moving around a lot more than usual*	1	2	3	4
Q309	*Thoughts that you would be better off dead or of hurting yourself in some way*	1	2	3	4
		Not hard at all	Some-what hard	Very hard	Extreme-ly hard
Q310	*If you had any of these problems, how hard have these problems made it for you to do your work, take care of things at home, or get along with other people?*	1	2	3	4

For peer review only - http://bmjopen.bmj.com/site/about/guidelines.xhtml

INDIVIDUAL QUESTIONNAIRE: **SEXUAL RELATIONSHIPS** **Q. No:**

REF.	QUESTIONS & FILTERS	CODING CATEGORIES		SKIP TO
Q401	10 minutes of informal discussion first to build rapport, trust & stress absence of prejudice. Explain the need to ask questions on the respondent's own experience of sexual relationships. Stress the importance of providing accurate information. Stress that strict confidentiality will be maintained - request privacy.			
Q402	*How old were you when you had sex for the first time?* Explain what we mean by "having sex".	Age in years	yrs	- Q404
		Not yet had sex	99	
Q403	*What is the main reason you have not yet started to have sexual relations?*	Too young	1	- Q434
		Not met partner	2	- Q434
		Not yet married	3	- Q434
		Risk of pregnancy	4	- Q434
		Risk of HIV/AIDS	5	- Q434
		Other (specify)	8	- Q434
Q404	*How many days is it since you last had sex?*		days	
	Skip to Q406 if less than one month.	More than one year	99	
Q405	*What is the main reason you are currently abstaining from sexual relations?* Options 1-4 could refer to the respondent or (if male) to his regular partner.	Current pregnancy	1	
		Recent birth	2	
		Terminal abstinence	3	
		Self or partner has an STD	4	
		Currently living apart	5	
		Risk of catching HIV/AIDS	6	
		Risk of passing on HIV/AIDS	7	
		Self or partner has HIV/AIDS	8	
		Religious reasons	9	
		Not currently married	10	
		No partner - although would like one	11	
		Ill-health	12	
		Under 1 year since spouse died	13	
		Don't like / not interested in having sex	14	
		Other (specify)	20	
		Don't know	98	
Q406	*Did you use condoms throughout the last time you had sex?*	Yes	1	
		No	2	
Q407	*Do you know whether the last person you had sex with has HIV infection?* If infected, ask if on ART.	Infected - on ART	1	
		Infected - not on ART	2	
		Uninfected	3	
		Prefers not to say	4	
		Don't know	98	
Q408	*If you took a guess, how many partners other than yourself (and any co-wives) do you think your current spouse/partner has had in the last 12 months?*	Number of non-regular partners		
Q409	Indicate data collection method used. For 'secret voting': (i) explain the procedure and the confidentiality safeguards carefully. (ii) establish whether able to use tablet.	Secret voting	1	
		Interview (if respondent unable to use tablet)	2	
Q410	*How many different REGULAR sexual partners have you had in your LIFETIME? By REGULAR, I mean someone you have been having sex with for a year or more.*	Number of partners	1	

For peer review only - http://bmjopen.bmj.com/site/about/guidelines.xhtml

INDIVIDUAL QUESTIONNAIRE: **SEXUAL RELATIONSHIPS** *Q. No:*

REF.	QUESTIONS & FILTERS	CODING CATEGORIES		SKIP TO
Q411	*For how many years have you been using condoms EVERY TIME you have sex with a REGULAR partner?*	Years<br>Ask respondent to write "0" if he/she does not use condoms with current regular partner(s).	2	
Q412	*How many different NON-REGULAR sexual partners have you had in your LIFETIME?*	Number of partners	3	
Q413	*For how many years have you been using condoms EVERY TIME you have sex with a NON-REGULAR sexual partner?*	Years<br>Ask respondent to write "0" if he/she doesn't use condoms with non-regular sexual partner(s) now.<br>Ask respondent to write "P" if he/she has never had a non-regular sexual partner.	4	
Q414	*How many different sexual partners have you had in the LAST 12 MONTHS?*	Number of partners	5	
Q415	*How many of these partners were you having sex with for the FIRST TIME?*	Number of new partners in last 12 months (STRESS)	6	
Q416	*How many of the sexual partners that you had in the last 12 months were REGULAR partners?*	Number of regular partners	7	
Q417	*How many of the sexual partners that you had in the last 12 months were NON-REGULAR partners?*<br>Ask respondent to check that this and the previous answer should add up to box #5.	Number of non-regular partners	8	
Q418	*How many sexual relationships do you consider yourself to be involved in at the moment?*	Number of current relationships	9	
Q419	*How many different partners have you had sex with in the last month?*	Number of partners in last month (total)	10	
Q420	Explain that you now wish to ask some questions about the last 3 persons the respondent had sex with.<br>Note: NO time restriction. Stress that these may be people he/she had sex with only once.<br>If secret voting is being used, ask the respondent to enter a "P" in each box if he/she has not had the minimum required number of partners in his/her lifetime.			
			LAST / PREVIOUS ...	
Q421	*How many times have you had sexual intercourse with this partner in the last 2 weeks?*	Number of times		1
Q422	*On how many of these occasions did you and your partner use condoms THROUGHOUT?*	Number of times		2
Q423	*Did you use condoms throughout the LAST TIME you had sex with this partner?*	Yes<br>No		3
Q424	*What was the month and year when you had sexual intercourse with this person for the FIRST time?*	Month first then year	mnth / yr (×3)	4
Q425	*When was the month and year you LAST had sexual intercourse with this person?*	Month first then year	mnth / yr (×3)	5

For peer review only

INDIVIDUAL QUESTIONNAIRE: **SEXUAL RELATIONSHIPS** ***Q. No:***

REF.	QUESTIONS & FILTERS	CODING CATEGORIES		SKIP TO
Q426	*Are you still having sex with this person?*	Yes<br>No		6
Q427	*Where were you when you had sex with this person for the first time?*<br>If secret voting is being used, show respondent the picture codes. (Code numbers as for Q212).	Code		7
Q428	*How many years old is this person?*	Age in years	yrs yrs yrs	8
Q429	*Is / was this person someone you are / were married to or cohabiting with?*	Yes<br>No		9
Q430	*Is this person married to someone other than yourself?*	Yes<br>No<br>Don't know		10
Q431	*Have you given or received money, goods or services in exchange for sex with this person in the last month?*	Yes<br>No		11
Q432	For men:<br>*Did you have sex with one or more men in the last 12 months?*	Yes<br>No	1<br>2	12
Q433	For men:<br>*Have you EVER been involved in a non-marital relationship of any kind where you gave anything in exchange for sex?*	Yes<br>No	1<br>2	- Q435<br>13
Q434	For women:<br>*Have you EVER been involved in a non-marital relationship of any kind where you received anything in exchange for sex?*	Yes<br>No	1<br>2	14
Q435	Record details of others present at this point.	Children under 10<br>Husband/wife<br>Other males<br>Other females	Y(1) N(2)<br>Y(1) N(2)<br>Y(1) N(2)<br>Y(1) N(2)	

For peer review only - http://bmjopen.bmj.com/site/about/guidelines.xhtml

INDIVIDUAL QUESTIONNAIRE: **HIV PREVENTION METHODS** **Q. No:**

REF.	QUESTIONS & FILTERS	CODING CATEGORIES	SKIP TO
Q501	*"Now I would like to ask you some questions about the methods of HIV prevention that you know about ..."*		
Q502	*What methods of HIV prevention have you heard about?*<br><br>Ask first without probing and then probe for factors that are not mentioned spontaneously.	Spont / Probed / Where / Who<br>VMMC<br>PrEP<br>Male condoms<br>Female condoms<br>Spiritual protection<br>Faithfulness to one partner<br>Abstinence<br>HIV testing & counselling<br>Treatment as prevention<br>Other (specify)	
Q503	For each method of HIV prevention mentioned (after probing), except TasP, ask WHERE (venue or activity) they obtained most of their information about this method<br>Enter in the 3rd column in Q502 the code from the list given here that matches the response.	HIV testing service (integrated) 1<br>HIV testing service (stand-alone) 2<br>Health centre (staff and/or posters, leaflets etc.) 3<br>School (lessons, health visitors, clubs) 4<br>Home (visits from health or community worker) 5<br>Workplace 6<br>Church service or meeting 7<br>Shop (e.g. pharmacy) 8<br>Social media 9<br>Internet (other) 10<br>TV, radio or newspaper 11<br>Community group meetings (e.g. womens group, RCS …) 12<br>Other community meetings 13<br>Word of mouth 14<br>Other (specify) 15	
Q504	For each method of HIV prevention mentioned (after probing), except TasP, WHO was the organisation or person that they got most of their information about this method from).<br>Enter in the 4th column in Q502 the code from the list given here that matches the response.	Ministry of Health staff 1<br>Health centre committee member(s) 2<br>Community health worker 3<br>Community leaders (Chief, counsellor) 4<br>Church leader or members 5<br>Traditional healer 6<br>PSI 7<br>FACT 8<br>Other NGO (specify) 9<br>Other (specify) 12	
Q505	*What were the main APPROACHES used by these organisations to encourage people like you to use each of these methods of HIV prevention?*<br>1. Info'n campaigns - e.g. mass media, posters, leaflets<br>2. Practical skills training - e.g. how to use the method<br>3. Social skills training - e.g. negotiating skills<br>4. Counselling<br>5. Mass distribution - e.g. mobile or outreach services<br>6. Text message reminders<br>7. Financial or material incentives<br>Tick each approach that applies for each method of HIV prevention mentioned in Q502. Enter '99' if not applicable. (e.g. because the HIV prevention method is not known).	1 / 2 / 3 / 4 / 5 …<br>VMMC<br>PrEP<br>Male condoms<br>Female condoms<br>Faithfulness to one partner<br>Abstinence<br>HIV testing & counselling	
Q506*	*Which methods of HIV prevention have you used in your lifetime?*<br><br>Enter '1' is 'Yes'; '2' if No'.<br><br>Probe to find out if more than one method has been used.	Ever / Now / Provider<br>VMMC<br>PrEP<br>Male condoms<br>Female condoms<br>Faithfulness to one partner<br>Abstinence<br>TasP<br>Other (specify)	
Q507**	*Which of the methods you know are you using now?*<br>Probe to find out if more than one method is being used.	Enter response in 2nd column in Q506.	
Q508	For methods used, ask for and record the current or most recent provider. Use codes from Q504.	Enter response in 3rd column in Q506.	
	Introduction explaining / defining VMMC & traditional circumcision.<br>For women, go to Q533.		

For peer review only

For peer review only - http://bmjopen.bmj.com/site/about/guidelines.xhtml

INDIVIDUAL QUESTIONNAIRE: HIV PREVENTION METHODS Q. No:

REF.	QUESTIONS & FILTERS	CODING CATEGORIES		SKIP TO
Q509**	*Have you ever had medical, traditional or religious male circumcision?*	Medical	1	- Q511
		Traditional or Religious (non-medical)	2	
		Both	3	- Q511
		None	8	
Q510	*Have you ever been offered VMMC?*	Yes	1	- Q519
		No	2	- Q519
	If not been offered, ask if heard of VMMC before.	Not heard of VMMC before today	99	- Q519
Q511	*Was it full or partial circumcision?*	Full	1	
	Show pictures to help distinguish.	Partial	2	- Q519
Q512	*Was the medical circumcision a surgical or non-surgical procedure?*	Surgical	1	
		Non-surgical	2	
	e.g. PREPEX is a non-surgical form of VMMC.	Don't know	98	
Q514	*How old were you when you received VMMC?*		yrs	- Q531 if <10 yrs
Q515	*Which of these organisations provided you with the VMMC service?*		Yes / No	
		Ministry of Health	1 / 2	
		PSI	1 / 2	
		ITEC	1 / 2	
	______________________	Other (specify)	1 / 2	
Q516	*How many visits to this place did you have to make for this procedure?*	Number of visits		
	i.e. from the first appointment to the final check-up. Record date of first appointment.	Date of first appointment	mth / yr	
Q517	*What costs did you incur when you had the VMMC done?*	Medical fees	$	
		Transport costs	$	
		Accommodation costs	$	
		Foregone income	$	
	______________________	Other costs (specify)	$	
Q518	*After you received VMMC, did you:*		More / Same / Less	
	(1) Increase, decrease or not change your number of sexual partners?		1 / 2 / 3	
	(2) Use condoms more, less or the same as before?		1 / 2 / 3	
	(3) Use condoms consistently with casual partners?	Yes	1	- Q529
		No	2	- Q529
		NA - no casual partners	99	- Q529
Q519	*Please tell me whether you strongly disagree, disagree, neither agree nor disagree, agree or strongly agree with the following statements …*		SD / D / N / A / SA	
	(1) I am confident I can get VMMC if I wanted to		1 / 2 / 3 / 4 / 5	
	(2) I am confident that I can get VMMC even if my partner disapproves of this		1 / 2 / 3 / 4 / 5	
	(3) I am confident I can get VMMC even if my friends disapprove of this		1 / 2 / 3 / 4 / 5	
	(4) I am confident I can get VMMC even if my parents and family elders disapprove		1 / 2 / 3 / 4 / 5	
Q520	*Are you able to discuss getting VMMC with your partner?*	Yes - already done so	1	
		Yes - not done already but can do this	2	
		No	3	
		No regular partner	99	- Q522
Q521	*Would your partner disapprove if you had VMMC?*	Yes	1	
		No	2	
		Don't know	3	
Q522**	*If you wanted to get VMMC yourself, do you know a place where someone like you can easily have it done?*	Yes	1	
		No	2	
Q523*	*If you wanted to get VMMC yourself, how easy would it be for you to access the service?*	Very easy	1	
		Easy	2	
		Neither easy nor difficult	3	
	Ask for an answer on a scale of 5 - from 1 (very easy) to 5 (very difficult).	Difficult	4	
		Very difficult	5	
		Don't know	98	

For peer review only - http://bmjopen.bmj.com/site/about/guidelines.xhtml

INDIVIDUAL QUESTIONNAIRE: **HIV PREVENTION METHODS** **Q. No:**

REF.	QUESTIONS & FILTERS	CODING CATEGORIES		SKIP TO
Q524	*What factors make it impractical or unsuitable for someone like you to access VMMC services?*<br><br>Ask first without probing and then probe for factors that are not mentioned spontaneously.	(Spont / Probed)<br>High costs (including loss of income)<br>Inability to work during/after procedure<br>Healthcare providers are female<br>Lack of privacy / confidentiality<br>Limited opening hours<br>Distance / travel difficulties<br>Respondent is HIV+ (not suitable for PLHIV)<br>Other (specify)		 <br><br><br><br><br><br>- Q532
Q525**	*Do you want to get VMMC if the service was freely accessible to you?*	Yes<br>No<br>Not sure	1<br>2<br>8	
Q526*	*How definitely do you want to get VMMC if the service was freely accessible to you?*<br><br>Ask for an answer on a scale of 5 - from 1 (definitely) to 5 (definitely not).	Definitely<br>Probably<br>No opinion<br>Probably not<br>Definitely not	1<br>2<br>3<br>4<br>5	
Q527	*Do you plan to get VMMC?*	Yes<br>No<br>Don't know	1<br>2<br>98	- Q529<br>- Q529
Q528	*How soon do you plan to have VMMC?*	Number of months / years	mths / yrs	
Q529	*What factors were/are important in encouraging or discouraging you to have VMMC?*<br><br>For each factor given, ask if this was a positive (encouraging) or negative (discouraging) factor.<br><br>Ask first without probing and then probe for factors that are not mentioned spontaneously.<br><br>Tick probed box to indicate probed responses.	(Pos / Neg / Neutral / Probed)<br>HIV/STI/HPV protection (for myself)<br>HIV/STI/HPV protection (for my partner(s))<br>Method of HIV prevention I can control<br>Pain<br>Operation cannot be reversed<br>Make me feel responsible or ashamed<br>Make me feel more manly<br>Own sexual pleasure (better/worse)<br>Partner's sexual pleasure (better/worse)<br>Able to have erection for longer/shorter<br>More able to attract partners<br>Risk of getting an infection<br>Penis will be cleaner / look better<br>Using other prevention method (note method)<br>Abstaining or faithful to one trusted partner<br>Other (specify) ______________	 1 2 8<br>1 2 8<br>1 2 8<br>1 2 8<br>1 2 8<br>1 2 8<br>1 2 8<br>1 2 8<br>1 2 8<br>1 2 8<br>1 2 8<br>1 2 8<br>1 2 8<br>1 2 8<br>1 2 8<br>1 2 8	
Q530	*Which people's views were/are important in encouraging or discouraging you to have VMMC?*<br><br>Instructions as for last question.	(Pos / Neg / Neutral / Probed)<br>Religious leaders (religious beliefs)<br>Parents' or family elders' approval/views<br>Partner's views<br>Friends views<br>Community views<br>Other (specify) ______________	 1 2 8<br>1 2 8<br>1 2 8<br>1 2 8<br>1 2 8<br>1 2 8	
Q531	*How far away is it from where you live to the nearest place where VMMC services are provided?*	Distance in kms<br>Don't know	kms<br>98	
Q532	*By how much do you think VMMC reduces a man's risk of getting HIV infection?*<br>Ask for or convert response to a percent.	Percent reduction	%	
Q533	*Have many of your friends (or their male partners) had VMMC?*	Yes<br>No<br>Don't know	1<br>2<br>98	
Q534	*If you have a son who is a teenager or a young man, do you think it would be a good idea if he got VMMC?*	(Teen-ager / Young man)<br>Yes<br>No<br>Don't know	 1 1<br>2 2<br>98 98	- Q536 if male respondent
Q535	*Do you think it is a good thing for your male partner to have VMMC?*	Yes<br>No<br>Don't know	1<br>2<br>98	

For peer review only - http://bmjopen.bmj.com/site/about/guidelines.xhtml

INDIVIDUAL QUESTIONNAIRE: **HIV PREVENTION METHODS** *Q. No:*

REF.	QUESTIONS & FILTERS	CODING CATEGORIES	CODE	SKIP TO
Q536*	*The following statement is correct. Did you know this already?*<br>'Pre-exposure prophylaxis (PrEP) is when someone who does not have HIV takes a pill on an ongoing basis to prevent them getting HIV. Most people who use PrEP take a pill everyday. PrEP needs to be taken BEFORE sex for it to be effective.'	I knew this already<br>I wasn't sure about this<br>I didn't know about this<br>I don't understand this	1<br>2<br>3<br>4	 <br>- Q566<br>- Q566
Q537**	*Have you ever taken PrEP?*	Yes<br>No	1<br>2	- Q539
Q538	*Have you ever been offered PrEP?*	Yes<br>No	1<br>2	 - Q548
Q539**	*Are you taking PrEP currently?*	Yes<br>No	1<br>2	 - Q545
Q540	*When did you start taking PrEP this time?*<br>Emphasise this is for the current period of use.		mth / yr	
Q541	*Which of these organisations is providing you with PrEP?*	Ministry of Health<br>PSI<br>FACT<br>Other (specify)	Yes No<br>1 2<br>1 2<br>1 2<br>1 2	
Q542	*How often have you taken PrEP in the last month?*	Every day<br>Most days<br>Occasionally<br>Never	1<br>2<br>3<br>4	- Q546
Q543	*What was the longest number of days that you didn't take the PrEP pills in the last month?*	Number of days		
Q544	*How many days in the last week (7 days) did you take the PrEP pills?*	Number of days		- Q546 if all 7 days
Q545	*Why did you stop using PrEP / not use PrEP on some days?*	High costs<br>Forgot to take pills<br>Ran out of pills<br>Judgemental staff / stigma<br>Lack of confidentiality<br>Limited opening hours<br>Distance / travel difficulties<br>Sexual partner(s) disapproved<br>Parents / elders / friends disapproved<br>Using another prevention method (specify method<br>Not currently sexually active<br>Abstaining or faithful to one trusted partner<br>Experienced side effects<br>Stock-outs of PrEP drugs at clinic<br>Other (specify)<br>Don't know or no particular reason	1<br>2<br>3<br>4<br>5<br>6<br>7<br>8<br>9<br>10<br>11<br>12<br>13<br>14<br>15<br>98	 <br><br><br><br><br><br><br><br><br>- Q548
Q546	*How often do you have a meal at the same time when you take PrEP?*	Always<br>Most of the time<br>Sometimes<br>Never	1<br>2<br>3<br>4	
Q547	*After you started taking PrEP, did you:*<br>(1) Increase, decrease or not change your number of sexual partners?<br>(2) Use condoms more, less or the same as before?<br>(3) Use condoms consistently with casual partners?	More Same Less<br>1 2 3<br>1 2 3<br>Yes<br>No<br>NA - no casual partners	 <br><br>1<br>2<br>99	
Q548	*Please tell me whether you strongly disagree, disagree, neither agree nor disagree, agree or strongly agree with the following statements …*			
Q549	*Have you ever received instructions or counselling on how to use PrEP?*	Yes<br>No	1<br>2	

Q548:

Statement	SD	D	N	A	SA
(1) I am confident I can use PrEP if I wanted to	1	2	3	4	5
(2) I am confident I can use PrEP even if I have to take it every day	1	2	3	4	5
(3) I am confident I can use PrEP even if I have to take it always after a meal	1	2	3	4	5
(4) I am confident I can use PrEP even if I have to hide it from my partner	1	2	3	4	5
(5) I am confident I can use PrEP even if my friends disapprove of this	1	2	3	4	5
(6) I am confident I can use PrEP even if my parents and family elders disapprove	1	2	3	4	5
(7) I am confident I can use PrEP even if my community would think I have HIV	1	2	3	4	5

For peer review only

For peer review only - http://bmjopen.bmj.com/site/about/guidelines.xhtml

REF.	QUESTIONS & FILTERS	CODING CATEGORIES		SKIP TO
Q550	*Are you able to discuss taking PrEP with your partner?*	Yes - already done so<br>Yes - not done already but can do this<br>No current sexual partner	1<br>2<br>8	
Q551	*Would / does you partner disapprove if you use PrEP?*	Yes<br>No<br>No current sexual partner	1<br>2<br>8	
Q552**	*If / when you want to use PrEP, do you know a place where someone like you can easily get it?*	Yes<br>No	1<br>2	
Q553*	*If / when you want to take PrEP yourself, how easy is it for you to access the service?*<br><br>Ask for an answer on a scale of 5 - from 1 (very easy) to 5 (very difficult).	Very easy<br>Easy<br>Neither easy nor difficult<br>Difficult<br>Very difficult<br>Don't know	1<br>2<br>3<br>4<br>5<br>98	
Q554	*What factors make it impractical or unsuitable for someone like you to access PrEP services?*<br><br>Ask first without probing and then probe for factors that are not mentioned spontaneously.	(Spont / Probed)<br>High costs<br>Lack of privacy / confidentiality<br>Embarrassed to go / ask<br>Limited opening hours<br>Distance / travel difficulties<br>Not suitable for men<br>Below eligible age for PrEP<br>Other (specify)<br>Not currently sexually active<br>Respondent is HIV+ (not suitable for PLHIV)		Q559 - if on PrEP now<br><br><br><br>- Q557<br>- Q562
Q555**	*Do you want to use PrEP if it was freely accessible to you?*	Yes<br>No<br>Not sure	1<br>2<br>8	
Q556*	*How definitely do you want to use PrEP if it was freely accessible to you?*<br><br>Ask for an answer on a scale of 5 - from 1 (definitely) to 5 (definitely not).	Definitely<br>Probably<br>No opinion<br>Probably not<br>Definitely not	1<br>2<br>3<br>4<br>5	
Q557	*Do you plan to start using PrEP?*	Yes<br>No<br>Don't know	1<br>2<br>98	 - Q559
Q558	*How soon do you plan to start using PrEP?*	Number of months	mths	
Q559	*What factors were/are important in encouraging or discouraging you to use PrEP?*<br><br>For each factor given, ask if this was a positive (encouraging) or negative (discouraging) factor.<br><br>Ask first without probing and then probe for factors that are not mentioned spontaneously.<br>Tick probed box to indicate probed responses.	(Pos / Neg / Neutral / Probed)<br>HIV/STI protection<br>Method of HIV prevention I can control<br>Allow me to become pregnant if I want<br>Possible side effects<br>Don't think PrEP works<br>High costs involved<br>Make me feel responsible or ashamed<br>Inconvenient to take pills daily<br>Using other prev'n method (note method)<br>Faithful to one trusted partner<br>Not currently sexually active<br>Other (specify) ______________	Pos 1 / Neg 2 / Neutral 8 (each row)	
Q560	*Which people's views were/are important in encouraging or discouraging you to use PrEP?*<br><br>Instructions as for last question.<br><br>For partner/friends' views - if mentioned spontaneously, ask for the reason.	(Pos / Neg / Neutral / Probed)<br>Religious leaders (religious beliefs)<br>Parents' or family elders' approval/views<br>Partner(s) will think I have HIV<br>Partner's views (other)<br>Friends / community think I have HIV<br>Friends / community views (other)<br>Other (specify) ______________	Pos 1 / Neg 2 / Neutral 8 (each row)	
Q561	*How far away is it from where you live to the nearest place where PrEP services are provided?*	Distance in kms	kms	
Q562	*By how much do you think PrEP reduces a person's risk of getting HIV infection?*<br>Ask for or convert response to a percent.	Percent reduction	%	
Q563	*Are many of your friends (or their partners) using PrEP?*	Yes<br>No<br>Don't know	1<br>2<br>98	

For peer review only - http://bmjopen.bmj.com/site/about/guidelines.xhtml

REF.	QUESTIONS & FILTERS	CODING CATEGORIES			SKIP TO
Q564	*If you have or had a daughter (or sister) who is a teenager or a young woman and she started having sex before getting married, do you think it would be a good thing for her to use PrEP?*		Teen-ager	Young woman	
		Yes	1	1	
		No	2	2	
		Don't know	98	98	
Q565	*If your sexual partner wanted to use PrEP, would you agree to him or her doing this?*		Non-reg	Regular	
		Yes	1	1	
		No	2	2	
	<u>Ask first for non-regular partners (if any).</u>	Don't know	98	98	
	<u>Then for regular partners.</u>	NA	99	99	
Q566**	*How often have you used a male condom when you had sexual intercourse with your REGULAR partner or partners in the last 2 weeks?*	Every time	1		
		Most times	2		
		Occasionally	3		
		Never	4		
	<u>Emphasise asking about male condoms only here.</u>	NA - no regular partner	99		
Q567**	*How often have you used a male condom when you had sexual intercourse with NON-REGULAR partners in the last 2 weeks?*	Every time	1		- Q569 if 'every time' for reg & non-regs
		Most times	2		
		Occasionally	3		
		Never	4		
		NA - no non-regular partners	99		
Q568	*Why did you not use male condoms on some occasions when you had sex in the last 2 weeks?*	High costs	1		
		Judgemental staff / stigma	2		
		Lack of privacy / confidentiality	3		
		Limited times when accessible	4		
		Distance / travel difficulties to access	5		
		Sexual partner(s) disapproved	6		
		Parents / elders / friends disapproved	7		
		Condoms reduce pleasure of sex	8		
		Condoms not effective	9		
	______________________	Using different prevention method (specify metho	10		
		Faithful to one trusted partner	11		
	______________________	Other (specify)	12		
		None	96		
		Don't know	98		
Q569	*Has a male condom ever broken when you were using it?*	Yes	1		
		No	2		- Q571
		NA - not using male condoms	99		- Q571
Q570	*What do you do when male condoms break?*	Replace the condom with a new one	1		
		Continue without replacing the condom	2		
	______________________	Other (specify)	99		
Q571	*After you started using male condoms, did you increase or decrease your number of sexual partners?*	Increase	1		
		Decrease	2		
		No change	8		
		Never used male condoms	99		

Q572 *Please tell me whether you strongly disagree, disagree, neither agree nor disagree, agree or strongly agree with the following statements …*

Statement	SD	D	N	A	SA
(1) I am confident I can use male condoms if I wanted to	1	2	3	4	5
(2) I am confident I can use male condoms even if I have to use them every time	1	2	3	4	5
(3) I am confident I can use male condoms even if my partner dislikes/disapproves	1	2	3	4	5
(4) I am confident I can use male condoms even if I'm drunk or have taken drugs	1	2	3	4	5
(5) I am confident I can use male condoms even if my friends disapprove	1	2	3	4	5
(6) I am confident I can use male condoms even if my parents and family elders disapprove	1	2	3	4	5

REF.	QUESTIONS & FILTERS	CODING CATEGORIES		SKIP TO
Q573	*Have you ever received instructions or counselling on how to use male condoms?*	Yes	1	
		No	2	
Q574	*Are you able to discuss using male condoms with your REGULAR partner?*	Yes - already done so	1	
		Yes - not done already but can do this	2	
		No	3	
		Never had sex	97	- Q580
		No regular partner	99	- Q577
Q575	*Would / does your regular partner disapprove if you use male condoms?*	Yes	1	
		No	2	
		Don't know	98	
Q576	*If your regular partner doesn't want to use male condoms, are you able to refuse to have sex with him/her?*	Yes - already done so	1	
		Yes - not done already but can do this	2	
		No	3	
		Don't know	98	
Q577	*Are you able to discuss using male condoms with your NON-REGULAR partner(s)?*	Yes - already done so	1	
		Yes - not done already but can do this	2	
		No	3	
		No non-regular partners	99	- Q580

For peer review only

For peer review only - http://bmjopen.bmj.com/site/about/guidelines.xhtml

INDIVIDUAL QUESTIONNAIRE: **HIV PREVENTION METHODS** *Q. No:* ______

REF.	QUESTIONS & FILTERS	CODING CATEGORIES		SKIP TO
Q578	*Would / do your non-regular partners disapprove if you use male condoms?*	Yes<br>No<br>Don't know	1<br>2<br>98	
Q579	*If a non-regular partner doesn't want to use male condoms, are you able to refuse to have sex with him/her?*	Yes - already done so<br>Yes - not done already but can do this<br>No	1<br>2<br>3	
Q580**	*If / when you want to use male condoms, do you know a place where someone like you can easily get them?*	Yes<br>No	1<br>2	
Q581*	*If / when you want to use male condoms yourself, how easy is it for you to access them?*<br><br>Ask for an answer on a scale of 5 - from 1 (very easy) to 5 (very difficult).	Very easy<br>Easy<br>Neither easy nor difficult<br>Difficult<br>Very difficult	1<br>2<br>3<br>4<br>5	

REF.	QUESTIONS & FILTERS	CODING CATEGORIES	Yes	No	DK
Q582	*Do you feel able to access male condoms from the following places?*<br><br>Ask each in turn.	Health clinic	1	2	98
		Community-based distributor	1	2	98
		Bars or beer halls	1	2	98
		Shops	1	2	98
		Your sexual partner(s)	1	2	98
		Friends	1	2	98
		Other (specify)	1	2	98

REF.	QUESTIONS & FILTERS	CODING CATEGORIES	Spont	Probed
Q583	*What factors make it impractical or unsuitable for someone like you to access male condoms?*<br><br>Ask first without probing and then probe for factors that are not mentioned spontaneously.	High costs		
		Lack of privacy / confidentiality		
		Embarrassed to go / ask		
		Limited opening hours		
		Distance / travel difficulties		
		Other (specify)		

REF.	QUESTIONS & FILTERS	CODING CATEGORIES		SKIP TO
Q584**	*Do you want to use male condoms with your regular partner(s) if they were freely accessible to you?*<br>Stress: no right or wrong answer.	Yes<br>No<br>Not sure<br>NA - no regular partner	1<br>2<br>98<br>99	 <br><br>- Q587
Q585*	*How definitely do you want to use male condoms with your regular partner(s) if they were freely accessible to you?*<br>Ask for an answer on a scale of 5 - from 1 (definitely) to 5 (definitely not).	Definitely<br>Probably<br>No opinion<br>Probably not<br>Definitely not	1<br>2<br>3<br>4<br>5	
Q586	*Do you plan to use male condoms the next time you have sex with your regular partner?*	Yes<br>No<br>Don't know	1<br>2<br>98	
Q587**	*Do you want to use male condoms with your non-regular partner(s) if they were freely accessible to you?*<br>Stress: no right or wrong answer.	Yes<br>No<br>Not sure<br>NA - no non-regular partner(s)	1<br>2<br>98<br>99	 <br><br>- Q590
Q588*	*How definitely do you want to use male condoms with your non-regular partner(s) if they were freely accessible to you?*<br>Ask for an answer on a scale of 5 - from 1 (definitely) to 5 (definitely not).	Definitely<br>Probably<br>No opinion<br>Probably not<br>Definitely not	1<br>2<br>3<br>4<br>5	
Q589	*Do you plan to use male condoms the next time you have sex with a non-regular partner?*<br>Stress: no right or wrong answer.	Yes<br>No<br>Don't know	1<br>2<br>98	

REF.	QUESTIONS & FILTERS	CODING CATEGORIES	Pos	Neg	Neutral	Probed
Q590	*What factors were/are important in encouraging or discouraging you to use male condoms?*<br><br>For each factor given, ask if this was a positive (encouraging) or negative (discouraging) factor.<br><br>Ask first without probing and then probe for factors that are not mentioned spontaneously.<br>Tick probed box to indicate probed responses.	HIV/STI protection	1	2	8	
		HIV/STI protection (for my partner(s))	1	2	8	
		Method of HIV prevention I can control	1	2	8	
		Reduce risk of pregnancy (self/partner)	1	2	8	
		Own sexual pleasure (better/worse)	1	2	8	
		Partner's sexual pleasure (better/worse)	1	2	8	
		Make me feel responsible or ashamed	1	2	8	
		Faithful to one trusted partner	1	2	8	
		Using other prev'n method (note method)	1	2	8	
		Not currently sexually active	1	2	8	
		Other (specify) ____________	1	2	8	

For peer review only

For peer review only - http://bmjopen.bmj.com/site/about/guidelines.xhtml

INDIVIDUAL QUESTIONNAIRE: **HIV PREVENTION METHODS** *Q. No:*

REF.	QUESTIONS & FILTERS	CODING CATEGORIES					SKIP TO
Q591	*Which people's views were/are important in encouraging or discouraging you to use male condoms?*<br><br>Instructions as for last question.<br><br>For partner/friends' views - if mentioned spontaneously, ask for the reason.		Pos	Neg	Neutral	Probed	
		Religious leaders (religious beliefs)	1	2	8		
		Parents' or family elders' approval/views	1	2	8		
		Partner(s) will think I have HIV	1	2	8		
		Partner will think I have other partners	1	2	8		
		Partner's views (other)	1	2	8		
		Friends / community think I have HIV	1	2	8		
		Friends / community views (other)	1	2	8		
		Other (specify) _____________	1	2	8		
Q592	*By how much do you think male condoms reduce a person's risk of getting HIV infection?*<br>Ask for or convert response to a percent.	Percent reduction				%	
Q593	*How far away is it from where you live to the nearest place where male condoms can be obtained?*	Distance in kms				kms	
Q594	*Are many of your friends (or their partners) using male condoms?*	Yes			1		
		No			2		
		Don't know			98		
Q595	*In what circumstances do you think it is acceptable for a husband and wife to use condoms?*				Yes	No	
		Always			1	2	
		If one of them is HIV+			1	2	
		If one spouse has other partners			1	2	
		If one spouse has an STD			1	2	
		To avoid pregnancy			1	2	
		Other (specify)			1	2	
Q596	*If you have or had a daughter (or sister) who was a teenager or young woman and she started having sex before getting married, do you think it would be a good thing for her to use male condoms with her partner?*			Teen-ager		Young woman	
		Yes		1		1	
		No		2		2	
		Don't know		98		98	
	"Now I would like to ask you some questions about FEMALE condoms …"						
Q597**	*How often have you used a female condom when you had sexual intercourse with your REGULAR partner or partners in the last 2 weeks?*	Every time			1		
		Most times			2		
		Occasionally			3		
		Never			4		
		NA - no regular partner			99		
		Not heard of female condoms			97		- Q59929
Q598**	*How often have you used a female condom when you had sexual intercourse with NON-REGULAR partners in the last 2 weeks?*	Every time			1		
		Most times			2		
		Occasionally			3		
		Never			4		
		NA - no non-regular partners			99		
Q599	*Why did you not use female condoms on some occasions when you had sex in the last 2 weeks?*	High costs			1		
		Difficult to use			2		
		Judgemental staff / stigma			3		
		Lack of privacy / confidentiality			4		
		Limited times when accessible			5		
		Distance / travel difficulties			6		
		Sexual partner(s) disapproved			7		
		Parents / elders / friends disapproved			8		
		Female condoms reduce pleasure of sex			9		
		Female condoms not effective			10		
		Using different prevention method (specify metho			11		
		Faithful to one trusted partner			12		
		Other (specify)			13		
		None			96		
		Don't know			98		
Q59901	*Has a female condom ever broken when you were using it?*	Yes			1		
		No			2		- Q59903
		NA - not using male condoms			99		- Q59903
Q59902	*What do you do when female condoms break?*	Replace the condom with a new one			1		
		Continue without replacing the condom			2		
		Other (specify)			99		
Q59903	*After you started using female condoms, did you increase or decrease your number of sexual partners?*	Increase			1		
		Decrease			2		
		No change			8		
		Never used female condoms			99		

FORM B

For peer review only

For peer review only - http://bmjopen.bmj.com/site/about/guidelines.xhtml

INDIVIDUAL QUESTIONNAIRE: **HIV PREVENTION METHODS** *Q. No:*

REF.	QUESTIONS & FILTERS	CODING CATEGORIES		SKIP TO
Q59904	*Please tell me whether you strongly disagree, disagree, neither agree nor disagree, agree or strongly agree with the following statements …*			

Statement	SD	D	N	A	SA
(1) I am confident I can use female condoms if I wanted to	1	2	3	4	5
(2) I am confident I can use female condoms even if I have to use them every time	1	2	3	4	5
(3) I am confident I can use female condoms even if my partner dislikes/disapproves	1	2	3	4	5
(4) I am confident I can use female condoms even if I'm drunk or have taken drugs	1	2	3	4	5
(5) I am confident I can use female condoms even if my friends disapprove of this	1	2	3	4	5
(6) I am confident I can use female condoms even if my parents and family elders disapprove	1	2	3	4	5

REF.	QUESTIONS & FILTERS	CODING CATEGORIES	CODE	SKIP TO
Q59905	*Have you ever received instructions or counselling on how to use female condoms?*	Yes	1	
		No	2	
Q59906	*Are you able to discuss using female condoms with your REGULAR partner?*	Yes - already done so	1	
		Yes - not done already but can do this	2	
		No	3	
		No regular partner	8	- Q59909
		Never had sex	99	- Q59912
Q59907	*Would / does your regular partner disapprove if you use female condoms?*	Yes	1	
		No	2	
		Don't know	98	
Q59908	*If your regular partner doesn't want to use female condoms, are you able to refuse to have sex with him/her?*	Yes - already done so	1	
		Yes - not done already but can do this	2	
		No	3	
		Don't know	98	
Q59909	*Are you able to discuss using female condoms with your NON-REGULAR partner(s)?*	Yes - already done so	1	
		Yes - not done already but can do this	2	
		No	3	
		No non-regular partners	99	- Q59912
Q59910	*Would / do your non-regular partners disapprove if you use female condoms?*	Yes	1	
		No	2	
		Don't know	98	
Q59911	*If a non-regular partner doesn't want to use female condoms, are you able to refuse to have sex with him/her?*	Yes - already done so	1	
		Yes - not done already but can do this	2	
		No	3	
Q59912*	*If / when you want to use female condoms, do you know a place where someone like you can easily get them?*	Yes	1	
		No	2	
Q59913*	*If / when you want to use female condoms yourself, how easy is it for you to access them?* Ask for an answer on a scale of 5 - from 1 (very easy) to 5 (very difficult).	Very easy	1	
		Easy	2	
		Neither easy nor difficult	3	
		Difficult	4	
		Very difficult	5	

REF.	QUESTIONS & FILTERS	CODING CATEGORIES	Yes	No	DK
Q59914	*Do you feel able to access female condoms from the following places?* Ask each in turn.	Health clinic	1	2	98
		Community-based distributor	1	2	98
		Bars or beer halls	1	2	98
		Shops	1	2	98
		Your sexual partner(s)	1	2	98
		Friends	1	2	98
		Other (specify)	1	2	98

REF.	QUESTIONS & FILTERS	CODING CATEGORIES	Spont	Probed
Q59915*	*What factors make it impractical or unsuitable for someone like you to access female condoms?* Ask first without probing and then probe for factors that are not mentioned spontaneously.	High costs		
		Lack of privacy / confidentiality		
		Embarrassed to go / ask		
		Limited opening hours		
		Distance / travel difficulties		
		Other (specify)		

REF.	QUESTIONS & FILTERS	CODING CATEGORIES	CODE	SKIP TO
Q59916	*Do you want to use female condoms with your regular partner(s) if they were freely accessible to you?*	Yes	1	
		No	2	
		Not sure	98	
		NA - no regular partner	99	- Q59919
Q59917*	*How definitely do you want to use female condoms with your regular partner(s) if they were freely accessible to you?* Ask for an answer on a scale of 5 - from 1 (definitely) to 5 (definitely not).	Definitely	1	
		Probably	2	
		No opinion	3	
		Probably not	4	
		Definitely not	5	

For peer review only

INDIVIDUAL QUESTIONNAIRE: **HIV PREVENTION METHODS** *Q. No:*

REF.	QUESTIONS & FILTERS	CODING CATEGORIES		SKIP TO
Q599 18*	*Do you plan to use female condoms the next time you have sex with your regular partner?*	Yes	1	
		No	2	
		Don't know	98	
Q599 19*	*Do you want to use female condoms with your non-regular partner(s) if they were freely accessible to you?*	Yes	1	
		No	2	
		Not sure	98	
		NA - no non-regular partner	99	- Q599 22
Q599 20*	*How definitely do you want to use female condoms with your non-regular partner(s) if they were freely accessible to you?* Ask for an answer on a scale of 5 - from 1 (definitely) to 5 (definitely not).	Definitely	1	
		Probably	2	
		No opinion	3	
		Probably not	4	
		Definitely not	5	
Q599 21*	*Do you plan to use female condoms the next time you have sex with a non-regular partner?*	Yes	1	
		No	2	
		Don't know	98	

Q599 22 *What factors were/are important in encouraging or discouraging you to use female condoms?*

For each factor given, ask if this was a positive (encouraging) or negative (discouraging) factor.

Ask first without probing and then probe for factors that are not mentioned spontaneously.

Tick probed box to indicate probed responses.

Factor	Pos	Neg	Neutral	Probed
HIV/STI protection	1	2	8	
HIV/STI protection (for my partner(s))	1	2	8	
Method of HIV prevention I can control	1	2	8	
Reduce risk of pregnancy (self/partner)	1	2	8	
Own sexual pleasure (better/worse)	1	2	8	
Partner's sexual pleasure (better/worse)	1	2	8	
Make me feel responsible or ashamed	1	2	8	
Difficult to use	1	2	8	
Fear of losing condom inside vagina	1	2	8	
Faithful to one trusted partner	1	2	8	
Using other prev'n method (note method)	1	2	8	
Not currently sexually active	1	2	8	
Other (specify) ______________	1	2	8	

Q599 23 *Which people's views were/are important in encouraging or discouraging you to use female condoms?*

Instructions as for last question.

For partner/friends' views - if mentioned spontaneously, ask for the reason.

View	Pos	Neg	Neutral	Probed
Religious leaders (religious beliefs)	1	2	8	
Parents' or family elders' approval/views	1	2	8	
Partner(s) will think I have HIV	1	2	8	
Partner will think I have other partners	1	2	8	
Partner's views (other)	1	2	8	
Friends / community think I have HIV	1	2	8	
Friends / community views (other)	1	2	8	
Other (specify) ______________	1	2	8	

Q599 23 *What are the main reasons that would/did*
Q599 24 *By how much do you think female condoms reduce a person's risk of getting HIV infection?*
Ask for or convert response to a percent.

Percent reduction — Spont / Probed: ____ %

Q599 25 *How far away is it from where you live to the nearest place where female condoms can be obtained?*

Distance in kms: ____ kms

REF.	QUESTIONS & FILTERS	CODING CATEGORIES		SKIP TO
Q599 26	*Are many of your friends (or their partners) using female condoms?*	Yes	1	
		No	2	
		Don't know	98	

Q599 27 *In what circumstances do you think it is acceptable for a husband and wife to use female condoms?*

Circumstance	Yes	No
Always	1	2
If one of them is HIV+	1	2
If one spouse has other partners	1	2
If one spouse has an STD	1	2
To avoid pregnancy	1	2
Other (specify)	1	2

Q599 28* *If you have or had a daughter (or sister) who was teenager or young woman and she started having sex before getting married, do you think it would be a good thing for her to use female condoms with her partner?*

	Teen-ager	Young woman
Yes	1	1
No	2	2
Don't know	98	98

REF.	QUESTIONS & FILTERS	CODING CATEGORIES		SKIP TO
Q599 29*	*Are you currently sticking to one regular sexual partner or not having sex?* Stress: no right or wrong answer.	Yes - one regular partner	1	
		Yes - not having sex	2	
		Neither	3	- Q599 31
Q599 30	*For how long have you been sticking to one regular partner or not having sex?*	Number or months or years	yrs / mths	

For peer review only

INDIVIDUAL QUESTIONNAIRE: **HIV PREVENTION METHODS** *Q. No:*

REF.	QUESTIONS & FILTERS	CODING CATEGORIES		SKIP TO
Q59931	*Why are you currently having more than one sexual partner?*	Multiple wives	1	
		Sex worker	2	
		Financial or material benefits / reduce poverty	3	
		Sexual pleasure	4	
		Emotional attachment (love)	5	
		Social status (impress peers etc.)	6	
		Other (specify)	7	
		Don't know	98	
Q59932*	*Please tell me whether you strongly disagree, disagree, neither agree nor disagree, agree or strongly agree with the following statements …*	SD / D / N / A / SA		
	(1) I am confident I can stick to one partner if I wanted to.	1 / 2 / 3 / 4 / 5		
	(2) I am confident I can stick to one partner even if my friends encourage me to have other partners	1 / 2 / 3 / 4 / 5		
	(3) I am confident I can stick to one partner even if I lose financial or material benefits	1 / 2 / 3 / 4 / 5		
Q59933	*If you wanted to stick to one partner, how easy would this be for you to do?*	Very easy	1	
		Easy	2	
		Neither easy nor difficult	3	
	Ask for an answer on a scale of 5 - from 1 (very easy) to 5 (very difficult).	Difficult	4	
		Very difficult	5	
Q59934	*What factors make it impractical or unsuitable for someone like you to stick to one partner?*		Spont / Probed	
		Multiple wives		
		Poverty		
	Ask first without probing and then probe for factors that are not mentioned spontaneously.	Threat of violence from partners		
		Peer pressure		
		Other (specify)		
Q59935	*How definitely do you want to stick to one partner in the near future?*	Definitely	1	
		Probably	2	
		No opinion	3	
	Ask for an answer on a scale of 5 - from 1 (definitely) to 5 (definitely not).	Probably not	4	
		Definitely not	5	
Q59936	*Do you plan to start sticking to one partner or to stop having sex?*	Yes - one regular partner	1	
		Yes - stop having sex	2	
		Neither	3	- Q59938
	Stress: no right or wrong answer.	Don't know	98	- Q59938
Q59937	*How soon do you plan to make this change?*	Number of months	mths	
Q59938	*What are the main reasons that would/did motivate you to stop having sex?*		Spont / Probed	
		Reduce risk of HIV & STDs (self/partner)		
		Reduce risk of pregnancy (self or partner)		
	Ask first without probing and then probe for factors that are not mentioned spontaneously.	Would feel a responsible person		
		Poor health		
		Children (daughters) now sexually active		
		Feel too tired / old		
		Other (specify)		
Q59939	*What are the main reasons that would/did discourage you from stopping having sex?*		Spont / Probed	
		Enjoy having sex		
		Desire/need to have (more) children		
		Partner would think I have HIV		
	Ask first without probing and then probe for factors that are not mentioned spontaneously.	Partner would leave me		
		Threat of violence from partner(s)		
		Friends would disapprove		
		Parents / family elders would disapprove		
		Community members would disapprove		
		Other (specify)		
Q59940	*What are the main reasons that would/did motivate you to stick to one partner?*		Spont / Probed	
		Reduce risk of HIV infection & STDs		
		Would feel a responsible person		
	Ask first without probing and then probe for factors that are not mentioned spontaneously.	Regular partner would approve		
		Parents / family elders would approve		
		Friends would approve		
		Other (specify)		
Q59941	*What are the main reasons that would/did discourage you from sticking to one partner?*		Spont / Probed	
		Sexual pleasure from having multiple partners		
		Loss of financial or material benefits		
	Ask first without probing and then probe for factors that are not mentioned spontaneously.	Loss of reputation / status in the community		
		Already married to multiple wives		
		Other (specify)		
Q59942	*Have you ever received training or counselling to reduce your number of sexual partners?*	Yes - organisation code (Q504)	1	
		No	2	
Q59943	*Would your sexual partner(s) accept it if you left him/her/them?*	Yes	1	
		No	2	

For peer review only

INDIVIDUAL QUESTIONNAIRE: **HIV AWARENESS & SOCIAL NORMS** **Q. No:**

REF.	QUESTIONS & FILTERS	CODING CATEGORIES		SKIP TO
Q601	*"Now I would like to ask you some questions about HIV and AIDS ..."*			
Q602	*Please tell me all the ways that an adult can get HIV infection?*<br>Tick 1 for each way mentioned spontaneously.<br>*Are there any other ways?*<br>Then proceed down the column, reading the description of each possible way not mentioned spontaneously. Make entries in "probed" column as follows:<br>1/11 Yes, HIV can be transmitted this way (spont / yes when probed)<br>2/12 No, HIV cannot be transmitted this way (spont / yes when probed)<br>98 Don't know	Sex with a person with HIV/AIDS<br>Touching a person with AIDS<br>Mosquito bites<br>Blood transfusion<br>Injection with a dirty needle<br>Sharing utensils with person with HIV/AIDS<br>Ritual scarification<br>Punishment from God<br>Other (specify) ______	Spont / Probed	
Q603	*Do you know of any factors which are likely to INCREASE the chances that a person will get HIV infection?*<br>Ask in same way as Q602.<br>Codes as in Q602.	Sex with a prostitute<br>Many sex partners<br>Not being circumcised (for men)<br>Other STDs present<br>Using condoms<br>Witchcraft or spiritual curse<br>Other (specify)	Spont / Probed	
Q604	*What are the ways in which an infant or child could have become infected with HIV?*<br>Ask in same way as Q602.<br>Codes as in Q602.	At birth - if mother infected<br>Witchcraft or spiritual curse<br>Mosquito bites<br>Injection with a dirty needle<br>Breastfed by infected woman not on ART<br>Blood transfusion<br>From an infected father<br>Other (specify)	Spont / Probed	
Q605	*Are ALL babies born to women who have HIV born with the infection?*	Yes<br>No<br>Don't know	1<br>2<br>98	
Q606	*Can all people infected with HIV be identified by looking at them?*	Yes<br>No<br>Don't know	1<br>2<br>98	
Q607	*Would you be willing to take care of a family member with AIDS?*	Yes<br>No<br>Don't know	1<br>2<br>98	- Q609
Q608	*Why would you not be willing to take care of a family member with AIDS?*	Not enough time<br>Too few resources<br>Not enough space<br>Too young<br>Not experienced enough<br>Unwell myself<br>Frightened of being exposed to HIV<br>Reluctant to be associated with people living with HIV<br>Other (specify)	1<br>2<br>3<br>4<br>5<br>6<br>7<br>8<br><br>12	
Q609	*How long does it usually take for a person infected with HIV to develop symptoms if they are not on ART?*	Number of years or months<br>Don't know	yrs / mths<br>998	
Q610	*How many people do you know who either died from HIV/AIDS or have the disease now?*	Number (>0)<br>Doesn't know of any	mths<br>998	
Q611	*Of these people, how many live(d) in your household; in the same village/town; and how many live(d) somewhere else?*<br>Enter numbers of people in each category.	Household<br>Village / town<br>Somewhere else<br>Check that these sum to same as in Q510.		

For peer review only

For peer review only - http://bmjopen.bmj.com/site/about/guidelines.xhtml

INDIVIDUAL QUESTIONNAIRE: **HIV AWARENESS & SOCIAL NORMS** ***Q. No:***

REF.	QUESTIONS & FILTERS	CODING CATEGORIES		SKIP TO
Q612	*What was your relationship to each of these people?* Enter numbers of people in each category.	Spouse/partner<br>Father or mother<br>Son or daughter<br>Other relative<br>Friend or neighbour<br>Work colleague<br>Someone else		
Q613	*Did you help to take care of any of these people on a daily basis?*	Yes<br>No	1<br>2	
Q614	*What are the chances that you are infected with HIV now?*	Certain / almost certain<br>High<br>Moderate<br>Small<br>None	1<br>2<br>3<br>4<br>5	
Q615	*What are the chances that you will become infected with HIV in the next 12 months if you are not infected now but continue with your current behaviour?*	Certain / almost certain<br>High<br>Moderate<br>Small<br>None	1<br>2<br>3<br>4<br>5	 <br><br><br>- Q617
Q616	*Why do you think you might become infected?*	Has multiple sex partners now<br>Not using condoms<br>Regular partner has other partners<br>Future partner may have other partners<br>Partner may be infected with HIV<br>Many friends/relatives dying of HIV/AIDS<br>Caring for an infected person<br>Other (specify)	1<br>2<br>3<br>4<br>5<br>6<br>7<br>8	

Q617 *For the following statements, please tell me whether you strongly disagree, disagree, neither agree nor disagree, agree or strongly agree …*

	SD	D	N	A	SA
(1) I can get HIV even if I only have sex with one	1	2	3	4	5
(2) I have never done anything that could have exposed me to HIV	1	2	3	4	5
(3) I would rather die a violent death (e.g. gunshot or car accident) than from HIV	1	2	3	4	5
(4) I feel vulnerable to HIV infection	1	2	3	4	5
(5) I am less likely to get HIV than my friends	1	2	3	4	5
(6) I'm worried that one of my current sexual partners may give me HIV	1	2	3	4	5
(7) I think about my chances of getting HIV whenever I have a new sexual partner	1	2	3	4	5
(8) Getting (a woman) pregnant when you don't want to is worse than getting infected with	1	2	3	4	5

REF.	QUESTIONS & FILTERS	CODING CATEGORIES		SKIP TO
Q618	*How often do you worry about getting infected with HIV?*	Never<br>Rarely<br>Sometimes<br>Often<br>Almost all the time	1<br>2<br>3<br>4<br>5	
Q619	*Who do you think is more likely to be infected with HIV in Manicaland: a 15-19 year-old man or a 25-29 year-old man?*	15-19 year-old man<br>25-29 year-old man	1<br>2	
Q620	*Who do you think is more likely to be infected with HIV in Manicaland: a 15-19 year-old woman or a 25-29 year-old man?*	15-19 year-old woman<br>25-29 year-old man	1<br>2	
Q621	*Would you buy fresh vegetables from a shopkeeper or vendor if you knew this person had HIV?*	Yes<br>No<br>Don't know/not sure/it depends	1<br>2<br>98	
Q622	*Do you think that children living with HIV should be able to attend school with children who are HIV negative?*	Yes<br>No<br>Don't know/not sure/it depends	1<br>2<br>98	
Q623	*If you thought your spouse/regular partner was having sexual intercourse with (a) casual partner(s) without using condoms, could you persuade him/her to stop?*	Yes<br>No<br>No regular partner<br>Don't know	1<br>2<br>96<br>98	

For peer review only

INDIVIDUAL QUESTIONNAIRE: **HIV AWARENESS & SOCIAL NORMS** **Q. No:**

REF.	QUESTIONS & FILTERS	CODING CATEGORIES		SKIP TO
Q624	*Do you think there are things you can do which will prevent you from becoming infected with HIV in the future?*	Yes<br>No<br>Don't know<br>NA - already infected	1<br>2<br>98<br>99	
Q625	*Which of these statements do you agree with?*<br>(1) Men have a sex drive that needs to be satisfied<br>(2) A man will lose respect if he admits to being sick<br>(3) Men who take sick children to the hospital, or cook at home should be proud of what they do<br>(4) Men are strong and therefore less likely to need a doctor<br>(5) Men are always ready for sex<br>(6) A man should not go with his partner for antenatal check-ups at the local clinic<br>(7) If a man is sick, he should not let others see he is in pain<br>(8) It is appropriate for a woman to be the primary breadwinner of a household<br>(9) A real man enjoys a bit of risk taking now and then<br>(10) Men should have had several sexual partners before they get married<br>Read out each in turn.		Agree / Disagree<br>1 2<br>1 2<br>1 2<br><br>1 2<br>1 2<br>1 2<br>1 2<br>1 2<br>1 2<br>1 2	
Q626	*Which of these statements would you say was true?*<br>(1) Minor illnesses can be fought off if you don't give in to it<br>(2) Men feel comfortable going to the hospital and have no problems seeking help<br>(3) There is no need to go and see a doctor unless you are very ill<br>(4) A man should make sure that he can recognise the early signs of an STI<br>(5) It is important for a man living with HIV to be on anti-retroviral therapy<br>(6) A man who goes to the hospital is considered weak<br>(7) A man gain respect if he goes for regular health checks<br>(8) Men get embarrassed if a brother is found to be HIV positive<br>Read out each in turn.		True / Not<br>1 2<br>1 2<br>1 2<br>1 2<br>1 2<br>1 2<br>1 2<br>1 2	
Q627	*Which of these statements do you agree with?*<br>(1) It is a good idea to make condoms available for young people in schools<br>(2) If I have / had a teenage daughter, I would tell her about condoms<br>(3) If I have / had a teenage daughter, I would tell her about PrEP<br>(4) If I have / had a teenage daughter and thought she might be having sex, I would encourage her to use PrEP and condoms<br>(5) If I have / had a teenage daughter and told her not to have sex until she gets married, she would comply<br>(6) If a young woman is married and her husband has HIV, she should use PrEP or condoms<br>(7) If I have / had a teenage daughter and she had sex before marriage, I would be OK with this<br>(8) Many young women have sex before marriage these days<br>(9) If I have / had a teenage son, I would encourage him to have medical circumcision<br>(10) I pay/get paid for sex because my friends do and because they encourage me		Agree / Disagree<br>1 2<br>1 2<br>1 2<br>1 2<br>1 2<br>1 2<br>1 2<br>1 2<br>1 2<br>1 2<br>1 2	
Q628	*Do you think a lot of young people are still getting infected with HIV these days?*	Yes<br>No	1<br>2	- Q630 if<br>- male
Q629	*Have you experienced any of the following from a male intimate partner in the past 12 months?*<br>(1) Slapped you or threw something at you that could hurt you<br>(2) Pushed or shoved you<br>(3) Hit you with a fist or something else that could hurt you<br>(4) Kicked or dragged you or beat you up<br>(5) Choked or burnt you<br>(6) Threatened or used a gun, knife or other weapon against you<br>(7) Physically forced you to have sexual intercourse against your will<br>(8) Forced you to do something sexual she found degrading or humiliating<br>(9) Made you afraid of what would happen if you did not have sexual intercourse		Yes / No<br>1 2<br>1 2<br>1 2<br>1 2<br>1 2<br>1 2<br>1 2<br>1 2<br>1 2	
Q630	*Imagine that we flipped a fair coin 10 times. The first 9 times, the coin comes down 'heads'. What are the chances that the 10th flip will also be 'heads'?*	Percentage chance	%	

For peer review only

REF.	QUESTIONS & FILTERS	CODING CATEGORIES	SKIP TO
Q631	I will ask you several questions about the chance or likelihood that certain events are going to happen. There are 10 beans in the cup. I would like you to choose some beans out of these 10 beans and put them in the plate to express what you think the likelihood or chance is of a specific event happening. One bean represents one chance out of 10. If you do not put any beans in the plate, it means you are sure that the event will NOT happen. As you add beans, it means that you think the likelihood that the event happens increases. For example, if you put one or two beans, it means you think the event is not likely to happen but it is still possible. If you pick 5 beans, it means that it is just as likely it happens as it does not happen (fifty-fifty). If you pick 6 beans, it means the event is slightly more likely to happen than not to happen. If you put 10 beans in the plate, it means you are sure the event will happen. There is no right or wrong answer, I just want to know what you think.<br><br>Let me give you an example. Imagine that we are playing draughts. Say, when asked about the chance that you will win, you put 7 beans in the plate. This means that you believe you would win 7 out of 10 games on average if we play for a long time.		
Q632	Report for each question the NUMBER OF BEANS put in the PLATE.<br>After each question, replace the beans in the cup (unless otherwise noted).<br><br>For questions Q633 to Q635: If respondent puts 10 (or 0) beans, prompt:<br>*"Are you sure that this event will almost certainly (not) happen?"*<br><br>CIRCLE 1 in column P if you prompted the respondent, and report the final answer only.		
Q633	*A baby in your community will die before his or her first birthday?*	# beans on plate [ ] prompt (0 or 10) 1	
Q634	*You will go to the market at least once within the next 2 days?*<br><br>LEAVE BEANS IN PLATE.	[ ] 1	
Q635	*You will go to the market at least once within the next 2 weeks?*	[ ] 1	Q637 if beans added
Q636	*Remember, as time goes by, you may find more time to go to the market. Therefore, you should have added beans to the plate. Let me ask you again. Now, add beans in the plate so that the number of beans in the plate reflects how likely you think it is that you will go to the market at least once within the next 2 weeks?*	[ ] 1	
Q637	*Please pick the number of beans that reflects how likely you think it is that …*<br><br>*You are infected with HIV now?*	# beans on plate [ ]	Q640 if unmarried
Q638	*Your spouse is infected with HIV now?*	[ ]	
Q639	*You will use a condom next time you have sex with your spouse / long-term partner?*<br><br>If no regular sexual partner(s), write '99'.	[ ]	
Q640	*You will use a condom next time you have sex with a non-regular partner?*<br><br>If no non-regular sexual partner(s), write '99'.	[ ]	Q701 if '99'
Q641	*Your current / most recent non-regular sexual partner is infected with HIV now?*<br><br>If no non-regular sexual partner(s), write '99'.	[ ]	
Q642	*You will use a condom next time you have sex with a non-regular partner?*<br><br>If no non-regular sexual partner(s), write '99'.	[ ]	
Q643	*You will use a condom next time you have sex with someone for the first time?*	[ ]	

For peer review only - http://bmjopen.bmj.com/site/about/guidelines.xhtml

INDIVIDUAL QUESTIONNAIRE: **HEALTH & ACCESS TO TREATMENT** **Q. No:**

REF.	QUESTIONS & FILTERS	CODING CATEGORIES	SKIP TO
Q701	*Has a medical doctor or nurse ever diagnosed you with the following conditions?*	(see grid below)	

	Yes	No	DK	Year	Tx	Pd
High blood pressure	1	2	98			
Diabetes	1	2	98			
High cholesterol	1	2	98			
Kidney diseases	1	2	98			
Heart diseases (incl. stroke)	1	2	98			
Depression	1	2	98			
Osteoporosis	1	2	98			
Tuberculosis	1	2	98			
Malaria	1	2	98			
Schistosomiasis	1	2	98			
Hepatitis B	1	2	98			
Hepatitis C	1	2	98			
Pregnancy/delivery complications	1	2	98			
Human papilloma virus	1	2	98			
Cancer (specify type)	1	2	98			
Genital herpes	1	2	98			
HIV infection	1	2	98			

REF.	QUESTIONS & FILTERS	CODING CATEGORIES	CODE	SKIP TO
Q702	For EACH condition mentioned in Q701, record the year of first diagnosis in the 4th column above.			
Q703	For EACH condition mentioned in Q701, record details of the institution providing the treatment in the 5th column (Tx) using the following codes:	Public sector healthcare provider	1	
		Church Mission healthcare provider	2	
		Private sector provider	3	
		NGO or academic research institution	4	
		Other	8	
		Not currently receiving treatment	99	
Q704	For EACH condition mentioned in Q701, record details of who paid for the medical fees and the costs of the drugs in the 6th column (Pd) using the following codes:	Private medical insurance only	1	
		Private medical insurance partly	2	
		Respondent and/or family	3	
		Other	8	
		Service was free	99	
Q705	*How long is it since you last experienced an illness?*	Days/weeks	days / weeks	
		More than one year ago	97	- Q714
Q706	*What was the main symptom of the illness?*	Fever - malaria (incl. cerebral)	1	
		Fever - non-malaria	2	
		Sickness/vomiting	3	
		Diarrhoea/weight loss	4	
		Swollen lymph nodes	5	
		Skin complaints/rashes	6	
		Genital conditions: incl. STDs	7	
		Flu/pneumonia	8	
		Accident/wound	9	
		Tuberculosis	10	
		Other (specify)	12	
Q707	*How long was it between the time when you first noticed symptoms and when you first sought help for this illness?*	Days and weeks	days / weeks	- Q709
		Did not seek assistance	97	
Q708	*What is the main reason you have not sought treatment?*	Costs of treatment/travel too high	1	- Q714
		Too little time to travel / wait for treatment	2	- Q714
		Do not feel that anyone can help	3	- Q714
		Able to cope myself / illness not serious	4	- Q714
		Fear of stigma	5	- Q714
		Other (specify)	8	- Q714

REF.	QUESTIONS & FILTERS	CODING CATEGORIES	Yes	No	SKIP TO
Q709	*Which of the following people did you visit or consult for this illness?* Read the options and tick all that apply.	Medical doctor or nurse	1	2	- Q711
		N'anga	1	2	- if not
		Faith healer	1	2	- n'anga or
		Other (specify)	1	2	- faith hlr
Q710	*Did you seek assistance from the n'anga (or faith healer) for any of these reasons?* Read the options and tick all that apply.	Clinic treatment not effective	1	2	- Q714
		Spiritual cure needed	1	2	- if did
		Witchcraft suspected	1	2	- not also
		Holy water/bath or prayer	1	2	- visit
		More confidential than clinic	1	2	- hospital
		More convenient to visit than clinic	1	2	- or
		Less expensive to visit than clinic	1	2	- clinic
		Other (specify)	1	2	-

REF.	QUESTIONS & FILTERS	CODING CATEGORIES	CODE	SKIP TO
Q711	*How long did it take you to travel from your home to the clinic/hospital?*	Minutes<br>Convert hours to minutes if necessary.	mins	
Q712	*How long did you have to wait before you were seen by a doctor or nurse?*	Minutes<br>Convert hours to minutes if necessary.	mins	

For peer review only

For peer review only - http://bmjopen.bmj.com/site/about/guidelines.xhtml

REF.	QUESTIONS & FILTERS	CODING CATEGORIES		SKIP TO
Q713	*Did you receive an HIV test as part of your treatment for this illness?*	Yes<br>No or don't know	1<br>2	
Q714	For men:<br>*Some men experience white, transparent or yellowish discharge from the penis, which might not cause discomfort or may be accompanied by mild burning paid on urination. In the last 12 months, have you had these symptoms?*<br>For women:<br>*Some women experience an unusual discharge from the vagina or pain in the lower stomach. In the last 12 months, have you had these symptoms?*	Yes - discharge & pain (both)<br>Yes - discharge only<br>Yes - pain only<br>No<br>Don't know<br>Clarify which & tick boxes as appropriate.	1<br>2<br>3<br>4<br>98	
Q715	*Some (wo)men experience sores in the genital area. During the last 12 months, have you noticed any such sores?*	Yes<br>No<br>Don't know	1<br>2<br>98	- Q717<br>- if not had<br>- symptoms
Q716	*What happened when you were treated at the health centre for these symptoms?*<br>Read the options and tick all that apply.	(Yes / No)<br>Not applicable - did not get treated at a clinic<br>Physical symptoms were inspected<br>Samples taken & tests done<br>HIV test was offered<br>Counseling provided<br>Free condoms provided<br>Symptoms went away after treatment	Yes / No<br>99<br>1 / 2<br>1 / 2<br>1 / 2<br>1 / 2<br>1 / 2<br>1 / 2	
Q717	*On how many different occasions have you had an HIV test and received the results: (i) in your lifetime; and (ii) in the last 3 years?*<br>Stress that these questions do *not* include self-tests.	Lifetime<br>Last 3 years		- If "0" go to Q725
Q718	*How long is it since you last had an HIV test?*	Period	mths / yrs	
Q719	*Did you receive counselling before you agreed to have the test?*<br>Explain what is meant by counselling.	Yes<br>No	1<br>2	
Q720	*Was the result of this HIV test positive?*<br>Stress that do not have to answer these questions but information is confidential.	Yes<br>No<br>Don't know<br>Prefers not to say	1<br>2<br>98<br>99	
Q721	*After the test, did you receive counselling and referrals for the following HIV prevention methods?*<br>Read the options and tick all that apply.<br>Don't ask about VMMC or PrEP if respondent is HIV-positive.	VMMC<br>PrEP<br>Male condoms<br>Female condoms<br>Being faithful to one partner<br>Not having sex<br>Treatment as prevention	Counselling (Yes 1 / No 2); Referrals (Yes 1 / No 2) for each	
Q722	*What is the name of the organisation that provided this HIV testing service?*	PSI - New Start<br>FACT<br>FHI360<br>Hospital/clinic (ANC/PMTCT)<br>Hospital/clinic (other)<br>Other (specify)<br>Don't know	1<br>2<br>3<br>4<br>5<br>8<br>98	
Q723	*After the HIV test, did you:*<br>(1) Use condoms more or less than before?<br>(2) Start having more or fewer sexual partners?		More 1 / Same 2 / Less 3<br>1 / 2 / 3<br>1 / 2 / 3	
Q724	*Did you join a post-test club or a group for people living with HIV and, if so, are you still a member?*<br>If HIV test result was +ve, go to Q737.	Post-test club<br>PLWHA<br>Neither	Still 1 / Was 2 / Never 3<br>1 / 2 / 3<br>1 / 2 / 3<br>8	
Q725	*Please tell me whether you strongly disagree, disagree, neither agree nor disagree, agree or strongly agree with the following statements …*<br>(1) I am confident I can get tested for HIV if I wanted to<br>(2) I am confident I can get tested for HIV even if the healthworker cannot keep the results confidential<br>(3) I am confident I can get tested for HIV even if my partner disapproves<br>(4) I am confident I can get tested for HIV even if I am afraid to be HIV+<br>(5) I am confident I can get tested for HIV even if I was stigmatised in my community if I was known or suspected to be HIV+		SD 1 / D 2 / N 3 / A 4 / SA 5 (for each statement)	

For peer review only

For peer review only - http://bmjopen.bmj.com/site/about/guidelines.xhtml

INDIVIDUAL QUESTIONNAIRE: **HEALTH & ACCESS TO TREATMENT** **Q. No:**

REF.	QUESTIONS & FILTERS	CODING CATEGORIES		SKIP TO
Q726	*Are you able to discuss HIV testing with your partner?*	Yes - already done so<br>Yes - not done already but can do this<br>No<br>No current partner	1<br>2<br>3<br>99	
Q727	*Would your partner disapprove if you got tested for HIV?*	Yes<br>No<br>Don't know	1<br>2<br>98	
Q728	*Would your partner come with you to get tested for HIV?*	Yes - already done so<br>Yes - not done already but can do this<br>No<br>Don't know	1<br>2<br>3<br>98	
Q729	*If / when you want to have an(other) HIV test, do you know a place where someone like you can easily get it?*	Yes<br>No	1<br>2	
Q730	*If / when you want to have an(other) HIV test yourself, how easy is it for you to access the service?*<br><br>Ask for an answer on a scale of 5 - from 1 (very easy) to 5 (very difficult).	Very easy<br>Easy<br>Neither easy nor difficult<br>Difficult<br>Very difficult<br>Don't know	1<br>2<br>3<br>4<br>5<br>98	
Q731	*What factors make it difficult or unsuitable for someone like you to get access to HIV testing services?*<br><br>Ask first without probing and then probe for factors that are not mentioned spontaneously.	High costs<br>Judgemental staff<br>Lack of privacy or confidentiality<br>Limited opening hours<br>Distance / travel difficulties<br>Not appropriate for me to go there<br>Other (specify)	Spont Probed	
Q732	*How far away is it from where you live to the nearest place where HIV tests are provided?*	Distance in kms	kms	
Q733	*Do you want to have an HIV test if it was freely accessible to you?*	Yes<br>No<br>Not sure	1<br>2<br>8	
Q734	*How definitely do you want to have an HIV test if it was freely accessible to you?*<br><br>Ask for an answer on a scale of 5 - from 1 (definitely) to 5 (definitely not).	Definitely<br>Probably<br>No opinion<br>Probably not<br>Definitely not	1<br>2<br>3<br>4<br>5	
Q735	*Do you plan to have an(other) HIV test?*	Yes<br>No<br>Don't know	1<br>2<br>98	 - Q737<br>- Q737
Q736	*How soon do you plan to have an(other) HIV test?*	Number of months	mths	
Q737	*What are the main reasons that woud/did motivate you to go for an HIV test?*<br><br>Ask first without probing and then probe for factors that are not mentioned spontaneously.	To access HIV treatment if I need it<br>Wanted re-assurance not infected<br>Avoid infecting partner(s)<br>Partner or child sick or died<br>Past risky behaviour<br>Partner's risky behaviour<br>Many friends going for HIV tests<br>Encouraged by family<br>Reassure partner<br>Contemplating marriage<br>Contemplating having a child<br>Prevent mother-to-child infection<br>Spouse/partner tested HIV+<br>Doctor/nurse suggested it<br>Life planning<br>Other (specify)	Spont Probed	
Q738	*What are the main reasons that would/did discourage you from having an HIV test?*<br><br>Ask first without probing and then probe for factors that are not mentioned spontaneously.	Psychological effects<br>Stigma & discrimination<br>Possible divorce/separation<br>Job loss<br>Positive result accelerates death<br>Fear - of being HIV+<br>Fear - of partner violence<br>Lack of confidentiality<br>Too expensive<br>Other (specify)	Spont Probed	

For peer review only

For peer review only - http://bmjopen.bmj.com/site/about/guidelines.xhtml

INDIVIDUAL QUESTIONNAIRE: **HEALTH & ACCESS TO TREATMENT** **Q. No:**

REF.	QUESTIONS & FILTERS	CODING CATEGORIES		SKIP TO
Q739	*If you have or had a daughter or son who is a teenager or a young adult and she/he started having sex, do you think it would be a good thing for her/him to have an HIV test?*	Yes<br>No<br>Don't know	Teen / YA<br>1 / 1<br>2 / 2<br>98 / 98	
Q740	Explain that there are HIV tests that you can do yourself.<br>*How long is it since you last had an HIV self-test?*	Period<br>Never had a self-test<br>Not heard of HIV self-tests	mths / yrs<br>97<br>99	 - Q743<br>- Q744
Q741	*Was the result of this HIV self-test positive or negative?*<br>Stress that do not have to answer these questions but information is confidential.	Positive<br>Negative<br>Don't know<br>Prefers not to say	1<br>2<br>98<br>99	
Q742	*Did you get this result confirmed at a health facility or other HIV testing facility?*<br>Stress that do not have to answer these questions but information is confidential.	Yes - result confirmed<br>No - went but result was different<br>No - didn't go for confirmation test<br>Prefers not to say	1<br>2<br>3<br>99	
Q743	*If / when you want to do an HIV self-test, do you know a place where someone like you can easily get a test kit?*	Yes<br>No	1<br>2	
Q744	*Have you heard of antiretroviral therapy (ART) - the drugs that prevent HIV from causing AIDS?*	Yes<br>No	1<br>2	 - Q748
Q745	*How far is it from here to the nearest place where these drugs can be obtained?*	Distance<br>Don't know a place	kms<br>98	 - Q748
Q746	*What type of place is this?*<br>"Roadside" here means a tarred road.<br>Record name of place..	Large town or city<br>Small town<br>Growth point<br>Commercial estate/mine<br>Roadside business centre<br>Rural business centre<br>Communal / resettlement area	1<br>2<br>3<br>4<br>5<br>6<br>7	
Q747	*How long does it take to travel from your home to this place?*<br>Convert hours to minutes if necessary.	Minutes	mins	
Q748	*Have you ever taken ART yourself?*<br>Check that not taking something that could be ART	Yes<br>No	1<br>2	- Q750
Q749	*What is the main reason you have not started taking these drugs?*	Costs too high<br>Not available locally<br>Not permitted by church<br>Side effects<br>Not needed: in good health<br>Not needed: HIV-<br>Other (specify)<br>Don't know	1<br>2<br>3<br>4<br>5<br>6<br>8<br>98	- Q801<br>- Q801<br>- Q801<br>- Q801<br>- Q801<br>- Q801<br>- Q801<br>- Q801
Q750	*How long is it since you first took these drugs?*	Period	weeks / years	
Q751	*What motivated you to start taking ART?*	HIV+: unwell<br>HIV+: well but wanted to remain healthy<br>Other (specify)	1<br>2<br>8	
Q752	*Have you stopped taking the drugs?*	Yes<br>No	1<br>2	 - Q754
Q753	*Why have you stopped taking the drugs?*	Costs too high<br>Not available locally<br>Side effects<br>Not needed: in good health<br>Other (specify)<br>Don't know	1<br>2<br>3<br>4<br>8<br>98	- Q756<br>- Q756<br>- Q756<br>- Q756<br>- Q756<br>- Q756
Q754	*Are there particular times when you take the drugs?*	All the time<br>When feeling unwell<br>When can afford or paid for<br>Other (specify)	1<br>2<br>3<br>8	
Q755	*Do you sometimes forget to take the drugs?*	Never<br>Occasionally<br>Quite often	1<br>2<br>3	
Q756	*How often have you taken ART in the last month?*	Every day<br>Most days<br>Occasionally<br>Not at all	1<br>2<br>3<br>4	

For peer review only

For peer review only - http://bmjopen.bmj.com/site/about/guidelines.xhtml

INDIVIDUAL QUESTIONNAIRE:	HEALTH & ACCESS TO TREATMENT	*Q. No:*

REF.	QUESTIONS & FILTERS	CODING CATEGORIES	SKIP TO
Q757	***After you started taking the drugs, did you:***<br>**(1) Start having more or fewer sexual partners?**<br>**(2) Use condoms more or less than before?**	More / Same / Less<br>1 2 3<br>1 2 3	
Q758	***Have you experienced any unpleasant side effects since you started the treatment?***	**Yes** 1<br>**No** 2	
Q759	***Have you (also) been receiving treatment from a traditional healer or faith healer for your HIV infection?***	**Traditional healer** 1<br>**Faith healer** 2<br>**Neither** 3	

- 28 - FORM B

For peer review only

INDIVIDUAL QUESTIONNAIRE: **FERTILITY HISTORIES** *Q. No:*

REF.	QUESTIONS & FILTERS	CODING CATEGORIES			SKIP TO
**Q801**	*Now I would like to talk to you about pregnancy and childbirth. Have you ever given birth (fathered a child)*?*	Yes<br>No	1<br>2		 - Q806
**Q802**	*Do you have any sons or daughters who are living with you now?*	Yes<br>No	1<br>2		 - Q804
**Q803**	*How many sons live with you? How many daughters live with you?*	Sons at home<br>Daughters at home			
**Q804**	*Do you have any sons or daughters who are alive but do not live with you?*	Yes<br>No	1<br>2		 - Q806
**Q805**	*How many sons are alive but do not live with you? How many daughters are alive but do not live with you?*	Sons elsewhere<br>Daughters elsewhere			
**Q806**	*Have you ever given birth to (or fathered) a boy or girl who was born alive but later died?*<br>**If no, probe:**<br>*Any (other) boy or girl who cried or showed any sign of life but only survived a few hours or days?*	Yes<br>No	1<br>2		 - Q808
**Q807**	*How many boys have died in this way? And how many girls died in this way?*	Boys who died<br>Girls who died			
**Q808**	**Sum answers to Q803, Q805 and Q807. Enter total.**	Total			
**Q809**	*In total, then, how many live births have you had (fathered)?*<br>**Compare response with total in Q708. If numbers are different, probe and correct Q801-Q808, as necessary.**				- for men, go to Q901
**Q810**	*Have you had a live birth in the last year?*	Yes<br>No	1<br>2		
**Q811**	*Have you been pregnant in the last year?*	Yes<br>No	1<br>2		

***Note:** **For male respondents use question wordings indicated in brackets.**

For peer review only - http://bmjopen.bmj.com/site/about/guidelines.xhtml

INDIVIDUAL QUESTIONNAIRE: RECENT PREGNANCY HISTORY *Q. No:* ____

REF.	QUESTIONS & FILTERS	CODING CATEGORIES		SKIP TO
Q901	*Are you pregnant at the moment?*	Yes<br>No	1<br>2	 - Q903
Q902	*How many months pregnant do you think you are?*<br>Ask for best estimate.	Number of months	☐	- Q905
Q903	*Have you ever had a pregnancy that ended in a live birth, miscarriage or still-birth?*	Yes<br>No<br>Not sure	1<br>2<br>98	 - Q1001<br>- Q1001
Q904	*When did your most recent such pregnancy end?*<br>Stress: including miscarriages.		mnth / yr	
Q905	*At the time you became pregnant, did you want to become pregnant then, did you want to wait until later, or did you not want to become pregnant at all?*	Then<br>Later<br>Not at all	1<br>2<br>3	
Q906	*Did you see anyone for antenatal care during this pregnancy?*	Yes<br>No	1<br>2	 - Q923
Q907	*After how many months of the pregnancy did you first go for an antenatal check-up?*	Months	☐	
Q908	*Did you have an HIV test while attending for ANC check-ups for this pregnancy or did you already know your status?*<br>If knew HIV+, ask if she was on ART.	Yes<br>No: already knew HIV+ - on ART<br>No: already knew HIV+ - not on ART<br>No: did not want	1<br>2<br>3<br>4	
Q909	*Did your husband (or pregnancy partner) also attend for PMTCT with you and have an HIV test during this pregnancy?*	Yes<br>No: already knew HIV+<br>No: did not want	1<br>2<br>3	
Q910	*Did YOU have HIV infection at the time of this pregnancy?*<br>Stress confidential but voluntary.	Yes<br>No<br>Don't know<br>No response	1<br>2<br>8<br>9	 - Q917<br>- Q917<br>- Q917
Q911	*Did the clinic provide you with any counselling on family planning for people with HIV?*	Yes<br>No<br>Don't recall	1<br>2<br>8	
Q912	*Did you receive any treatment to take YOURSELF to prevent the baby from getting infected? If so, what type?*<br>Stress that this is treatment taken by the mother herself.	Yes: already on ART<br>Yes: initiated on ART during pregnancy<br>Yes: other (specify) ________<br>No	1<br>2<br>3<br>4	- Q915<br><br>- Q915<br>- Q915
Q913	*Are you still taking ART now?*	Yes<br>No	1<br>2	- Q915
Q914	*Why did you decide to stop taking ART?* ________	No longer breastfeeding the baby<br>Healthy so no need for ART<br>Side effects<br>Other (specify)<br>Don't know	1<br>2<br>3<br><br>98	

FORM B

For peer review only - http://bmjopen.bmj.com/site/about/guidelines.xhtml

INDIVIDUAL QUESTIONNAIRE: **RECENT PREGNANCY HISTORY** **Q. No:**

REF.	QUESTIONS & FILTERS	CODING CATEGORIES		SKIP TO
Q915	*Did the baby receive any of these forms of treatment to prevent him/her from getting infected with HIV?*		Y N	
		ART (i.e. continuous)	1 2	
		NVP syrup	1 2	
		Other (specify)	1 2	
Q916	*Did you or the baby ever stop or miss taking the treatment you were given?*	Yes	1	
		No	2	
		Baby died before completed treatment	3	
Q917	*Was the baby delivered at a clinic/hospital or at home?*	Clinic	1	
		Home	2	
Q918	*Did this pregnancy end in a miscarriage, abortion or stillbirth?*	Yes	1	
		No	2	- Q920
Q919	*How many months pregnant were you when this pregnancy ended?*		mths	
Q920	*Did the baby ever have an HIV test?*	Yes: infected	1	
		Yes: uninfected	2	
	If yes, ask for result.	No	3	
	Stress confidential but voluntary.	DK or rather not say	8	
Q921	*Has the baby been initiated on ART?*	Yes: started & still taking	1	
		Yes: started but since stopped	2	
		No	3	
		Don't know	8	
Q922	*Has the baby received medical male circumcision?*	Yes: medical circumcision	1	- Q927
		No: but traditional circumcision	2	
		No: not circumcised at all (yet)	3	
		No: baby is a girl	99	- Q927
Q923	*Would you have liked the baby to have received medical male circumcision?*	Yes	1	
		No	2	
Q924	*Were medical male circumcision services for infants available in your area at the time this baby was born?*	Yes	1	
		No	2	
Q925	*Is the baby still alive?*	Yes	1	- Q929
		No	2	
Q926	*How old was the baby when he/she passed away?*	Convert to months.	mths	
		<1m = '0' months.		
Q927	*Did you ever feed this baby at the breast?*	Yes	1	
		No	2	- Q1001
Q928	*Are you still breastfeeding?*	Yes	1	- Q1001
		No	2	
		Child has died	99	
Q929	*For how long did you breastfeed this baby?*			
	Exclusively? (i.e. no liquids or solids)	Months		
	In total?	Months		
	If total > 6 mths, go to Q1001.			
Q930	*Why did you not breastfeed this baby (for longer)?*	Baby sick or died	1	
		Mother sick	2	
		Risk of HIV	3	
		Pregnant	4	
		Resumed sex	5	
		Other	8	

FORM B

For peer review only - http://bmjopen.bmj.com/site/about/guidelines.xhtml

INDIVIDUAL QUESTIONNAIRE: **CONCLUSION** ***Q. No:***

REF.	QUESTIONS & FILTERS			
Q1001	*For how much of the last 3 years have you and your regular partner been using a method of contraception?*	None	1	- Q1004
		Some of the time	2	
		Most/all of the time	3	
		Not sure	98	
Q1002	*Which of these methods were the main methods you used?* Ask about each method in turn.		Y N	
		Pill	1 2	
		Injections	1 2	
		Condoms	1 2	
		Femidoms	1 2	
		Sterilization	1 2	
		Safe period	1 2	
		Withdrawal	1 2	
		Other (specify)	1 2	
Q1003	*What were your main reasons for wanting to delay or prevent another pregnancy?* Probe for other reasons, but do not prompt.	Enough children	1	
		Birth spacing	2	
		Child HIV+ risk	3	
		Child orphan risk	4	
		Mother HIV+: accelerate AIDS	5	
		Not yet ready to have children	6	
		Other (specify)	8	
Q1004	Record current time.	Hour and minutes	hr mins	
Q1005	Record contact details for follow-up interviews if required.	Cell number (1)		
		Cell number (2)		
Q1006	*What are your views of the value of this research?*	Useful	1	
		Do not see the point	2	
		No opinion	98	
Q1007	Record respondent's comments and your own observations in the space below. Remind respondent of arrangements for the BE procedures (where relevant) and for HIV testing.			

RESPONDENT'S COMMENTS:

On the research?

Further HIV prevention, care and support activities needed?

ENUMERATOR'S OBSERVATIONS:

For peer review only - http://bmjopen.bmj.com/site/about/guidelines.xhtml

STROBE Statement—Checklist of items that should be included in reports of ***cross-sectional studies***

	Item No	Recommendation	Page No
**Title and abstract**	1	(*a*) Indicate the study's design with a commonly used term in the title or the abstract	1
		(*b*) Provide in the abstract an informative and balanced summary of what was done and what was found	2
**Introduction**			
Background/rationale	2	Explain the scientific background and rationale for the investigation being reported	4
Objectives	3	State specific objectives, including any prespecified hypotheses	4
**Methods**			
Study design	4	Present key elements of study design early in the paper	5-6
Setting	5	Describe the setting, locations, and relevant dates, including periods of recruitment, exposure, follow-up, and data collection	5
Participants	6	(*a*) Give the eligibility criteria, and the sources and methods of selection of participants	5
Variables	7	Clearly define all outcomes, exposures, predictors, potential confounders, and effect modifiers. Give diagnostic criteria, if applicable	6-8
Data sources/ measurement	8*	For each variable of interest, give sources of data and details of methods of assessment (measurement). Describe comparability of assessment methods if there is more than one group	6-8
Bias	9	Describe any efforts to address potential sources of bias	5-9
Study size	10	Explain how the study size was arrived at	5-6
Quantitative variables	11	Explain how quantitative variables were handled in the analyses. If applicable, describe which groupings were chosen and why	5-8
Statistical methods	12	(*a*) Describe all statistical methods, including those used to control for confounding	6-8
		(*b*) Describe any methods used to examine subgroups and interactions	6-8
		(*c*) Explain how missing data were addressed	6-8
		(*d*) If applicable, describe analytical methods taking account of sampling strategy	6
		(*e*) Describe any sensitivity analyses	NA
**Results**			
Participants	13*	(a) Report numbers of individuals at each stage of study—eg numbers potentially eligible, examined for eligibility, confirmed eligible, included in the study, completing follow-up, and analysed	9 and Fig. 1
		(b) Give reasons for non-participation at each stage	9 and Fig. 1
		(c) Consider use of a flow diagram	Fig. 1
Descriptive data	14*	(a) Give characteristics of study participants (eg demographic, clinical, social) and information on exposures and potential confounders	9-11
		(b) Indicate number of participants with missing data for each variable of interest	10-11
Outcome data	15*	Report numbers of outcome events or summary measures	11-16

For peer review only - http://bmjopen.bmj.com/site/about/guidelines.xhtml

Main results	16	(*a*) Give unadjusted estimates and, if applicable, confounder-adjusted estimates and their precision (eg, 95% confidence interval). Make clear which confounders were adjusted for and why they were included	11-16
		(*b*) Report category boundaries when continuous variables were categorized	11-16
		(*c*) If relevant, consider translating estimates of relative risk into absolute risk for a meaningful time period	NA
Other analyses	17	Report other analyses done—eg analyses of subgroups and interactions, and sensitivity analyses	11-16 and Fig. 2
**Discussion**			
Key results	18	Summarise key results with reference to study objectives	16
Limitations	19	Discuss limitations of the study, taking into account sources of potential bias or imprecision. Discuss both direction and magnitude of any potential bias	18-19
Interpretation	20	Give a cautious overall interpretation of results considering objectives, limitations, multiplicity of analyses, results from similar studies, and other relevant evidence	16-17
Generalisability	21	Discuss the generalisability (external validity) of the study results	16-18
**Other information**			
Funding	22	Give the source of funding and the role of the funders for the present study and, if applicable, for the original study on which the present article is based	20

*Give information separately for exposed and unexposed groups.

Note: An Explanation and Elaboration article discusses each checklist item and gives methodological background and published examples of transparent reporting. The STROBE checklist is best used in conjunction with this article (freely available on the Web sites of PLoS Medicine at http://www.plosmedicine.org/, Annals of Internal Medicine at http://www.annals.org/, and Epidemiology at http://www.epidem.com/). Information on the STROBE Initiative is available at www.strobe-statement.org.

For peer review only

For peer review only - http://bmjopen.bmj.com/site/about/guidelines.xhtml

BMJ Open

Associations between HIV status and self-reported hypertension in a high HIV prevalence sub-Saharan African population: A cross-sectional study

Journal:	*BMJ Open*
Manuscript ID	bmjopen-2022-067327.R1
Article Type:	Original research
Date Submitted by the Author:	22-Nov-2022
Complete List of Authors:	Davis, Katherine ; Imperial College London, MRC Centre for Global Infectious Disease Analysis, Department of Infectious Disease Epidemiology<br>Moorhouse, Louisa ; Imperial College London, MRC Centre for Global Infectious Disease Analysis, Department of Infectious Disease Epidemiology<br>Maswera, Rufurwokuda; Biomedical Research and Training Institute<br>Mandizvidza, Phyllis; Biomedical Research and Training Institute<br>DADIRAI, Tawanda; Biomedical Research and Training Institute<br>Museka, Tafadzwa; Biomedical Research and Training Institute<br>Nyamukapa, Constance; Imperial College London, MRC Centre for Global Infectious Disease Analysis, Department of Infectious Disease Epidemiology; Biomedical Research and Training Institute<br>Smit, Mikaela ; Imperial College London<br>Gregson, Simon; Imperial College London, MRC Centre for Global Infectious Disease Analysis, Department of Infectious Disease Epidemiology
**Primary Subject Heading**:	Epidemiology
Secondary Subject Heading:	Global health, HIV/AIDS, Infectious diseases, Cardiovascular medicine, Public health
Keywords:	Hypertension < CARDIOLOGY, HIV & AIDS < INFECTIOUS DISEASES, EPIDEMIOLOGY, PUBLIC HEALTH

SCHOLARONE™
Manuscripts

I, the Submitting Author has the right to grant and does grant on behalf of all authors of the Work (as defined in the below author licence), an exclusive licence and/or a non-exclusive licence for contributions from authors who are: i) UK Crown employees; ii) where BMJ has agreed a CC-BY licence shall apply, and/or iii) in accordance with the terms applicable for US Federal Government officers or employees acting as part of their official duties; on a worldwide, perpetual, irrevocable, royalty-free basis to BMJ Publishing Group Ltd ("BMJ") its licensees and where the relevant Journal is co-owned by BMJ to the co-owners of the Journal, to publish the Work in this journal and any other BMJ products and to exploit all rights, as set out in our licence.

The Submitting Author accepts and understands that any supply made under these terms is made by BMJ to the Submitting Author unless you are acting as an employee on behalf of your employer or a postgraduate student of an affiliated institution which is paying any applicable article publishing charge ("APC") for Open Access articles. Where the Submitting Author wishes to make the Work available on an Open Access basis (and intends to pay the relevant APC), the terms of reuse of such Open Access shall be governed by a Creative Commons licence – details of these licences and which Creative Commons licence will apply to this Work are set out in our licence referred to above.

Other than as permitted in any relevant BMJ Author's Self Archiving Policies, I confirm this Work has not been accepted for publication elsewhere, is not being considered for publication elsewhere and does not duplicate material already published. I confirm all authors consent to publication of this Work and authorise the granting of this licence.

For peer review only

Associations between HIV status and self-reported hypertension in a high HIV prevalence sub-Saharan African population: A cross-sectional study

For peer review only

Katherine DAVIS, MRes[1], Louisa MOORHOUSE, MSc[1], Rufurwokuda MASWERA, BSw[2], Phyllis MANDIZVIDZA, SRN[2], Tawanda DADIRAI, BSc[2], Tafadzwa MUSEKA, MSc[2], Constance NYAMUKAPA, PhD[1,2], Mikaela SMIT, PhD[1], Simon GREGSON, DPhil[1,2]

1. MRC Centre for Global Infectious Disease Analysis, Department of Infectious Disease Epidemiology, Imperial College London, London, UK

2. Biomedical Research and Training Institute, Harare, Zimbabwe

Corresponding author/contact for reprints: Katherine Davis, Department of Infectious Disease Epidemiology, St Mary's Campus, Imperial College London, London, W2 1PG, UK, K.davis18@imperial.ac.uk, +44 (0)20 7594 5777.

Word count: 3,926/4,000

For peer review only

Abstract

Objectives: This study examined whether HIV status and antiretroviral therapy (ART)-exposure were associated with self-reported hypertension in Zimbabwe.

Design: Study data were taken from a cross-sectional, general population survey, which included HIV testing (July 2018 – December 2019).

Setting: The data were collected in Manicaland Province, Zimbabwe.

Participants: 9,780 people aged 15 years and above were included.

Outcome measure: Self-reported hypertension was the outcome measure. This was defined as reporting a previous diagnosis of hypertension by a doctor or nurse. After weighting of survey responses by age and sex using household census data, chi-squared tests and logistic regression were used to explore whether HIV status and ART-exposure were associated with self-reported hypertension.

Results: The weighted prevalence of self-reported hypertension was 13.6% (95% Confidence Interval: 12.9%-14.2%) and the weighted prevalence of HIV was 11.1% (10.4%-11.7%). In univariable analyses, there was no evidence of a difference in the weighted prevalence of self-reported hypertension between people living with HIV (PLHIV) and HIV-negative people (14.1%, 11.9%-16.3% vs 13.3%, 12.6%-14.0%; p=0.503) or between ART-exposed and ART-naive PLHIV (14.8%, 12.0%-17.7% vs 12.8%, 9.1%-16.4%, p=0.388). Adjusting for socio-demographic variables in logistic regression did not alter this finding (odds ratios: HIV status: 0.88, 0.70-1.10, p=0.261; ART-exposure: 0.83, 0.53-1.30, p=0.411).

Conclusions: Approximately one in seven PLHIV self-reported having hypertension, highlighting an important burden of disease. However, no associations were found between HIV status or ART-exposure and self-reported hypertension, suggesting that it will be valuable to focus on managing other risk factors for hypertension in this population. These findings should be fully accounted for as Zimbabwe re-orients its health system towards non-communicable disease control and management.

Keywords: HIV & AIDS, hypertension, epidemiology, public health

For peer review only

Strengths and limitations

- This is the first study to compare the prevalence of self-reported hypertension between people living with HIV and HIV-negative people in a non-clinical setting in Zimbabwe.
- We used data from a large population survey, which included a diverse range of men and women, and could be weighted to local census information, increasing the representativeness of our results.
- We focused on self-reported hypertension, so hypertensive people who were unaware of their status were not included in our prevalence estimates.
- Our study used cross-sectional data that excluded individuals in hospital settings and did not capture data on all risk factors for hypertension.

For peer review only

Introduction

Hypertension is the leading modifiable cause of death and disability worldwide.[1] In sub-Saharan Africa, the region most affected by infectious diseases such as HIV, the burden of hypertension is growing rapidly.[2] Yet despite these newly coinciding burdens, the relationship between HIV and hypertension is poorly understood.[3]

Various plausible mechanisms that could generate a difference in the prevalence of hypertension between people living with HIV (PLHIV) and HIV-negative people have been proposed. For example, chronic inflammation, increased microbial translocation, renal disease, and blood vessel damage resulting from long-term ART exposure could increase the prevalence of hypertension among PLHIV, as could higher levels of behavioural risk factors among PLHIV in some communities.[4–6] Conversely, possible reasons for a reduced hypertension burden among PLHIV include low blood pressure resulting from advanced HIV disease, better control of blood pressure due to additional healthcare support and lower levels of behavioural risk factors among PLHIV in some settings.[7–9] The conflicting outcomes of the proposed mechanisms mean that it is unclear whether any difference in hypertension prevalence by HIV status exists and in what direction it acts.

As a variety of physiological and treatment-related mechanisms by which HIV could raise or lower hypertension risk have been proposed, epidemiological studies are vital to furthering understanding.[5,7] Globally, there is some observational evidence that people living with HIV (PLHIV) might face a higher burden of hypertension.[4,6,10–12] However, the relationship appears to vary by region and it has been suggested that, in African countries, PLHIV may experience a lower prevalence of hypertension.[6] For example, in a Ugandan population, PLHIV were found to have 30% lower odds of hypertension than matched HIV-negative controls.[13] Detailed information about how the burden of hypertension differs by HIV status remains scarce across sub-Saharan Africa, constraining understanding of the association between HIV and hypertension; this is of particular concern as the relationship may differ between populations.[6] In Zimbabwe, only three studies have examined the burden of hypertension among PLHIV in the community, and all lacked a comparison group of HIV-negative people.[14–16] Just one of these studies examined the effect of antiretroviral therapy (ART) on hypertension, reporting that long-term use of ART was associated

For peer review only

with increased prevalence of hypertension, a finding which has not been replicated in this setting.[14]

Bridging the data gap will be crucial to supporting strategic decision-making about provision of preventative services and hypertension treatment. Currently, capacity to prevent and control hypertension in Zimbabwe is limited, but major changes are being planned to manage the rising burden.[14,17–19] Delivering these changes efficiently will require an understanding of the role of HIV status and ART-exposure in hypertension. This study aimed to contribute to that understanding, by examining whether HIV status and ART-exposure were associated with self-reported hypertension in Manicaland, East Zimbabwe.

Methods

Data

Study data were taken from the Manicaland General Population Open Cohort Study (Manicaland Study), a long-running, large-scale HIV sero-survey.[20,21] Each survey round of the cohort has involved testing participants for HIV and asking questions on topics including participants' sociodemographic characteristics, general health, and knowledge of HIV.[21] The most recent round, which ran between July 2018 and December 2019, is unique as it provides the first insight into the burden of hypertension and other non-communicable diseases. Consequently, cross-sectional data from the most recent round was used in this analysis (questionnaire in supplementary material).

Eight sites were included in the round: a subsistence farming area (Bonda Mission), a tea estate (Eastern Highlands), a forestry estate (Selbourne), two small towns (Nyazura and Nyanga), a roadside settlement (Watsomba) and two urban areas (Sakubva and Hobhouse). A team of Zimbabwean researchers led data collection at the sites, gathering survey responses on electronic tablets. Initially, households were enumerated in a census which included a brief questionnaire that asked about relationships between those in the household and household assets. After the census, more detailed data were collected through individual interviews. All women aged 15-24 years and

For peer review only

all men aged 15-29 years, as well as a random sample of two thirds of older adults, in enumerated households were eligible to complete the individual interview. The research team contacted eligible participants and interviewed them at central sites, such as health centres, or in their homes. If eligible people were unavailable or couldn't be contacted when first approached, at least two further attempts were made to reach them. The team aimed to achieve a sample size of approximately 9,500, in order to detect changes in the primary outcome measures of the Manicaland Study, which will be reported elsewhere.[22–24]

During the individual interview, participants provided a range of socio-demographic and health information. For example, they self-reported whether they had ever been diagnosed with hypertension by a doctor or nurse and whether they had ever taken ART.

In addition to responding to interview questions, participants were offered provider-initiated testing and counselling (PITC) for HIV and asked to provide a dried blood spot (DBS) specimen for laboratory HIV testing. PITC was performed by accredited members of the research team following the standard procedures and testing algorithm used in Ministry of Health and Child Care routine PITC services. DBS specimens were tested using the same algorithm at the Biomedical Research and Training Institute (BRTI) in Harare.

Funding limitations meant that blood pressure was not measured during the household census or individual interview.

The Manicaland Study was conducted with the understanding and informed consent of participants. Ethical approval was given by Imperial College Research Ethics Committee (17IC4160), the BRTI Institutional Review Board (AP140/2017) and the Medical Research Council of Zimbabwe (MRCZ/A/2243).

Weighting

The selection criteria for the individual interview, and the pattern of response and non-response, generated a young cohort, with more women than men. As a result, weights were used to adjust variable distributions to better match the population in the household census. First, census data

For peer review only

were analysed to calculate the proportion of the population in 5-year age groups between 15 and 74 years, as well as a 75+ year-old age-group, by sex. Iterative proportional fitting, also known as raking, was then used to generate weights that matched the age and sex proportions in the interview sample to those in the household census. Participant data were weighted for all further analyses; this approach meant that 42 people with missing age or sex information were excluded.

Association between HIV and self-reported hypertension

After weighting, prevalence estimates for self-reported hypertension and HIV were generated. Self-reported hypertension was defined as reporting ever having been diagnosed with hypertension by a doctor or nurse. HIV status was determined from PITC results; however, if a person opted out of PITC but consented to provide a DBS specimen, the DBS specimen was tested in the laboratory and this result was used instead. Consequently, our sample of PLHIV included both people who knew their status at the time of interview and people who did not. Prevalence estimates were calculated as the weighted number of people living with HIV or self-reporting hypertension, divided by the weighted total population. To compare the prevalence of self-reported hypertension between PLHIV and HIV-negative people, estimates for the prevalence of self-reported hypertension stratified by HIV status, age and sex were produced in the same manner. Age was grouped into six classes: '15-24', '25-34', '35-44', '45-54', '55-64' '65 and over'. Categories were chosen to reflect the Zimbabwean population distribution, in which most people are young, but a minority have reached old age. Crude comparisons of the prevalence of self-reported hypertension between PLHIV and HIV-negative people were made using Chi-squared tests, with Rao-Scott adjustment. These were performed both for the overall population and within age and sex strata.

Logistic regression models were employed to examine the relationship between HIV and self-reported hypertension, while controlling for available sociodemographic variables. All models were complete case analyses. Initially, a model was built including HIV, age, and sex. This was then developed into a fully adjusted model. First, a range of potential sociodemographic confounders were identified through the literature. Second, any potential confounders that were found to be associated with self-reported hypertension in models including age and sex, at a threshold p-value of 0.1, were added to the initial model. Lastly, the new model was refined by backward elimination of potential confounders, with a threshold p-value of 0.05. This created a final model for generating

For peer review only

adjusted estimates of the association between HIV status and self-reported hypertension.

The potential confounders that were considered for inclusion in the model were employment status, relationship status, education status, study site and wealth. Employment status was classified as 'employed or in education' or 'unemployed'; people who worked in the informal sector, doing petty trading or agricultural work, were categorised as 'employed or in education'. Relationship status was coded as 'married or in a long-term relationship', 'never been in a long-term relationship', 'separated or divorced' or 'widowed'. A long-term relationship was defined as a relationship of 12 months or more. Assessment of education status was based on the highest level of schooling that a person had obtained. Two categories were used: 'none or primary' and 'secondary or higher'; this reflected the fact that few people reported no schooling or education above secondary level. Wealth was classified using an index based on whether households owned a range of assets, as described by Lopman et al.[25] Assets included sellable items, such as bicycles and cattle, as well as fixed assets, such as water and electricity supplies. For each asset, binary and categorical responses were coded so that they lay between zero and one. For example, owning a bicycle was coded as one, whereas not owning a bicycle was coded as zero. The values for each asset were then summed, and the total was divided by the overall number of assets, to give an asset ownership score between zero (no assets owned) and one (all assets owned) for each household. Households were then grouped into five equally sized quintiles to create a household level wealth index with five categories. Each person in the dataset was then given the wealth index for their household.

The category representing the absence of a characteristic was chosen as the baseline for most variables. For variables where there was not an absent category, the category with the most participants, or that had been chosen as the baseline in other analyses of Manicaland Study data, was selected.[26,27]

Association between ART-exposure and self-reported hypertension

The association between ART-exposure in PLHIV and self-reported hypertension was evaluated using the approach to logistic regression described above. People who reported ever taking ART were defined as ART-exposed, allowing the prevalence of ART-exposure to be calculated as the

For peer review only

weighted number of PLHIV who were ART-exposed, divided by the weighted total number of PLHIV. Prevalence estimates for self-reported hypertension, stratified by ART-exposure, age, and sex, were then produced and a crude comparison between ART-exposed and ART-naive PLHIV was made using Chi-squared tests, with Rao-Scott adjustment. Finally, logistic regression models were developed, using the same model building strategy as was applied for the HIV status analysis, to produce adjusted estimates.

As a sensitivity analysis, we explored how the results changed when current ART usage, defined as reporting both having ever taken ART and not having stopped taking ART, was assessed instead of ART-exposure, using the approach described above. We also used the same methods to examine the association between adherent current ART usage and hypertension, with adherent current ART usage defined as reporting all the following: having ever taken ART, not having stopped taking ART, taking ART all the time, not forgetting to take ART, and having taken ART every day in the last month.

Software

Analyses were performed in R 4.0.3 using the survey package. [28,29]

Patient and public involvement

Members of the local community provided input into the research through small meetings held with community members at the beginning of the Manicaland Study and through providing feedback at meetings that were held to disseminate results in successive rounds of the survey. Feedback included recommendations for improving the recruitment and conduct of the study, as well as for reducing the burden of participation. In addition, the team worked closely with local guides (mainly community health workers) who also provided feedback as the study progressed. Input into the detail of the study design, research questions and outcome measures, was provided by local members of the research team too.

It is intended that the main results of this study will be disseminated to local stakeholders, research participants and other community members, as part of a wider plan to share results from the Manicaland Study. As in previous survey rounds, involvement of community members and local members of the research team will be key to the development of the dissemination strategy.

For peer review only

Results

During the census 7,610 households were contacted (Fig. 1). Of these, 7,550 households (99.2%) completed the questionnaire, making some of their members eligible for the individual interview. Among the 12,761 eligible people, 2,939 (23.0%) were unable to participate in, or did not consent to, the individual interview (reasons shown in Fig. 1). More of those who did not consent came from wealthier households (X^2=47.9, p<0.001) and urban sites (X^2=158.89, p<0.001). After exclusion of people without age or sex data, there were 9,780 participants in the study population.

Following weighting, there were marginally more women than men in the interviewed population (women: 52.2%; Table 1). The population was young; nearly a third were under 25 years old (31.0%). Unemployment was high (43.8%, 95% CI=42.9%-44.7%), yet most participants had secondary education or higher (75.7%, 74.9%-76.4%).

For peer review only

Table 1. Characteristics of study participants.

	Total Population (N=9,780)[1] Weighted proportion, % (95% Confidence Interval)	N[1]	People living with HIV (N=936)[1] Weighted proportion, % (95% Confidence Interval)	N[1]	HIV-negative people (N=8,378)[1] Weighted proportion, % (95% Confidence Interval)	N[1]
**Hypertension**						
Not reported	86.4% (85.7%-87.1%)	8,453	85.9% (83.7-88.1%)	791	86.7% (86.0-87.4%)	7,273
Reported	13.6% (12.9%-14.2%)	1,252	14.1% (11.9-16.3%)	139	13.3% (12.6-14.0%)	1,043
Missing	-	75	-	6	-	62
**HIV status**						
Negative	88.9% (88.3%-89.6%)	8,378	-	-	-	-
Positive	11.1% (10.4%-11.7%)	936	-	-	-	-
Missing	-	466	-	-	-	-
**ART-exposure[2]**						
ART-naïve	-	-	34.1% (31.0-37.2%)	323	-	-
ART-exposed	-	-	65.8% (62.8-69.0%)	612	-	-
Missing	-	-	-	1	-	-
**Sex[3]**						
Male	47.8%	4,064	43.6% (40.6%-46.7%)	331	48.2% (47.8%-48.7%)	3,544
Female	52.2%	5,716	56.4% (53.3%-59.4%)	605	51.8% (51.3%-52.2%)	4,834
**Age, years[3]**						
15-24	31.0%	3,923	7.1% (5.8%-8.5%)	97	34.7% (34.4%-35.1)	3,708
25-34	22.0%	1,988	19.8% (17.4%-22.2%)	191	22.2% (21.9%-22.6%)	1,689
35-44	18.5%	1,561	31.4% (28.6%-34.2%)	285	16.9% (16.5%-17.3%)	1,193
45-54	11.4%	1,022	27.1% (24.5%-29.7%)	253	9.4% (9.0%-9.7%)	704
55-64	7.2%	539	9.9% (7.8%-12.0%)	72	6.7% (6.4%-7.0%)	432
65+	9.8%	747	4.7% (3.3%-6.2%)	38	10.0% (9.7%-10.3%)	652
**Site**						
Bonda Mission (subsistence farming area)	15.9% (15.2%-16.7%)	1,497	14.4% (12.0%-16.8%)	129	16.7% (15.8%-17.5%)	1,343
Eastern Highlands (tea estate)	10.7% (10.0%-11.3%)	1,019	13.1% (10.9%-15.3%)	117	10.8% (10.1%-11.5%)	888
Selbourne (forestry estate)	14.4% (13.7%-15.1%)	1,377	15.6% (13.2%-18.0%)	145	14.8% (14.0%-15.6%)	1,210
Nyazura (town)	14.8% (14.1%-15.6%)	1,444	19.8% (17.1%-22.4%)	184	14.4% (13.7%-15.3%)	1,211
Nyanga (town)	11.4% (10.8%-12.0%)	1,157	6.8% (5.2%-8.4%)	69	10.0% (9.3%-10.6%)	866
Watsomba (roadside settlement)	17.4% (16.7%-18.2%)	1,694	16.1% (13.8%-18.5%)	154	18.1% (17.2%-18.9%)	1,496
Sakubva (urban)	7.8% (7.2%-8.3%)	789	8.6% (6.7%-10.4%)	82	7.5% (6.9%-8.1%)	654
Hobhouse (urban)	7.5% (7.0%-8.1%)	803	5.6% (4.2%-7.1%)	56	7.8% (7.2%-8.3%)	710

For peer review only

**Employment**						
Unemployed	43.8 (42.9%-44.7%)	4,322	47.6% (44.3%-50.8%)	472	43.4% (42.4%-44.4%)	4,722
Employed or in education	56.2 (55.3%-57.1%)	5,458	52.4% (49.2%-55.7%)	464	56.6% (55.6%-57.6%)	3,656
**Relationship status**						
Married or in a long-term relationship	59.5% (58.7%-60.3%)	5,389	60.4% (57.3%-63.6%)	536	59.4% (58.5%-60.3%)	4,594
Never been in a long-term relationship	25.9% (25.3%-26.5%)	3,033	10.2% (8.4%-12.1%)	111	28.3% (27.7%-29.0%)	2,821
Separated or divorced	6.8% (6.3%-7.4%)	663	13.2% (11.0%-15.3%)	131	5.8% (5.3%-6.4%)	483
Widowed	7.8% (7.3%-8.2%)	695	16.2% (13.8%-18.5%)	158	6.4% (5.9%-6.9%)	480
**Education**						
None or primary	22.9% (22.2%-23.7%)	2,023	27.1% (24.2%-30.1%)	244	22.4% (21.6%-23.2%)	1,685
Secondary or higher	77.1% (76.3%-77.8%)	7,595	72.9% (69.9%-75.8%)	663	77.6% (76.8%-78.4%)	6,566
Missing	-	162	-	29	-	127
**Asset-based wealth**						
1 (poorest)	19.9% (19.1%-20.7%)	1,908	22.3% (19.6%-25.1%)	210	20.4% (19.5%-21.3%)	1,667
2	19.7% (18.8%-20.5%)	1,866	20.8% (18.1%-23.5%)	192	19.8% (18.9%-20.7%)	1,609
3	20.7% (19.8%-21.5%)	1,977	22.3% (19.6%-25.1%)	207	20.9% (20.0%-21.8%)	1,710
4	19.9% (19.0%-20.7%)	1,963	20.7% (18.0%-23.4%)	191	19.6% (18.7%-20.4%)	1,662
5 (richest)	19.9% (19.1%-20.7%)	1,980	13.8% (11.6%-16.1%)	131	19.4% (18.5%-20.3%)	1,653
Missing	-	86	-	5	-	77

[1]Values are unweighted. In addition, HIV status was missing for some participants, so the unweighted Ns for People Living with HIV and HIV-negative people do not sum to the total population unweighted N.

[2]ART, antiretroviral therapy.

[3]Confidence intervals are not shown for variables used in weighting.

For peer review only

Association between HIV and self-reported hypertension

In this population, the weighted prevalence of self-reported hypertension was 13.6% (12.9%-14.2%) and the weighted prevalence of HIV was 11.1% (10.4%-11.7%). The prevalence of self-reported hypertension in PLHIV (14.1%, 11.9%-16.3%) was similar to the prevalence of self-reported hypertension in HIV-negative people in crude univariable analyses (13.3%, 12.6%-14.0%, F=0.4, p=0.503). The lack of evidence for a difference in univariable analyses remained when analysing the prevalence of self-reported hypertension within age and sex groups for most groups (Fig. 2, Supplementary table 1). However, there was statistical support for lower prevalence of self-reported hypertension in PLHIV than in HIV-negative people among women aged 45-54 (PLHIV:

For peer review only - http://bmjopen.bmj.com/site/about/guidelines.xhtml

20.2%, 13.8%-26.7%; HIV-negative: 30.1%, 25.7%-34.5%; F=5.4, p=0.021) and men aged over 65 (PLHIV: 6.7%, 0.0%-19.3%; HIV-negative: 31.2%, 25.3%-37.2%, F=4.1, p=0.043) in univariable analyses.

In the initial multivariable logistic regression analysis adjusted for age and sex, no evidence was found for an association between HIV and self-reported hypertension (odds ratio, OR=0.87, 95% CI=0.70-1.07, p=0.191). Introducing further confounders (employment, relationship status, wealth, and site) to generate a fully adjusted multivariable model did not alter this finding (0.88, 0.70-1.10, p=0.261; Table 2).

For peer review only

Table 2. HIV as a determinant of hypertension.

	Weighted hypertension prevalence, % (95% Confidence Interval)	Odds ratio adjusted for only age and sex (95% confidence interval)	P-value	Fully adjusted odds ratio (95% confidence interval)	P-value
**HIV status**			0.191		0.261
Negative	13.3% (12.6%-14.0%)	1		1	
Positive	14.1% (11.9%-16.3%)	0.87 (0.70-1.07)		0.88 (0.70-1.10)	
**Sex**			<0.001		<0.001
Male	6.7% (5.9%-7.6%)	1		1	
Female	19.8% (18.8%-20.8%)	4.37 (3.71-5.15)		4.48 (3.70-5.41)	
**Age, years**			<0.001		<0.001
15-24	3.9% (3.3%-4.5%)	1		1	
25-34	8.2% (7.1%-9.3%)	2.24 (1.79-2.80)		1.97 (1.50-2.59)	
35-44	12.2% (10.7%-13.8%)	3.57 (2.87-4.34)		3.24 (2.45-4.27)	
45-54	17.6% (15.4%-19.8%)	5.79 (4.63-7.26)		5.70 (4.28-7.59)	
55-64	27.6% (24.0%-31.2%)	10.93 (8.49-14.07)		10.90 (7.92-15.01)	
65 and over	43.7% (40.1%-47.2%)	24.32 (19.37-30.53)		26.77 (19.69-36.38)	
**Site**			<0.001		<0.001
Bonda Mission (subsistence farming area)	14.1% (12.3%-15.9%)	1		1	
Eastern Highlands (tea estate)	9.4% (4.6%-7.6%)	0.92 (0.66-1.29)		0.83 (0.59-1.18)	
Selbourne (forestry estate)	9.4% (7.8%-11.0%)	1.04 (0.79-1.39)		1.16 (0.87-1.54)	
Nyazura (town)	13.3% (11.4%-15.1%)	1.40 (1.08-1.82)		1.32 (1.01-1.72)	
Nyanga (town)	9.3% (7.5%-11.1%)	1.09 (0.82-1.46)		0.80 (0.57-1.12)	
Watsomba (roadside settlement)	16.9% (15.0%-18.7%)	1.43 (1.13-1.80)		1.48 (1.16-1.88)	
Sakubva (urban)	28.2% (24.9%-31.5%)	5.28 (4.03-6.91)		4.36 (3.21-5.90)	
Hobhouse (urban)	15.5% (12.8%-18.1%)	2.73 (2.03-3.66)		1.93 (1.38-2.70)	
**Employment**			0.001		0.006
Unemployed	19.5% (18.3%-20.6%)	1		1	
Employed or in education	9.0% (8.2%-9.8%)	0.79 (0.68-0.91)		0.80 (0.69-0.94)	
**Relationship status**			<0.001		<0.001
Married or in a long-term relationship	14.6% (13.6%-15.5%)	1		1	

For peer review only

Never been in a long-term relationship	3.9% (3.2%-4.7%)	0.50 (0.38-0.66)		0.54 (0.41-0.73)	
Separated or divorced	15.8% (13.1%-18.6%)	0.92 (0.72-1.18)		1.03 (0.79-1.33)	
Widowed	35.9% (32.3%-39.4%)	0.67 (0.54-0.83)		0.74 (0.58-0.95)	
**Education**			<0.001		-
None or primary	24.1% (22.3%-26.0%)	1		-	
Secondary or higher	10.1% (9.5%-10.9%)	1.52 (1.24-1.86)		-	
**Asset-based wealth**			<0.001		<0.001
1 (poorest)	11.8% (10.3%-13.3%)	1		1	
2	11.4% (9.9%-12.9%)	1.09 (0.86-1.39)		1.15 (0.89-1.47)	
3	13.0% (11.5%-14.6%)	1.38 (1.10-1.74)		1.40 (1.10-1.79)	
4	15.8% (14.1%-17.4%)	2.11 (1.69-2.65)		1.52 (1.17-1.98)	
5 (richest)	16.0% (14.3%-17.7%)	2.31 (1.84-2.90)		2.11 (1.61-2.76)	

Association between ART-exposure and self-reported hypertension

Over half of PLHIV were ART-exposed (65.8%, 62.8-69.0%, Table 1). The prevalence of self-reported hypertension was similar among ART-exposed PLHIV (14.8%, 12.0%-17.7%) and ART-naïve PLHIV in crude univariable comparisons (12.8%, 9.1%-16.4%, F=0.7, p=0.388) and the lack of a difference remained in univariable analyses of the prevalence of self-reported hypertension split by age and sex (Supplementary fig. 1).

In multivariable logistic regression analysis, there was no evidence of a relationship between ART-exposure and self-reported hypertension after adjustment for age and sex (OR=0.87, 95% CI=0.56-1.33, p=0.519) or after further adjustment for site (0.83, 0.53-1.30, p=0.411; Table 3).

Results for sensitivity analyses focused on current ART usage and adherent current ART usage were similar to those for ART-exposure (Supplementary tables 2-4). Fully adjusted multivariable logistic regression models showed no evidence of a relationship between current ART usage and hypertension (0.80, 0.51-1.25, p=0.312; Supplementary table 3), or adherent current ART usage and hypertension (0.70, 0.46-1.08, p=0.107; Supplementary table 4).

For peer review only

Table 3. Antiretroviral therapy as a determinant of hypertension in people living with HIV.

	Weighted hypertension prevalence, % (95% Confidence Interval)	Odds ratio adjusted for only age and sex (95% confidence interval)	P-value	Fully adjusted odds ratio (95% confidence interval)	P-value
**ART-exposure[1]**			0.519		0.411
ART-naïve	12.8% (9.1%-16.4%)	1		1	
ART-exposed	14.8% (12.0%-17.7%)	0.87 (0.56-1.33)		0.83 (0.53-1.30)	
**Sex**			<0.001		<0.001
Male	6.0% (3.3%-8.8%)	1		1	
Female	20.3% (17.1%-23.6%)	5.11 (2.95-8.84)		5.73 (3.23-10.17)	
**Age, years**			<0.001		<0.001
15-24	7.9% (2.6%-13.1%)	1		1	
25-34	12.3% (7.8%-16.8%)	1.69 (0.72-3.94)		2.03 (0.86-4.80)	
35-44	13.1% (9.2%-17.0%)	1.87 (0.83-4.23)		2.58 (1.12-5.93)	
45-54	12.0% (8.2%-15.8%)	2.01 (0.88-4.58)		2.80 (1.19-6.59)	
55-64	24.7% (14.7%-34.6%)	6.19 (2.43-15.75)		9.54 (3.43-26.49)	
65 and over	27.8% (13.8%-41.9%)	7.53 (2.71-20.94)		12.51 (4.16-37.67)	
**Site**			<0.001		<0.001
Bonda Mission (subsistence farming area)	13.3% (7.8%-18.9%)	1		1	
Eastern Highlands (tea estate)	6.1% (2.0%-10.2%)	0.64 (0.26-1.57)		0.64 (0.26-1.56)	
Selbourne (forestry estate)	8.3% (3.9%-12.6%)	0.82 (0.37-1.82)		0.81 (0.37-1.80)	
Nyazura (town)	19.6% (13.5%-25.8%)	2.13 (1.10-4.14)		2.15 (1.10-4.17)	
Nyanga (town)	6.1% (0.1%-12.0%)	0.44 (0.13-1.45)		0.43 (0.13-1.44)	
Watsomba (roadside settlement)	10.6% (5.8%-15.3%)	0.84 (0.42-1.67)		0.82 (0.41-1.64)	
Sakubva (urban)	30.9% (20.9%-40.8%)	4.58 (2.21-9.51)		4.53 (2.19-9.41)	
Hobhouse (urban)	25.8% (14.4%-37.3%)	3.29 (1.43-7.54)		3.27 (1.43-7.50)	
**Employment**			0.352		-
Unemployed	16.0% (12.7%-19.3%)	1		-	
Employed or in education	12.3% (9.3%-15.4%)	1.20 (0.81-1.78)		-	
**Relationship status**			0.671		-
Married or in a long-term relationship	12.4% (9.6%-15.2%)	1		-	

For peer review only

Never been in a long-term relationship	11.1% (4.2%-18.0%)	0.81 (0.33-2.03)		-	
Separated or divorced	17.2% (10.9%-23.4%)	1.17 (0.70-1.95)		-	
Widowed	19.8% (13.6%-25.9%)	0.81 (0.50-1.31)		-	
**Education**			0.562		-
None or primary	17.6% (12.8%-22.4%)	1		-	
Secondary or higher	12.6% (10.0%-15.1%)	1.15 (0.72-1.81)		-	
**Asset-based wealth**			0.003		-
1 (poorest)	8.8% (5.1%-12.5%)	1		-	
2	12.0% (7.5%-16.5%)	1.67 (0.88-3.16)		-	
3	12.6% (8.0%-17.2%)	1.64 (0.86-3.15)		-	
4	18.0% (12.7%-23.3%)	2.75 (1.49-5.09)		-	
5 (richest)	22.9% (15.3%-30.5%)	3.15 (1.64-6.07)		-	

[1]ART, antiretroviral therapy.

Discussion

This study aimed to examine whether HIV status and ART-exposure were associated with self-reported hypertension in Manicaland, East Zimbabwe. In this setting, approximately one in seven PLHIV self-reported hypertension. There was no evidence of a difference in the prevalence of self-reported hypertension by HIV status. Furthermore, no evidence was found for an association between ART-exposure and self-reported hypertension among PLHIV.

The results of this study corroborate previous estimates of the prevalence of hypertension in Zimbabwean PLHIV. The prevalence of self-reported hypertension in PLHIV in Manicaland was 14.1% (11.9%-16.3%), which is similar to earlier estimates of 8.9%, 18.3% and 19.8% from elsewhere in the country.[14–16] The alignment of this study's findings with previous estimates indicates that the results may be a realistic representation of the prevalence of hypertension. However, this means that there is already an important burden of hypertension in PLHIV in Zimbabwe, which is likely to grow as the population grows older and unhealthy lifestyles become more widespread.[30,31] The use of newer integrase inhibitors may also increase weight gain among PLHIV and raise the prevalence of hypertension further.[32,33] Consequently, more prevention and treatment services

For peer review only

may be required to avoid worsening outcomes.

Knowledge of the burden of hypertension experienced by PLHIV is key to providing prevention and treatment services as efficiently as possible, particularly given the scare resources available.[31,34] This study found a similar prevalence of self-reported hypertension among PLHIV and HIV-negative people. These findings differ from those from a systematic review which pooled prevalence estimates gathered across Africa and indicated that PLHIV might experience a lower prevalence of hypertension.[6] The difference may be explained in part by regional variation in the relationship between HIV and hypertension within Africa and in part due to the different approaches to detection of hypertension that were used. The systematic review focused on studies reporting clinical diagnoses of hypertension, excluding data on self-reported hypertension as is presented here.[6] Further, a study of data from the Agincourt Health and Demographic Surveillance System in South Africa, which neighbours Zimbabwe, also reported that the prevalence of hypertension among PLHIV was comparable to that of HIV-negative people.[35] The South African study and the global systematic review suggest that regional differences may exist, which should be fully accounted for when developing guidelines for scale-up of hypertension services.[6,35] Notably, it will be important to identify and manage other risk factors that are associated with hypertension in Zimbabwe.

Our findings further indicated that the prevalence of self-reported hypertension was similar among ART-exposed and ART-naïve PLHIV. Several large studies, including the Data Collection on Adverse Events of Anti-HIV Drugs (D:A:D) study, have obtained similar results, but a previous study in Zimbabwe reported a higher prevalence of hypertension among PLHIV on ART.[14,36] The conflicting results from the two Zimbabwean studies may be due to differences in how ART-exposure was measured. While this study focused on whether PLHIV had ever taken ART, the previous Zimbabwean study examined the effect of taking ART for more than five years.[14] Detailed data on ART interruption and discontinuation over time are not available from the Manicaland Study. However, additional research examining how patterns of ART-exposure over time relate to hypertension risk might clarify the source of these differences and give further insight into how hypertension services should be delivered.

For peer review only - http://bmjopen.bmj.com/site/about/guidelines.xhtml

Strengths and limitations

To our knowledge, this is the first study to provide estimates of the prevalence of hypertension among comparable populations of PLHIV and HIV-negative people in a non-clinical setting in Zimbabwe. This study also used robust methodology to explore links between HIV status, ART-exposure, and self-reported hypertension, building the evidence base on links between HIV and non-communicable diseases. In addition, the use of data from a large population survey should have increased the generalisability of the findings to other similar settings in Zimbabwe. Finally, all analyses were underpinned by individual-level data that included a diverse range of men and women, and could be weighted to local census data, which enhanced the representativeness of results.

However, this work also has some limitations. These fall across five broad areas: the definition of hypertension, unmeasured risk factors for hypertension, selection biases towards the healthy, the use of a large number of significance tests, and the cross-sectional nature of the analysis. Firstly, funding limitations led to a reliance on self-report of previous diagnoses by a doctor or nurse as a proxy to detect disease, which may have resulted in underestimation of the underlying hypertension burden.[37] Limited access to screening and diagnosis will likely have meant that some hypertensive people in our study population were at the early stages of the hypertension care cascade and unaware of their hypertension, and so were excluded from our estimates.[38–41] Notably, a 2015 meta-analysis of four studies indicated that the prevalence of hypertension in the general population in Zimbabwe may be 30%, which is higher than our estimates of the prevalence of self-reported hypertension among PLHIV and HIV-negative people. The difference between the two reports suggests that a substantial proportion of the participants in this study could have been unaware that they had hypertension and so were not included in our estimates. [42] This would reduce the generalisability of our findings for settings in which hypertension screening and diagnosis is more readily available. In addition, HIV-negative people may have had less contact with the healthcare system for hypertension diagnosis than PLHIV, which could have artificially reduced self-reporting of hypertension in this group.[43] Yet the prevalence of hypertension was comparable among PLHIV who knew their status (14.7%, 11.9%-17.5%) and PLHIV who did not (15.1%, 10.6%-19.5%), suggesting that contact with the healthcare system among diagnosed PLHIV did not increase self-reporting of hypertension. In future survey rounds, quantification of the prevalence of

For peer review only

hypertension by measuring participants' blood pressure during the individual interview, and asking questions to detect medications for hypertension, would provide more reliable estimates of this burden. [44] The results of this analysis could then be updated accordingly.

As well as limitations linked to the definition of hypertension, data were not available on several risk factors for hypertension, such as body mass index (BMI), diet and physical activity, which precluded their use in the analysis.[45,46] Future studies should measure the height and weight of participants and calculate their BMI, and gather data on diet and physical activity levels among participants, so that analyses can be adjusted for these risk factors. Moreover, people with hypertension, or conditions caused by hypertension, were more likely to be seriously ill and so unable to complete the individual interview, or in locations, such as hospitals, which were not reached by the field teams. In addition, the analysis involved a large number of significance tests, without correcting for multiple comparisons, which can increase the probability of a false positive finding due to chance. Finally, assessment of the association between HIV and ART and self-reported hypertension was constrained by the cross-sectional nature of the analysis. The development of a cohort that consisted of matched PLHIV and HIV-negative controls in Manicaland, which could be followed over time, would allow more certainty in the drawing of conclusions about the relationship.[14]

Conclusion

Approximately one in seven PLHIV self-reported hypertension in Manicaland, Zimbabwe. However, HIV status and ART-exposure were not associated with self-reported hypertension. Zimbabwe is re-orienting its health system towards control of hypertension and other non-communicable diseases; this study highlights how carefully designed, sufficiently resourced prevention and care interventions for PLHIV and HIV-negative people will be required to make this a success.

For peer review only

Acknowledgements

The authors would like to express our gratitude to everyone who has been involved with the Manicaland Study over the past 20 years, especially the participants and research staff. Without them, this study would not exist.

Funding

This work was supported by the Wellcome Trust [220098/Z/20/Z to KD]; the Bill and Melinda Gates Foundation [OPP1161471 to SG]; the Civilian Research and Development Foundation Global (prime is President's Emergency Plan for AIDS Relief) [OISE-9531011 to MS]; the National Institutes of Health [5R01MH114562-02 to SG and CN; 1R21AG053093-01 to MS] and the Medical Research Council Centre for Global Infectious Disease Analysis [MR/R015600/1 to KD, LM, CN, MS and SG], which is jointly funded by the UK Medical Research Council and the UK Foreign, Commonwealth & Development Office (FCDO), under the MRC/FCDO Concordat agreement and is also part of the EDCTP2 programme supported by the European Union.

The funders had no role in study design, data collection and analysis, decision to publish, or preparation of the manuscript.

Competing interests

SG declares holding shares in AstraZeneca and GlaxoSmithKline. All other authors declare that no competing interests exist.

Authors' contributions

MS, SG and KD conceived the analysis and developed the methods. SG, CN, RM, PM, and TM led the data collection and TD and LM cleaned, extracted, and prepared the data. KD performed the analysis and generated figures and estimates. KD led the writing of the manuscript with MS and SG, and all authors commented on and contributed to the finalisation of the manuscript.

For peer review only

Data availability

Data are available upon reasonable request. The data are deidentified participant data and can be obtained by sending a data request form (available from the Manicaland Study website: http://www.manicalandhivproject.org/data.html) to s.gregson@imperial.ac.uk.

Ethics statement

The Manicaland Study was conducted with the understanding and informed consent of participants. Ethical approval was given by Imperial College Research Ethics Committee (17IC4160), the Biomedical Research and Training Institute Institutional Review Board (AP140/2017) and the Medical Research Council of Zimbabwe (MRCZ/A/2243).

For peer review only

References

1 Stanaway JD, Afshin A, Gakidou E, *et al.* Global, regional, and national comparative risk assessment of 84 behavioural, environmental and occupational and metabolic risks or clusters of risks for 195 countries and territories 1990-2017: A systematic analysis for the Global Burden of Disease Study. *Lancet* 2018;**392**:1923–94. doi:10.1016/S0140-6736(18)32225-6

2 Mills KT, Bundy JD, Kelly TN, *et al.* Global Disparities of Hypertension Prevalence and Control. *Circulation* 2016;**134**:441–50. doi:10.1161/CIRCULATIONAHA.115.018912

3 Angkurawaranon C, Nitsch D, Larke N, *et al.* Ecological Study of HIV Infection and Hypertension in Sub-Saharan Africa: Is There a Double Burden of Disease?. *PLoS One* 2016;**11**:e0166375. doi:10.1371/journal.pone.0166375

4 van Zoest RA, van den Born B-JH, Reiss P. Hypertension in people living with HIV. *Curr Opin HIV AIDS* 2017;**12**:513–22. doi:10.1097/COH.0000000000000406

5 Fahme SA, Bloomfield GS, Peck R. Hypertension in HIV-Infected Adults: Novel Pathophysiologic Mechanisms. *Hypertension* 2018;**72**:44–55. doi:10.1161/HYPERTENSIONAHA.118.10893

6 Davis K, Perez-Guzman P, Hoyer A, *et al.* Association between HIV infection and hypertension: a global systematic review and meta-analysis of cross-sectional studies. *BMC Med* 2021;**19**:105. doi:10.1186/s12916-021-01978-7

7 Okello S, Ueda P, Kanyesigye M, *et al.* Association between HIV and blood pressure in adults and role of body weight as a mediator: Cross-sectional study in Uganda. *J Clin Hypertens* 2017;**19**:1181–91. doi:10.1111/jch.13092

8 Dillon DG, Gurdasani D, Riha J, *et al.* Association of HIV and ART with cardiometabolic traits in sub-Saharan Africa: a systematic review and meta-analysis. *Int J Epidemiol* 2013;**42**:1754–71. doi:10.1093/ije/dyt198

9 Kwarisiima D, Atukunda M, Owaraganise A, *et al.* Hypertension control in integrated HIV and chronic disease clinics in Uganda in the SEARCH study. *BMC Public Health* 2019;**19**:511.

For peer review only

doi:10.1186/s12889-019-6838-6

10 Althoff KN, Smit M, Reiss P, *et al.* HIV and ageing: Improving quantity and quality of life. *Curr Opin HIV AIDS* 2016;**11**:527–36. doi:10.1097/COH.0000000000000305

11 van Zoest RA, Wit FW, Kooij KW, *et al.* Higher Prevalence of Hypertension in HIV-1-Infected Patients on Combination Antiretroviral Therapy Is Associated With Changes in Body Composition and Prior Stavudine Exposure. *Clin Infect Dis* 2016;**63**:205–13. doi:10.1093/cid/ciw285

12 Mayer KH, Loo S, Crawford PM, *et al.* Excess clinical comorbidity among HIV-infected patients accessing primary care in US community health centers. *Public Health Rep* 2018;**133**:109–18. doi:10.1177/0033354917748670

13 Dalal S, Beunza JJ, Volmink J, *et al.* Non-communicable diseases in sub-Saharan Africa: what we know now. *Int J Epidemiol* 2011;**40**:885–901. doi:10.1093/ije/dyr050

14 Gonah L, Moodley I, Hlongwana K. Prevalence of diabetes mellitus and hypertension in people living with human immunodeficiency virus on antiretroviral therapy in Gweru district, Zimbabwe. *African J Prim Heal Care Fam Med* 2020;**12**:2473. doi:10.4102/PHCFM.V12I1.2473

15 Cheza A, Tlou B, Zhou DT. Incidence of non-communicable diseases (NCDs) in HIV patients on ART in a developing country: Case of Zimbabwe's Chitungwiza Central Hospital—A retrospective cohort study (2010–2019). *PLoS One* 2021;**16**:e0252180. doi:10.1371/journal.pone.0252180

16 Bleasel JM, Heron JE, Shamu T, *et al.* Body mass index and noninfectious comorbidity in HIV-positive patients commencing antiretroviral therapy in Zimbabwe. *HIV Med* 2020;**21**:674–9. doi:10.1111/HIV.12934

17 Zimbabwe Ministry of Health and Child Care. Zimbabwe Health Financing Strategy 2017. Harare, Zimbabwe: 2017. https://zdhr.uz.ac.zw/xmlui/bitstream/handle/123456789/1374/Zimbabwe National Health Financing Strategy 2017.pdf?sequence=1&isAllowed=y

18 Government of Zimbabwe. Zimbabwe National HIV and AIDS strategic plan: 2021-2025.

For peer review only

Harare: 2021. https://www.nac.org.zw/wp-content/uploads/2022/02/ZIMBABWE-NATIONAL-HIV-STATEGIC-PLAN_2021-2025-1.pdf.

19 Government of Zimbabwe. Vision 2030: Towards a Prosperous & Empowered Upper Middle Income Society by 2030. Harare: 2018. http://www.zim.gov.zw/index.php/en/government-documents.

20 Manicaland General Population Cohort. Manicaland HIV project. 2021.http://www.manicalandhivproject.org/.

21 Gregson S, Mugurungi O, Eaton J, *et al.* Documenting and explaining the HIV decline in east Zimbabwe: the Manicaland General Population Cohort. *BMJ Open* 2017;**7**:e015898. doi:10.1136/bmjopen-2017-015898

22 Manicaland Centre for Public Health Research. Multi-Level Behavioural Economics and Community Psychology Approaches to Strengthen HIV Prevention Cascades in Adolescent Girls and Young Women in East Zimbabwe - Study Protocol. Harare: 2018.

23 Thomas R, Skovdal M, Galizzi MM, *et al.* Improving risk perception and uptake of pre-exposure prophylaxis (PrEP) through interactive feedback-based counselling with and without community engagement in young women in Manicaland, East Zimbabwe: study protocol for a pilot randomized trial. *Trials 2019 201* 2019;**20**:1–9. doi:10.1186/S13063-019-3791-8

24 Thomas R, Skovdal M, Galizzi MM, *et al.* Improving risk perception and uptake of voluntary medical male circumcision with peer-education sessions and incentives, in Manicaland, East Zimbabwe: Study protocol for a pilot randomised trial. *Trials* 2020;**21**:108. doi:10.1186/s13063-020-4048-2

25 Lopman B, Lewis J, Nyamukapa C, *et al.* HIV incidence and poverty in Manicaland, Zimbabwe: is HIV becoming a disease of the poor? *AIDS* 2007;**21 Suppl 7**:S57-66. doi:10.1097/01.aids.0000300536.82354.52

26 Tlhajoane M, Eaton JW, Takaruza A, *et al.* Prevalence and Associations of Psychological Distress, HIV Infection and HIV Care Service Utilization in East Zimbabwe. *AIDS Behav* 2018;**22**:1485–95. doi:10.1007/s10461-017-1705-x

For peer review only

27 Rhead R, Elmes J, Otobo E, *et al.* Do female sex workers have lower uptake of HIV treatment services than non-sex workers? A cross-sectional study from east Zimbabwe. *BMJ Open* 2018;**8**:18751. doi:10.1136/bmjopen-2017-018751

28 R Core Team. R: A language and environment for statistical computing. 2020.https://www.r-project.org/.

29 Lumely T. Analysis of complex survey samples. *J Stat Softw* 2004;**9**:1–19. doi:10.18637/jss.v009.i08

30 Ibrahim MM, Damasceno A. Hypertension in developing countries. *Lancet* 2012;**380**:611–9. doi:10.1016/S0140-6736(12)60861-7

31 Smit M, Olney J, Ford NP, *et al.* The growing burden of noncommunicable disease among persons living with HIV in Zimbabwe. *AIDS* 2018;**32**:773–82. doi:10.1097/QAD.0000000000001754

32 Byonanebye DM, Polizzotto MN, Neesgaard B, *et al.* Incidence of hypertension in people with HIV who are treated with integrase inhibitors versus other antiretroviral regimens in the RESPOND cohort consortium. *HIV Med* 2022;**23**:895–910. doi:10.1111/HIV.13273

33 Ruderman SA, Crane HM, Nance RM, *et al.* Brief Report: Weight Gain Following ART Initiation in ART-Naïve People Living With HIV in the Current Treatment Era. *J Acquir Immune Defic Syndr* 2021;**86**:339–43. doi:10.1097/QAI.0000000000002556

34 Oparil S, Acelajado MC, Bakris GL, *et al.* Hypertension. *Nat Rev Dis Prim* 2018;**4**:18014. doi:10.1038/nrdp.2018.14

35 Clark SJ, Gomez-Olive FX, Houle B, *et al.* Cardiometabolic disease risk and HIV status in rural South Africa: establishing a baseline. *BMC Public Health* 2015;**15**:135. doi:10.1186/s12889-015-1467-1

36 Hatleberg CI, Ryom L, d'Arminio Monforte A, *et al.* Association between exposure to antiretroviral drugs and the incidence of hypertension in HIV-positive persons: the Data Collection on Adverse Events of Anti-HIV Drugs (D:A:D) study. *HIV Med* 2018;**19**:605–18. doi:10.1111/hiv.12639

For peer review only

37 Ataklte F, Erqou S, Kaptoge S, *et al.* Burden of Undiagnosed Hypertension in Sub-Saharan Africa. *Hypertension* 2015;**65**:291–8. doi:10.1161/HYPERTENSIONAHA.114.04394

38 Mungati M, Manangazira P, Takundwa L, *et al.* Factors affecting diagnosis and management of hypertension in Mazowe District of Mashonaland Central Province in Zimbabwe: 2012. *BMC Cardiovasc Disord* 2014;**14**:102. doi:10.1186/1471-2261-14-102

39 Sorato MM, Davari M, Kebriaeezadeh A, *et al.* Reasons for poor blood pressure control in Eastern Sub-Saharan Africa: looking into 4P's (primary care, professional, patient, and public health policy) for improving blood pressure control: a scoping review. *BMC Cardiovasc Disord* 2021;**21**. doi:10.1186/s12872-021-01934-6

40 Wozniak G, Khan T, Gillespie C, *et al.* Hypertension Control Cascade: A Framework to Improve Hypertension Awareness, Treatment, and Control. *J Clin Hypertens* 2016;**18**:232–9. doi:10.1111/JCH.12654

41 Berry KM, Parker WA, McHiza ZJ, *et al.* Quantifying unmet need for hypertension care in South Africa through a care cascade: evidence from the SANHANES, 2011-2012. *BMJ Glob Heal* 2017;**2**:e000348. doi:10.1136/BMJGH-2017-000348

42 Mutowo MP, Mangwiro JC, Lorgelly P, *et al.* Hypertension in Zimbabwe: A meta-analysis to quantify its burden and policy implications. *World J Meta-Analysis* 2015;**3**:54. doi:10.13105/wjma.v3.i1.54

43 Manne-Goehler J, Montana L, Gomez-Olive FX, *et al.* The ART Advantage: Health Care Utilization for Diabetes and Hypertension in Rural South Africa. *J Acquir Immune Defic Syndr* 2017;**75**:561–7. doi:10.1097/QAI.0000000000001445

44 Thomas R, Burger R, Hauck K. Richer, wiser and in better health? The socioeconomic gradient in hypertension prevalence, unawareness and control in South Africa. *Soc Sci Med* 2018;**217**:18–30. doi:10.1016/j.socscimed.2018.09.038

45 World Health Organisation. A global brief on hypertension. Geneva: 2013. https://apps.who.int/iris/handle/10665/79059.

46 Linderman GC, Lu J, Lu Y, *et al.* Association of Body Mass Index With Blood Pressure Among 1.7 Million Chinese Adults. *JAMA Netw Open* 2018;**1**:e181271–e181271.

For peer review only

doi:10.1001/JAMANETWORKOPEN.2018.1271

Legends

Figure 1. Flow of study participants. DBS, dried blood spot.

Figure 2. Prevalence of reported hypertension in men and women by age and HIV status. Error bars: 95% confidence intervals.

For peer review only

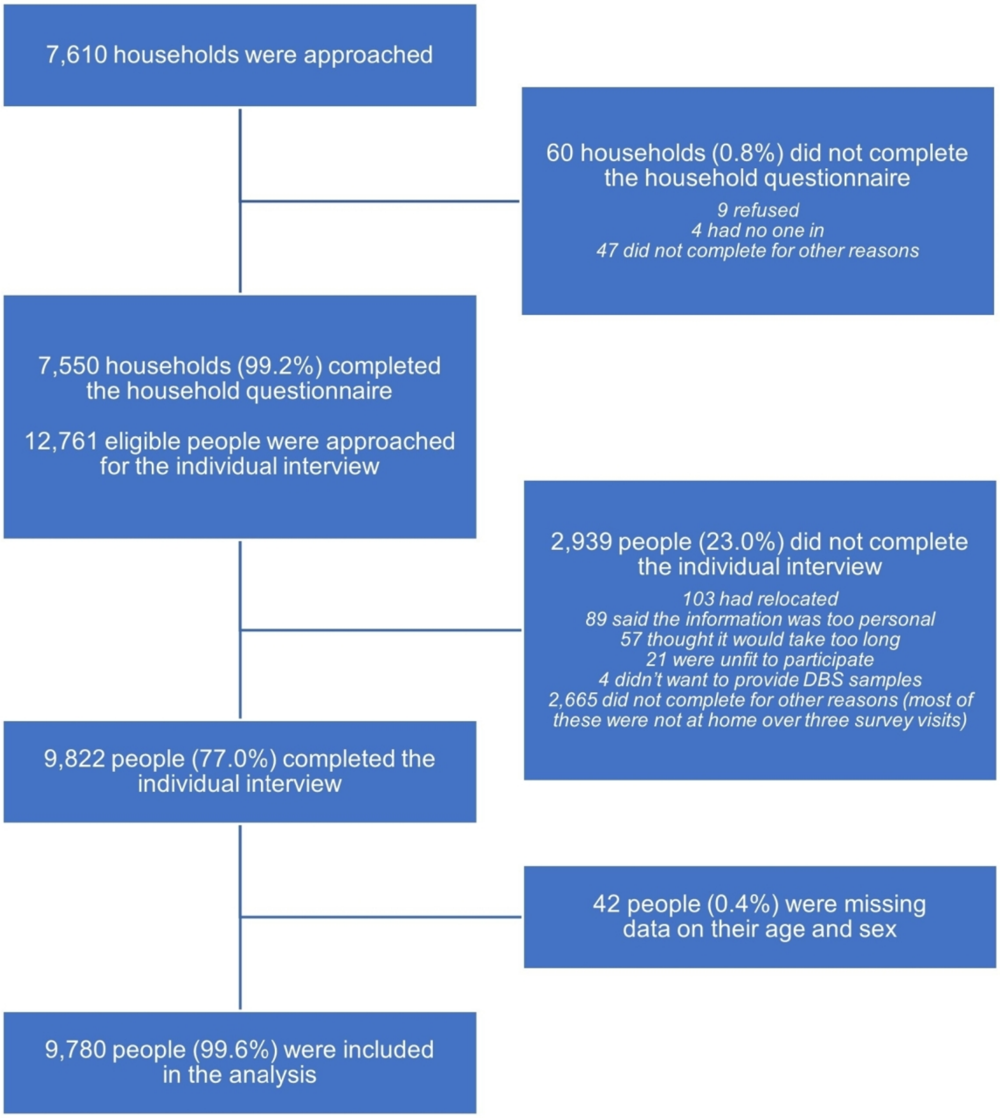

Figure 1. Flow of study participants. DBS, dried blood spot.

145x167mm (300 x 300 DPI)

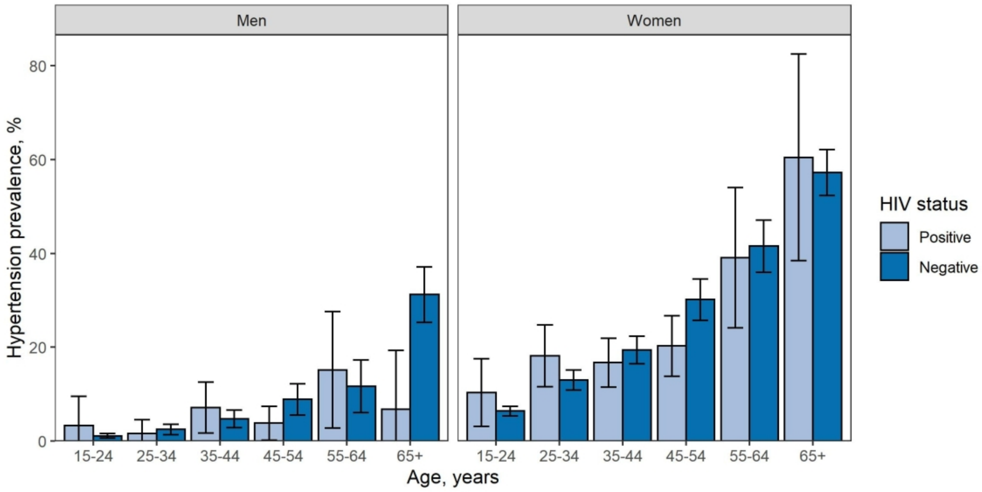

Figure 2. Prevalence of reported hypertension in men and women by age and HIV status. Error bars: 95% confidence intervals.

203x101mm (300 x 300 DPI)

Supplementary material

Supplementary table 1. Reported hypertension (HPN) prevalence by HIV status, age, and sex. 2

Supplementary figure 1. Prevalence of reported hypertension in men and women by age and ART exposure. Error bars: 95% confidence intervals. 3

Supplementary table 2. Prevalence of current antiretroviral therapy usage and adherent current antiretroviral therapy usage among people living with HIV (N=936) 4

Supplementary table 3. Current antiretroviral therapy usage as a determinant of hypertension in people living with HIV. 5

Supplementary table 4. Adherent current antiretroviral therapy usage as a determinant of hypertension in people living with HIV. 7

Manicaland Study Questionnaires. 9

For peer review only

Supplementary table 1. Reported hypertension (HPN) prevalence by HIV status, age, and sex.

Sex	Age	People living with HIV			HIV-negative people			F-statistic	p-value
		Weighted prevalence, % (95% Confidence Interval)	N for those with HPN[1]	N for those with no HPN[1]	Weighted prevalence, % (95% Confidence Interval)	N for those with HPN[1]	N for those with no HPN[1]		
Men	15-24	3.2% (0-8.5%)	1	29	1.1% (0.6-1.6%)	17	1,590	1.3	0.261
Men	25-34	1.5% (0-4.5%)	1	57	2.4% (1.3-3.6%)	18	721	0.2	0.645
Men	35-44	7.1% (1.6-12.6%)	6	80	4.7% (2.8-6.5%)	23	474	0.9	0.343
Men	45-54	3.8% (0.1-7.4%)	4	100	8.8% (5.5-12.2%)	25	261	2.9	0.090
Men	55-64	15.1% (2.7-27.6%)	5	26	11.6% (6.0-17.2%)	15	116	0.3	0.586
Men	65+	6.7% (0-19.3%)	1	18	31.2% (25.3-37.2%)	74	168	4.1	0.043
Women	15-24	10.3% (3.1-17.5%)	7	60	6.4% (5.3-7.4%)	133	1,932	1.7	0.197
Women	25-34	18.1% (11.6-24.7%)	24	107	12.9% (10.8-15.1%)	123	819	2.6	0.104
Women	35-44	16.7 (11.5-21.9%)	33	163	19.4% (16.4-22.3%)	134	555	0.7	0.402
Women	45-54	20.2% (13.8-26.7%)	30	118	30.1% (25.7-34.5%)	124	287	5.4	0.021
Women	55-64	39.1% (24.1-54.0%)	16	25	41.5% (36.0-47.1%)	124	176	0.1	0.763
Women	65+	60.5% (38.5-82.5%)	11	8	57.2% (52.3-62.1%)	233	174	0.1	0.778

[1]Values are unweighted. Individuals without a recorded HIV status or with missing data for hypertension reporting were excluded.

For peer review only

Supplementary figure 1. Prevalence of reported hypertension in men and women by age and ART exposure. Error bars: 95% confidence intervals.

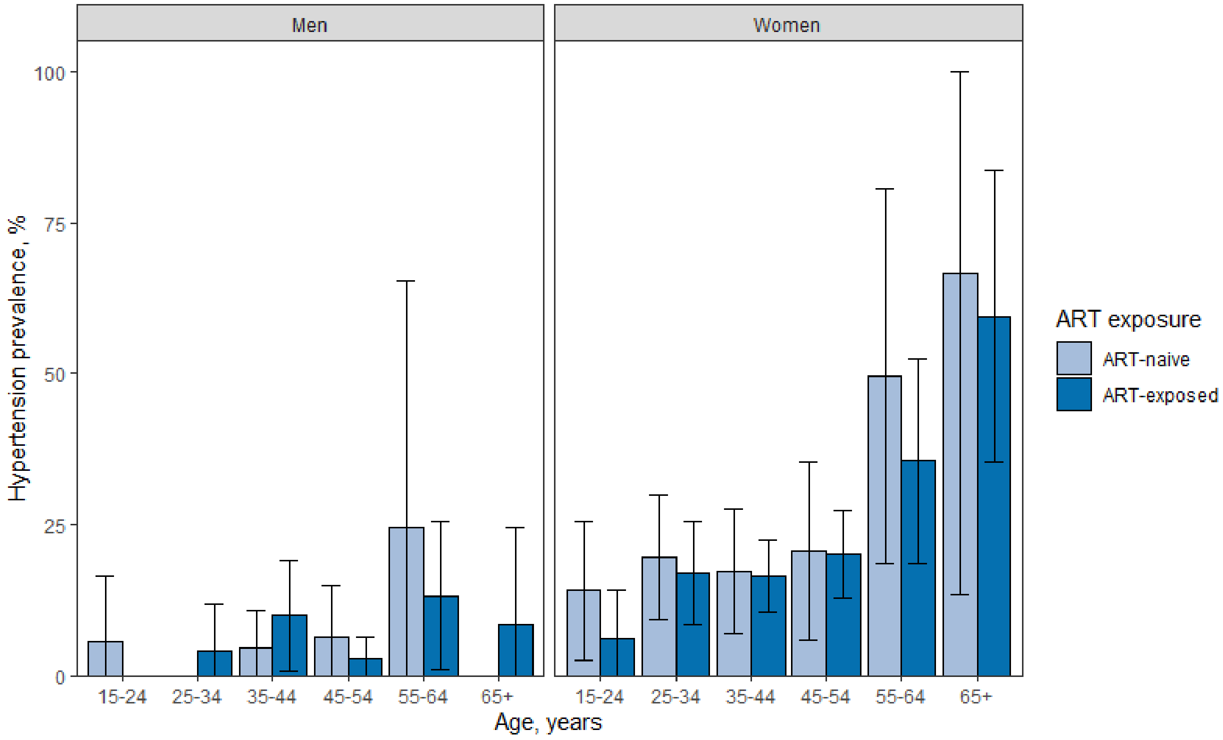

review only

Supplementary table 2. Prevalence of current antiretroviral therapy usage and adherent current antiretroviral therapy usage among people living with HIV (N=936)

	Weighted proportion, % (95% Confidence Interval)	N[1]
**Current ART usage[2]**		
Not using ART	34.5% (31.4%-37.6%)	328
Using ART	65.5% (62.3%-68.6%)	607
Missing	-	1
**Adherent current ART usage[2]**		
Not currently using ART or not adherent to ART	39.8% (36.6%-43.0%)	377
Currently using and adherent to ART	60.2% (57.0%-63.4%)	558
Missing	-	1

[1]Values are unweighted.

[2]ART, antiretroviral therapy.

For peer review only

Supplementary table 3. Current antiretroviral therapy usage as a determinant of hypertension in people living with HIV.

	Weighted hypertension prevalence, % (95% Confidence Interval)	Odds ratio adjusted for only age and sex (95% confidence interval)	P-value	Fully adjusted odds ratio (95% confidence interval)	P-value
**Current ART usage**[1]			0.383		0.321
Not using ART	13.2% (9.5%-16.8%)	1		1	
Using ART	14.6% (11.8%-17.5%)	0.83 (0.54-1.27)		0.80 (0.51-1.25)	
**Sex**			<0.001		<0.001
Male	6.0% (3.3%-8.8%)	1		1	
Female	20.3% (17.1%-23.6%)	5.11 (2.95-8.84)		5.74 (3.24-10.19)	
**Age, years**			<0.001		<0.001
15-24	7.9% (2.6%-13.1%)	1		1	
25-34	12.3% (7.8%-16.8%)	1.69 (0.72-3.94)		2.07 (0.87-4.90)	
35-44	13.1% (9.2%-17.0%)	1.87 (0.83-4.23)		2.64 (1.14-6.11)	
45-54	12.0% (8.2%-15.8%)	2.01 (0.88-4.58)		2.87 (1.21-6.82)	
55-64	24.7% (14.7%-34.6%)	6.19 (2.43-15.75)		9.75 (3.48-27.31)	
65 and over	27.8% (13.8%-41.9%)	7.53 (2.71-20.94)		12.86 (4.22-39.14)	
**Site**			<0.001		<0.001
Bonda Mission (subsistence farming area)	13.3% (7.8%-18.9%)	1		1	
Eastern Highlands (tea estate)	6.1% (2.0%-10.2%)	0.64 (0.26-1.57)		0.63 (0.26-1.56)	
Selbourne (forestry estate)	8.3% (3.9%-12.6%)	0.82 (0.37-1.82)		0.81 (0.37-1.80)	
Nyazura (town)	19.6% (13.5%-25.8%)	2.13 (1.10-4.14)		2.15 (1.10-4.18)	
Nyanga (town)	6.1% (0.1%-12.0%)	0.44 (0.13-1.45)		0.43 (0.13-1.44)	
Watsomba (roadside settlement)	10.6% (5.8%-15.3%)	0.84 (0.42-1.67)		0.81 (0.40-1.62)	
Sakubva (urban)	30.9% (20.9%-40.8%)	4.58 (2.21-9.51)		4.49 (2.16-9.32)	
Hobhouse (urban)	25.8% (14.4%-37.3%)	3.29 (1.43-7.54)		3.26 (1.42-7.47)	
**Employment**			0.352		-
Unemployed	16.0% (12.7%-19.3%)	1		-	
Employed or in education	12.3% (9.3%-15.4%)	1.20 (0.81-1.78)		-	
**Relationship status**			0.671		-
Married or in a long-term relationship	12.4% (9.6%-15.2%)	1		-	
Never been in a long-term relationship	11.1% (4.2%-18.0%)	0.81 (0.33-2.03)		-	
Separated or divorced	17.2% (10.9%-23.4%)	1.17 (0.70-1.95)		-	
Widowed	19.8% (13.6%-25.9%)	0.81 (0.50-1.31)		-	

For peer review only

**Education**			0.562	-
None or primary	17.6% (12.8%-22.4%)	1	-	
Secondary or higher	12.6% (10.0%-15.1%)	1.15 (0.72-1.81)	-	
**Asset-based wealth**			0.003	-
1 (poorest)	8.8% (5.1%-12.5%)	1	-	
2	12.0% (7.5%-16.5%)	1.67 (0.88-3.16)	-	
3	12.6% (8.0%-17.2%)	1.64 (0.86-3.15)	-	
4	18.0% (12.7%-23.3%)	2.75 (1.49-5.09)	-	
5 (richest)	22.9% (15.3%-30.5%)	3.15 (1.64-6.07)	-	

[1]ART, antiretroviral therapy.

For peer review only

Supplementary table 4. Adherent current antiretroviral therapy usage as a determinant of hypertension in people living with HIV.

	Weighted hypertension prevalence, % (95% Confidence Interval)	Odds ratio adjusted for only age and sex (95% confidence interval)	P-value	Fully adjusted odds ratio (95% confidence interval)	P-value
**Adherent current ART usage[1]**			0.125		0.107
Not currently using ART or not adherent to ART	14.0% (10.5%-17.5%)	1		1	
Currently using and adherent to ART	14.2% (11.3%-17.1%)	0.73 (0.49-1.09)		0.70 (0.46-1.08)	
**Sex**			<0.001		<0.001
Male	6.0% (3.3%-8.8%)	1		1	
Female	20.3% (17.1%-23.6%)	5.11 (2.95-8.84)		5.86 (3.32-10.36)	
**Age, years**			<0.001		<0.001
15-24	7.9% (2.6%-13.1%)	1		1	
25-34	12.3% (7.8%-16.8%)	1.69 (0.72-3.94)		2.12 (0.89-5.07)	
35-44	13.1% (9.2%-17.0%)	1.87 (0.83-4.23)		2.74 (1.18-6.34)	
45-54	12.0% (8.2%-15.8%)	2.01 (0.88-4.58)		3.00 (1.27-7.10)	
55-64	24.7% (14.7%-34.6%)	6.19 (2.43-15.75)		10.30 (3.69-28.74)	
65 and over	27.8% (13.8%-41.9%)	7.53 (2.71-20.94)		13.77 (4.52-41.96)	
**Site**			<0.001		<0.001
Bonda Mission (subsistence farming area)	13.3% (7.8%-18.9%)	1		1	
Eastern Highlands (tea estate)	6.1% (2.0%-10.2%)	0.64 (0.26-1.57)		0.63 (0.26-1.56)	
Selbourne (forestry estate)	8.3% (3.9%-12.6%)	0.82 (0.37-1.82)		0.80 (0.36-1.78)	
Nyazura (town)	19.6% (13.5%-25.8%)	2.13 (1.10-4.14)		2.16 (1.11-4.20)	
Nyanga (town)	6.1% (0.1%-12.0%)	0.44 (0.13-1.45)		0.42 (0.13-1.39)	
Watsomba (roadside settlement)	10.6% (5.8%-15.3%)	0.84 (0.42-1.67)		0.79 (0.40-1.58)	
Sakubva (urban)	30.9% (20.9%-40.8%)	4.58 (2.21-9.51)		4.37 (2.10-9.11)	
Hobhouse (urban)	25.8% (14.4%-37.3%)	3.29 (1.43-7.54)		3.22 (1.40-7.42)	
**Employment**			0.352		-
Unemployed	16.0% (12.7%-19.3%)	1		-	
Employed or in education	12.3% (9.3%-15.4%)	1.20 (0.81-1.78)		-	
**Relationship status**			0.671		-
Married or in a long-term relationship	12.4% (9.6%-15.2%)	1		-	

For peer review only

Never been in a long-term relationship	11.1% (4.2%-18.0%)	0.81 (0.33-2.03)		-	
Separated or divorced	17.2% (10.9%-23.4%)	1.17 (0.70-1.95)		-	
Widowed	19.8% (13.6%-25.9%)	0.81 (0.50-1.31)		-	
**Education**			0.562		-
None or primary	17.6% (12.8%-22.4%)	1		-	
Secondary or higher	12.6% (10.0%-15.1%)	1.15 (0.72-1.81)		-	
**Asset-based wealth**			0.003		-
1 (poorest)	8.8% (5.1%-12.5%)	1		-	
2	12.0% (7.5%-16.5%)	1.67 (0.88-3.16)		-	
3	12.6% (8.0%-17.2%)	1.64 (0.86-3.15)		-	
4	18.0% (12.7%-23.3%)	2.75 (1.49-5.09)		-	
5 (richest)	22.9% (15.3%-30.5%)	3.15 (1.64-6.07)		-	

[1]ART, antiretroviral therapy.

For peer review only

Manicaland Study Questionnaires.

The household and individual questionnaires are copied below.

For peer review only

HOUSEHOLD QUESTIONNAIRE **FORM A**

Individual questionnaire eligibility:

IVQs: young adults	Y N
IVQs: older adults	Y N

Questionnaire processing dates:

Corrections generated
Corrections completed
Batch

HOUSEHOLD IDENTIFICATION

Q001 **Census district:** ____________ **Ward:** [ ]

Q002 **Village:** ____________

Q003 **Name of household head (R6):** ____________

Q004 **Name of household head (R7):** ____________

Q005 **Category of household (R6)*:** [ ]

Q006 **Study site reference & cluster.** [ ][ ]

Q007 **Household number:** [ ]

Q008 **Growth point or compound:** Yes 1 / No 2

Q009 **Distance from nearest (tarred) roadside business centre** [ ]

Q010 **GPS coordinates** Longitude / Latitude / Altitude

INTERVIEWER VISIT

		1	2	3
Q011	**Date:**	___	___	___
Q012	**Time:**	___	___	___
Q013	**Interviewer:**	___	___	___
Q014	**Result**:**	[ ]	[ ]	[ ]

Q015 *How many deaths have there been in the household in the last 12 months?* [ ]

Q016 *How many of the people who died were aged 15-59 years at their last birthday?* [ ]

Do NOT correct after completing interview.

CHECKED BY SUPERVISOR

Q017 **Signature:** ____________

Q018 **Date:** ____________

*HOUSEHOLD CATEGORY

Follow-up household from R6	1
Household refused at R6	4
Household missed at R6	5
New household in the area since R6	8
New study site	6
Other (specify) ____________	98

**RESULT CODES

Completed	1
No one at home	2
Household away for duration of survey	3
Postponed	4
Refused	5
Dwelling vacant or address not a dwelling	6
Dwelling destroyed	7
Dwelling not found	8
Other (specify) ____________	9
Household relocated - within study areas	10
Household relocated - outside study areas	11
Household dispersed (split up)	12

For peer review only - http://bmjopen.bmj.com/site/about/guidelines.xhtml

HOUSEHOLD MEMBERS FORM: UPDATE ON MEMBERS RECORDED IN 2012-2013 SURVEY VISIT

Q. No: [ ]

"Now I would like to get some information about the people who were staying in the household when we came to see you last time"...

LINE NO	USUAL RESIDENTS AND REGULAR VISITORS (ROUND 6)	REL'SHIP TO H.O.H. (R6)	(R7)	SPOUSE IN SAME HOUSEHOLD		SEX	AGE ('12-'13)	PARENTS SURVIVAL FOR CHILDREN UNDER 18 YEARS OLD: FALIVE	FBC	YFDIED	MALIVE	MBC	YMDIED	EDUCATION		HOUSEHOLD MEMBER'S SURVIVAL STATUS NOW			RESIDENCE				ELIGIBILITY FOR INTERVIEW: IVDONE ('12-'13)	CAT'GY (R6)	SELECT (R6)
Q019	Q020	Q021	Q022	Q023		Q024	Q025	Q026		Q027	Q028		Q029	Q030	Q031	Q032			Q034	Q035	Q036	Q037		Q038	Q039
	Circle line no. of respondent.			*Is (NAME) married to another member of this household?* If yes: record spouse's line no.				*Is (NAME)'s NATURAL BIOLOGICAL father still alive?*	Tick "BC" if checked identity - birth certificate.	Year died	*Is (NAME)'s NATURAL BIOLOGICAL mother still alive?*	Tick "BC" if checked identity - birth certificate.	Year died	*State highest level of education completed.***		*Is (NAME) still alive?* If died, record month and year of death. Go to Q035 if more than a year ago.			*Was (NAME) staying here in the same month last year?*	*Does (NAME) still stay in this household on a regular basis? (at death)*	*How many nights is it since (NAME) last slept in / left (see Q035) this household?*	*How many nights has (NAME) slept here in the last month?*	Codes for CATEGORY: …		
				Y N	#	M F	Years	Y N DK	BC	Year	Y N DK	BC	Year	Level	Years	Y N DK	Mth	Yr	Y N	Y N	# or >1yr	#	MUT6NO	#	Code
1	Serina Nyauzanza	1		1 2		2	46	1 2 8			1 2 8					1 2 8			1 2	1 2				1	7
2	Angelina Nyazuna	3		1 2		2	27	1 2 8			1 2 8					1 2 8			1 2	1 2			1	2	1
3	Edmore Nyazuna	5		1 2		1	8	1 2 8			1 2 8					1 2 8			1 2	1 2				3	
4	Telma Nyazuna	5		1 2		2	4	1 2 8			1 2 8					1 2 8			1 2	1 2				4	

For peer review only - http://bmjopen.bmj.com/site/about/guidelines.xhtml

For peer review only

HOUSEHOLD MEMBERS FORM

Q. No: ______

"Now I would like to get some information about the people who were NOT members of the household when we came 5 years ago but who are staying here now or who have done so since we came last time " ...

LINE NO	USUAL RESIDENTS AND REGULAR VISITORS‡	RELATED TO H.O.H. (R7)	SPOUSE IN SAME HOUSEHOLD		SEX	AGE	PARENTS SURVIVAL RELATIONSHIP TO CARER FOR CHILDREN UNDER 18 YEARS OLD						EDUCATION		HOUSEHOLD MEMBER'S SURVIVAL STATUS			RESIDENCE						ELIGIBILITY FOR INTERVIEW CAT'Y	SELECT
Q019	Q020	Q022	Q023		Q024	Q025	Q026		Q027	Q028		Q029	Q030	Q031	Q032			Q033		Q034	Q035	Q036	Q037	Q038	Q039
	Please give me the names of the new persons who have been staying (physically) in this househo on a regular basis (starting with the head of household) Circle line no. of respondent.	*What is the relationship of (NAME) to the head of household?*	*Is (NAME) married to another member of this household?* If yes: record spouse's line no.		*Is (NAME) male or female?*	*How old is (NAME) ?*	*Is (NAME)'s* NATURAL BIOLOGICAL *father still alive?* Tick "BC" if checked identity - birth certificate.		Year died.	*Is (NAME)'s* NATURAL BIOLOGICAL *mother still alive?* Tick "BC" if checked identity - birth certificate.		Year died.	*State highest level of education completed.***		*Is (NAME) still alive?*			*When did (NAME) start staying in this house-hold?*		*Was (NAME) staying here in the same month last year?*	*Does (NAME) STILL stay in this household on a regular basis? (/at death)*	*How many nights is it since (NAME) last slept in this household?*	*How many nights has (NAME) slept here in the last month?*	Codes …	Circle line no if person selected for inter-view***
		See footnote*	Y N	#	M F	Years	Y N DK	BC	Year	Y N DK	BC	Year	Level	Years	Y N DK	Mth	Yr	Mth	Yr	Y N	Y N	#	#		
70 1			1 2		1 2		1 2 8			1 2 8					1 2 8					1 2	1 2				70 1
70 2			1 2		1 2		1 2 8			1 2 8					1 2 8					1 2	1 2				70 2
70 3			1 2		1 2		1 2 8			1 2 8					1 2 8					1 2	1 2				70 3
70 4			1 2		1 2		1 2 8			1 2 8					1 2 8					1 2	1 2				70 4
70 5			1 2		1 2		1 2 8			1 2 8					1 2 8					1 2	1 2				70 5
70 6			1 2		1 2		1 2 8			1 2 8					1 2 8					1 2	1 2				70 6
70 7			1 2		1 2		1 2 8			1 2 8					1 2 8					1 2	1 2				70 7
70 8			1 2		1 2		1 2 8			1 2 8					1 2 8					1 2	1 2				70 8
70 9			1 2		1 2		1 2 8			1 2 8					1 2 8					1 2	1 2				70 9
71 0			1 2		1 2		1 2 8			1 2 8					1 2 8					1 2	1 2				71 0

Tick here, if a further continuation sheet is used: ☐

Enter total numbers of individuals selected for interview:
AGYW (15-24 yrs) ☐
Young men (15-29 yrs) ☐
Older adults (25/30+ yrs) ☐

"Just to make sure that I have a complete listing" ...

Q040 *Are there any other persons such as small children or infants that we have not yet listed?* Number: ☐ Add each in table above. ☐

Q041 *In addition, are there any other people who may not be members of your family, such as lodgers, friends or domestic servants who live here now or have done so in the last three years?* Number: ☐ Add each in table above. ☐

Q042 *Are there any other family members or other people who usually stay elsewhere, but who sleep here from time to time and stayed overnight within the last month?* Number: ☐ Add each in table above. ☐

Q043 *Are there any other people who stayed here for a while but have now passed away?* Number: ☐ Add each in table above. ☐

* CODES FOR Q021: RELATIONSHIP TO HEAD OF HOUSEHOLD

01 Head
02 Wife or husband
03 Son or daughter (natural)
04 Son or daughter-in-law
05 Grandchild
06 Father / mother (natural)
07 Parent-in-law
08 Brother or sister (natural)
09 Stepfather/mother (father's co-wife)
10 Maternal aunt
11 Paternal aunt
12 Maternal uncle
13 Paternal uncle
14/15 Maternal grandfather/mother
16/17 Paternal grandfather/mother
18 Other relative/cousin
19 Adopted/foster child
20 Not related

** CODES FOR Q030 & Q031: LEVEL & YEARS OF EDUCATION

Level:
0 None
1 Primary
2 Secondary

Years: years completed:
0 Less than one year completed
98 Don't know

***ELIGIBLE PERSONS (Q038/Q039)

Usual residents or regular visitors who stayed in the household at least 4 nights last month
11 AGYW (15-24 yrs)
12 Young men (15-29 yrs)
13 Older adults (25/30+ yrs) - 'Yes' households only

‡ Regular visitors are people who, on average, stay in the household at least once a month

FORM A

For peer review only

HOUSEHOLD SOCIO-ECONOMIC STATUS ***Q. No:***

REF.	QUESTIONS & FILTERS	CODING CATEGORIES			SKIP TO
**Q044**	*What is the main source of drinking water for members of your household?*	**Piped into residence**	1		
		**Private tap in yard or plot**	2		
		**Communal tap**	3		
		**Own well or borehole**	4		
		**Other well or borehole**	5		
		**Protected spring**	6		
		**Other (specify)**	10		
**Q045**	*What kind of toilet facility does your household have?*	**Flush toilet**	1		
		**Blair toilet**	2		
		**Pit latrine**	3		
		**Other (specify)**	4		
		**No facilities**	5		**- Q047**
**Q046**	*Is this toilet facility used by members of your household alone, shared with neighbours, or is it communal?*	**Household alone**	1		
		**Shared with neighbours**	2		
		**Communal**	3		
**Q047**	*Does your household have:*		Y	N	
	Electricity?	**Electricity**	1	2	
	A refridgerator?	**Refridgerator**	1	2	
	A radio?	**Radio**	1	2	
	A television?	**Television**	1	2	
**Q048**	**Record house type.**	**Pole and dagga structure**	1		
		**Brick house - thatched roof**	2		
		**Brick house - tiled/sheeting roof**	3		
		**Cabin/other**	8		
**Q049**	**Observe and record type of floor of the main dwelling.**	**Natural floor (earth/sand/dung)**	1		
		**Rudimentary (planks/palm/bamboo)**	2		
		**Finished (wood/cement/carpet...)**	3		
**Q050**	*Does any member of your household own? …*		Y	N	
	A bicycle?	**Bicycle**	1	2	
	A motorcycle?	**Motorcycle**	1	2	
	A car?	**Car**	1	2	
	A tractor?	**Tractor**	1	2	
**Q051**	*How many cattle does your household own?*	**Number of cattle**			

REF.	QUESTIONS & FILTERS	Y	N	DK
**Q052**	*Now I would like to ask you some questions about food: During the last 12 MONTHS, was there a time when:*			
	1) You or others in your household worried about not having enough food to eat because of a lack of money or other resources?	1	2	98
	2) Still thinking about the last 12 MONTHS, was there a time when you or others in your household were unable to eat healthy and nutritious food because of a lack of money or other resources?	1	2	98
	3) Was there a time when you or others in your household ate only a few kinds of food because of lack of money or other resources?	1	2	98
	4) Was there a time when you or others in your household had to skip a meal because there was not enough money or other resources to get food?	1	2	98
	5) Still thinking about the last 12 MONTHS, was there a time when you or others in your household ate less than you thought you should because of a lack of money or other resources?	1	2	98
	6) Was there a time when your household ran out of food because of a lack of money or other resources?	1	2	98
	7) Was there a time when you or others in your household were hungry but did not eat because there was a lack of money or other resources for food?	1	2	98
	8) Was there a time when you or other in your household went without eating for a whole day because of a lack of money or other resources?	1	2	98

For peer review only

For peer review only - http://bmjopen.bmj.com/site/about/guidelines.xhtml

HOUSEHOLD MEMBERS FORM: INDIVIDUAL INTERVIEW PROCESSING *Q. No:*

Study site: **HHID:**

Village: **Growth point:**

Name of household head (R7): **Cell #:**

LINE NO	HOUSEHOLD MEMBERS SELECTED FOR INDIVIDUAL INTERVIEW	CONTACT DETAILS	APPOINTMENTS									IVQ REF.
Q053	Q054	Q055	Q056			Q057			Q058			Q059
From Q019 (ii/iii)	Name	Cell #	1st App't			2nd App't			3rd App't			Note ref.
No.	*Adults (15 yrs +)*		Time	Day/Mth	Res(1)	Time	Day/Mth	Res(2)	Time	Day/Mth	Res(3)	MUT7NO

(1) RESULT CODES

1. Completed
2. Partially completed - questionnaire only
3. Partially completed - BE only
4. Partially completed - questionnaire & BE only (HTC outstanding)
5. Missed appointment - rescheduled
6. Missed appointment - requires follow-up
7. Attended but refused
8. Other (specify)

* Result fields to be completed by supervisors

For peer review only - http://bmjopen.bmj.com/site/about/guidelines.xhtml

Individual questionnaire - baseline survey

For peer review only

INDIVIDUAL QUESTIONNAIRE ***FORM B***

MUT7number:

Questionnaire processing dates:

Consent form
Corrections completed

QUESTIONNAIRE IDENTIFICATION

Q001 **Census district:** ______ **Ward:** [ ]

Q002 **Village:** ______ **Cluster:** [ ]

Q003 **Name of head of household:** ______

Q004 **Category of respondent:** R6 MUTNO [ ]

Q005 **Study site reference:** Date left R6 area R6 site [ ]

Q006 **Household number:** [ ]

Q007 **Line number on household questionnaire:** [ ]

INTERVIEWER VISIT

	1	2	3
**Q008** **Date:**	___	___	___
**Q009** **Time:**	___	___	___
**Q010** **Interviewer:**	___	___	___
**Q011** **Result**:**	[ ]	[ ]	[ ]

Q012 **Interview timing:**
Before BE measurement 1
After BE measurement 2

CHECKED BY SUPERVISOR

Q013 **Signature:** ______

Q014 **Date:** ______

*RESPONDENT CATEGORY

Follow-up respondent (from R6)	1
New respondent: previously under age	2
New respondent: in-migrant (since R6)	5
New respondent: selected but unavailable R6	6
New respondent: selected but refused R6	7
New respondent: household missed R6	9
New respondent: new study site in R7	10
Other (specify) ______	8

**RESULT CODES

Completed	1
Not at home	2
Refused	3
Partially completed	4
Sick/hospital	5
Out-migrated	100
Other (specify) ______	8

For peer review only

INDIVIDUAL QUESTIONNAIRE: **INVITATION TO JOIN THE STUDY**

Explain the purpose of the study - including potential benefits nationally and to the community.
Explain what is involved in participating in the study - show/read invitation letter and consent form.
Ask about and discuss any concerns the respondent might have.
Seek consent to participate in the study - request signature on consent form.

REF.	QUESTIONS & FILTERS	CODING CATEGORIES		SKIP TO
Q101	Indicate whether the respondent wishes to participate in the study.	Yes	1	- Q103
		No	2	
Q102	Indicate the main reason why he/she does not wish to participate.	Insufficient time*	1	
	* Ask for another appointment if reason is insufficient time.	DBS samples	3	
		Information too personal	4	
		Other (specify)	8	
Q103	Record details of others present at this point.		Yes / No	
		Children under 10	1 / 2	
		Husband/wife	1 / 2	
		Other males	1 / 2	
		Other females	1 / 2	

INDIVIDUAL QUESTIONNAIRE: **BACKGROUND CHARACTERISTICS** ***Q. No:***

REF.	QUESTIONS & FILTERS	CODING CATEGORIES		SKIP TO
Q201	Record the current time (24 hour clock).	Hour / Minutes	hr / mins	
Q202	Record sex of respondent.	Male	1	
		Female	2	
Q203	*In what month and year were you born?*		mnth / yr	
Q204	*How old were you at your last birthday?* Check consistency with Q203.	Age in COMPLETED years	yrs	
Q205	*Are you currently enrolled in school full-time?*	Yes	1	- Q208
		No	2	
Q206	*How old were you when you left school?*	Age in completed years	yrs	
		Never been to school	99	- Q209
Q207	*What was your reason for leaving school?*	Insufficient funds	1	
		Found a job	2	
		To go to college or university	3	
		Inadequate exam passes	4	
		Needed to help at home	5	
		Marriage	6	
		Pregnancy/childbirth - voluntarily	7	
		Pregnancy - expelled	8	
		Caught having sex - expelled	9	
	If parents decided, probe for the underlying reason.	Expelled - other reasons	10	
		Finished secondary school	11	
		Other (specify)	18	
Q208	*What is the highest grade of school you have completed?*		Level / Years	
		None	0	
		Primary	1 / 1-7	
	For "years", enter number of years (excl. repeats) at highest level reached.	Secondary	2 / 1-6	- Q210
		Higher	3 / 1-6	- Q210
Q209	*Can you read a letter or newspaper in any language?*	Yes	1	
		No	2	
Q210	*How long have you been living in this homestead?*	Years	yrs	
		Since birth	995	
		Visitor	996	

For peer review only - http://bmjopen.bmj.com/site/about/guidelines.xhtml

INDIVIDUAL QUESTIONNAIRE: **BACKGROUND CHARACTERISTICS** ***Q. No:*** [ ]

REF.	QUESTIONS & FILTERS	CODING CATEGORIES	CODE	SKIP TO
Q211	*How long have you been living in (NAME OF VILLAGE)?*	Years<br>Since birth<br>Visitor	yrs<br>995<br>996	 - Q213
Q212	*What type of place was your previous place of residence?*<br>Record place of current home if the respondent is a visitor.<br>"Roadside" here means a tarred road.<br>Record the name of the place below.<br>________	Large town or city<br>Small town<br>Growth point<br>Commercial estate/mine<br>Roadside business centre<br>Rural business centre<br>Communal/resettlement area	1<br>2<br>3<br>4<br>5<br>6<br>7	
Q213	*Which church denomination do you belong to?*<br>________<br>________	Traditional<br>Pentecostal<br>Methodist<br>Anglican<br>Roman Catholic<br>ZAOGA<br>Apostolic Faith Mission<br>Marange Apostolic<br>Mazowe Apostolic<br>Zviratidzo Apostolic<br>Other Apostolic (specify)<br>Zionist<br>Mughodi<br>Other (specify)<br>None	1<br>2<br>4<br>5<br>6<br>7<br>8<br>9<br>10<br>12<br>13<br>15<br>20<br>17<br>97	
Q214	*In which sector of employment do you work?*<br>________	Estates: tea, coffee, forestry etc<br>Manufacturing or building trade<br>Police or army<br>Teacher: primary school<br>Teacher: secondary school<br>Nurse<br>Services or retail: shops<br>Informal: petty trading (veg etc)<br>Informal: subsistence agriculture<br>Student<br>Unemployed: excl. agriculture<br>Other (specify)	1<br>2<br>3<br>4<br>5<br>6<br>7<br>8<br>9<br>10<br>11<br>12	 <br><br><br><br><br><br>- Q216<br>- Q216<br>- Q216<br>- Q216
Q215	*What type of work do you do?*	Professional or managerial<br>Self-employed: small business<br>Skilled labour<br>Manual/unskilled labour	1<br>2<br>3<br>4	
Q216	*Over the last 12 months, how many times per month, on average, have you had a drink containing alcohol?*	Number of times per month	[ ]	- Q218 if '0'
Q217	*How many drinks containing alcohol do you have on a typical sitting?*	Number of drinks per sitting	[ ]	
Q218	*How many times have you visited a bar or beer-hall in the last month?*	Number of times	[ ]	
Q219	*Do you smoke cigarettes?*	Yes<br>No	1<br>2	
Q220	*Do you take any of these types of drugs for pleasure?*	 Injecting drugs<br>Drugs you smoke<br>Prescription drugs<br>Other drugs you swallow	Yes / No<br>1 / 2<br>1 / 2<br>1 / 2<br>1 / 2	
Q221	*Have you ever been married or in a long-term or cohabiting relationship?*<br>Relationships of 12 months or more should be treated as "long-term".	Yes<br>No	1<br>2	 - Q235
Q222	*How many such relationships have you experienced in your lifetime?*	Include current relationships.	[ ]	
Q223	*How old were you when you first entered such a relationship?*	Age (years)	yrs	

For peer review only

INDIVIDUAL QUESTIONNAIRE: **BACKGROUND CHARACTERISTICS** *Q. No:*

REF.	QUESTIONS & FILTERS	CODING CATEGORIES					SKIP TO
Q224	*Are you currently widowed, divorced or separated from your most recent spouse/partner?*	Widowed	1				- Q235
		Divorced	2				- Q235
		Separated	3				- Q235
		Still in union	4				
Q225	*How many spouses/long-term partners do you have at present?*	(Not zero!)					
	For women, ask how many other wives her husband has.	Ask questions Q230 to Q238 for first spouse, then the second, and so on ...					
Q226	*How old was your partner at his/her last birthday?*	Age in completed years	yrs	yrs	yrs	yrs	
		Don't know	98	98	98	98	
Q227	*Did you and your spouse have an HIV test before you agreed to get married?*	Self only tested	2	2	2	2	
		Partner only tested	3	3	3	3	
		Neither tested	4	4	4	4	- Q229
		Not yet 'married'	5	5	5	5	- Q229
		Both tested seperately	11	11	11	11	
		Both tested together	12	12	12	12	- Q229
Q228	*Did you tell each other your results?*	Yes	1	1	1	1	
	Ask equivalent if only one tested.	No	2	2	2	2	
Q229	*Do you know whether this person has HIV infection now?*	Infected	1	1	1	1	
		Uninfected	2	2	2	2	- Q231
		Prefers not to say	96	96	96	96	- Q231
	If yes, ask for partner's status.	Don't know	98	98	98	98	- Q231
Q230	*Is this person receiving ART (i.e. treatment to prevent AIDS)?*	Yes	1	1	1	1	
		No	2	2	2	2	
		Prefers not to say	96	96	96	96	
		Don't know	98	98	98	98	
Q231	*Has he/she had a sexually transmitted infection (other than HIV) in the last months?*	Yes	1	1	1	1	- Q233 if respondent is male
		No	2	2	2	2	
		Don't know	98	98	98	98	
Q232	*Has he been circumcised?*	Yes - full	1	1	1	1	
	Show respondent pictures to establish whether fully or partially circumcised.	Yes - partial	2	2	2	2	
		No	3	3	3	3	
		Don't know	98	98	98	98	
Q233	*What is the highest grade of school your partner has completed?*	None	0	0	0	0	
		Primary	1	1	1	1	
		Secondary	2	2	2	2	
		Higher	3	3	3	3	
Q234	*In which sector of employment does he/she work?*	Estates	1	1	1	1	
		Manuf'trg/building	2	2	2	2	
		Police/army	3	3	3	3	
		Teacher: primary	4	4	4	4	
		Teacher: secondary	5	5	5	5	
		Nurse	6	6	6	6	
		Services/retail: shops	7	7	7	7	
		Informal: trading	8	8	8	8	- Q235
		Informal: incl agric	9	9	9	9	- Q235
		Student	10	10	10	10	- Q235
		Unemployed: excl. agr	11	11	11	11	- Q235
		Other (specify)	12	12	12	12	
Q235	*What type of work does he/she do?*	Prof/manage't	1	1	1	1	
		Self-employed	2	2	2	2	
		Skilled labour	3	3	3	3	
		Manual/unskilled	4	4	4	4	
Q236	*Which of the following groups exist in your home area and which are you a member of?*			Exist	Member	Rating	
		Church groups					
		Women's groups					
		Co-operative					
	For those where he/she is a member:	Farmers group					
		Burial society					
	Would you say that this group functions: (1) well; (2) OK; or (3) poorly?	Savings club (RCS)					
		Youth group					
		Sports club					
		HIV/AIDS group					
		Political party					
Q237	*What is the principal activity of the group you spend the most time with?*	Code from Q235					
		None	99				

For peer review only

INDIVIDUAL QUESTIONNAIRE: BACKGROUND CHARACTERISTICS Q. No:

REF.	QUESTIONS & FILTERS	CODING CATEGORIES	Yes	No	SKIP TO
Q238	*Do you play one or more of these roles in your community?*	Chief, Headman or Kraal Head	1	2	
		DA or CEO	1	2	
		Political leader (MP, councillor …)	1	2	
		Church leader	1	2	
		Teacher	1	2	
		Traditional healer	1	2	
		Faith healer	1	2	
		Doctor or nurse	1	2	
		Village health worker	1	2	
		Formal sector manager/employer	1	2	
		Other (specify)	1	2	
Q239	*Are you related to a young woman aged between 15-24 years in the following ways?*	Parent	1	2	
		Sister or brother (same parents)	1	2	
		Aunt or uncle	1	2	
		Grandparent	1	2	
		Spouse or regular partner	1	2	
Q240	*Are you related to a young man aged between 15-29 years in the following ways?*	Parent	1	2	
		Sister or brother (same parents)	1	2	
		Aunt or uncle	1	2	
		Grandparent	1	2	
		Spouse or regular partner	1	2	

PSYCHOLOGICAL HEALTH

"In the past 2 weeks, how often have you been bothered by any of the following problems? …"

REF.		Not at all	Several days	> 1/2 days	Nearly every day
Q301	*Little interest or pleasure in doing things*	1	2	3	4
Q302	*Feeling down, depressed, or hopeless*	1	2	3	4
Q303	*Trouble falling asleep, staying asleep, or sleeping too much*	1	2	3	4
Q304	*Feeling tired or having little energy*	1	2	3	4
Q305	*Poor appetite or overeating*	1	2	3	4
Q306	*Feeling bad about yourself - or that you're a failure or have let yourself or your family down*	1	2	3	4
Q307	*Trouble concentrating on things such as reading a newspaper or watching TV*	1	2	3	4
Q308	*Moving or speaking so slowly that other people could have noticed. Or, the opposite - being fidgety or restless that you have been moving around a lot more than usual*	1	2	3	4
Q309	*Thoughts that you would be better off dead or of hurting yourself in some way*	1	2	3	4

REF.		Not hard at all	Some-what hard	Very hard	Extreme-ly hard
Q310	*If you had any of these problems, how hard have these problems made it for you to do your work, take care of things at home, or get along with other people?*	1	2	3	4

For peer review only

INDIVIDUAL QUESTIONNAIRE: **SEXUAL RELATIONSHIPS** ***Q. No:***

REF.	QUESTIONS & FILTERS	CODING CATEGORIES		SKIP TO
**Q401**	**10 minutes of informal discussion first to build rapport, trust & stress absence of prejudice.**<br>**Explain the need to ask questions on the respondent's own experience of sexual relationships.**<br>**Stress the importance of providing accurate information.**<br>**Stress that strict confidentiality will be maintained - request privacy.**			
**Q402**	***How old were you when you had sex for the first time?***<br>**Explain what we mean by "having sex".**	Age in years<br>Not yet had sex	yrs<br>99	- Q404
**Q403**	***What is the main reason you have not yet started to have sexual relations?***	Too young<br>Not met partner<br>Not yet married<br>Risk of pregnancy<br>Risk of HIV/AIDS<br>Other (specify)	1<br>2<br>3<br>4<br>5<br>8	- Q434<br>- Q434<br>- Q434<br>- Q434<br>- Q434<br>- Q434
**Q404**	***How many days is it since you last had sex?***<br>**Skip to Q406 if less than one month.**	 More than one year	days<br>99	
**Q405**	***What is the main reason you are currently abstaining from sexual relations?***<br>**Options 1-4 could refer to the respondent or (if male) to his regular partner.**	Current pregnancy<br>Recent birth<br>Terminal abstinence<br>Self or partner has an STD<br>Currently living apart<br>Risk of catching HIV/AIDS<br>Risk of passing on HIV/AIDS<br>Self or partner has HIV/AIDS<br>Religious reasons<br>Not currently married<br>No partner - although would like one<br>Ill-health<br>Under 1 year since spouse died<br>Don't like / not interested in having sex<br>Other (specify)<br>Don't know	1<br>2<br>3<br>4<br>5<br>6<br>7<br>8<br>9<br>10<br>11<br>12<br>13<br>14<br>20<br>98	
**Q406**	***Did you use condoms throughout the last time you had sex?***	Yes<br>No	1<br>2	
**Q407**	***Do you know whether the last person you had sex with has HIV infection?***<br>**If infected, ask if on ART.**	Infected - on ART<br>Infected - not on ART<br>Uninfected<br>Prefers not to say<br>Don't know	1<br>2<br>3<br>4<br>98	
**Q408**	***If you took a guess, how many partners other than yourself (and any co-wives) do you think your current spouse/partner has had in the last 12 months?***	Number of non-regular partners		
**Q409**	**Indicate data collection method used.**<br>**For 'secret voting':**<br>**(i) explain the procedure and the confidentiality safeguards carefully.**<br>**(ii) establish whether able to use tablet.**	Secret voting<br>Interview (if respondent unable to use tablet)	1<br>2	
**Q410**	***How many different REGULAR sexual partners have you had in your LIFETIME?***<br>***By REGULAR, I mean someone you have been having sex with for a year or more.***	Number of partners	1	

For peer review only - http://bmjopen.bmj.com/site/about/guidelines.xhtml

INDIVIDUAL QUESTIONNAIRE: SEXUAL RELATIONSHIPS **Q. No:**

REF.	QUESTIONS & FILTERS	CODING CATEGORIES		SKIP TO
Q411	*For how many years have you been using condoms EVERY TIME you have sex with a REGULAR partner?*	Years<br>Ask respondent to write "0" if he/she does not use condoms with current regular partner(s).	2	
Q412	*How many different NON-REGULAR sexual partners have you had in your LIFETIME?*	Number of partners	3	
Q413	*For how many years have you been using condoms EVERY TIME you have sex with a NON-REGULAR sexual partner?*	Years<br>Ask respondent to write "0" if he/she doesn't use condoms with non-regular sexual partner(s) now.<br>Ask respondent to write "P" if he/she has never had a non-regular sexual partner.	4	
Q414	*How many different sexual partners have you had in the LAST 12 MONTHS?*	Number of partners	5	
Q415	*How many of these partners were you having sex with for the FIRST TIME?*	Number of new partners in last 12 months (STRESS)	6	
Q416	*How many of the sexual partners that you had in the last 12 months were REGULAR partners?*	Number of regular partners	7	
Q417	*How many of the sexual partners that you had in the last 12 months were NON-REGULAR partners?*<br>Ask respondent to check that this and the previous answer should add up to box #5.	Number of non-regular partners	8	
Q418	*How many sexual relationships do you consider yourself to be involved in at the moment?*	Number of current relationships	9	
Q419	*How many different partners have you had sex with in the last month?*	Number of partners in last month (total)	10	
Q420	Explain that you now wish to ask some questions about the last 3 persons the respondent had sex with.<br>Note: NO time restriction. Stress that these may be people he/she had sex with only once.<br>If secret voting is being used, ask the respondent to enter a "P" in each box if he/she has not had the minimum required number of partners in his/her lifetime.			
			LAST PREVIOUS ...	
Q421	*How many times have you had sexual intercourse with this partner in the last 2 weeks?*	Number of times		1
Q422	*On how many of these occasions did you and your partner use condoms THROUGHOUT?*	Number of times		2
Q423	*Did you use condoms throughout the LAST TIME you had sex with this partner?*	Yes<br>No		3
Q424	*What was the month and year when you had sexual intercourse with this person for the FIRST time?*	Month first then year	mnth mnth mnth<br>yr yr yr	4
Q425	*When was the month and year you LAST had sexual intercourse with this person?*	Month first then year	mnth mnth mnth<br>yr yr yr	5

FORM B

For peer review only - http://bmjopen.bmj.com/site/about/guidelines.xhtml

INDIVIDUAL QUESTIONNAIRE: **SEXUAL RELATIONSHIPS** **_Q. No:_**

REF.	QUESTIONS & FILTERS	CODING CATEGORIES		SKIP TO
Q426	*Are you still having sex with this person?*	Yes<br>No		6
Q427	*Where were you when you had sex with this person for the first time?*<br>If secret voting is being used, show respondent the picture codes. (Code numbers as for Q212).	Code		7
Q428	*How many years old is this person?*	Age in years	yrs yrs yrs	8
Q429	*Is / was this person someone you are / were married to or cohabiting with?*	Yes<br>No		9
Q430	*Is this person married to someone other than yourself?*	Yes<br>No<br>Don't know		10
Q431	*Have you given or received money, goods or services in exchange for sex with this person in the last month?*	Yes<br>No		11
Q432	For men:<br>*Did you have sex with one or more men in the last 12 months?*	Yes<br>No	1<br>2	12
Q433	For men:<br>*Have you EVER been involved in a non-marital relationship of any kind where you gave anything in exchange for sex?*	Yes<br>No	1<br>2	- Q435<br>13
Q434	For women:<br>*Have you EVER been involved in a non-marital relationship of any kind where you received anything in exchange for sex?*	Yes<br>No	1<br>2	14
Q435	Record details of others present at this point.	Children under 10<br>Husband/wife<br>Other males<br>Other females	Y(1) N(2)<br>Y(1) N(2)<br>Y(1) N(2)<br>Y(1) N(2)	

For peer review only - http://bmjopen.bmj.com/site/about/guidelines.xhtml

INDIVIDUAL QUESTIONNAIRE: **HIV PREVENTION METHODS** **Q. No:**

REF.	QUESTIONS & FILTERS	CODING CATEGORIES	SKIP TO
Q501	*"Now I would like to ask you some questions about the methods of HIV prevention that you know about ..."*		
Q502	*What methods of HIV prevention have you heard about?*<br><br>Ask first without probing and then probe for factors that are not mentioned spontaneously.	(Spont / Probed / Where / Who)<br>VMMC<br>PrEP<br>Male condoms<br>Female condoms<br>Spiritual protection<br>Faithfulness to one partner<br>Abstinence<br>HIV testing & counselling<br>Treatment as prevention<br>Other (specify)	
Q503	For each method of HIV prevention mentioned (after probing), except TasP, ask WHERE (venue or activity) they obtained most of their information about this method<br>Enter in the 3rd column in Q502 the code from the list given here that matches the response.	HIV testing service (integrated) 1<br>HIV testing service (stand-alone) 2<br>Health centre (staff and/or posters, leaflets etc.) 3<br>School (lessons, health visitors, clubs) 4<br>Home (visits from health or community worker) 5<br>Workplace 6<br>Church service or meeting 7<br>Shop (e.g. pharmacy) 8<br>Social media 9<br>Internet (other) 10<br>TV, radio or newspaper 11<br>Community group meetings (e.g. womens group, RCS ...) 12<br>Other community meetings 13<br>Word of mouth 14<br>Other (specify) 15	
Q504	For each method of HIV prevention mentioned (after probing), except TasP, WHO was the organisation or person that they got most of their information about this method from).<br>Enter in the 4th column in Q502 the code from the list given here that matches the response.	Ministry of Health staff 1<br>Health centre committee member(s) 2<br>Community health worker 3<br>Community leaders (Chief, counsellor) 4<br>Church leader or members 5<br>Traditional healer 6<br>PSI 7<br>FACT 8<br>Other NGO (specify) 9<br>Other (specify) 12	
Q505	*What were the main APPROACHES used by these organisations to encourage people like you to use each of these methods of HIV prevention?*<br>1. Info'n campaigns - e.g. mass media, posters, leaflets<br>2. Practical skills training - e.g. how to use the method<br>3. Social skills training - e.g. negotiating skills<br>4. Counselling<br>5. Mass distribution - e.g. mobile or outreach services<br>6. Text message reminders<br>7. Financial or material incentives<br>Tick each approach that applies for each method of HIV prevention mentioned in Q502. Enter '99' if not applicable. (e.g. because the HIV prevention method is not known).	(1 / 2 / 3 / 4 / 5 ...)<br>VMMC<br>PrEP<br>Male condoms<br>Female condoms<br>Faithfulness to one partner<br>Abstinence<br>HIV testing & counselling	
Q506*	*Which methods of HIV prevention have you used in your lifetime?*<br><br>Enter '1' is 'Yes'; '2' if No'.<br><br>Probe to find out if more than one method has been used.	(Ever / Now / Provider)<br>VMMC<br>PrEP<br>Male condoms<br>Female condoms<br>Faithfulness to one partner<br>Abstinence<br>TasP<br>Other (specify)	
Q507**	*Which of the methods you know are you using now?*<br>Probe to find out if more than one method is being used.	Enter response in 2nd column in Q506.	
Q508	For methods used, ask for and record the current or most recent provider. Use codes from Q504.	Enter response in 3rd column in Q506.	
	Introduction explaining / defining VMMC & traditional circumcision.<br>For women, go to Q533.		

For peer review only - http://bmjopen.bmj.com/site/about/guidelines.xhtml

INDIVIDUAL QUESTIONNAIRE: **HIV PREVENTION METHODS** *Q. No:*

REF.	QUESTIONS & FILTERS	CODING CATEGORIES		SKIP TO
Q509**	*Have you ever had medical, traditional or religious male circumcision?*	Medical	1	- Q511
		Traditional or Religious (non-medical)	2	
		Both	3	- Q511
		None	8	
Q510	*Have you ever been offered VMMC?*	Yes	1	- Q519
		No	2	- Q519
	If not been offered, ask if heard of VMMC before.	Not heard of VMMC before today	99	- Q519
Q511	*Was it full or partial circumcision?*	Full	1	
	Show pictures to help distinguish.	Partial	2	- Q519
Q512	*Was the medical circumcision a surgical or non-surgical procedure?*	Surgical	1	
		Non-surgical	2	
	e.g. PREPEX is a non-surgical form of VMMC.	Don't know	98	
Q514	*How old were you when you received VMMC?*		yrs	- Q531 if <10 yrs
Q515	*Which of these organisations provided you with the VMMC service?*		Yes / No	
		Ministry of Health	1 / 2	
		PSI	1 / 2	
		ITEC	1 / 2	
		Other (specify)	1 / 2	
Q516	*How many visits to this place did you have to make for this procedure?*	Number of visits		
	i.e. from the first appointment to the final check-up. Record date of first appointment.	Date of first appointment	mth / yr	
Q517	*What costs did you incur when you had the VMMC done?*	Medical fees	$	
		Transport costs	$	
		Accommodation costs	$	
		Foregone income	$	
		Other costs (specify)	$	
Q518	*After you received VMMC, did you:*		More / Same / Less	
	(1) Increase, decrease or not change your number of sexual partners?		1 / 2 / 3	
	(2) Use condoms more, less or the same as before?		1 / 2 / 3	
	(3) Use condoms consistently with casual partners?	Yes	1	- Q529
		No	2	- Q529
		NA - no casual partners	99	- Q529
Q519	*Please tell me whether you strongly disagree, disagree, neither agree nor disagree, agree or strongly agree with the following statements …*		SD / D / N / A / SA	
	(1) I am confident I can get VMMC if I wanted to		1 / 2 / 3 / 4 / 5	
	(2) I am confident that I can get VMMC even if my partner disapproves of this		1 / 2 / 3 / 4 / 5	
	(3) I am confident I can get VMMC even if my friends disapprove of this		1 / 2 / 3 / 4 / 5	
	(4) I am confident I can get VMMC even if my parents and family elders disapprove		1 / 2 / 3 / 4 / 5	
Q520	*Are you able to discuss getting VMMC with your partner?*	Yes - already done so	1	
		Yes - not done already but can do this	2	
		No	3	
		No regular partner	99	- Q522
Q521	*Would your partner disapprove if you had VMMC?*	Yes	1	
		No	2	
		Don't know	3	
Q522**	*If you wanted to get VMMC yourself, do you know a place where someone like you can easily have it done?*	Yes	1	
		No	2	
Q523*	*If you wanted to get VMMC yourself, how easy would it be for you to access the service?*	Very easy	1	
		Easy	2	
		Neither easy nor difficult	3	
	Ask for an answer on a scale of 5 - from 1 (very easy) to 5 (very difficult).	Difficult	4	
		Very difficult	5	
		Don't know	98	

For peer review only - http://bmjopen.bmj.com/site/about/guidelines.xhtml

INDIVIDUAL QUESTIONNAIRE: **HIV PREVENTION METHODS** ***Q. No:*** ☐

REF.	QUESTIONS & FILTERS	CODING CATEGORIES					SKIP TO
Q524	*What factors make it impractical or unsuitable for someone like you to access VMMC services?*<br><br>Ask first without probing and then probe for factors that are not mentioned spontaneously.<br>________________				Spont	Probed	
		High costs (including loss of income)			☐	☐	
		Inability to work during/after procedure			☐	☐	
		Healthcare providers are female			☐	☐	
		Lack of privacy / confidentiality			☐	☐	
		Limited opening hours			☐	☐	
		Distance / travel difficulties			☐	☐	
		Respondent is HIV+ (not suitable for PLHIV)			☐	☐	- Q532
		Other (specify)			☐	☐	
Q525**	*Do you want to get VMMC if the service was freely accessible to you?*	Yes			1	☐	
		No			2	☐	
		Not sure			8	☐	
Q526*	*How definitely do you want to get VMMC if the service was freely accessible to you?*<br><br>Ask for an answer on a scale of 5 - from 1 (definitely) to 5 (definitely not).	Definitely			1	☐	
		Probably			2	☐	
		No opinion			3	☐	
		Probably not			4	☐	
		Definitely not			5	☐	
Q527	*Do you plan to get VMMC?*	Yes			1	☐	- Q529
		No			2	☐	- Q529
		Don't know			98	☐	
Q528	*How soon do you plan to have VMMC?*	Number of months / years			☐ mths	☐ yrs	
Q529	*What factors were/are important in encouraging or discouraging you to have VMMC?*<br><br>For each factor given, ask if this was a positive (encouraging) or negative (discouraging) factor.<br><br>Ask first without probing and then probe for factors that are not mentioned spontaneously.<br><br>Tick probed box to indicate probed responses.<br>________________		Pos	Neg	Neutral	Probed	
		HIV/STI/HPV protection (for myself)	1	2	8	☐	
		HIV/STI/HPV protection (for my partner(s))	1	2	8	☐	
		Method of HIV prevention I can control	1	2	8	☐	
		Pain	1	2	8	☐	
		Operation cannot be reversed	1	2	8	☐	
		Make me feel responsible or ashamed	1	2	8	☐	
		Make me feel more manly	1	2	8	☐	
		Own sexual pleasure (better/worse)	1	2	8	☐	
		Partner's sexual pleasure (better/worse)	1	2	8	☐	
		Able to have erection for longer/shorter	1	2	8	☐	
		More able to attract partners	1	2	8	☐	
		Risk of getting an infection	1	2	8	☐	
		Penis will be cleaner / look better	1	2	8	☐	
		Using other prevention method (note method)	1	2	8	☐	
		Abstaining or faithful to one trusted partner	1	2	8	☐	
		Other (specify) ____________	1	2	8	☐	
Q530	*Which people's views were/are important in encouraging or discouraging you to have VMMC?*<br><br>Instructions as for last question.		Pos	Neg	Neutral	Probed	
		Religious leaders (religious beliefs)	1	2	8	☐	
		Parents' or family elders' approval/views	1	2	8	☐	
		Partner's views	1	2	8	☐	
		Friends views	1	2	8	☐	
		Community views	1	2	8	☐	
		Other (specify) ____________	1	2	8	☐	
Q531	*How far away is it from where you live to the nearest place where VMMC services are provided?*	Distance in kms				☐ kms	
		Don't know			98	☐	
Q532	*By how much do you think VMMC reduces a man's risk of getting HIV infection?*<br>Ask for or convert response to a percent.	Percent reduction				☐ %	
Q533	*Have many of your friends (or their male partners) had VMMC?*	Yes			1	☐	
		No			2	☐	
		Don't know			98	☐	
Q534	*If you have a son who is a teenager or a young man, do you think it would be a good idea if he got VMMC?*			Teen-ager		Young man	
		Yes		1		1	- Q536 if male respondent
		No		2		2	
		Don't know		98		98	
Q535	*Do you think it is a good thing for your male partner to have VMMC?*	Yes			1	☐	
		No			2	☐	
		Don't know			98	☐	

For peer review only - http://bmjopen.bmj.com/site/about/guidelines.xhtml

INDIVIDUAL QUESTIONNAIRE: **HIV PREVENTION METHODS** *Q. No:*

REF.	QUESTIONS & FILTERS	CODING CATEGORIES	SKIP TO
Q536*	*The following statement is correct. Did you know this already?*<br>'Pre-exposure prophylaxis (PrEP) is when someone who does not have HIV takes a pill on an ongoing basis to prevent them getting HIV. Most people who use PrEP take a pill everyday. PrEP needs to be taken BEFORE sex for it to be effective.'	I knew this already 1<br>I wasn't sure about this 2<br>I didn't know about this 3<br>I don't understand this 4	 <br>- Q566<br>- Q566
Q537**	*Have you ever taken PrEP?*	Yes 1<br>No 2	- Q539
Q538	*Have you ever been offered PrEP?*	Yes 1<br>No 2	 - Q548
Q539**	*Are you taking PrEP currently?*	Yes 1<br>No 2	 - Q545
Q540	*When did you start taking PrEP this time?*<br>Emphasise this is for the current period of use.	mth yr	
Q541	*Which of these organisations is providing you with PrEP?*	(Yes / No)<br>Ministry of Health 1 2<br>PSI 1 2<br>FACT 1 2<br>Other (specify) 1 2	
Q542	*How often have you taken PrEP in the last month?*	Every day 1<br>Most days 2<br>Occasionally 3<br>Never 4	- Q546
Q543	*What was the longest number of days that you didn't take the PrEP pills in the last month?*	Number of days	
Q544	*How many days in the last week (7 days) did you take the PrEP pills?*	Number of days	- Q546 if all 7 days
Q545	*Why did you stop using PrEP / not use PrEP on some days?*	High costs 1<br>Forgot to take pills 2<br>Ran out of pills 3<br>Judgemental staff / stigma 4<br>Lack of confidentiality 5<br>Limited opening hours 6<br>Distance / travel difficulties 7<br>Sexual partner(s) disapproved 8<br>Parents / elders / friends disapproved 9<br>Using another prevention method (specify method 10<br>Not currently sexually active 11<br>Abstaining or faithful to one trusted partner 12<br>Experienced side effects 13<br>Stock-outs of PrEP drugs at clinic 14<br>Other (specify) 15<br>Don't know or no particular reason 98	- Q548
Q546	*How often do you have a meal at the same time when you take PrEP?*	Always 1<br>Most of the time 2<br>Sometimes 3<br>Never 4	
Q547	*After you started taking PrEP, did you:*<br>(1) Increase, decrease or not change your number of sexual partners?<br>(2) Use condoms more, less or the same as before?<br>(3) Use condoms consistently with casual partners?	(More / Same / Less)<br>(1) 1 2 3<br>(2) 1 2 3<br>(3) Yes 1<br>No 2<br>NA - no casual partners 99	
Q548	*Please tell me whether you strongly disagree, disagree, neither agree nor disagree, agree or strongly agree with the following statements …*<br>(1) I am confident I can use PrEP if I wanted to<br>(2) I am confident I can use PrEP even if I have to take it every day<br>(3) I am confident I can use PrEP even if I have to take it always after a meal<br>(4) I am confident I can use PrEP even if I have to hide it from my partner<br>(5) I am confident I can use PrEP even if my friends disapprove of this<br>(6) I am confident I can use PrEP even if my parents and family elders disapprove<br>(7) I am confident I can use PrEP even if my community would think I have HIV	SD D N A SA<br>(1) 1 2 3 4 5<br>(2) 1 2 3 4 5<br>(3) 1 2 3 4 5<br>(4) 1 2 3 4 5<br>(5) 1 2 3 4 5<br>(6) 1 2 3 4 5<br>(7) 1 2 3 4 5	
Q549	*Have you ever received instructions or counselling on how to use PrEP?*	Yes 1<br>No 2	

For peer review only

INDIVIDUAL QUESTIONNAIRE: **HIV PREVENTION METHODS** ***Q. No:***

REF.	QUESTIONS & FILTERS	CODING CATEGORIES		SKIP TO
Q550	*Are you able to discuss taking PrEP with your partner?*	Yes - already done so<br>Yes - not done already but can do this<br>No current sexual partner	1<br>2<br>8	
Q551	*Would / does you partner disapprove if you use PrEP?*	Yes<br>No<br>No current sexual partner	1<br>2<br>8	
Q552**	*If / when you want to use PrEP, do you know a place where someone like you can easily get it?*	Yes<br>No	1<br>2	
Q553*	*If / when you want to take PrEP yourself, how easy is it for you to access the service?*<br>Ask for an answer on a scale of 5 - from 1 (very easy) to 5 (very difficult).	Very easy<br>Easy<br>Neither easy nor difficult<br>Difficult<br>Very difficult<br>Don't know	1<br>2<br>3<br>4<br>5<br>98	
Q554	*What factors make it impractical or unsuitable for someone like you to access PrEP services?*<br>Ask first without probing and then probe for factors that are not mentioned spontaneously.	High costs<br>Lack of privacy / confidentiality<br>Embarrassed to go / ask<br>Limited opening hours<br>Distance / travel difficulties<br>Not suitable for men<br>Below eligible age for PrEP<br>Other (specify)<br>Not currently sexually active<br>Respondent is HIV+ (not suitable for PLHIV)	Spont / Probed	Q559 - if on PrEP now<br><br>- Q557 (Not currently sexually active)<br>- Q562 (Respondent is HIV+)
Q555**	*Do you want to use PrEP if it was freely accessible to you?*	Yes<br>No<br>Not sure	1<br>2<br>8	
Q556*	*How definitely do you want to use PrEP if it was freely accessible to you?*<br>Ask for an answer on a scale of 5 - from 1 (definitely) to 5 (definitely not).	Definitely<br>Probably<br>No opinion<br>Probably not<br>Definitely not	1<br>2<br>3<br>4<br>5	
Q557	*Do you plan to start using PrEP?*	Yes<br>No<br>Don't know	1<br>2<br>98	- Q559 (No)
Q558	*How soon do you plan to start using PrEP?*	Number of months	mths	
Q559	*What factors were/are important in encouraging or discouraging you to use PrEP?*<br>For each factor given, ask if this was a positive (encouraging) or negative (discouraging) factor.<br>Ask first without probing and then probe for factors that are not mentioned spontaneously.<br>Tick probed box to indicate probed responses.	See table below		
Q560	*Which people's views were/are important in encouraging or discouraging you to use PrEP?*<br>Instructions as for last question.<br>For partner/friends' views - if mentioned spontaneously, ask for the reason.	See table below		
Q561	*How far away is it from where you live to the nearest place where PrEP services are provided?*	Distance in kms	kms	
Q562	*By how much do you think PrEP reduces a person's risk of getting HIV infection?*<br>Ask for or convert response to a percent.	Percent reduction	%	
Q563	*Are many of your friends (or their partners) using PrEP?*	Yes<br>No<br>Don't know	1<br>2<br>98	

Q559:

Factor	Pos	Neg	Neutral	Probed
HIV/STI protection	1	2	8	
Method of HIV prevention I can control	1	2	8	
Allow me to become pregnant if I want	1	2	8	
Possible side effects	1	2	8	
Don't think PrEP works	1	2	8	
High costs involved	1	2	8	
Make me feel responsible or ashamed	1	2	8	
Inconvenient to take pills daily	1	2	8	
Using other prev'n method (note method)	1	2	8	
Faithful to one trusted partner	1	2	8	
Not currently sexually active	1	2	8	
Other (specify) ______________	1	2	8	

Q560:

Factor	Pos	Neg	Neutral	Probed
Religious leaders (religious beliefs)	1	2	8	
Parents' or family elders' approval/views	1	2	8	
Partner(s) will think I have HIV	1	2	8	
Partner's views (other)	1	2	8	
Friends / community think I have HIV	1	2	8	
Friends / community views (other)	1	2	8	
Other (specify) ______________	1	2	8	

For peer review only - http://bmjopen.bmj.com/site/about/guidelines.xhtml

REF.	QUESTIONS & FILTERS	CODING CATEGORIES			SKIP TO
			Teen-ager	Young woman	
Q564	*If you have or had a daughter (or sister) who is a teenager or a young woman and she started having sex before getting married, do you think it would be a good thing for her to use PrEP?*	Yes<br>No<br>Don't know	1<br>2<br>98	1<br>2<br>98	
			Non-reg	Regular	
Q565	*If your sexual partner wanted to use PrEP, would you agree to him or her doing this?*<br><br>Ask first for non-regular partners (if any).<br>Then for regular partners.	Yes<br>No<br>Don't know<br>NA	1<br>2<br>98<br>99	1<br>2<br>98<br>99	
Q566**	*How often have you used a male condom when you had sexual intercourse with your REGULAR partner or partners in the last 2 weeks?*<br><br>Emphasise asking about male condoms only here.	Every time<br>Most times<br>Occasionally<br>Never<br>NA - no regular partner	1<br>2<br>3<br>4<br>99		
Q567**	*How often have you used a male condom when you had sexual intercourse with NON-REGULAR partners in the last 2 weeks?*	Every time<br>Most times<br>Occasionally<br>Never<br>NA - no non-regular partners	1<br>2<br>3<br>4<br>99		- Q569 if 'every time' for reg & non-regs
Q568	*Why did you not use male condoms on some occasions when you had sex in the last 2 weeks?*<br><br>__________<br>__________	High costs<br>Judgemental staff / stigma<br>Lack of privacy / confidentiality<br>Limited times when accessible<br>Distance / travel difficulties to access<br>Sexual partner(s) disapproved<br>Parents / elders / friends disapproved<br>Condoms reduce pleasure of sex<br>Condoms not effective<br>Using different prevention method (specify metho<br>Faithful to one trusted partner<br>Other (specify)<br>None<br>Don't know	1<br>2<br>3<br>4<br>5<br>6<br>7<br>8<br>9<br>10<br>11<br>12<br>96<br>98		
Q569	*Has a male condom ever broken when you were using it?*	Yes<br>No<br>NA - not using male condoms	1<br>2<br>99		 - Q571<br>- Q571
Q570	*What do you do when male condoms break?*<br><br>__________	Replace the condom with a new one<br>Continue without replacing the condom<br>Other (specify)	1<br>2<br>99		
Q571	*After you started using male condoms, did you increase or decrease your number of sexual partners?*	Increase<br>Decrease<br>No change<br>Never used male condoms	1<br>2<br>8<br>99		

Q572	*Please tell me whether you strongly disagree, disagree, neither agree nor disagree, agree or strongly agree with the following statements …*	SD	D	N	A	SA
	(1) I am confident I can use male condoms if I wanted to	1	2	3	4	5
	(2) I am confident I can use male condoms even if I have to use them every time	1	2	3	4	5
	(3) I am confident I can use male condoms even if my partner dislikes/disapproves	1	2	3	4	5
	(4) I am confident I can use male condoms even if I'm drunk or have taken drugs	1	2	3	4	5
	(5) I am confident I can use male condoms even if my friends disapprove	1	2	3	4	5
	(6) I am confident I can use male condoms even if my parents and family elders disapprove	1	2	3	4	5

REF.	QUESTIONS & FILTERS	CODING CATEGORIES		SKIP TO
Q573	*Have you ever received instructions or counselling on how to use male condoms?*	Yes<br>No	1<br>2	
Q574	*Are you able to discuss using male condoms with your REGULAR partner?*	Yes - already done so<br>Yes - not done already but can do this<br>No<br>Never had sex<br>No regular partner	1<br>2<br>3<br>97<br>99	 <br><br>- Q580<br>- Q577
Q575	*Would / does your regular partner disapprove if you use male condoms?*	Yes<br>No<br>Don't know	1<br>2<br>98	
Q576	*If your regular partner doesn't want to use male condoms, are you able to refuse to have sex with him/her?*	Yes - already done so<br>Yes - not done already but can do this<br>No<br>Don't know	1<br>2<br>3<br>98	
Q577	*Are you able to discuss using male condoms with your NON-REGULAR partner(s)?*	Yes - already done so<br>Yes - not done already but can do this<br>No<br>No non-regular partners	1<br>2<br>3<br>99	 <br><br>- Q580

For peer review only

For peer review only - http://bmjopen.bmj.com/site/about/guidelines.xhtml

INDIVIDUAL QUESTIONNAIRE: **HIV PREVENTION METHODS** *Q. No:*

REF.	QUESTIONS & FILTERS	CODING CATEGORIES	SKIP TO
Q578	*Would / do your non-regular partners disapprove if you use male condoms?*	Yes 1<br>No 2<br>Don't know 98	
Q579	*If a non-regular partner doesn't want to use male condoms, are you able to refuse to have sex with him/her?*	Yes - already done so 1<br>Yes - not done already but can do this 2<br>No 3	
Q580**	*If / when you want to use male condoms, do you know a place where someone like you can easily get them?*	Yes 1<br>No 2	
Q581*	*If / when you want to use male condoms yourself, how easy is it for you to access them?*<br><br>Ask for an answer on a scale of 5 - from 1 (very easy) to 5 (very difficult).	Very easy 1<br>Easy 2<br>Neither easy nor difficult 3<br>Difficult 4<br>Very difficult 5	
Q582	*Do you feel able to access male condoms from the following places?*<br><br>Ask each in turn.	(Yes / No / DK)<br>Health clinic 1 2 98<br>Community-based distributor 1 2 98<br>Bars or beer halls 1 2 98<br>Shops 1 2 98<br>Your sexual partner(s) 1 2 98<br>Friends 1 2 98<br>Other (specify) 1 2 98	
Q583	*What factors make it impractical or unsuitable for someone like you to access male condoms?*<br><br>Ask first without probing and then probe for factors that are not mentioned spontaneously.	(Spont / Probed)<br>High costs<br>Lack of privacy / confidentiality<br>Embarrassed to go / ask<br>Limited opening hours<br>Distance / travel difficulties<br>Other (specify)	
Q584**	*Do you want to use male condoms with your regular partner(s) if they were freely accessible to you?*<br>Stress: no right or wrong answer.	Yes 1<br>No 2<br>Not sure 98<br>NA - no regular partner 99	- Q587
Q585*	*How definitely do you want to use male condoms with your regular partner(s) if they were freely accessible to you?*<br>Ask for an answer on a scale of 5 - from 1 (definitely) to 5 (definitely not).	Definitely 1<br>Probably 2<br>No opinion 3<br>Probably not 4<br>Definitely not 5	
Q586	*Do you plan to use male condoms the next time you have sex with your regular partner?*	Yes 1<br>No 2<br>Don't know 98	
Q587**	*Do you want to use male condoms with your non-regular partner(s) if they were freely accessible to you?*<br>Stress: no right or wrong answer.	Yes 1<br>No 2<br>Not sure 98<br>NA - no non-regular partner(s) 99	- Q590
Q588*	*How definitely do you want to use male condoms with your non-regular partner(s) if they were freely accessible to you?*<br>Ask for an answer on a scale of 5 - from 1 (definitely) to 5 (definitely not).	Definitely 1<br>Probably 2<br>No opinion 3<br>Probably not 4<br>Definitely not 5	
Q589	*Do you plan to use male condoms the next time you have sex with a non-regular partner?*<br>Stress: no right or wrong answer.	Yes 1<br>No 2<br>Don't know 98	
Q590	*What factors were/are important in encouraging or discouraging you to use male condoms?*<br><br>For each factor given, ask if this was a positive (encouraging) or negative (discouraging) factor.<br><br>Ask first without probing and then probe for factors that are not mentioned spontaneously. Tick probed box to indicate probed responses.	(Pos / Neg / Neutral / Probed)<br>HIV/STI protection 1 2 8<br>HIV/STI protection (for my partner(s)) 1 2 8<br>Method of HIV prevention I can control 1 2 8<br>Reduce risk of pregnancy (self/partner) 1 2 8<br>Own sexual pleasure (better/worse) 1 2 8<br>Partner's sexual pleasure (better/worse) 1 2 8<br>Make me feel responsible or ashamed 1 2 8<br>Faithful to one trusted partner 1 2 8<br>Using other prev'n method (note method) 1 2 8<br>Not currently sexually active 1 2 8<br>Other (specify) ______________ 1 2 8	

For peer review only

For peer review only - http://bmjopen.bmj.com/site/about/guidelines.xhtml

INDIVIDUAL QUESTIONNAIRE: **HIV PREVENTION METHODS** *Q. No:*

REF.	QUESTIONS & FILTERS	CODING CATEGORIES					SKIP TO
Q591	*Which people's views were/are important in encouraging or discouraging you to use male condoms?*		Pos	Neg	Neutral	Probed	
	Instructions as for last question.	Religious leaders (religious beliefs)	1	2	8		
	For partner/friends' views - if mentioned spontaneously, ask for the reason.	Parents' or family elders' approval/views	1	2	8		
		Partner(s) will think I have HIV	1	2	8		
		Partner will think I have other partners	1	2	8		
		Partner's views (other)	1	2	8		
		Friends / community think I have HIV	1	2	8		
		Friends / community views (other)	1	2	8		
		Other (specify) ______________	1	2	8		
Q592	*By how much do you think male condoms reduce a person's risk of getting HIV infection?* Ask for or convert response to a percent.	Percent reduction				%	
Q593	*How far away is it from where you live to the nearest place where male condoms can be obtained?*	Distance in kms				kms	
Q594	*Are many of your friends (or their partners) using male condoms?*	Yes			1		
		No			2		
		Don't know			98		
Q595	*In what circumstances do you think it is acceptable for a husband and wife to use condoms?*				Yes	No	
		Always			1	2	
		If one of them is HIV+			1	2	
		If one spouse has other partners			1	2	
		If one spouse has an STD			1	2	
		To avoid pregnancy			1	2	
	______________________________	Other (specify)			1	2	
Q596	*If you have or had a daughter (or sister) who was a teenager or young woman and she started having sex before getting married, do you think it would be a good thing for her to use male condoms with her partner?*			Teen-ager		Young woman	
		Yes		1		1	
		No		2		2	
		Don't know		98		98	
	"Now I would like to ask you some questions about FEMALE condoms ... "						
Q597**	*How often have you used a female condom when you had sexual intercourse with your REGULAR partner or partners in the last 2 weeks?*	Every time			1		
		Most times			2		
		Occasionally			3		
		Never			4		
		NA - no regular partner			99		
		Not heard of female condoms			97		- Q59929
Q598**	*How often have you used a female condom when you had sexual intercourse with NON-REGULAR partners in the last 2 weeks?*	Every time			1		
		Most times			2		
		Occasionally			3		
		Never			4		
		NA - no non-regular partners			99		
Q599	*Why did you not use female condoms on some occasions when you had sex in the last 2 weeks?*	High costs			1		
		Difficult to use			2		
		Judgemental staff / stigma			3		
		Lack of privacy / confidentiality			4		
		Limited times when accessible			5		
		Distance / travel difficulties			6		
		Sexual partner(s) disapproved			7		
		Parents / elders / friends disapproved			8		
		Female condoms reduce pleasure of sex			9		
		Female condoms not effective			10		
	______________________________	Using different prevention method (specify metho			11		
		Faithful to one trusted partner			12		
	______________________________	Other (specify)			13		
		None			96		
		Don't know			98		
Q59901	*Has a female condom ever broken when you were using it?*	Yes			1		
		No			2		- Q59903
		NA - not using male condoms			99		- Q59903
Q59902	*What do you do when female condoms break?*	Replace the condom with a new one			1		
		Continue without replacing the condom			2		
	______________________________	Other (specify)			99		
Q59903	*After you started using female condoms, did you increase or decrease your number of sexual partners?*	Increase			1		
		Decrease			2		
		No change			8		
		Never used female condoms			99		

For peer review only

For peer review only - http://bmjopen.bmj.com/site/about/guidelines.xhtml

INDIVIDUAL QUESTIONNAIRE: **HIV PREVENTION METHODS** **Q. No:**

REF.	QUESTIONS & FILTERS	CODING CATEGORIES		SKIP TO
Q59904	*Please tell me whether you strongly disagree, disagree, neither agree nor disagree, agree or strongly agree with the following statements …*	SD / D / N / A / SA		
	(1) I am confident I can use female condoms if I wanted to	1 / 2 / 3 / 4 / 5		
	(2) I am confident I can use female condoms even if I have to use them every time	1 / 2 / 3 / 4 / 5		
	(3) I am confident I can use female condoms even if my partner dislikes/disapproves	1 / 2 / 3 / 4 / 5		
	(4) I am confident I can use female condoms even if I'm drunk or have taken drugs	1 / 2 / 3 / 4 / 5		
	(5) I am confident I can use female condoms even if my friends disapprove of this	1 / 2 / 3 / 4 / 5		
	(6) I am confident I can use female condoms even if my parents and family elders disapprove	1 / 2 / 3 / 4 / 5		
Q59905	*Have you ever received instructions or counselling on how to use female condoms?*	Yes	1	
		No	2	
Q59906	*Are you able to discuss using female condoms with your REGULAR partner?*	Yes - already done so	1	
		Yes - not done already but can do this	2	
		No	3	
		No regular partner	8	- Q59909
		Never had sex	99	- Q59912
Q59907	*Would / does your regular partner disapprove if you use female condoms?*	Yes	1	
		No	2	
		Don't know	98	
Q59908	*If your regular partner doesn't want to use female condoms, are you able to refuse to have sex with him/her?*	Yes - already done so	1	
		Yes - not done already but can do this	2	
		No	3	
		Don't know	98	
Q59909	*Are you able to discuss using female condoms with your NON-REGULAR partner(s)?*	Yes - already done so	1	
		Yes - not done already but can do this	2	
		No	3	
		No non-regular partners	99	- Q59912
Q59910	*Would / do your non-regular partners disapprove if you use female condoms?*	Yes	1	
		No	2	
		Don't know	98	
Q59911	*If a non-regular partner doesn't want to use female condoms, are you able to refuse to have sex with him/her?*	Yes - already done so	1	
		Yes - not done already but can do this	2	
		No	3	
Q59912*	*If / when you want to use female condoms, do you know a place where someone like you can easily get them?*	Yes	1	
		No	2	
Q59913*	*If / when you want to use female condoms yourself, how easy is it for you to access them?*	Very easy	1	
		Easy	2	
		Neither easy nor difficult	3	
	Ask for an answer on a scale of 5 - from 1 (very easy) to 5 (very difficult).	Difficult	4	
		Very difficult	5	
Q59914	*Do you feel able to access female condoms from the following places?*		Yes / No / DK	
	Ask each in turn.	Health clinic	1 / 2 / 98	
		Community-based distributor	1 / 2 / 98	
		Bars or beer halls	1 / 2 / 98	
		Shops	1 / 2 / 98	
		Your sexual partner(s)	1 / 2 / 98	
		Friends	1 / 2 / 98	
		Other (specify)	1 / 2 / 98	
Q59915*	*What factors make it impractical or unsuitable for someone like you to access female condoms?*		Spont / Probed	
	Ask first without probing and then probe for factors that are not mentioned spontaneously.	High costs		
		Lack of privacy / confidentiality		
		Embarrassed to go / ask		
		Limited opening hours		
		Distance / travel difficulties		
		Other (specify)		
Q59916	*Do you want to use female condoms with your regular partner(s) if they were freely accessible to you?*	Yes	1	
		No	2	
		Not sure	98	
		NA - no regular partner	99	- Q59919
Q59917*	*How definitely do you want to use female condoms with your regular partner(s) if they were freely accessible to you?*	Definitely	1	
		Probably	2	
		No opinion	3	
	Ask for an answer on a scale of 5 - from 1 (definitely) to 5 (definitely not).	Probably not	4	
		Definitely not	5	

For peer review only

INDIVIDUAL QUESTIONNAIRE: **HIV PREVENTION METHODS** ***Q. No:*** ______

REF.	QUESTIONS & FILTERS	CODING CATEGORIES	CODE	SKIP TO
Q59918*	*Do you plan to use female condoms the next time you have sex with your regular partner?*	Yes<br>No<br>Don't know	1<br>2<br>98	
Q59919*	*Do you want to use female condoms with your non-regular partner(s) if they were freely accessible to you?*	Yes<br>No<br>Not sure<br>NA - no non-regular partner	1<br>2<br>98<br>99	 <br><br>- Q59922
Q59920*	*How definitely do you want to use female condoms with your non-regular partner(s) if they were freely accessible to you?*<br>Ask for an answer on a scale of 5 - from 1 (definitely) to 5 (definitely not).	Definitely<br>Probably<br>No opinion<br>Probably not<br>Definitely not	1<br>2<br>3<br>4<br>5	
Q59921*	*Do you plan to use female condoms the next time you have sex with a non-regular partner?*	Yes<br>No<br>Don't know	1<br>2<br>98	

Q59922 *What factors were/are important in encouraging or discouraging you to use female condoms?*

For each factor given, ask if this was a positive (encouraging) or negative (discouraging) factor.

Ask first without probing and then probe for factors that are not mentioned spontaneously.

Tick probed box to indicate probed responses.

	Pos	Neg	Neutral	Probed
HIV/STI protection	1	2	8	
HIV/STI protection (for my partner(s))	1	2	8	
Method of HIV prevention I can control	1	2	8	
Reduce risk of pregnancy (self/partner)	1	2	8	
Own sexual pleasure (better/worse)	1	2	8	
Partner's sexual pleasure (better/worse)	1	2	8	
Make me feel responsible or ashamed	1	2	8	
Difficult to use	1	2	8	
Fear of losing condom inside vagina	1	2	8	
Faithful to one trusted partner	1	2	8	
Using other prev'n method (note method)	1	2	8	
Not currently sexually active	1	2	8	
Other (specify) ______________	1	2	8	

Q59923 *Which people's views were/are important in encouraging or discouraging you to use female condoms?*

Instructions as for last question.

For partner/friends' views - if mentioned spontaneously, ask for the reason.

	Pos	Neg	Neutral	Probed
Religious leaders (religious beliefs)	1	2	8	
Parents' or family elders' approval/views	1	2	8	
Partner(s) will think I have HIV	1	2	8	
Partner will think I have other partners	1	2	8	
Partner's views (other)	1	2	8	
Friends / community think I have HIV	1	2	8	
Friends / community views (other)	1	2	8	
Other (specify) ______________	1	2	8	

Q59923 *What are the main reasons that would/did*
Q59924 *By how much do you think female condoms reduce a person's risk of getting HIV infection?*
Ask for or convert response to a percent.

Percent reduction — Spont / Probed: ____ %

Q59925 *How far away is it from where you live to the nearest place where female condoms can be obtained?*

Distance in kms: ____ kms

REF.	QUESTIONS & FILTERS	CODING CATEGORIES	CODE	SKIP TO
Q59926	*Are many of your friends (or their partners) using female condoms?*	Yes<br>No<br>Don't know	1<br>2<br>98	

Q59927 *In what circumstances do you think it is acceptable for a husband and wife to use female condoms?*

	Yes	No
Always	1	2
If one of them is HIV+	1	2
If one spouse has other partners	1	2
If one spouse has an STD	1	2
To avoid pregnancy	1	2
Other (specify)	1	2

Q59928* *If you have or had a daughter (or sister) who was teenager or young woman and she started having sex before getting married, do you think it would be a good thing for her to use female condoms with her partner?*

	Teen-ager	Young woman
Yes	1	1
No	2	2
Don't know	98	98

REF.	QUESTIONS & FILTERS	CODING CATEGORIES	CODE	SKIP TO
Q59929*	*Are you currently sticking to one regular sexual partner or not having sex?*<br>Stress: no right or wrong answer.	Yes - one regular partner<br>Yes - not having sex<br>Neither	1<br>2<br>3	 <br>- Q59931
Q59930	*For how long have you been sticking to one regular partner or not having sex?*	Number or months or years	____ yrs ____ mths	

For peer review only

INDIVIDUAL QUESTIONNAIRE: **HIV PREVENTION METHODS** ***Q. No:***

REF.	QUESTIONS & FILTERS	CODING CATEGORIES		SKIP TO
Q59931	*Why are you currently having more than one sexual partner?*	Multiple wives	1	
		Sex worker	2	
		Financial or material benefits / reduce poverty	3	
		Sexual pleasure	4	
		Emotional attachment (love)	5	
		Social status (impress peers etc.)	6	
		Other (specify)	7	
		Don't know	98	

Q59932* *Please tell me whether you strongly disagree, disagree, neither agree nor disagree, agree or strongly agree with the following statements …*

Statement	SD	D	N	A	SA
(1) I am confident I can stick to one partner if I wanted to.	1	2	3	4	5
(2) I am confident I can stick to one partner even if my friends encourage me to have other partners	1	2	3	4	5
(3) I am confident I can stick to one partner even if I lose financial or material benefits	1	2	3	4	5

REF.	QUESTIONS & FILTERS	CODING CATEGORIES		SKIP TO
Q59933	*If you wanted to stick to one partner, how easy would this be for you to do?*	Very easy	1	
	Ask for an answer on a scale of 5 - from 1 (very easy) to 5 (very difficult).	Easy	2	
		Neither easy nor difficult	3	
		Difficult	4	
		Very difficult	5	
Q59934	*What factors make it impractical or unsuitable for someone like you to stick to one partner?*		Spont / Probed	
	Ask first without probing and then probe for factors that are not mentioned spontaneously.	Multiple wives		
		Poverty		
		Threat of violence from partners		
		Peer pressure		
		Other (specify)		
Q59935	*How definitely do you want to stick to one partner in the near future?*	Definitely	1	
	Ask for an answer on a scale of 5 - from 1 (definitely) to 5 (definitely not).	Probably	2	
		No opinion	3	
		Probably not	4	
		Definitely not	5	
Q59936	*Do you plan to start sticking to one partner or to stop having sex?*	Yes - one regular partner	1	
	Stress: no right or wrong answer.	Yes - stop having sex	2	
		Neither	3	- Q59938
		Don't know	98	- Q59938
Q59937	*How soon do you plan to make this change?*	Number of months	mths	
Q59938	*What are the main reasons that would/did motivate you to stop having sex?*		Spont / Probed	
	Ask first without probing and then probe for factors that are not mentioned spontaneously.	Reduce risk of HIV & STDs (self/partner)		
		Reduce risk of pregnancy (self or partner)		
		Would feel a responsible person		
		Poor health		
		Children (daughters) now sexually active		
		Feel too tired / old		
		Other (specify)		
Q59939	*What are the main reasons that would/did discourage you from stopping having sex?*		Spont / Probed	
	Ask first without probing and then probe for factors that are not mentioned spontaneously.	Enjoy having sex		
		Desire/need to have (more) children		
		Partner would think I have HIV		
		Partner would leave me		
		Threat of violence from partner(s)		
		Friends would disapprove		
		Parents / family elders would disapprove		
		Community members would disapprove		
		Other (specify)		
Q59940	*What are the main reasons that would/did motivate you to stick to one partner?*		Spont / Probed	
	Ask first without probing and then probe for factors that are not mentioned spontaneously.	Reduce risk of HIV infection & STDs		
		Would feel a responsible person		
		Regular partner would approve		
		Parents / family elders would approve		
		Friends would approve		
		Other (specify)		
Q59941	*What are the main reasons that would/did discourage you from sticking to one partner?*		Spont / Probed	
	Ask first without probing and then probe for factors that are not mentioned spontaneously.	Sexual pleasure from having multiple partners		
		Loss of financial or material benefits		
		Loss of reputation / status in the community		
		Already married to multiple wives		
		Other (specify)		
Q59942	*Have you ever received training or counselling to reduce your number of sexual partners?*	Yes - organisation code (Q504)	1	
		No	2	
Q59943	*Would your sexual partner(s) accept it if you left him/her/them?*	Yes	1	
		No	2	

For peer review only

For peer review only - http://bmjopen.bmj.com/site/about/guidelines.xhtml

INDIVIDUAL QUESTIONNAIRE: **HIV AWARENESS & SOCIAL NORMS** ***Q. No:***

REF.	QUESTIONS & FILTERS	CODING CATEGORIES		SKIP TO
Q601	*"Now I would like to ask you some questions about HIV and AIDS …"*			
Q602	*Please tell me all the ways that an adult can get HIV infection?* **Tick 1 for each way mentioned spontaneously.** *Are there any other ways?* **Then proceed down the column, reading the description of each possible way not mentioned spontaneously. Make entries in "probed" column as follows:**<br>1/11 Yes, HIV can be transmitted this way (spont / yes when probed)<br>2/12 No, HIV cannot be transmitted this way (spont / yes when probed)<br>98 Don't know		Spont / Probed	
		**Sex with a person with HIV/AIDS**		
		**Touching a person with AIDS**		
		**Mosquito bites**		
		**Blood transfusion**		
		**Injection with a dirty needle**		
		**Sharing utensils with person with HIV/AIDS**		
		**Ritual scarification**		
		**Punishment from God**		
		**Other (specify)** ____________		
Q603	*Do you know of any factors which are likely to INCREASE the chances that a person will get HIV infection?*<br>**Ask in same way as Q602.**<br>Codes as in Q602.		Spont / Probed	
		**Sex with a prostitute**		
		**Many sex partners**		
		**Not being circumcised (for men)**		
		**Other STDs present**		
		**Using condoms**		
		**Witchcraft or spiritual curse**		
		**Other (specify)**		
Q604	*What are the ways in which an infant or child could have become infected with HIV?*<br>**Ask in same way as Q602.**<br>Codes as in Q602.		Spont / Probed	
		**At birth - if mother infected**		
		**Witchcraft or spiritual curse**		
		**Mosquito bites**		
		**Injection with a dirty needle**		
		**Breastfed by infected woman not on ART**		
		**Blood transfusion**		
		**From an infected father**		
		**Other (specify)**		
Q605	*Are ALL babies born to women who have HIV born with the infection?*	**Yes**	1	
		**No**	2	
		**Don't know**	98	
Q606	*Can all people infected with HIV be identified by looking at them?*	**Yes**	1	
		**No**	2	
		**Don't know**	98	
Q607	*Would you be willing to take care of a family member with AIDS?*	**Yes**	1	- Q609
		**No**	2	
		**Don't know**	98	
Q608	*Why would you not be willing to take care of a family member with AIDS?*	**Not enough time**	1	
		**Too few resources**	2	
		**Not enough space**	3	
		**Too young**	4	
		**Not experienced enough**	5	
		**Unwell myself**	6	
		**Frightened of being exposed to HIV**	7	
		**Reluctant to be associated with people living with HIV**	8	
		**Other (specify)**	12	
Q609	*How long does it usually take for a person infected with HIV to develop symptoms if they are not on ART?*	**Number of years or months**	yrs / mths	
		**Don't know**	998	
Q610	*How many people do you know who either died from HIV/AIDS or have the disease now?*	**Number (>0)**	mths	
		**Doesn't know of any**	998	
Q611	*Of these people, how many live(d) in your household; in the same village/town; and how many live(d) somewhere else?*<br>**Enter numbers of people in each category.**	**Household**		
		**Village / town**		
		**Somewhere else**		
		**Check that these sum to same as in Q510.**		

For peer review only

For peer review only - http://bmjopen.bmj.com/site/about/guidelines.xhtml

INDIVIDUAL QUESTIONNAIRE: **HIV AWARENESS & SOCIAL NORMS** **Q. No:**

REF.	QUESTIONS & FILTERS	CODING CATEGORIES		SKIP TO
Q612	*What was your relationship to each of these people?*<br><br>Enter numbers of people in each category.	Spouse/partner<br>Father or mother<br>Son or daughter<br>Other relative<br>Friend or neighbour<br>Work colleague<br>Someone else		
Q613	*Did you help to take care of any of these people on a daily basis?*	Yes<br>No	1<br>2	
Q614	*What are the chances that you are infected with HIV now?*	Certain / almost certain<br>High<br>Moderate<br>Small<br>None	1<br>2<br>3<br>4<br>5	
Q615	*What are the chances that you will become infected with HIV in the next 12 months if you are not infected now but continue with your current behaviour?*	Certain / almost certain<br>High<br>Moderate<br>Small<br>None	1<br>2<br>3<br>4<br>5	 <br><br><br>- Q617
Q616	*Why do you think you might become infected?*	Has multiple sex partners now<br>Not using condoms<br>Regular partner has other partners<br>Future partner may have other partners<br>Partner may be infected with HIV<br>Many friends/relatives dying of HIV/AIDS<br>Caring for an infected person<br>Other (specify)	1<br>2<br>3<br>4<br>5<br>6<br>7<br>8	

Q617 *For the following statements, please tell me whether you strongly disagree, disagree, neither agree nor disagree, agree or strongly agree …*

	SD	D	N	A	SA
(1) I can get HIV even if I only have sex with one	1	2	3	4	5
(2) I have never done anything that could have exposed me to HIV	1	2	3	4	5
(3) I would rather die a violent death (e.g. gunshot or car accident) than from HIV	1	2	3	4	5
(4) I feel vulnerable to HIV infection	1	2	3	4	5
(5) I am less likely to get HIV than my friends	1	2	3	4	5
(6) I'm worried that one of my current sexual partners may give me HIV	1	2	3	4	5
(7) I think about my chances of getting HIV whenever I have a new sexual partner	1	2	3	4	5
(8) Getting (a woman) pregnant when you don't want to is worse than getting infected with	1	2	3	4	5

REF.	QUESTIONS & FILTERS	CODING CATEGORIES		SKIP TO
Q618	*How often do you worry about getting infected with HIV?*	Never<br>Rarely<br>Sometimes<br>Often<br>Almost all the time	1<br>2<br>3<br>4<br>5	
Q619	*Who do you think is more likely to be infected with HIV in Manicaland: a 15-19 year-old man or a 25-29 year-old man?*	15-19 year-old man<br>25-29 year-old man	1<br>2	
Q620	*Who do you think is more likely to be infected with HIV in Manicaland: a 15-19 year-old woman or a 25-29 year-old man?*	15-19 year-old woman<br>25-29 year-old man	1<br>2	
Q621	*Would you buy fresh vegetables from a shopkeeper or vendor if you knew this person had HIV?*	Yes<br>No<br>Don't know/not sure/it depends	1<br>2<br>98	
Q622	*Do you think that children living with HIV should be able to attend school with children who are HIV negative?*	Yes<br>No<br>Don't know/not sure/it depends	1<br>2<br>98	
Q623	*If you thought your spouse/regular partner was having sexual intercourse with (a) casual partner(s) without using condoms, could you persuade him/her to stop?*	Yes<br>No<br>No regular partner<br>Don't know	1<br>2<br>96<br>98	

For peer review only - http://bmjopen.bmj.com/site/about/guidelines.xhtml

INDIVIDUAL QUESTIONNAIRE: **HIV AWARENESS & SOCIAL NORMS** *Q. No:*

REF.	QUESTIONS & FILTERS	CODING CATEGORIES		SKIP TO
Q624	*Do you think there are things you can do which will prevent you from becoming infected with HIV in the future?*	Yes	1	
		No	2	
		Don't know	98	
		NA - already infected	99	

Q625 *Which of these statements do you agree with?*

Statement	Agree	Disagree
(1) Men have a sex drive that needs to be satisfied	1	2
(2) A man will lose respect if he admits to being sick	1	2
(3) Men who take sick children to the hospital, or cook at home should be proud of what they do	1	2
(4) Men are strong and therefore less likely to need a doctor	1	2
(5) Men are always ready for sex	1	2
(6) A man should not go with his partner for antenatal check-ups at the local clinic	1	2
(7) If a man is sick, he should not let others see he is in pain	1	2
(8) It is appropriate for a woman to be the primary breadwinner of a household	1	2
(9) A real man enjoys a bit of risk taking now and then	1	2
(10) Men should have had several sexual partners before they get married	1	2

Read out each in turn.

Q626 *Which of these statements would you say was true?*

Statement	True	Not
(1) Minor illnesses can be fought off if you don't give in to it	1	2
(2) Men feel comfortable going to the hospital and have no problems seeking help	1	2
(3) There is no need to go and see a doctor unless you are very ill	1	2
(4) A man should make sure that he can recognise the early signs of an STI	1	2
(5) It is important for a man living with HIV to be on anti-retroviral therapy	1	2
(6) A man who goes to the hospital is considered weak	1	2
(7) A man gain respect if he goes for regular health checks	1	2
(8) Men get embarrassed if a brother is found to be HIV positive	1	2

Read out each in turn.

Q627 *Which of these statements do you agree with?*

Statement	Agree	Disagree
(1) It is a good idea to make condoms available for young people in schools	1	2
(2) If I have / had a teenage daughter, I would tell her about condoms	1	2
(3) If I have / had a teenage daughter, I would tell her about PrEP	1	2
(4) If I have / had a teenage daughter and thought she might be having sex, I would encourage her to use PrEP and condoms	1	2
	1	2
(5) If I have / had a teenage daughter and told her not to have sex until she gets married, she would comply	1	2
(6) If a young woman is married and her husband has HIV, she should use PrEP or condoms	1	2
(7) If I have / had a teenage daughter and she had sex before marriage, I would be OK with this	1	2
(8) Many young women have sex before marriage these days	1	2
(9) If I have / had a teenage son, I would encourage him to have medical circumcision	1	2
(10) I pay/get paid for sex because my friends do and because they encourage me	1	2

REF.	QUESTIONS & FILTERS	CODING CATEGORIES		SKIP TO
Q628	*Do you think a lot of young people are still getting infected with HIV these days?*	Yes	1	- Q630 if
		No	2	- male

Q629 *Have you experienced any of the following from a male intimate partner in the past 12 months?*

Item	Yes	No
(1) Slapped you or threw something at you that could hurt you	1	2
(2) Pushed or shoved you	1	2
(3) Hit you with a fist or something else that could hurt you	1	2
(4) Kicked or dragged you or beat you up	1	2
(5) Choked or burnt you	1	2
(6) Threatened or used a gun, knife or other weapon against you	1	2
(7) Physically forced you to have sexual intercourse against your will	1	2
(8) Forced you to do something sexual she found degrading or humiliating	1	2
(9) Made you afraid of what would happen if you did not have sexual intercourse	1	2

REF.	QUESTIONS & FILTERS	CODING CATEGORIES		SKIP TO
Q630	*Imagine that we flipped a fair coin 10 times. The first 9 times, the coin comes down 'heads'. What are the chances that the 10th flip will also be 'heads'?*	Percentage chance	%	

For peer review only - http://bmjopen.bmj.com/site/about/guidelines.xhtml

INDIVIDUAL QUESTIONNAIRE: **HIV AWARENESS & SOCIAL NORMS** **Q. No:**

REF.	QUESTIONS & FILTERS	CODING CATEGORIES	SKIP TO
Q631	I will ask you several questions about the chance or likelihood that certain events are going to happen. There are 10 beans in the cup. I would like you to choose some beans out of these 10 beans and put them in the plate to express what you think the likelihood or chance is of a specific event happening. One bean represents one chance out of 10. If you do not put any beans in the plate, it means you are sure that the event will NOT happen. As you add beans, it means that you think the likelihood that the event happens increases. For example, if you put one or two beans, it means you think the event is not likely to happen but it is still possible. If you pick 5 beans, it means that it is just as likely it happens as it does not happen (fifty-fifty). If you pick 6 beans, it means the event is slightly more likely to happen than not to happen. If you put 10 beans in the plate, it means you are sure the event will happen. There is no right or wrong answer, I just want to know what you think.<br><br>Let me give you an example. Imagine that we are playing draughts. Say, when asked about the chance that you will win, you put 7 beans in the plate. This means that you believe you would win 7 out of 10 games on average if we play for a long time.		
Q632	Report for each question the NUMBER OF BEANS put in the PLATE.<br>After each question, replace the beans in the cup (unless otherwise noted).<br><br>For questions Q633 to Q635: If respondent puts 10 (or 0) beans, prompt:<br>*"Are you sure that this event will almost certainly (not) happen?"*<br><br>CIRCLE 1 in column P if you prompted the respondent, and report the final answer only.		
Q633	*A baby in your community will die before his or her first birthday?*	# beans on plate: ☐ prompt (0 or 10): 1	
Q634	*You will go to the market at least once within the next 2 days?*<br><br>LEAVE BEANS IN PLATE.	☐ 1	
Q635	*You will go to the market at least once within the next 2 weeks?*	☐ 1	- Q637 if beans added
Q636	*Remember, as time goes by, you may find more time to go to the market. Therefore, you should have added beans to the plate. Let me ask you again. Now, add beans in the plate so that the number of beans in the plate reflects how likely you think it is that you will go to the market at least once within the next 2 weeks?*	☐ 1	
Q637	*Please pick the number of beans that reflects how likely you think it is that …*<br><br>*You are infected with HIV now?*	# beans on plate: ☐	- Q640 if unmarried
Q638	*Your spouse is infected with HIV now?*	☐	
Q639	*You will use a condom next time you have sex with your spouse / long-term partner?*<br><br>If no regular sexual partner(s), write '99'.	☐	
Q640	*You will use a condom next time you have sex with a non-regular partner?*<br><br>If no non-regular sexual partner(s), write '99'.	☐	- Q701 if '99'
Q641	*Your current / most recent non-regular sexual partner is infected with HIV now?*<br><br>If no non-regular sexual partner(s), write '99'.	☐	
Q642	*You will use a condom next time you have sex with a non-regular partner?*<br><br>If no non-regular sexual partner(s), write '99'.	☐	
Q643	*You will use a condom next time you have sex with someone for the first time?*	☐	

For peer review only

INDIVIDUAL QUESTIONNAIRE: **HEALTH & ACCESS TO TREATMENT** ***Q. No:***

REF.	QUESTIONS & FILTERS	CODING CATEGORIES	SKIP TO
Q701	*Has a medical doctor or nurse ever diagnosed you with the following conditions?*	(see grid below)	

Condition	Yes	No	DK	Year	Tx	Pd
High blood pressure	1	2	98			
Diabetes	1	2	98			
High cholesterol	1	2	98			
Kidney diseases	1	2	98			
Heart diseases (incl. stroke)	1	2	98			
Depression	1	2	98			
Osteoporosis	1	2	98			
Tuberculosis	1	2	98			
Malaria	1	2	98			
Schistosomiasis	1	2	98			
Hepatitis B	1	2	98			
Hepatitis C	1	2	98			
Pregnancy/delivery complications	1	2	98			
Human papilloma virus	1	2	98			
Cancer (specify type)	1	2	98			
Genital herpes	1	2	98			
HIV infection	1	2	98			

REF.	QUESTIONS & FILTERS	CODING CATEGORIES	CODE	SKIP TO
Q702	For EACH condition mentioned in Q701, record the year of first diagnosis in the 4th column above.			
Q703	For EACH condition mentioned in Q701, record details of the institution providing the treatment in the 5th column (Tx) using the following codes:	Public sector healthcare provider	1	
		Church Mission healthcare provider	2	
		Private sector provider	3	
		NGO or academic research institution	4	
		Other	8	
		Not currently receiving treatment	99	
Q704	For EACH condition mentioned in Q701, record details of who paid for the medical fees and the costs of the drugs in the 6th column (Pd) using the following codes:	Private medical insurance only	1	
		Private medical insurance partly	2	
		Respondent and/or family	3	
		Other	8	
		Service was free	99	
Q705	*How long is it since you last experienced an illness?*	Days/weeks	days / weeks	
		More than one year ago	97	- Q714
Q706	*What was the main symptom of the illness?*	Fever - malaria (incl. cerebral)	1	
		Fever - non-malaria	2	
		Sickness/vomiting	3	
		Diarrhoea/weight loss	4	
		Swollen lymph nodes	5	
		Skin complaints/rashes	6	
		Genital conditions: incl. STDs	7	
		Flu/pneumonia	8	
		Accident/wound	9	
		Tuberculosis	10	
		Other (specify)	12	
Q707	*How long was it between the time when you first noticed symptoms and when you first sought help for this illness?*	Days and weeks	days / weeks	
		Did not seek assistance	97	- Q709
Q708	*What is the main reason you have not sought treatment?*	Costs of treatment/travel too high	1	- Q714
		Too little time to travel / wait for treatment	2	- Q714
		Do not feel that anyone can help	3	- Q714
		Able to cope myself / illness not serious	4	- Q714
		Fear of stigma	5	- Q714
		Other (specify)	8	- Q714
Q709	*Which of the following people did you visit or consult for this illness?* Read the options and tick all that apply.		Yes / No	
		Medical doctor or nurse	1 / 2	- Q711
		N'anga	1 / 2	- if not
		Faith healer	1 / 2	- n'anga or
		Other (specify)	1 / 2	- faith hlr
Q710	*Did you seek assistance from the n'anga (or faith healer) for any of these reasons?* Read the options and tick all that apply.		Yes / No	
		Clinic treatment not effective	1 / 2	- Q714
		Spiritual cure needed	1 / 2	- if did
		Witchcraft suspected	1 / 2	- not also
		Holy water/bath or prayer	1 / 2	- visit
		More confidential than clinic	1 / 2	- hospital
		More convenient to visit than clinic	1 / 2	- or
		Less expensive to visit than clinic	1 / 2	- clinic
		Other (specify)	1 / 2	-
Q711	*How long did it take you to travel from your home to the clinic/hospital?*	Minutes<br>Convert hours to minutes if necessary.	mins	
Q712	*How long did you have to wait before you were seen by a doctor or nurse?*	Minutes<br>Convert hours to minutes if necessary.	mins	

For peer review only

For peer review only - http://bmjopen.bmj.com/site/about/guidelines.xhtml

INDIVIDUAL QUESTIONNAIRE: **HEALTH & ACCESS TO TREATMENT** **Q. No:**

REF.	QUESTIONS & FILTERS	CODING CATEGORIES	SKIP TO
Q713	*Did you receive an HIV test as part of your treatment for this illness?*	Yes 1<br>No or don't know 2	
Q714	For men:<br>*Some men experience white, transparent or yellowish discharge from the penis, which might not cause discomfort or may be accompanied by mild burning paid on urination. In the last 12 months, have you had these symptoms?*<br>For women:<br>*Some women experience an unusual discharge from the vagina or pain in the lower stomach. In the last 12 months, have you had these symptoms?*	Yes - discharge & pain (both) 1<br>Yes - discharge only 2<br>Yes - pain only 3<br>No 4<br>Don't know 98<br>Clarify which & tick boxes as appropriate.	
Q715	*Some (wo)men experience sores in the genital area. During the last 12 months, have you noticed any such sores?*	Yes 1<br>No 2<br>Don't know 98	- Q717<br>- if not had<br>- symptoms
Q716	*What happened when you were treated at the health centre for these symptoms?*<br>Read the options and tick all that apply.	(Yes / No)<br>Not applicable - did not get treated at a clinic 99<br>Physical symptoms were inspected 1 / 2<br>Samples taken & tests done 1 / 2<br>HIV test was offered 1 / 2<br>Counseling provided 1 / 2<br>Free condoms provided 1 / 2<br>Symptoms went away after treatment 1 / 2	
Q717	*On how many different occasions have you had an HIV test and received the results: (i) in your lifetime; and (ii) in the last 3 years?*<br>Stress that these questions do *not* include self-tests.	Lifetime<br>Last 3 years	- If "0" go to Q725
Q718	*How long is it since you last had an HIV test?*	Period (mths / yrs)	
Q719	*Did you receive counselling before you agreed to have the test?*<br>Explain what is meant by counselling.	Yes 1<br>No 2	
Q720	*Was the result of this HIV test positive?*<br>Stress that do not have to answer these questions but information is confidential.	Yes 1<br>No 2<br>Don't know 98<br>Prefers not to say 99	
Q721	*After the test, did you receive counselling and referrals for the following HIV prevention methods?*<br>Read the options and tick all that apply.<br>Don't ask about VMMC or PrEP if respondent is HIV-positive.	(Counselling Yes / No; Referrals Yes / No)<br>VMMC 1 / 2; 1 / 2<br>PrEP 1 / 2; 1 / 2<br>Male condoms 1 / 2; 1 / 2<br>Female condoms 1 / 2; 1 / 2<br>Being faithful to one partner 1 / 2; 1 / 2<br>Not having sex 1 / 2; 1 / 2<br>Treatment as prevention 1 / 2; 1 / 2	
Q722	*What is the name of the organisation that provided this HIV testing service?*	PSI - New Start 1<br>FACT 2<br>FHI360 3<br>Hospital/clinic (ANC/PMTCT) 4<br>Hospital/clinic (other) 5<br>Other (specify) 8<br>Don't know 98	
Q723	*After the HIV test, did you:*<br>(1) Use condoms more or less than before?<br>(2) Start having more or fewer sexual partners?	(More / Same / Less)<br>(1) 1 / 2 / 3<br>(2) 1 / 2 / 3	
Q724	*Did you join a post-test club or a group for people living with HIV and, if so, are you still a member?*<br>If HIV test result was +ve, go to Q737.	(Still / Was / Never)<br>Post-test club 1 / 2 / 3<br>PLWHA 1 / 2 / 3<br>Neither 8	
Q725	*Please tell me whether you strongly disagree, disagree, neither agree nor disagree, agree or strongly agree with the following statements …*<br>(1) I am confident I can get tested for HIV if I wanted tc<br>(2) I am confident I can get tested for HIV even if the healthworker cannot keep the results confidential<br>(3) I am confident I can get tested for HIV even if my partner disapproves<br>(4) I am confident I can get tested for HIV even if I am afraid to be HIV+<br>(5) I am confident I can get tested for HIV even if I was stigmatised in my community if I was known or suspected to be HIV+	(SD / D / N / A / SA)<br>(1) 1 / 2 / 3 / 4 / 5<br>(2) 1 / 2 / 3 / 4 / 5<br>(3) 1 / 2 / 3 / 4 / 5<br>(4) 1 / 2 / 3 / 4 / 5<br>(5) 1 / 2 / 3 / 4 / 5	

For peer review only

INDIVIDUAL QUESTIONNAIRE: **HEALTH & ACCESS TO TREATMENT** **Q. No:**

REF.	QUESTIONS & FILTERS	CODING CATEGORIES		SKIP TO
Q726	*Are you able to discuss HIV testing with your partner?*	Yes - already done so<br>Yes - not done already but can do this<br>No<br>No current partner	1<br>2<br>3<br>99	
Q727	*Would your partner disapprove if you got tested for HIV?*	Yes<br>No<br>Don't know	1<br>2<br>98	
Q728	*Would your partner come with you to get tested for HIV?*	Yes - already done so<br>Yes - not done already but can do this<br>No<br>Don't know	1<br>2<br>3<br>98	
Q729	*If / when you want to have an(other) HIV test, do you know a place where someone like you can easily get it?*	Yes<br>No	1<br>2	
Q730	*If / when you want to have an(other) HIV test yourself, how easy is it for you to access the service?*<br><br>Ask for an answer on a scale of 5 - from 1 (very easy) to 5 (very difficult).	Very easy<br>Easy<br>Neither easy nor difficult<br>Difficult<br>Very difficult<br>Don't know	1<br>2<br>3<br>4<br>5<br>98	
Q731	*What factors make it difficult or unsuitable for someone like you to get access to HIV testing services?*<br><br>Ask first without probing and then probe for factors that are not mentioned spontaneously.	High costs<br>Judgemental staff<br>Lack of privacy or confidentiality<br>Limited opening hours<br>Distance / travel difficulties<br>Not appropriate for me to go there<br>Other (specify)	Spont / Probed	
Q732	*How far away is it from where you live to the nearest place where HIV tests are provided?*	Distance in kms	kms	
Q733	*Do you want to have an HIV test if it was freely accessible to you?*	Yes<br>No<br>Not sure	1<br>2<br>8	
Q734	*How definitely do you want to have an HIV test if it was freely accessible to you?*<br><br>Ask for an answer on a scale of 5 - from 1 (definitely) to 5 (definitely not).	Definitely<br>Probably<br>No opinion<br>Probably not<br>Definitely not	1<br>2<br>3<br>4<br>5	
Q735	*Do you plan to have an(other) HIV test?*	Yes<br>No<br>Don't know	1<br>2<br>98	 - Q737<br>- Q737
Q736	*How soon do you plan to have an(other) HIV test?*	Number of months	mths	
Q737	*What are the main reasons that woud/did motivate you to go for an HIV test?*<br><br>Ask first without probing and then probe for factors that are not mentioned spontaneously.	To access HIV treatment if I need it<br>Wanted re-assurance not infected<br>Avoid infecting partner(s)<br>Partner or child sick or died<br>Past risky behaviour<br>Partner's risky behaviour<br>Many friends going for HIV tests<br>Encouraged by family<br>Reassure partner<br>Contemplating marriage<br>Contemplating having a child<br>Prevent mother-to-child infection<br>Spouse/partner tested HIV+<br>Doctor/nurse suggested it<br>Life planning<br>Other (specify)	Spont / Probed	
Q738	*What are the main reasons that would/did discourage you from having an HIV test?*<br><br>Ask first without probing and then probe for factors that are not mentioned spontaneously.	Psychological effects<br>Stigma & discrimination<br>Possible divorce/separation<br>Job loss<br>Positive result accelerates death<br>Fear - of being HIV+<br>Fear - of partner violence<br>Lack of confidentiality<br>Too expensive<br>Other (specify)	Spont / Probed	

For peer review only

For peer review only - http://bmjopen.bmj.com/site/about/guidelines.xhtml

INDIVIDUAL QUESTIONNAIRE: **HEALTH & ACCESS TO TREATMENT** *Q. No:*

REF.	QUESTIONS & FILTERS	CODING CATEGORIES		SKIP TO
Q739	*If you have or had a daughter or son who is a teenager or a young adult and she/he started having sex, do you think it would be a good thing for her/him to have an HIV test?*	Yes<br>No<br>Don't know	Teen / YA<br>1 / 1<br>2 / 2<br>98 / 98	
Q740	Explain that there are HIV tests that you can do yourself.<br>*How long is it since you last had an HIV self-test?*	Period<br>Never had a self-test<br>Not heard of HIV self-tests	mths / yrs<br>97<br>99	 - Q743<br>- Q744
Q741	*Was the result of this HIV self-test positive or negative?*<br>Stress that do not have to answer these questions but information is confidential.	Positive<br>Negative<br>Don't know<br>Prefers not to say	1<br>2<br>98<br>99	
Q742	*Did you get this result confirmed at a health facility or other HIV testing facility?*<br>Stress that do not have to answer these questions but information is confidential.	Yes - result confirmed<br>No - went but result was different<br>No - didn't go for confirmation test<br>Prefers not to say	1<br>2<br>3<br>99	
Q743	*If / when you want to do an HIV self-test, do you know a place where someone like you can easily get a test kit?*	Yes<br>No	1<br>2	
Q744	*Have you heard of antiretroviral therapy (ART) - the drugs that prevent HIV from causing AIDS?*	Yes<br>No	1<br>2	 - Q748
Q745	*How far is it from here to the nearest place where these drugs can be obtained?*	Distance<br>Don't know a place	kms<br>98	 - Q748
Q746	*What type of place is this?*<br>"Roadside" here means a tarred road.<br>Record name of place..	Large town or city<br>Small town<br>Growth point<br>Commercial estate/mine<br>Roadside business centre<br>Rural business centre<br>Communal / resettlement area	1<br>2<br>3<br>4<br>5<br>6<br>7	
Q747	*How long does it take to travel from your home to this place?*<br>Convert hours to minutes if necessary.	Minutes	mins	
Q748	*Have you ever taken ART yourself?*<br>Check that not taking something that could be ART	Yes<br>No	1<br>2	- Q750
Q749	*What is the main reason you have not started taking these drugs?*	Costs too high<br>Not available locally<br>Not permitted by church<br>Side effects<br>Not needed: in good health<br>Not needed: HIV-<br>Other (specify)<br>Don't know	1<br>2<br>3<br>4<br>5<br>6<br>8<br>98	- Q801<br>- Q801<br>- Q801<br>- Q801<br>- Q801<br>- Q801<br>- Q801<br>- Q801
Q750	*How long is it since you first took these drugs?*	Period	weeks / years	
Q751	*What motivated you to start taking ART?*	HIV+: unwell<br>HIV+: well but wanted to remain healthy<br>Other (specify)	1<br>2<br>8	
Q752	*Have you stopped taking the drugs?*	Yes<br>No	1<br>2	 - Q754
Q753	*Why have you stopped taking the drugs?*	Costs too high<br>Not available locally<br>Side effects<br>Not needed: in good health<br>Other (specify)<br>Don't know	1<br>2<br>3<br>4<br>8<br>98	- Q756<br>- Q756<br>- Q756<br>- Q756<br>- Q756<br>- Q756
Q754	*Are there particular times when you take the drugs?*	All the time<br>When feeling unwell<br>When can afford or paid for<br>Other (specify)	1<br>2<br>3<br>8	
Q755	*Do you sometimes forget to take the drugs?*	Never<br>Occasionally<br>Quite often	1<br>2<br>3	
Q756	*How often have you taken ART in the last month?*	Every day<br>Most days<br>Occasionally<br>Not at all	1<br>2<br>3<br>4	

For peer review only

For peer review only - http://bmjopen.bmj.com/site/about/guidelines.xhtml

INDIVIDUAL QUESTIONNAIRE:	HEALTH & ACCESS TO TREATMENT	*Q. No:*

REF.	QUESTIONS & FILTERS	CODING CATEGORIES	SKIP TO
Q757	*After you started taking the drugs, did you:*<br>(1) Start having more or fewer sexual partners?<br>(2) Use condoms more or less than before?	More / Same / Less<br>(1) 1 / 2 / 3<br>(2) 1 / 2 / 3	
Q758	*Have you experienced any unpleasant side effects since you started the treatment?*	Yes 1<br>No 2	
Q759	*Have you (also) been receiving treatment from a traditional healer or faith healer for your HIV infection?*	Traditional healer 1<br>Faith healer 2<br>Neither 3	

FORM B

For peer review only

INDIVIDUAL QUESTIONNAIRE: **FERTILITY HISTORIES** *Q. No:*

REF.	QUESTIONS & FILTERS	CODING CATEGORIES		SKIP TO
Q801	*Now I would like to talk to you about pregnancy and childbirth. Have you ever given birth (fathered a child)*?*	Yes<br>No	1<br>2	 - Q806
Q802	*Do you have any sons or daughters who are living with you now?*	Yes<br>No	1<br>2	 - Q804
Q803	*How many sons live with you? How many daughters live with you?*	Sons at home<br>Daughters at home		
Q804	*Do you have any sons or daughters who are alive but do not live with you?*	Yes<br>No	1<br>2	 - Q806
Q805	*How many sons are alive but do not live with you? How many daughters are alive but do not live with you?*	Sons elsewhere<br>Daughters elsewhere		
Q806	*Have you ever given birth to (or fathered) a boy or girl who was born alive but later died?*<br>If no, probe:<br>*Any (other) boy or girl who cried or showed any sign of life but only survived a few hours or days?*	Yes<br>No	1<br>2	 - Q808
Q807	*How many boys have died in this way? And how many girls died in this way?*	Boys who died<br>Girls who died		
Q808	Sum answers to Q803, Q805 and Q807. Enter total.	Total		
Q809	*In total, then, how many live births have you had (fathered)?*<br>Compare response with total in Q708. If numbers are different, probe and correct Q801-Q808, as necessary.			- for men, go to Q901
Q810	*Have you had a live birth in the last year?*	Yes<br>No	1<br>2	
Q811	*Have you been pregnant in the last year?*	Yes<br>No	1<br>2	

*Note: For male respondents use question wordings indicated in brackets.

For peer review only - http://bmjopen.bmj.com/site/about/guidelines.xhtml

INDIVIDUAL QUESTIONNAIRE: **RECENT PREGNANCY HISTORY** **Q. No:**

REF.	QUESTIONS & FILTERS	CODING CATEGORIES		SKIP TO
Q901	*Are you pregnant at the moment?*	Yes<br>No	1<br>2	 - Q903
Q902	*How many months pregnant do you think you are?*<br>Ask for best estimate.	Number of months		- Q905
Q903	*Have you ever had a pregnancy that ended in a live birth, miscarriage or still-birth?*	Yes<br>No<br>Not sure	1<br>2<br>98	 - Q1001<br>- Q1001
Q904	*When did your most recent such pregnancy end?*<br>Stress: including miscarriages.		mnth yr	
Q905	*At the time you became pregnant, did you want to become pregnant then, did you want to wait until later, or did you not want to become pregnant at all?*	Then<br>Later<br>Not at all	1<br>2<br>3	
Q906	*Did you see anyone for antenatal care during this pregnancy?*	Yes<br>No	1<br>2	 - Q923
Q907	*After how many months of the pregnancy did you first go for an antenatal check-up?*	Months		
Q908	*Did you have an HIV test while attending for ANC check-ups for this pregnancy or did you already know your status?*<br>If knew HIV+, ask if she was on ART.	Yes<br>No: already knew HIV+ - on ART<br>No: already knew HIV+ - not on ART<br>No: did not want	1<br>2<br>3<br>4	
Q909	*Did your husband (or pregnancy partner) also attend for PMTCT with you and have an HIV test during this pregnancy?*	Yes<br>No: already knew HIV+<br>No: did not want	1<br>2<br>3	
Q910	*Did YOU have HIV infection at the time of this pregnancy?*<br>Stress confidential but voluntary.	Yes<br>No<br>Don't know<br>No response	1<br>2<br>8<br>9	 - Q917<br>- Q917<br>- Q917
Q911	*Did the clinic provide you with any counselling on family planning for people with HIV?*	Yes<br>No<br>Don't recall	1<br>2<br>8	
Q912	*Did you receive any treatment to take YOURSELF to prevent the baby from getting infected? If so, what type?*<br>Stress that this is treatment taken by the mother herself.	Yes: already on ART<br>Yes: initiated on ART during pregnancy<br>Yes: other (specify)<br>__________<br>No	1<br>2<br>3<br><br>4	- Q915<br><br>- Q915<br><br>- Q915
Q913	*Are you still taking ART now?*	Yes<br>No	1<br>2	- Q915
Q914	*Why did you decide to stop taking ART?*<br>__________	No longer breastfeeding the baby<br>Healthy so no need for ART<br>Side effects<br>Other (specify)<br>Don't know	1<br>2<br>3<br><br>98	

For peer review only - http://bmjopen.bmj.com/site/about/guidelines.xhtml

INDIVIDUAL QUESTIONNAIRE: **RECENT PREGNANCY HISTORY** **Q. No:**

REF.	QUESTIONS & FILTERS	CODING CATEGORIES		SKIP TO
Q915	*Did the baby receive any of these forms of treatment to prevent him/her from getting infected with HIV?*	ART (i.e. continuous)<br>NVP syrup<br>Other (specify)	Y N<br>1 2<br>1 2<br>1 2	
Q916	*Did you or the baby ever stop or miss taking the treatment you were given?*	Yes<br>No<br>Baby died before completed treatment	1<br>2<br>3	
Q917	*Was the baby delivered at a clinic/hospital or at home?*	Clinic<br>Home	1<br>2	
Q918	*Did this pregnancy end in a miscarriage, abortion or stillbirth?*	Yes<br>No	1<br>2	 - Q920
Q919	*How many months pregnant were you when this pregnancy ended?*		mths	
Q920	*Did the baby ever have an HIV test?*<br>If yes, ask for result.<br>Stress confidential but voluntary.	Yes: infected<br>Yes: uninfected<br>No<br>DK or rather not say	1<br>2<br>3<br>8	
Q921	*Has the baby been initiated on ART?*	Yes: started & still taking<br>Yes: started but since stopped<br>No<br>Don't know	1<br>2<br>3<br>8	
Q922	*Has the baby received medical male circumcision?*	Yes: medical circumcision<br>No: but traditional circumcision<br>No: not circumcised at all (yet)<br>No: baby is a girl	1<br>2<br>3<br>99	- Q927<br><br><br>- Q927
Q923	*Would you have liked the baby to have received medical male circumcision?*	Yes<br>No	1<br>2	
Q924	*Were medical male circumcision services for infants available in your area at the time this baby was born?*	Yes<br>No	1<br>2	
Q925	*Is the baby still alive?*	Yes<br>No	1<br>2	- Q929
Q926	*How old was the baby when he/she passed away?*	Convert to months.<br><1m = '0' months.	mths	
Q927	*Did you ever feed this baby at the breast?*	Yes<br>No	1<br>2	 - Q1001
Q928	*Are you still breastfeeding?*	Yes<br>No<br>Child has died	1<br>2<br>99	- Q1001
Q929	*For how long did you breastfeed this baby?*<br>Exclusively? (i.e. no liquids or solids)<br>In total?<br>If total > 6 mths, go to Q1001.	 Months<br>Months		
Q930	*Why did you not breastfeed this baby (for longer)?*	Baby sick or died<br>Mother sick<br>Risk of HIV<br>Pregnant<br>Resumed sex<br>Other	1<br>2<br>3<br>4<br>5<br>8	

FORM B

For peer review only - http://bmjopen.bmj.com/site/about/guidelines.xhtml

INDIVIDUAL QUESTIONNAIRE: **CONCLUSION** *Q. No:*

REF.	QUESTIONS & FILTERS			
Q1001	*For how much of the last 3 years have you and your regular partner been using a method of contraception?*	None	1	- Q1004
		Some of the time	2	
		Most/all of the time	3	
		Not sure	98	
Q1002	*Which of these methods were the main methods you used?*<br>Ask about each method in turn.		Y N	
		Pill	1 2	
		Injections	1 2	
		Condoms	1 2	
		Femidoms	1 2	
		Sterilization	1 2	
		Safe period	1 2	
		Withdrawal	1 2	
		Other (specify)	1 2	
Q1003	*What were your main reasons for wanting to delay or prevent another pregnancy?*<br>Probe for other reasons, but do not prompt.	Enough children	1	
		Birth spacing	2	
		Child HIV+ risk	3	
		Child orphan risk	4	
		Mother HIV+: accelerate AIDS	5	
		Not yet ready to have children	6	
		Other (specify)	8	
Q1004	Record current time.	Hour and minutes	hr mins	
Q1005	Record contact details for follow-up interviews if required.	Cell number (1)		
		Cell number (2)		
Q1006	*What are your views of the value of this research?*	Useful	1	
		Do not see the point	2	
		No opinion	98	
Q1007	Record respondent's comments and your own observations in the space below.<br>Remind respondent of arrangements for the BE procedures (where relevant) and for HIV testing.			

RESPONDENT'S COMMENTS:

On the research?

Further HIV prevention, care and support activities needed?

ENUMERATOR'S OBSERVATIONS:

FORM B

For peer review only - http://bmjopen.bmj.com/site/about/guidelines.xhtml

STROBE Statement—Checklist of items that should be included in reports of ***cross-sectional studies***

	Item No	Recommendation	Page No
**Title and abstract**	1	(*a*) Indicate the study's design with a commonly used term in the title or the abstract	1
		(*b*) Provide in the abstract an informative and balanced summary of what was done and what was found	2
**Introduction**			
Background/rationale	2	Explain the scientific background and rationale for the investigation being reported	4-5
Objectives	3	State specific objectives, including any prespecified hypotheses	5
**Methods**			
Study design	4	Present key elements of study design early in the paper	5-6
Setting	5	Describe the setting, locations, and relevant dates, including periods of recruitment, exposure, follow-up, and data collection	5-6
Participants	6	(*a*) Give the eligibility criteria, and the sources and methods of selection of participants	5-6
Variables	7	Clearly define all outcomes, exposures, predictors, potential confounders, and effect modifiers. Give diagnostic criteria, if applicable	7-9
Data sources/ measurement	8*	For each variable of interest, give sources of data and details of methods of assessment (measurement). Describe comparability of assessment methods if there is more than one group	7-9
Bias	9	Describe any efforts to address potential sources of bias	5-9
Study size	10	Explain how the study size was arrived at	6
Quantitative variables	11	Explain how quantitative variables were handled in the analyses. If applicable, describe which groupings were chosen and why	6-9
Statistical methods	12	(*a*) Describe all statistical methods, including those used to control for confounding	6-9
		(*b*) Describe any methods used to examine subgroups and interactions	7-9
		(*c*) Explain how missing data were addressed	6-9
		(*d*) If applicable, describe analytical methods taking account of sampling strategy	6
		(*e*) Describe any sensitivity analyses	9
**Results**			
Participants	13*	(a) Report numbers of individuals at each stage of study—eg numbers potentially eligible, examined for eligibility, confirmed eligible, included in the study, completing follow-up, and analysed	10 and Fig. 1
		(b) Give reasons for non-participation at each stage	10 and Fig. 1
		(c) Consider use of a flow diagram	Fig. 1
Descriptive data	14*	(a) Give characteristics of study participants (eg demographic, clinical, social) and information on exposures and potential confounders	10-12
		(b) Indicate number of participants with missing data for each variable of interest	11-12
Outcome data	15*	Report numbers of outcome events or summary measures	12-17

For peer review only

Main results	16	(*a*) Give unadjusted estimates and, if applicable, confounder-adjusted estimates and their precision (eg, 95% confidence interval). Make clear which confounders were adjusted for and why they were included	12-17
		(*b*) Report category boundaries when continuous variables were categorized	12-17
		(*c*) If relevant, consider translating estimates of relative risk into absolute risk for a meaningful time period	NA
Other analyses	17	Report other analyses done—eg analyses of subgroups and interactions, and sensitivity analyses	12-17 and Fig. 2
**Discussion**			
Key results	18	Summarise key results with reference to study objectives	17
Limitations	19	Discuss limitations of the study, taking into account sources of potential bias or imprecision. Discuss both direction and magnitude of any potential bias	18-20
Interpretation	20	Give a cautious overall interpretation of results considering objectives, limitations, multiplicity of analyses, results from similar studies, and other relevant evidence	17-18
Generalisability	21	Discuss the generalisability (external validity) of the study results	17-19
**Other information**			
Funding	22	Give the source of funding and the role of the funders for the present study and, if applicable, for the original study on which the present article is based	21

*Give information separately for exposed and unexposed groups.

Note: An Explanation and Elaboration article discusses each checklist item and gives methodological background and published examples of transparent reporting. The STROBE checklist is best used in conjunction with this article (freely available on the Web sites of PLoS Medicine at http://www.plosmedicine.org/, Annals of Internal Medicine at http://www.annals.org/, and Epidemiology at http://www.epidem.com/). Information on the STROBE Initiative is available at www.strobe-statement.org.

For peer review only

For peer review only - http://bmjopen.bmj.com/site/about/guidelines.xhtml
